# Regioselective C‑Arylation of Functionalized Nitroalkanes with Furan, Thiophene, and Substituted Thiophenes

**DOI:** 10.1021/acs.joc.5c02410

**Published:** 2025-11-21

**Authors:** Katarína R. Detková, Kristína Jakubcová, Tomáš Malatinský, Juraj Filo, Marek Cigáň, Šimon Budzák, Miroslav Medved’, Pavol Jakubec

**Affiliations:** † Department of Organic Chemistry, 61791 Slovak University of Technology, Radlinského 9, Bratislava 1, Bratislava 811 07, Slovakia; ‡ Department of Organic Chemistry, Faculty of Natural Sciences, 164047 Comenius University, Ilkovičova 6, Bratislava SK-842 15, Slovakia; § Department of Chemistry, Faculty of Natural Sciences, Matej Bel University, Tajovského 40, Banská Bystrica SK-97400, Slovakia; ∥ Regional Centre of Advanced Technologies and Materials, Czech Advanced Technology and Research Institute (CATRIN), Palacký University Olomouc, Šlechtitelů 241/27, Olomouc 783 71, Czech Republic

## Abstract

A user-friendly, highly regioselective C-arylation of nitroalkanes with thiophenes and furan, employing a fascinating one-pot cascade process, has been developed. The formal C–H activation proceeds under mild conditions and follows a well-understood reaction mechanism supported by both experimental data and thorough theoretical investigation.

## Graphical abstract

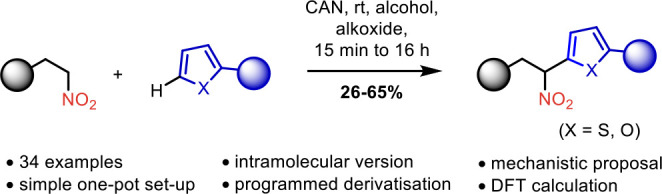

## Introduction

The relentless pursuit of new methodologies to create functionalized molecules of high structural complexity from simple and accessible starting materials remains one of modern chemistry’s primary goals and challenges. Driven by the stringent economic and ecological demands, novel practical methods are expected to provide predictable reactivity patterns with high selectivity and build complexity in a single operation. Herein, we describe the discovery of novel, practical chemo- and regioselective C-arylation of functionalized nitroalkanes with bulk heterocyclic compounds, providing straightforward access to functionalized nitroalkylated furans and thiophenes. C-Arylation of nitroalkanes has emerged as a synthetic methodology to create a bond between two essential building blocks of organic chemistry—arenes and nitroalkanes. Considering the well-known and predictable individual chemical behavior of arenes^1^ and the nitro group,^2–4^ arylation has immense synthetic potential in the synthesis of complex molecules. The existing general strategies for arylations of nitroalkanes developed so far rely on prefunctionalized aromatic compounds as the reaction partners (Scheme 1, part A). Early pioneering work employed aryl iodonium salts^5–8^ and organometallics, with organobismuth,^9^ organolead,^10^ organomercury,^11^ and organothallium compounds^12^ frequently utilized.

**Scheme 1:**
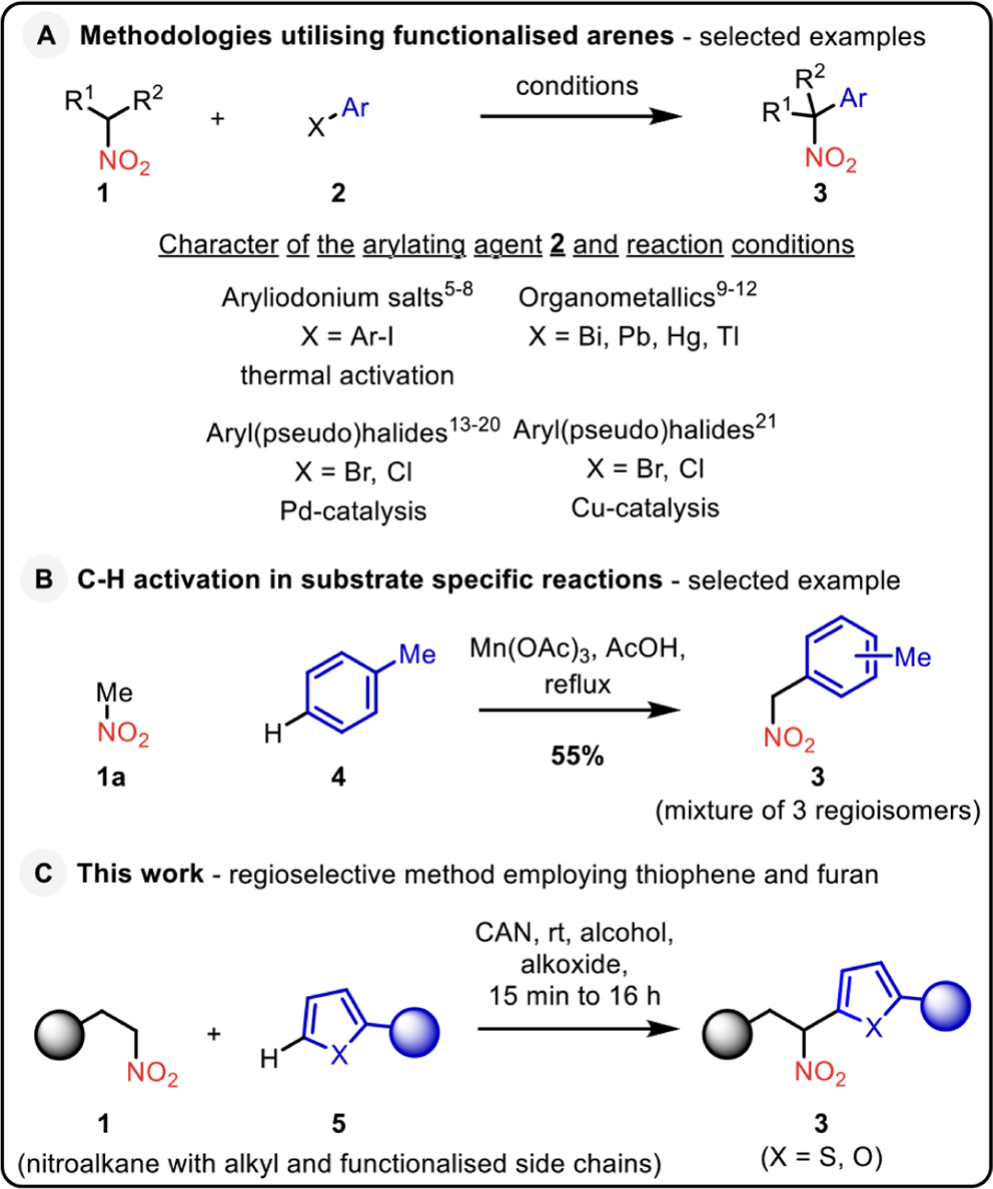
State-of-the-art in the C-Arylation of Nitroalkanes

Recent developments have culminated in the merging of transition metal catalysis and haloarenes reactivity.^13–21^ Direct arylation with arenes, formally C–H activation,^22^ has been limited to certain substrate-specific reactions (Scheme 1, part B).^23,24^ Compared to methods employing prefunctionalized arenes, this approach often lacks the necessary regioselectivity and generality.

## Results and Discussion

The surprising lack of methods for the direct preparation of highly valuable C-heteroarylated products from heterocycles inspired us to investigate this reaction using readily available electron-rich heteroaromatic bulk raw materials. Our study demonstrates a novel strategy for the regioselective synthesis of nitroalkylated thiophene, functionalized thiophenes, and furan (Scheme 1, part C). The proposed design relies on the practical transformation of nitroalkanes into highly reactive α-nitroalkyl radicals,^25–28^ which react with readily available electron-rich heterocycles to form arylated products through a cascade of reactions. Initially, feasibility studies were conducted using representative nitroalkane **1b** (1 equiv) and bulk chemical thiophene (**5a**) (Table 1). The phenyl-substituted nitroalkane **1b** was selected due to the distinctive NMR spectrum of the desired product **3a** (Table 1) and its lower volatility. Inspired by the work of Arai and Narasaka,^25^ potassium *tert*-butoxide in methanol was chosen for the in situ formation of nitronate **6a** achieved via deprotonation (Table 1, entry 1). We anticipated that the subsequent addition of excess thiophene and CAN would trigger a cascade of reactions, resulting in the formation of the desired arylated nitroalkane **3a**. However, when the reaction was performed under cryogenic conditions only the unreacted nitroalkane **1b** was recovered (Table 1, entry 1). The following experiment, in which the reaction mixture was warmed to room temperature, produced a 20% yield of the desired product **3a** alongside the unreacted starting material **1b** and the dimer **7** (Table 1, entry 2). When the same reaction was carried out at 0–5 °C, the chemical yield improved to 34% (Table 1, entry 3). To avoid manipulation with hygroscopic solids, potassium *tert*-butoxide was replaced with a solution of sodium methoxide in methanol (Table 1, entry 4). Due to the significant formation of the dimer in earlier experiments, the amount of the readily available arene was increased to 20 mol equiv. As anticipated, the formation of dimer **7** was suppressed, and the NMR yield of desired product **3a** increased to 52% (Table 1, entry 5). Changing the solvent to ethanol (Table 1, entry 6) and radical increase of the amount of base (Table 1, entry 7) proved to be detrimental to the chemical yield. However, further fine-tuning of the base and oxidant amounts, concentrations, reaction temperature, and reaction time helped identify optimal conditions resulting in an acceptable 62% NMR yield (Table 1, entry 8). To our delight, the reaction proceeded with high regioselectivity, and one major regioisomer was observed in the crude reaction mixture, as confirmed by comparison with independently synthesized regioisomer **3a’**.^29,30^ Isolation by chromatography provided the desired product **3a** in 33% yield. Despite the moderate NMR yield and unoptimized isolated yield, we pursued the development of the methodology. We believe that the high regioselectivity, easily accessible reagents, and technical simplicity of the reaction setup altogether represent highly attractive features of the novel synthetic tool. With preferred conditions identified, the reaction scope was then investigated. A range of simple and functionalized nitroalkanes **1** was prepared using either literature or novel procedures.^29^ The selection of the arylation heterocyclic partner **5** was made to showcase the possibility of building complexity within a single operation while simultaneously demonstrating the regioselectivity of the reactions. Any C-arylation of thiophene (**5a**) can produce only two possible regioisomers (e.g., **3a** and **3a’**, Table 1).

**Table 1:**
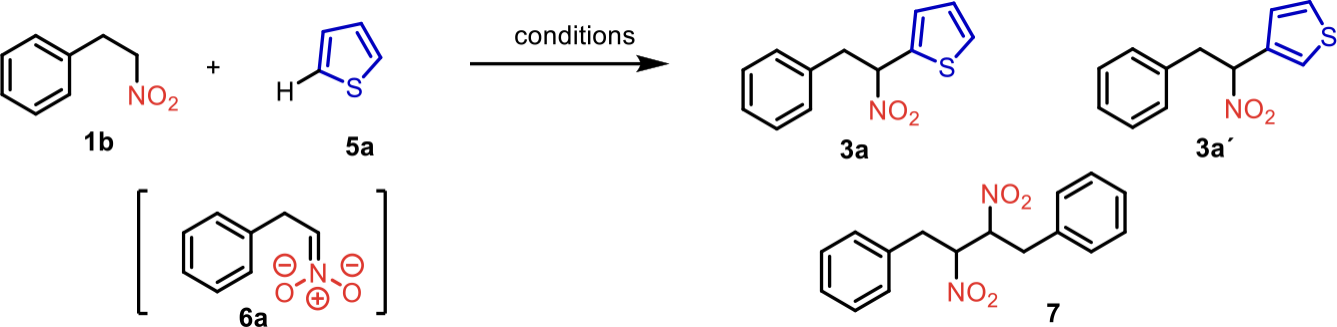
Optimization of the Reaction Conditions

Entry	Reaction conditions^a^	Recovery **1b** ^b^	Yield **3a** ^b^ ^,^ ^c^	Yield **7** ^b^
1	*t*BuOK (1.2 equiv), **5a** (5 equiv), CAN (2.0), MeOH (0.2 M), –78 °C, 1 h	65%	0%	0%
2	*t*BuOK (1.2 equiv), **5a** (5 equiv), CAN (2.0), MeOH (0.2 M), –78 °C to rt, 1 h	4%	20%	40%
3	*t*BuOK (1.2 equiv), **5a** (5 equiv), CAN (2.0), MeOH (0.2 M), 0–5 °C, 1 h	8%	34%	37%
4	NaOMe (1.2 equiv), **5a** (5 equiv), CAN (2.0), MeOH (0.2 M), 0–5 °C, 1 h	7%	32%	47%
5	NaOMe (1.2 equiv), **5a** (20 equiv), CAN (2.0), MeOH (0.2 M), 0–5 °C, 1 h	4%	52%	41%
6	NaOMe (1.2 equiv), **5a** (20 equiv), CAN (2.0), EtOH (0.2 M), 0–5 °C, 1 h	4%	29%	41%
7	NaOMe (3 equiv), **5a** (20 equiv), CAN (2.0), MeOH (0.2 M), 0–5 °C, 1 h	4%	29%	28%
8	NaOMe (1.3 equiv), **5a** (20 equiv), CAN (2.2), MeOH (0.4 M), rt, 15 min	4%	62% (33%)^d^	13%

a All Reactions Were Performed on a 0.4 mmol Scale of Nitroalkane **1b** Using Reagent-Grade Chemicals without an Inert Atmosphere.

b Determined by ^1^H NMR against an internal standard (Cl_2_CHCH_2_Cl).

c Regioisomeric excess r.e. >95% is based on the ^1^H NMR analysis of the crude mixture.^29^

d Isolated yield after preparative HPLC.

On the other hand, monosubstituted thiophenes can react at three different positions, potentially yielding three distinct regioisomers. A functional group present in the thiophene may offer further opportunities to build complexity during the arylation step. To assess the methodology’s regioselectivity and functional group tolerance, we continued our investigation with 2-substituted thiophenes in reactions with a range of nitroalkanes bearing various functional groups (Scheme 2). Employing standard reaction conditions, the model nitroalkane **1b** reacted with 2-methyltiophene (**5b**) in a highly regioselective manner, producing the desired product **3b** (Scheme 2). The independent synthesis of the other two possible regioisomers confirmed the selective formation of 2,5-disubstituted isomer **3b**.^29–31^ Pleased with the highly selective nature of the process, nonfunctionalized linear and branched nitroalkanes were employed next. A regioselective C-arylation occurred smoothly in both cases, producing arylated derivatives **3c** and **3d**. Noteworthy is the observed regioselectivity, as the desired products were isolated as a major regioisomer.^31^ The reaction also tolerated other hydrocarbon moieties such as alkenes, alkynes, and arenes, as demonstrated by the preparation of compounds **3e**–**3k**. Intending to introduce more reactive functionalities, our attention turned to arylation with nitroalkanes containing a hydroxy group, an ether, a silyl ether, an acetal, an ester, an amide and a carbamate. The corresponding desired products **3l**–**3r** were isolated in respectable yields in all cases. The method could even be extended to nitroalkanes derived from protected saccharides and peptides, exemplified by the complex arylated products **3s** and **3t**. Having explored structural modifications of nitroalkanes, we next focused on alterations of the heteroaromatic partner. Various substituents were tolerated at position two of the heterocycle **5**. Thus, 2-hexyl, 2-bromo-, and 2-hydroxymethyl-substituted derivatives **3u**–**3x** were prepared.

**Scheme 2:**
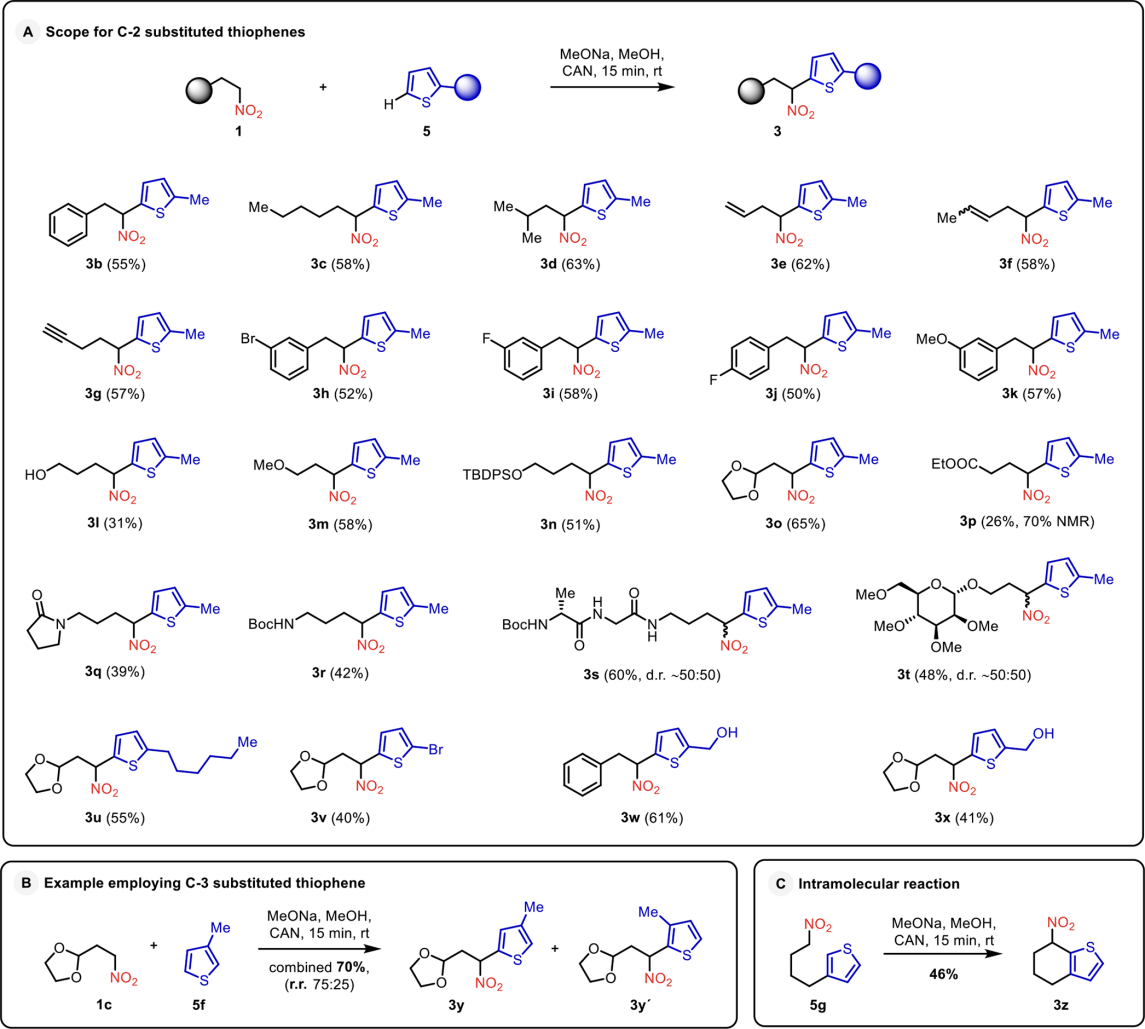
Reaction Scope with Monosubstituted Thiophenes

Although the excellent selectivity observed for 2-substituted derivatives decreased, arylation with 3-substituted thiophene **5f** remained regioselective, as two isomers **3y** and **3y’**, were isolated in a combined 70% yield with r.r. 75:25. The investigation of an intramolecular version of the transformation was also fruitful. Due to an intramolecular arylation, bicyclic derivative **3z** was isolated as a single regioisomer from 3-substituted thiophene **5g** containing a tethered nitroalkyl chain.

Recognizing the synthetic potential of this novel arylation strategy in modifying other electron-rich heterocycles, we decided to investigate whether an analogous transformation of furan (**5h**) was feasible. Pleasingly, the arylation successfully proceeded, yielding nitroalkylated furans **3aa**–**3ai**, albeit with the need for additional optimization of solvent and reaction time (Scheme 3). The reaction rapidly yielded the nonaromatic intermediate **8**, then proceeded smoothly via aromatization in ethanol within 16 h at ambient temperature, providing coupling products **3** in moderate yields. Various structural modifications of nitroalkanes **1** were tolerated. A nonfunctionalized branched nitroalkane was successfully coupled with furan (**5h**) producing the nitroalkylated heterocycle **3aa** in a moderate yield. Substrates bearing more reactive functional groups such as C=C double and C≡C triple bonds, aromatic rings, free hydroxyl group, methyl ether, and ester were also competent in the coupling, yielding nitro compounds **3ab**–**3ag**. Scrutinizing the method’s limits, furan (**5h**) was reacted with nitroalkanes derived from a protected saccharide and peptide. To our delight, the C-2 functionalized furan **3ah** and **3ai** were obtained as epimeric mixtures at the stereogenic center bearing the nitro group (Scheme 3). The described method allows rapid access to variously functionalized heteroarylated nitroalkanes. This class of compounds has not been very populous,^32^ arguably due to the limited number of existing synthetic methods. However, it undeniably has significant synthetic potential due to the well-developed chemistry of both structural fragments—the heteroaromatic ring and the nitro group. To demonstrate the importance and synthetic potential of compounds prepared by this methodology, we selected nitro compound **3c** as the model substrate (Scheme 4). Extensive reduction of the nitro group in **3c** led to the formation of amine **9a** when zinc was employed as the reducing agent. In combination with benzaldehyde and acetic acid, zinc again served as the reductant in the high-yielding formation of nitrone **9b**. The chameleon-like character of the nitro group^33,34^ was further demonstrated by the synthesis of ketone **9c** via the Nef reaction.^35^ The collected NMR data, which matched previously published spectra,^36^ provided further evidence of the proposed regioselectivity in the arylation step. Dearomative hydrodesulfurization of thiophene offers a fascinating transformation of an aromatic compound to an aliphatic derivative.^37^ In conjunction with alkylation using an alcohol under Ni-catalyzed hydrogen- borrowing conditions,^38,39^ it enabled the preparation of secondary amine **9d**.

**Scheme 3:**
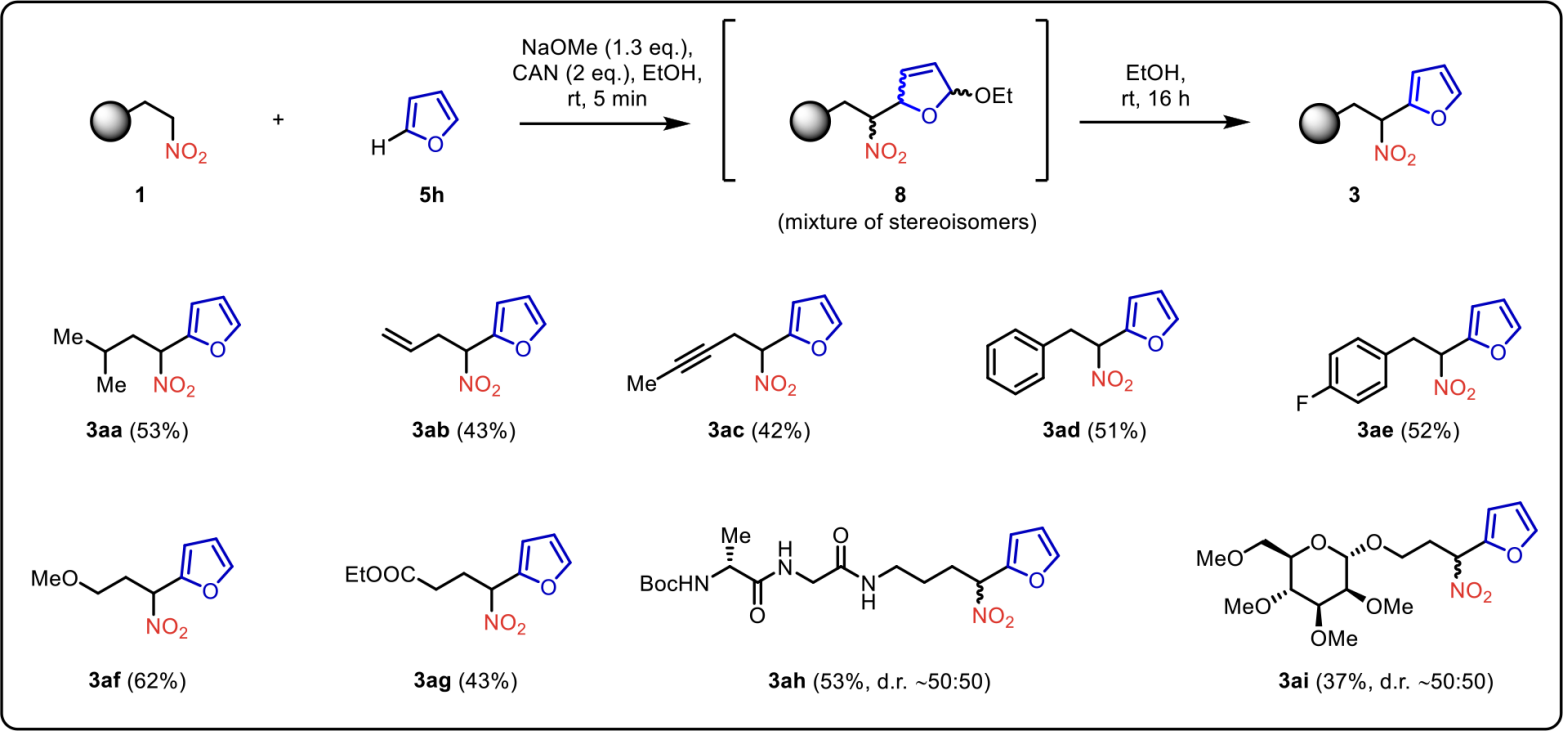
Reaction Scope of C-Arylation of Nitroalkanes with Furan

**Scheme 4:**
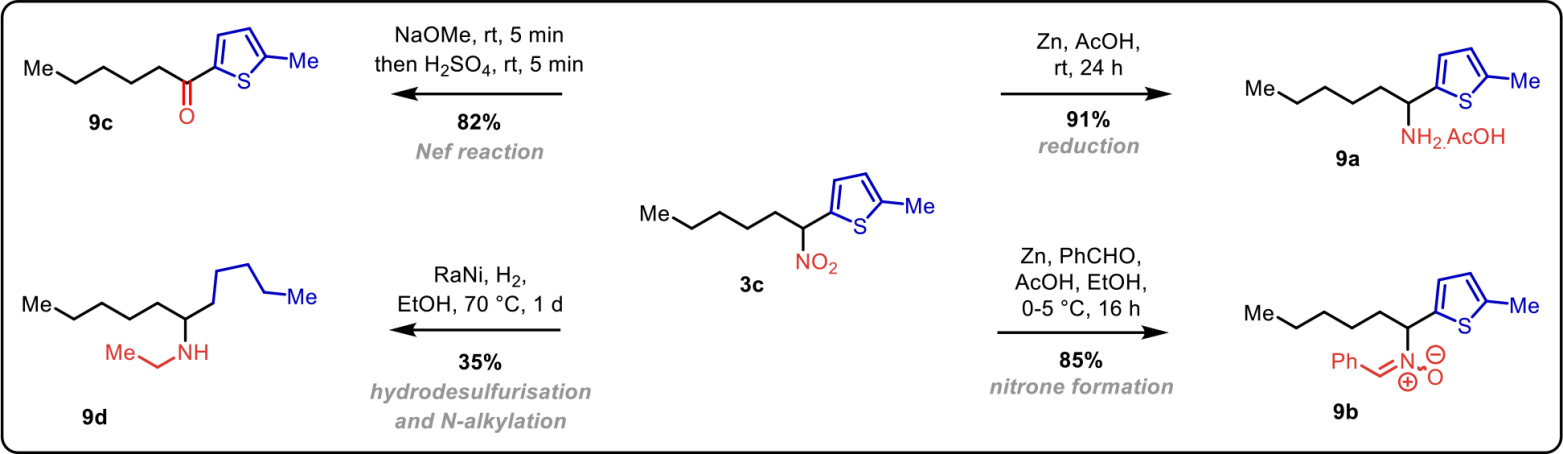
Synthetic Applications of a C-Arylated Nitroalkane

Given the direct evidence for the presence of nonaromatic intermediate **13** in the furan series and dimers **7** in all reactions, we postulate that the reaction proceeds via the mechanism shown in Scheme 5. In the first step, nitroalkane **1** is fully deprotonated by sodium alkoxide, to form the nitronate anion **6**. Upon addition of CAN as an oxidizing agent, a single-electron transfer (SET) occurs, generating radical **10**. Next, the heteroaromatic compound **5** quickly reacts with the radical **10**, generating the more stable radical **11**. This is followed by another single-electron transfer step (SET step), forming the stabilized allylic carbocation **12**. Subsequently, the carbocation **12** undergoes nucleophilic addition of the alkoxide to form the intermediate **13**, which exists as a mixture of racemic diastereomers. Finally, intermediate **13** undergoes aromatization to afford the desired product **3**. Alongside with the desired product **3**, dimers **7** were formed through the recombination of the α-nitroalkyl radical **10**. Further experimental support for the radical pathway was obtained when the arylation of **1b** with thiophene **5a** was performed in the presence of the radical scavenger TEMPO. Arylated product **3** was not observed, instead, an adduct of radical **10** and TEMPO was isolated.^29^ Unfortunately, even the aromatization of dihydrofuran, which is the rate-determining step, cannot be monitored by ^1^H/^19^F NMR kinetics due to significant overlap of signals.

**Scheme 5:**
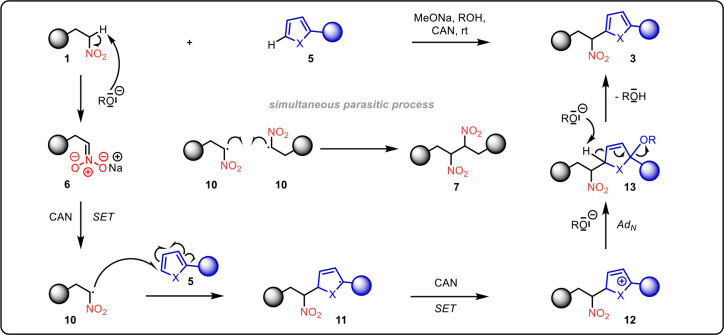
Proposed Reaction Mechanism

Therefore, to support the proposed mechanism and rationalize the observed regioselectivity, we performed density functional theory (DFT) calculations employing the ωB97X-D4 functional^40,41^ containing an empirical dispersion correction in combination with the def2-TZVPP basis set.^42^ The solvent effects were included via the universal implicit solvation model based on solute density (SMD).^43^ In the case of solvent-assisted reactions, relevant explicit solvent molecules were included in the model (see Section 3 in the Supporting Information). To better address the regioselectivity issue, a reaction of **5b** offering three nonequivalent positions for the addition of a representative phenyl-substituted nitroalkane **1j** was pursued as a primary working example. The calculated Gibbs energy profile indicates that all reaction steps are thermodynamically favorable and are either barrierless or their activation barriers can be readily overcome at room temperature (Figure 1). In particular, the activation of nitroalkane into radical **10j** is strongly exergonic and proceeds through a low activation barrier (∼8 kcal/mol) related to the deprotonation step. The formation of radical adduct **11j** is slightly slower, and the activation energy is position dependent (vide infra). The cationic adduct **12j** can be transformed to **3j** either via barrierless deprotonation from the position #2 of the heteroaromatic ring or through intermediate **13j** formed readily by addition of alkoxide (Figure 2a). The conversion of **12j** to **3j** appears to be ruled by stereochemistry, as the deprotonation is only feasible if an alkoxide anion occurs nearby the hydrogen atom in position #2. The addition of alkoxide occurs preferentially on position #5, which is the most electrophilic site on the ring (Figure 2b,c).

**Figure 1:**
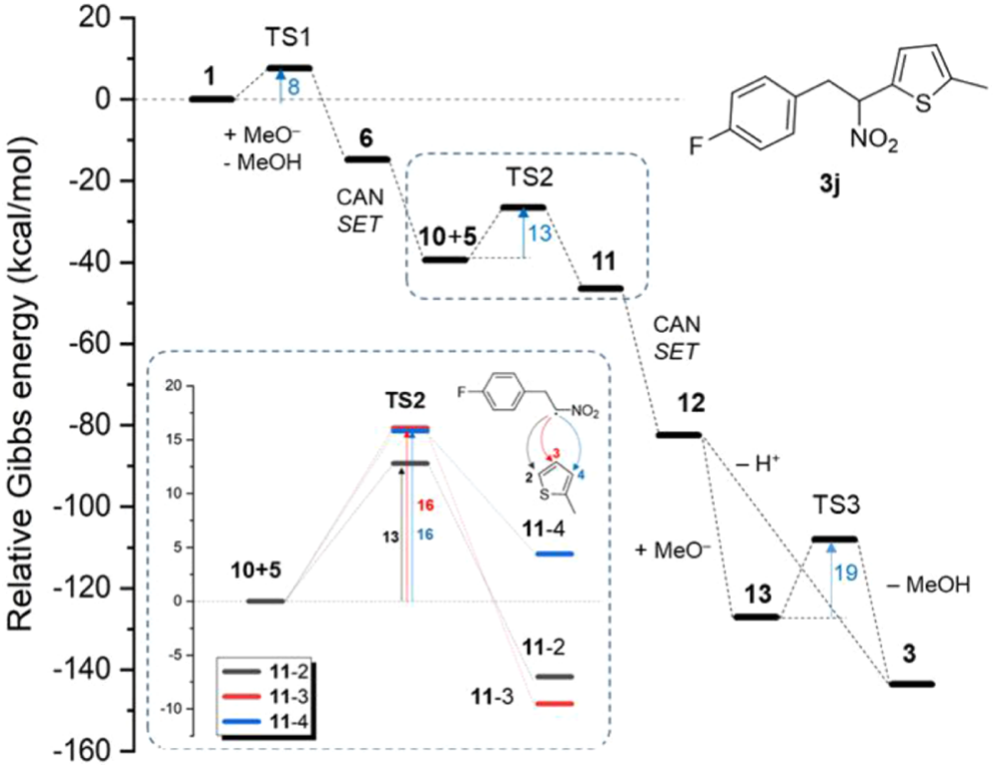
Theoretical Gibbs energy profile (in kcal/mol) of the formation of **3j** from **1k** and **5b** following the mechanism shown in Scheme 5. Inset: Comparison of activation barriers of the formation of regioisomers **11j** via radical addition of **10j** to different positions on **5b**, which is a critical step for the regioselectivity of the whole reaction cascade. All values were obtained at the ωB97X-D4/def2-TZVPP/SMD­(methanol) level of theory.

**Figure 2:**
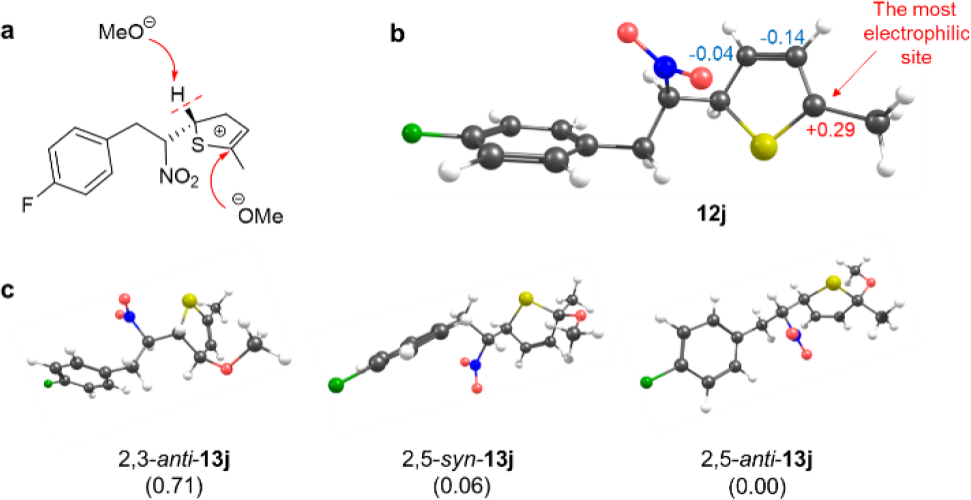
(a) Two alternative attacks of a methoxide anion on cationic intermediate **12j** involving either barrierless deprotonation from the position #2 of the heteroaromatic ring or the formation of adduct **13j**. (b) The optimized structure of **12j** with Mulliken atomic charges on relevant carbons. (c) Isomers of **13j** and their relative Gibbs energies (in kcal/mol).

The activation barrier for the elimination of alcohol from intermediate **13j** is ∼19 kcal/mol, which is apparently too small for capturing this intermediate experimentally. Indeed, the analysis of an analogous reaction pathway for a furan derivative **3ad** (Figure S41) revealed that the activation barrier for the alcohol elimination increased to ∼23 kcal/mol, explaining the higher chance to observe intermediate **13ad**. The observed regioselectivity is not driven by the thermodynamic stability of products. In fact, differences in the stability of regioisomers of **3j** are only minor (<0.3 kcal/mol), and actually the most stable is the one with the nitroalkane moiety attached to carbon #4 (Figure S40). For the furan derivative, the regioisomer **3ad**-2 is also only slightly more stable than **3ad**-3 (Figure S44). On the other hand, the position #2 was found to be kinetically most favorable (by about 3 and 4 kcal/mol for **11j** and **11ad**, respectively) for the attack of radical **10** on the heteroaromatic ring **5** (see inset in Figures 1 and Figures S37 and S41). Although the radical adduct **11j**-3 with nitroalkane in position #3 is thermodynamically more stable due to a cyclization involving the nitro group (Figure S37), it is reasonable to assume that the addition spatially limited to a 5-membered ring proceeds via the pathway with the smallest activation barrier, i.e., toward regioisomer **11j**-2. In addition, the cationic form of the cyclic structure **12j**-3 is thermodynamically unstable and thus, even if formed, would presumably convert to much more favorable structure **12j**-2 (Figure S38).

## Conclusion

In conclusion, we have developed a novel regioselective arylation of nitroalkanes utilizing readily available raw heterocyclic materials. The process, which applies to thiophene, thiophene derivatives, and furan, is straightforward to execute and provides an attractive tool for the rapid construction of functionalized heterocycles with predictable regioselectivity. The synthetic utility of the resulting derivatives was exemplified through the selective manipulation of both the nitro group and the thiophene ring. We have gained a comprehensive understanding of the reaction mechanism through a combination of computational studies and experimental mechanistic investigations.

## Supplementary Material





## Data Availability

The data underlying this study are available in the published article and its Supporting Information.

## Notes

Experimental procedures, characterization data, ^1^H and ^13^C NMR spectra and computational details (PDF)
Cartesian coordinates and corresponding Gibbs energy values (XYZ)
