# Supplementary material for: Regioselective C‑Arylation of Functionalized Nitroalkanes with Furan, Thiophene, and Substituted Thiophenes
Source: J Org Chem. 2025 Nov 21;91(4):1602–8. doi: 10.1021/acs.joc.5c02410 (PMC12865766; doi:10.1021/acs.joc.5c02410)
Supplement: Supplementary file 1 [file jo5c02410_si_001.pdf]

Supporting Information

Regioselective C-Arylation of Functionalized Nitroalkanes with Furan, Thiophene, and Substituted Thiophenes

Katarína R. Detková,[a] Kristína Jakubcová,[a] Tomáš Malatinský,[a] Juraj Filo,[b] Marek Cigáň,[b] Šimon Budzák,[c] Miroslav Medveď,*[c,d] and Pavol Jakubec*[a]

[a] K. R. Detková, K. Jakubcová, Dr. T. Malatinský, Dr. P. Jakubec*
Department of Organic Chemistry, Slovak University of Technology, Radlinského 9, Bratislava 1, Slovakia
E-mail: pavol.jakubec@stuba.sk

[b] Dr. J. Filo, Dr. M. Cigáň, Department of Organic Chemistry, Faculty of Natural Sciences, Comenius University, Ilkovičova 6, Bratislava SK-842 15, Slovakia

[c] Dr. Š. Budzák, Dr. M. Medveď*
Department of Chemistry, Faculty of Natural Sciences, Matej Bel University, Tajovského 40, SK-97400 Banská Bystrica, Slovakia, E-mail: Miroslav.Medved@umb.sk

[d] Dr. M. Medveď*
Regional Centre of Advanced Technologies and Materials, Czech Advanced Technology and Research Institute (CATRIN), Palacký University Olomouc, Šlechtitelů 241/27, Olomouc, 783 71, Czech Republic

Contents

1. General experimental and methods S7

1.1 Reagents and solvents S7

1.2 Chromatography S7

1.3 Spectroscopy and instruments S7

1.4 Starting materials S8

2. Practical experimental S9

2.1 Synthesis and characterisation of nitroalkanes S9

2.1.1 5-Nitropent-2-ene (1g) S9

2.1.2 1-Bromo-3-(2-nitroethyl)benzene (1l) S9

2.1.3 4-Nitrobutanol (1n) S10

2.1.4 1-Methoxy-3-nitropropane (1o) S10

2.1.5 *tert*-Butyl(4-nitrobutoxy)diphenylsilane (1p) S11

2.1.6 Ethyl 4-nitrobutanoate (1q) S11

2.1.7 Synthesis of nitroalkane 1t S12

2.1.7.1 (*R*)-*tert*-Butyl (1-((2-((4-hydroxybutyl)amino)-2-oxoethyl)amino)-1-oxopropan-2-yl)carbamate (24) S12

2.1.7.2 (*R*)-*tert*-Butyl (1-((2-((4-bromobutyl)amino)-2-oxoethyl)amino)-1-oxopropan-2-yl)carbamate (25) S12

2.1.8 Synthesis of nitroalkane (1u) S13

2.1.8.1 (2*S*,3*S*,4*S*,5*R*,6*R*)-2-(3-Bromopropoxy)-3,4,5-trimethoxy-6-(methoxymethyl)tetrahydro-2H-pyran (28) S13

2.1.8.2 (2*R*,3*R*,4*S*,5*S*,6*S*)-3,4,5-Trimethoxy-2-(methoxymethyl)-6-(3-nitropropoxy)tetrahydro-2H-pyran (1u) S14

2.1.9 3-(4-Nitrobutyl)thiophene (5g) S14

2.2 Synthesis and characterisation of nitroalkanes C-arylated with thiophenes S15

2.2.1 General procedure A for C-arylation with thiophenes S15

2.2.2 2-(2-Phenyl-1-nitroethyl)thiophene (3a) S15

2.2.3 2-Methyl-5-(2-phenyl-1-nitroethyl)thiophene (3b) S16

2.2.4 2-Methyl-5-(1-nitrohexyl)thiophene (3c) S16

2.2.4.1 Synthesis of compound 3c on 5 mmol scale S16

2.2.5 2-Methyl-5-(3-methyl-1-nitrobutyl)thiophene (3d) S17

2.2.6 2-Methyl-5-(1-nitrobut-3-en-1-yl)thiophene (3e) S17

2.2.7 2-Methyl-5-(1-nitropent-3-en-1-yl)thiophene (3f) S18

2.2.8 2-Methyl-5-(1-nitropent-4-yn-1-yl)thiophene (3g) S18

2.2.9 2-(3-Bromophenethyl)-5-methylthiophene (3h) S19

2.2.10 2-(2-(3-Fluorophenyl)-1-nitroethyl)-5-methylthiophene (3i) S19

2.2.11 2-(2-(4-Fluorophenyl)-1-nitroethyl)-5-methylthiophene (3j) S20

2.2.12 2-(2-(3-Methoxyphenyl)-1-nitroethyl)-5-methylthiophene (3k) S20

2.2.13 4-(5-Methylthiophen-2-yl)-4-nitrobutan-1-ol (3l) S21

2.2.15 *tert*-Butyl(4-(5-methylthiophen-2-yl)-4-nitrobutoxy)diphenylsilane (3n) S22

2.2.16 2-(2-(5-Methylthiophen-2-yl)-2-nitroethyl)-1,3-dioxolane (3o) S22

2.2.17 Ethyl 4-(5-methylthiophen-2-yl)-4-nitrobutanoate (3p) S23

2.2.18 1-(4-(5-Methylthiophen-2-yl)-4-nitrobutyl)pyrrolidin-2-one (3q) S23

2.2.19 *tert*-Butyl-(4-(5-(hydroxymethyl)thiophen-2-yl)-4-nitrobutyl)carbamate (3r) S24

2.2.21 *tert*-Butyl ((*2R*)-1-((2-((4-(5-methylthiophen-2-yl)-4-nitrobutyl)amino)-2-oxoethyl)amino)-1-oxopropan-2-yl)carbamate (3s) S24

2.2.20 (*2S,3S,4S,5R,6R*)-3,4,5-Trimethoxy-2-(methoxymethyl)-6-(3-(5-methylthiophen-2-yl)-3-nitropropoxy)tetrahydro-2H-pyran (3t) S25

2.2.22 2-(2-(5-Hexylthiophen-2-yl)-2-nitroethyl)-1,3-dioxolane (3u) S25

2.2.23 2-(2-(5-Bromothiophen-2-yl)ethyl)-1,3-dioxolane (3v) S26

2.2.24 (5-(1-Nitro-2-phenylethyl)thiophen-2-yl)methanol (3w) S26

2.2.25 (5-(2-(1,3-Dioxolan-2-yl)-1-nitroethyl)thiophen-2-yl)methanol (3x) S27

2.2.26 2-(2-(4-Methylthiophen-2-yl)-2-nitroethyl)-1,3-dioxolane (3y) and 2-(2-(3-methylthiophen-2-yl)-2-nitroethyl)-1,3-dioxolane (3y′) S27

2.2.26 7-Nitro-4,5,6,7-tetrahydrobenzo[b]thiophene (3z) S28

2.3 Synthesis and characterisation of nitroalkanes C-arylated with furan S29

2.3.1 General Procedure B for C-arylation of nitroalkanes with furan S29

2.3.2 2-(3-Methyl-1-nitrobutyl)furan (3aa) S29

2.3.3 2-(1-Nitrobut-3-en-1-yl)furan (3ab) S30

2.3.4 2-(1-Nitropent-3-yn-1-yl)furan (3ac) S30

2.3.5 2-(1-Nitro-2-phenylethyl)furan (3ad) S31

2.3.6 2-(2-(4-Fluorophenyl)-1-nitroethyl)furan (3ae) S31

2.3.7 2-(3-Methoxy-1-nitropropyl)furan (3af) S32

2.3.8 Ethyl 4-(furan-2-yl)-4-nitrobutanoate (3ag) S32

2.3.9 *tert*-Butyl ((2R)-1-((2-((4-(furan-2-yl)-4-nitrobutyl)amino)-2-oxoethyl)amino)-1-oxopropan-2-yl)carbamate (3ah) S33

2.3.10 (2*S*,3*S*,4*S*,5*R*,6*R*)-2-(3-(furan-2-yl)-3-nitropropoxy)-3,4,5-trimethoxy-6-(methoxymethyl)tetrahydro-2H-pyran (3ai) S33

2.4 Regioselectivity determination S34

2.4.1 Independent synthesis and characterisation of 3-(1-nitro-2-phenylethyl)thiophene 3a′ S34

2.4.1.1 3-(1-Nitro-2-phenylethyl)thiophene (3a′) S34

2.4.2 Independent synthesis and characterisation of 2-methyl-3-(1-nitro-2-phenylethyl)thiophene (3b′) S34
2.4.2.1 2-Methyl-3-(1-nitro-2-phenylvinyl)thiophene (21e) S35
2.4.2.2 2-Methyl-3-(1-nitro-2-phenylethyl)thiophene (3b′) S35
2.4.3 Independent synthesis and characterisation of 2-methyl-4-(1-nitro-2-phenylethyl)thiophene (3b′′) S35
2.4.3.1 2-Methyl-4-(1-nitro-2-phenylvinyl)thiophene (21f) S36
2.4.3.2 2-Methyl-4-(1-nitro-2-phenylethyl)thiophene (3b′′) S36
2.4.4 Comparison of ^{1}H NMR spectra of 3a′ and crude 3a S37
2.4.5 Comparison of ^{1}H NMR spectra of 3b′, 3b′′ and crude 3b S38
2.5 Derivatisation of C-arylated products S39
2.5.1 1-(5-Methylthiophen-2-yl)hexan-1-amine acetate (9a) S39
2.5.3 (*E*)-N-benzylidene-1-(5-methylthiophen-2-yl)hexan-1-amine oxide (9b) S39
2.5.2 1-(5-Methylthiophen-2-yl)hexan-1-one (9c) S40
2.5.4 *N*-Ethylundecan-6-amine (9d) S40
2.6 Experiments to support the mechanistic proposal S41
2.6.1 Independent synthesis of the dimer 7 .... S41
2.6.1.1 (2,3-Dinitrobutane-1,4-diyl)dibenzene (7) S41
2.6.2 Synthesis and characterisation of the non-aromatic intermediate (8a) S41
2.6.2.1 2-Ethoxy-5-(1-nitro-2-phenylethyl)-2,5-dihydrofuran (8a) S41
2.6.3 Experiment in the presence of radical scavenger TEMPO S42
2.6.3.1 Synthesis and characterisation of 2,2,6,6-tetramethyl-1-(1-nitro-2-phenylethoxy)piperidine (31) S42
3. Theoretical mechanistic investigation S43
4. NMR spectra S48
4.1 NMR spectra of 1g S48
4.2 NMR spectra of 1l S49
4.3 NMR spectra of 1n S50
4.4 NMR spectra of 1o S51
4.5 NMR spectra of 1p S52
4.6 NMR spectra of 1q S53
4.7 NMR spectra of 24 .... S54
4.8 NMR spectra of 25 .... S55
4.9 NMR spectra of 1t S56
4.10 NMR spectra of 28 .... S57
4.11 NMR spectra of 1u S58
4.12 NMR spectra of 3a S59

4.13 NMR spectra of 3b S60
4.14 NMR spectra of 3c S61
4.15 NMR spectra of 3d S62
4.16 NMR spectra of 3e S63
4.17 NMR spectra of 3f S64
4.18 NMR spectra of 3g S65
4.19 NMR spectra of 3h S66
4.20 NMR spectra of 3i S67
4.21 NMR spectra of 3j S69
4.22 NMR spectra of 3k S71
4.23 NMR spectra of 3l S72
4.24 NMR spectra of 3m S73
4.25 NMR spectra of 3n S74
4.26 NMR spectra of 3o S75
4.27 NMRspectra of 3p S76
4.28 NMR spectra of 3q S77
4.29 NMR spectra of 3r S78
4.30 NMR spectra of 3s S79
4.31 NMR spectra of 3t S80
4.32 NMR spectra of 3u S81
4.33 NMR spectra of 3v S82
4.34 NMR spectra of 3w S83
4.35 NMR spectra of 3x S84
4.36 NMR spectra of 3y and 3y´ S85
4.37 NMR spectra of 3z S86
4.38 NMR spectra of 3aa S87
4.39 NMR spectra of 3ab S88
4.40 NMR spectra of 3ac S89
4.41 NMR spectra of 3ad S90
4.42. NMR spectra of 3ae S91
4.43 NMR spectra of 3ef S93
4.44 NMR spectra of 3eg S94
4.45 NMR spectra of 3ah S95
4.46 NMR spectra of 3i S96
4.47 NMR spectra of 9a S97
4.48 NMR spectra of 9b S98

4.49 NMR spectra of 9c S99
4.50 NMR spectra of 9d S100
4.51 NMR spectra of 3a´ S101
4.52 NMR spectra of 21e S102
4.53 NMR spectra of 3b´ S103
4.54 NMR spectra of 21f S104
4.55 NMR spectra of 3b´´ S105
4.56 NMR spectra of 7 .... S106
4.57 NMR spectra of 8a S107
4.58 NMR spectra of 31 .... S108
5. References S109

1. General experimental and methods

All reactions were performed without special precautions to avoid the presence of moisture unless otherwise stated.

1.1 Reagents and solvents

Commercially available reagents and solvents were used without further purification. They were obtained from commercial suppliers (Sigma Aldrich, Fluorochem, Abcr, Acros Organics). Solvents were removed under reduced pressure using Büchi Rotavapor and Heidolph apparatus.

1.2 Chromatography

Column chromatography was carried out using silica gel: Merck Kieselgel 60 (15 - 40 µm). All reactions were monitored by thin-layer chromatography (TLC - Merck Kieselgel 60 F254 (230 - 400 mesh) fluorescent treated silica). For practical use, it was visualized under UV light (254 nm) or by staining with a solution of aqueous basic potassium permanganate.

Reverse-phase HPLC used to purify the reaction products was operating with the following parts: Hardware: E-COM machine set-up using ECD2000 detector series. Software: Clarity. Columns: Nucleodur phenyl-hexyl, 5 µm 250 × 100 mm from Macherey-Nagel. Mobile phases: Gradient using 0.1% aqueous HCOOH and acetonitrile.

1.3 Spectroscopy and instruments

^{1}H and ^{13}C NMR spectra were recorded on spectrometers (Varian/NOVA-300 and Varian VNMR S-600), operating at frequencies of 300 MHz and 600 MHz (for ^{1}H nuclei) and 75 MHz and 151 MHz (for ^{13}C nuclei). The resonance frequency of tetramethylsilane (TMS) served as a reference frequency for the calculation of the chemical shift scales ($\delta_H = \delta_C = 0.000$ ppm). For the ^{1}H spectra, the residual non-perdeuterated solvent signal ($CHCl_3$: $\delta_H = 7.260$ ppm) was used as a secondary reference whilst correctly shifting the chemical shift scale. For the ^{13}C spectra, the deuterated solvent signal ($CDCl_3$: $\delta_C = 77.16$ ppm) was used analogously. Chemical shifts (δ) are reported in parts per million (ppm), and coupling constants (J) are given in Hertz (Hz). The ^{1}H NMR spectra are reported as follows: δ/ppm [number of protons, multiplicity (s = singlet, d = doublet, t = triplet, q = quartet, quin = quintet, m = multiplet, "t" = apparent triplet, b = broad), coupling constants J/Hz (where appropriate)]. Data were analyzed using the Mestrenova program. All NMR spectra and measurement parameters are available upon request from the corresponding author. High-resolution mass spectra (HRMS) were recorded on Orbitrap Elite (Thermo Fisher Scientific).

1.4 Starting materials

All utilised nitroalkanes were either commercially available or prepared either according to referenced literature procedures or newly developed procedures described in the text below. All starting materials for their preparation are commercially available from major worldwide suppliers.

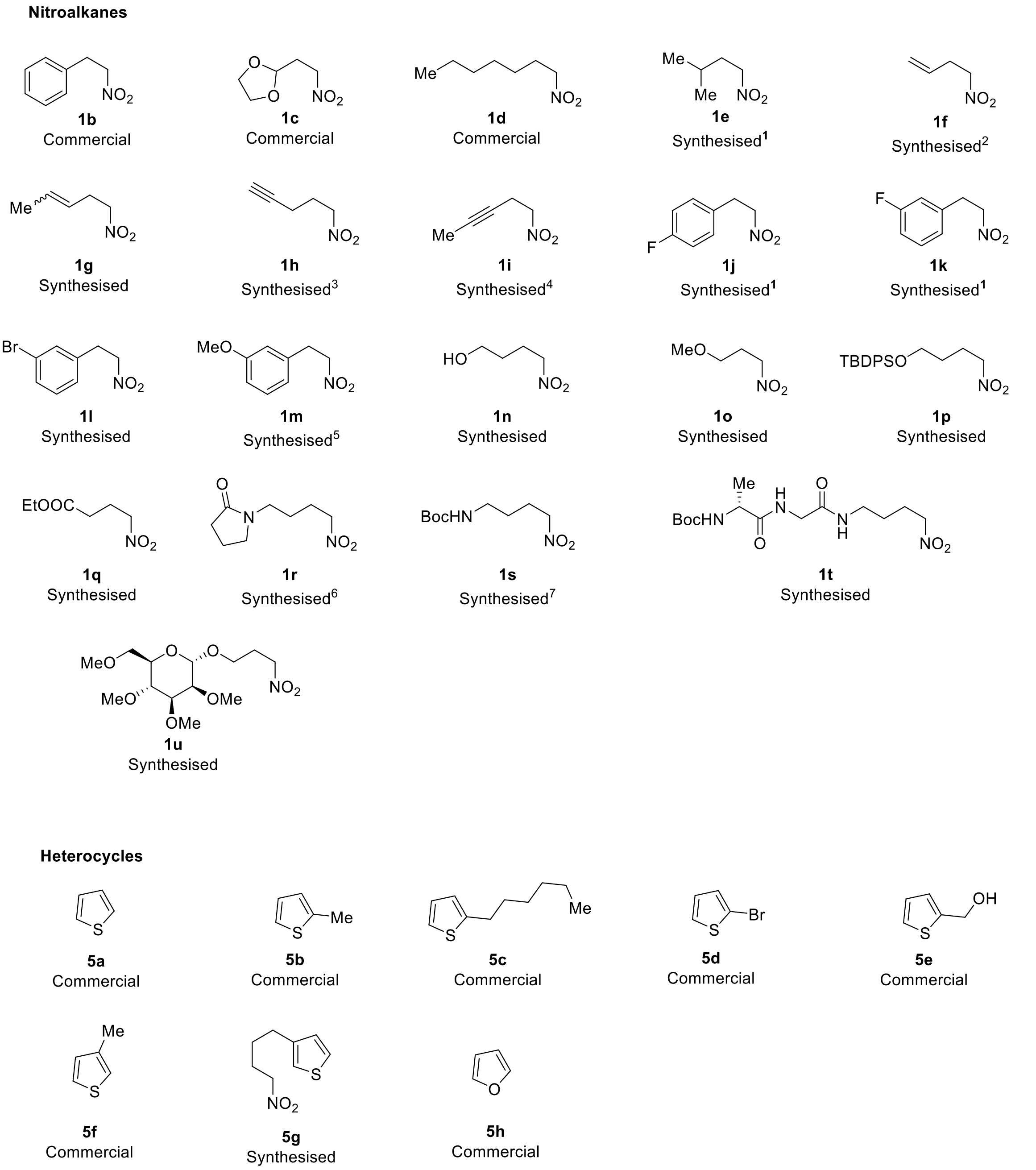

Figure S1

2. Practical experimental

2.1 Synthesis and characterisation of nitroalkanes

2.1.1 5-Nitropent-2-ene (1g)

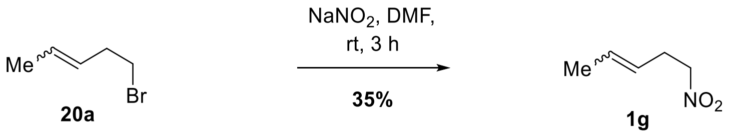

Scheme S1

To a mixture of 5-bromopent-2-ene (**20a**) (40.0 mol, 5.961 g, mixture of diastereomers) and DMF (80 mL) was added portion wise sodium nitrite (48.0 mmol, 4.08 g) at rt and the mixture was stirred at rt. After 3 h the mixture was diluted with H_2O (400 mL, pre-cooled to 4 °C) and the resulting mixture was extracted with Et_2O (3 × 50 mL). The combined organics were washed with brine (50 mL), dried (Na_2SO_4) and concentrated *in vacuo* (p = 600 mbar, T of the bath 40 °C) yielding a pale-yellow liquid (7.2 g). This residue was purified by column chromatography (gradient eluent hexane:EtOAc 98:2 to 96:4) yielding nitroalkane **1g** (1.62 g, 35%, a mixture of diastereomers) as a pale-yellow liquid.

^{1}H NMR (300 MHz, $CDCl_3$) δ 5.67 – 5.54 (m, 1H), 5.42 – 5.31 (m, 1H), 4.38 (t, *J* = 7.0 Hz, 2H), 2.70 – 2.59 (m, 2H), 1.70 – 1.62 (m, 3H). **^{13}C{^{1}H} NMR** (75 MHz, $CDCl_3$) δ 130.1, 124.3, 75.5, 30.6, 18.0. **HRMS** (*m/z*): $[M+H]^+$ calcd for $C_5H_{10}NO_2^+$, 116.07060; found, 116.07072.

2.1.2 1-Bromo-3-(2-nitroethyl)benzene (1l)

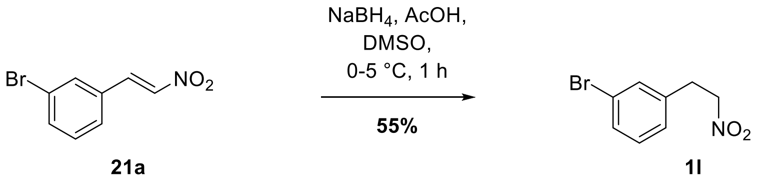

Scheme S2

A mixture of nitrostyrene **21a** (8.000 mmol, 1.824 g) and DMSO (8 mL) was cooled to 0-5 °C (ice-bath) under argon. AcOH (2.2 eq, 18.0 mmol, 1.06 g, 1.00 mL) was added. Subsequently, sodium borohydride (1.2 eq., 9.60 mmol, 0.371 g) was added portion wise over one hour and the mixture was left stirring at 0-5 °C (ice-bath). After 2.5 hours, water (40 mL) was added and the mixture was extracted with Et_2O (2 × 40 mL). The combined organic layers were washed with saturated aqueous solution of $NaHCO_3$ (40 mL), dried (Na_2SO_4) and concentrated *in vacuo* yielding yellow oil. The crude product was purified by flash column chromatography (gradient hexane to hexane:EtOAc 99:1 to 95:5), yielding nitroalkane **1l** (1.01 g, 55%) as a pale-yellow oil.

^{1}H NMR (300 MHz, $CDCl_3$) δ 7.47 – 7.32 (m, 1H), 7.27 – 7.06 (m, 1H), 4.59 (t, *J* = 7.3 Hz, 1H), 3.27 (t, *J* = 7.2 Hz, 1H). **^{13}C{^{1}H} NMR** (75 MHz, $CDCl_3$) δ 138.0, 131.7, 130.7, 130.6, 127.3, 122.9, 75.8, 32.9. All data are in good agreement with previously published data.[8]

2.1.3 4-Nitrobutanol (1n)

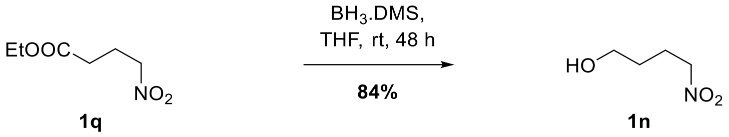

Scheme S3

A mixture of nitro ester **1q** (25.00 mmol, 4.029 g) and THF (25 mL) was cooled to 0-5 °C (ice-bath) under argon and BH_3.DMS (1.2 eq., 30 mmol, 2.3 g, 2.8 mL) was added drop wise over 30 min. The mixture was warmed to rt and stirred at rt under argon. After 48 h the mixture was cooled to 0-5 °C (ice-bath) and water (2.8 mL) was added carefully over 30 min (visious evolution of gas observed which eventually ceased) and the mixture was concentrated *in vacuo* yielding colorless heterogeneous mixure. Saturated aqueous $NaHCO_3$ solution (10 mL) was added to the residue and the mixture was extracted with EtOAc (3 × 10 mL), the combined organics were washed with brine (10 mL), dried (Na_2SO_4) and concentrated *in vacuo* yielding colorless liquid (2.8 g). This residue was purified by column chromatography (gradient eluent hexane to hexane:Et_2O 95:5) affording 4-nitrobutanol (**1n**) (2.5 g, 84%) as a colorless liquid.

^{1}H NMR (300 MHz, $CDCl_3$) δ 4.43 (t, *J* = 7.0 Hz, 2H), 3.67 (t, *J* = 6.2 Hz, 2H), 2.20 – 2.01 (m, 2H), 1.92 (bs, 1H), 1.73 – 1.55 (m, 2H). **^{13}C{^{1}H} NMR** (75 MHz, $CDCl_3$) δ 75.5, 61.6, 29.0, 24.1. All data are in good agreement with previously published data.[9]

2.1.4 1-Methoxy-3-nitropropane (1o)

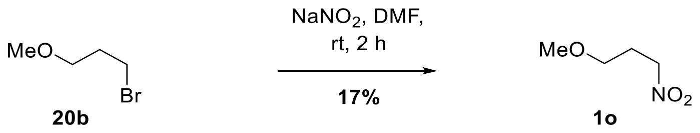

Scheme S4

To a mixture of sodium nitrite (1.2 eq., 60.0 mmol, 4.14 g) and DMF (50 mL) was added portion wise over 10 min 1-bromo-3-methoxypropane (**20b**) (50.0 mmol, 7.651 g) at rt and the mixture was stirred at rt. After 2 h the mixture was diluted with H_2O (200 mL, pre-cooled to 4 °C) and the resulting mixture was extracted with Et_2O (3 × 50 mL). The combined organics were washed with brine (50 mL), dried (Na_2SO_4) and concentrated *in vacuo* (p = 600 mbar, T of the bath 40 °C) yielding a pale-yellow liquid (5.0 g). This residue was purified by column chromatography (gradient eluent hexane to hexane:Et_2O 80:20) yielding nitroalkane **1o** (1.0 g, 17%) as a colorless liquid.

^{1}H NMR (300 MHz, $CDCl_3$) δ 4.48 (t, *J* = 6.9 Hz, 2H), 3.46 (t, *J* = 6.9 Hz, 2H), 3.32 (s, 3H), 2.25 (m, 2H). **^{13}C{^{1}H} NMR** (75 MHz, $CDCl_3$) δ 72.7, 68.6, 58.8, 27.6. **HRMS** (*m/z*): $[M+H]^+$ calcd for $C_4H_{10}NO_3^+$, 120.06552; found, 120.06525.

2.1.5 *tert*-Butyl(4-nitrobutoxy)diphenylsilane (1p)

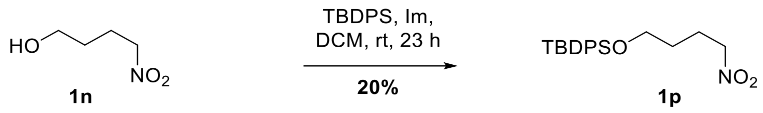

Scheme S5

To a mixture of 4-nitrobutanol (**1n**) (16.79 mmol, 2.000 g) and DCM (170 mL) was added imidazole (1.2 eq., 20.15 mmol, 1.372 g). After 5 min TBDPSCl (16.79 mmol, 4.615 g) was added and the mixture was stirred at rt. After 23 h water (70 mL) was added, the phases were separated and the aqueous phase was extracted with DCM (2 × 60 mL). The combined organic phases were washed with brine (70 mL), dried (Na_2SO_4) and concentrated *in vacuo* yielding a colorless liquid (6.82 g). This residue was purified by column chromatography (gradient eluent hexane to hexane:EtOAc 95:5 to 90:10) yielding colorless oil (2.4 g) containing nitroalkane **1p** and TBDPSCl-related impurities. This mixture was purified by reverse-phase preparative HPLC yielding nitroalkane **1p** (1.2 g, 20%) as a colorless liquid.

^{1}H NMR (400 MHz, $CDCl_3$) δ 7.68 – 7.61 (m, 4H), 7.48 – 7.39 (m, 6H), 4.40 (t, J = 7.1 Hz, 2H), 3.70 (t, J = 5.9 Hz, 2H), 2.14 (quin., J = 7.2 Hz, 2H), 1.69 – 1.57 (m, 2H), 1.06 (s, 9H). **^{13}C{^{1}H} NMR** (151 MHz, $CDCl_3$) δ 135.7, 133.7, 129.9, 127.9, 75.6, 62.8, 29.1, 27.0, 24.4, 19.3. **HRMS** (*m/z*): $[M+H]^+$ calcd for $C_{20}H_{28}NO_3Si^+$, 358.18330; found, 358.18292.

2.1.6 Ethyl 4-nitrobutanoate (1q)

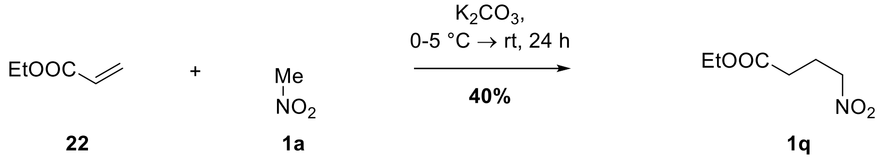

Scheme S6

Nitromethane (**1a**) (20 eq., 2.00 mol, 122 g, 107 mL) was added to ethyl acrylate (100 mmol, 10.0 g, 10.9 mL) and the mixture was cooled to 0-5 ºC (ice-bath). K_2CO_3 (100 mmol, 13.8 g) was added and the mixture, while being stirred, was warmed to rt over 24 h. Then, water (100 mL) was added and the resulting mixture was extracted with EtOAc (2 × 50 mL). The combined organics were washed with brine (100 mL), dried (Na_2SO_4) and concentrated *in vacuo* yielding an orange liquid. The residue was purified by distillation yielding nitroester **1q** (6.45 g, 40%) as a colorless liquid.

Bp = 108-118 °C/3-4 mbar, lit.[10] bp = 96 °C/ 2 torr. **^{1}H NMR** (300 MHz, $CDCl_3$) δ 4.45 (t, J = 6.7 Hz, 2H), 4.12 (q, J = 7.1 Hz, 2H), 2.50 – 2.38 (m, 2H), 2.38 – 2.18 (m, 2H), 1.23 (t, J = 7.1 Hz, 3H). **^{13}C{^{1}H} NMR** (75 MHz, $CDCl_3$) δ 171.9, 74.4, 60.9, 30.5, 22.4, 14.2. All data are in good agreement with previously published data.[11]

2.1.7 Synthesis of nitroalkane 1t

2.1.7.1 (*R*)-*tert*-Butyl (1-((2-((4-hydroxybutyl)amino)-2-oxoethyl)amino)-1-oxopropan-2-yl)carbamate (24)

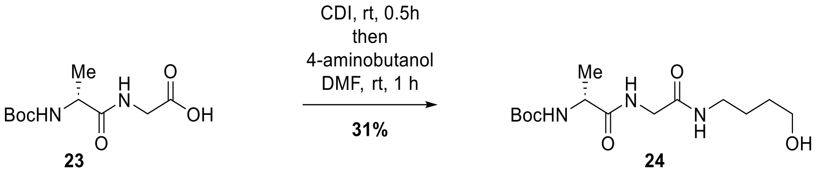

Scheme S7

To a mixture of acid **23** (10.00 mmol, 2.463 g) and DCM (50 mL) was added CDI (10.00 mmol, 1.622 g) and the mixture was stirred at rt with a bubbler attached to the reaction vessel. After 0.5 h to the reaction mixture was added DMF (5 mL) and 4-aminobutanol (1.5 eq., 15.00 mol, 1.337 g) and the resulting mixture was stirred at rt under argon. After 1 h the mixture was concentrated *in vacuo* yielding colorless oil (7.2 g). The residue was purified by column chromatography (gradient eluent hexane:EtOAc 50:50 to EtOAc to EtOAc:MeOH:AcOH 90:10:5) yielding alcohol **24** (0.975 g, 31%) as a colorless oil.

^{1}H NMR (300 MHz, $CDCl_3$) δ 7.28 (bs, 1H), 7.22 (bs, 1H), 5.37 (bs, 1H), 4.12 (quin., *J* = 7.0 Hz, 1H), 3.97 (dd, *J* = 16.7, 6.2 Hz, 1H), 3.86 (dd, *J* = 16.7, 5.5 Hz, 1H), 3.63 (t, *J* = 5.8 Hz, 2H), 3.31 (m, 1H), 3.21 (m, 1H), 1.65 – 1.53 (m, 4H), 1.43 (s, 9H), 1.36 (d, *J* = 7.1 Hz, 3H). **^{13}C{^{1}H} NMR** (151 MHz, $CDCl_3$) δ 173.7, 169.3, 156.2, 80.7, 62.3, 50.9, 43.3, 39.4, 29.9, 28.4, 26.1, 18.1. **HRMS** (*m/z*): $[M+H]^+$ calcd for $C_{14}H_{28}N_3O_5^+$, 318.20235; found, 318.20276.

2.1.7.2 (*R*)-*tert*-Butyl (1-((2-((4-bromobutyl)amino)-2-oxoethyl)amino)-1-oxopropan-2-yl)carbamate (25)

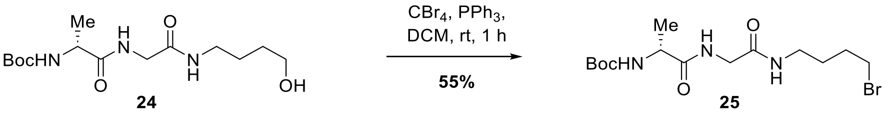

Scheme S8

To a mixture of alcohol **24** (2.99 mmol, 0.950 g) and DCM (30 mL) was added tetrabromomethane (2 eq., 5.986 mmol, 1.985 g) and then portion wise Ph_3P (2 eq., 5.986 mmol, 1.570 g). The mixture was stirred at rt. After 1 h the reaction mixture was concentrated *in vacuo* yielding a pale-yellow heterogeneous mixture (5.2 g). This residue was purified by column chromatography (gradient eluent hexane:EtOAc 50:50 to EtOAc) yielding bromide **25** (0.62 g, 55%) as a colorless oil.

^{1}H NMR (600 MHz, $CDCl_3$) δ 6.96 (bs, 1H), 6.91 (bs, 1H), 5.16 (d, *J* = 5.3 Hz, 1H), 4.08 (qd, *J* = 7.1, 5.7 Hz, 1H), 3.98 – 3.87 (m, 2H), 3.41 (t, *J* = 6.6 Hz, 2H), 3.31 (m, 1H), 3.21 (m, 1H), 1.91 – 1.83 (m, 2H), 1.67 (m, 2H), 1.43 (s, 9H), 1.37 (d, *J* = 7.1 Hz, 3H). **^{13}C{^{1}H} NMR** (151 MHz, $CDCl_3$) δ 173.4, 169.1, 156.1, 80.8, 51.2, 43.3, 38.7, 33.4, 30.0, 28.44, 28.1, 18.0. **HRMS** (m/z): $[M+H]^+$ calcd for $C_{14}H_{28}BrN_3O_4^+$, 380.11795 and 382.11590; found, 380.11777 and 382.11584.

2.1.7.3 (*R*)-*tert*-Butyl (1-((2-((4-nitrobutyl)amino)-2-oxoethyl)amino)-1-oxopropan-2-yl)carbamate (1t)

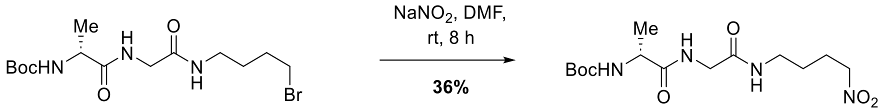

25 → **1t**

Scheme S9

To a mixture of bromide **25** (1.32 mmol, 0.500 g) and DMF (6.6 mL) was added sodium nitrite (2 eq., 2.63 mmol, 0.181 g) and the mixture was stirred at rt. After 8 h the mixture was diluted with H_2O (33 mL, pre-cooled to 4 °C) and extracted with Et_2O (3 × 10 mL). The combined organics were washed with brine (10 mL), dried (Na_2SO_4) and concentrated *in vacuo* yielding a pale-yellow oil (0.36 g). This residue was purified by column chromatography (gradient eluent hexane:EtOAc 50:50 to EtOAc to EtOAc:MeOH 98:2 to 95:5) yielding nitroalkane **1t** (0.171 g, 36%) as a colorless oil.

^{1}H NMR (300 MHz, $CDCl_3$) δ 7.06 (bs, 1H), 6.98 (bs, 1H), 5.19 (d, *J* = 5.2 Hz, 1H), 4.41 (t, *J* = 6.9 Hz, 2H), 4.09 – 4.01 (m, 1H), 3.97 – 3.87 (m, 2H), 3.35 (dq, *J* = 13.4, 6.7 Hz, 1H), 3.21 (dd, *J* = 13.3, 6.6 Hz, 1H), 2.05 – 1.97 (m, 2H), 1.66 – 1.56 (m, 2H), 1.42 (s, 9H), 1.37 (d, *J* = 7.1 Hz, 3H). **^{13}C{^{1}H} NMR** (75 MHz, $CDCl_3$) δ 173.5, 169.3, 156.2, 80.9, 75.2, 51.3, 43.3, 38.5, 28.4, 26.3, 24.6, 17.8. **HRMS** (*m/z*): $[M+H]^+$ calcd for $C_{14}H_{27}N_4O_6^+$, 347.19251; found, 347.19287.

2.1.8 Synthesis of nitroalkane (1u)

2.1.8.1 (2*S*,3*S*,4*S*,5*R*,6*R*)-2-(3-Bromopropoxy)-3,4,5-trimethoxy-6-(methoxymethyl)tetrahydro-2H-pyran (28)

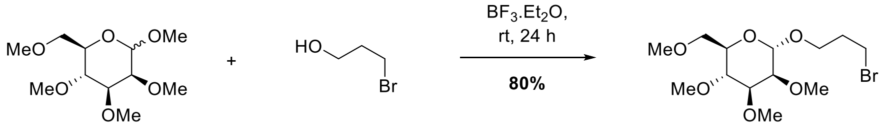

26 **27** **28**

Scheme S10

A mixture of pentamethylmannose (**26**)[12] (1.68 mmol, 0.420 g) and DCM (17 mL) was cooled to 0-5 °C (ice-bath) under argon. $BF_3.Et_2O$ (10 eq., 17 mmol, 2.4 g, 2.1 mL) was added followed by drop wise addition of 3-bromopropanol (**27**) (3 eq., 5.03 mmol, 0.700 g). The mixture was warmed to rt and stirred at rt under argon. After 24 h the reaction mixture was poured onto saturated aqueous $NaHCO_3$ solution (80 mL) and the resulting mixture was extracted with EtOAc (2 × 40 mL). The combined organics were washed with brine (80 mL), dried (Na_2SO_4) and concentrated *in vacuo* yielding brown oil (1.1 g). This residue was purified by column chromatography (gradient eluent hexane:EtOAc 90:10 to 85:15 to 75:25 to 50:50) yielding bromide **28** (0.48 g, 80%) as a pale-yellow oil.

^{1}H NMR (300 MHz, $CDCl_3$) δ 4.89 (d, *J* = 1.8 Hz, 1H), 3.83 (dt, *J* = 10.0, 5.8 Hz, 1H), 3.67 – 3.33 (m, 21H), 2.15 – 2.06 (m, 2H). **^{13}C{^{1}H} NMR** (75 MHz, $CDCl_3$) δ 97.1, 81.4, 77.2, 76.5, 71.8, 71.6, 65.2, 60.8, 59.4, 59.1, 57.9, 32.6, 30.3. **HRMS** (*m/z*): $[M+H]^+$ calcd for $C_{13}H_{26}BrO_6^+$, 357.09073 and 359.08868; found, 357.09080 and 359.08883.

2.1.8.2 (2*R*,3*R*,4*S*,5*S*,6*S*)-3,4,5-Trimethoxy-2-(methoxymethyl)-6-(3-nitropropoxy)tetrahydro-2H-pyran (1u)

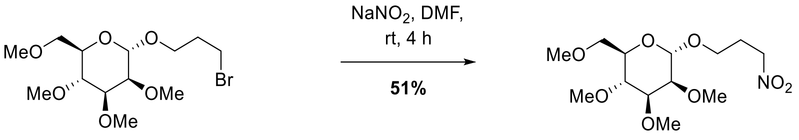

28 1u

Scheme S11

To a mixture of bromide **28** (0.700 mmol, 0.250 g) and DMF (3.5 mL) was added sodium nitrite (2 eq., 1.40 mmol, 0.097 g) and the mixture was stirred at rt. After 4 h the mixture was diluted with H_2O (20 mL, pre-cooled to 4 °C) and the resulting mixture was extracted with EtOAc (3 × 20 mL). The combined organics were washed with brine (20 mL), dried (Na_2SO_4) and concentrated *in vacuo* yielding a pale-yellow oil (0.19 g). This residue was purified by column chromatography (gradient eluent hexane:EtOAc 75:25 to 50:50) yielding nitroalkane **1u** (0.115 g, 51%) as a pale-yellow oil.

^{1}H NMR (600 MHz, $CDCl_3$) δ 4.86 (d, *J* = 1.9 Hz, 1H), 4.55 – 4.41 (m, 2H), 3.82 (ddd, *J* = 10.3, 6.9, 4.7 Hz, 1H), 3.62 – 3.34 (m, 19H), 2.37 – 2.22 (m, 2H). **^{13}C{^{1}H} NMR** (151 MHz, $CDCl_3$) δ 97.3, 81.3, 77.1, 76.5, 73.0, 71.9, 64.2, 60.8, 59.4, 59.1, 57.9, 27.4. **HRMS** (*m/z*): $[M+Na]^+$ calcd for $C_{13}H_{25}NNaO_8^+$, 346.14724; found, 346.14745.

2.1.9 3-(4-Nitrobutyl)thiophene (5g)

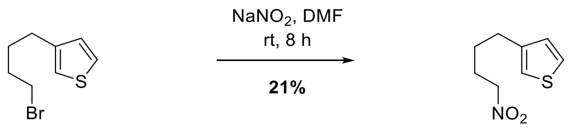

20c 5g

Scheme S12

To a mixture of sodium nitrite (1.2 eq., 6.00 mmol, 0.414 g) and DMF (5 mL) was added bromide **20c**[13] (5.000 mmol, 1.016 g) and the mixture was stirred at rt. After 5 h the mixture was diluted with H_2O (25 mL, pre-cooled to 4 °C) and the resulting mixture was extracted with Et_2O (2 × 10 mL). The combined organics were washed with brine (10 mL), dried (Na_2SO_4) and concentrated *in vacuo* yielding an orange liquid (0.95 g). This residue was purified by column chromatography (gradient eluent hexane to hexane:EtOAc 90:10) yielding impure nitroalkane **5g** (0.36 g) as a pale-yellow oil. This material was purified by reverse-phase HPLC yielding nitroalkane **5g** (0.194 g, 21%) as a yellow oil.

^{1}H NMR (600 MHz, $CDCl_3$) δ 7.27 – 7.26 (m, 1H), 6.97 – 6.90 (m, 2H), 4.38 (t, *J* = 7.0 Hz, 2H), 2.70 (t, *J* = 7.5 Hz, 2H), 2.06 – 2.00 (m, 2H), 1.76 – 1.70 (m, 2H). **^{13}C{^{1}H} NMR** (151 MHz, $CDCl_3$) δ 141.5, 128.0, 125.9, 120.7, 75.6, 29.5, 27.2, 27.0. **HRMS** (*m/z*): $[M+H]^+$ calcd for $C_8H_{12}NO_2S^+$, 186.05833; found, 186.05844.

2.2 Synthesis and characterisation of nitroalkanes C-arylated with thiophenes

2.2.1 General procedure A for C-arylation with thiophenes

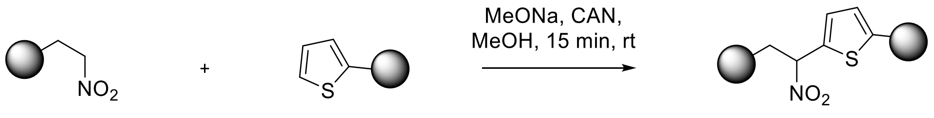

Scheme S13

To a solution of nitroalkane **1** (0.60 mmol, 1 eq.) in MeOH (3.0 mL) was added MeONa (1.3 eq., 0.78 mmol, 25% w/w solution in MeOH), arene **5** (12 mmol, 20 eq.) and CAN (1.32 mmol, 2.2 eq.) and the mixture was left to stir at room temperature (500 rpm). After 15 minutes, a saturated solution of $Na_2S_2O_3$ (10 mL) and water (12 mL) were added and the mixture was extracted with EtOAc (2 × 10 mL). Combined organic phases were washed with brine (10 mL), dried (Na_2SO_4) and concentrated *in vacuo*. The crude mixture was purified by column chromatography.

2.2.2 2-(2-Phenyl-1-nitroethyl)thiophene (3a)

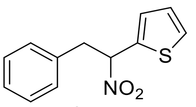

Figure S2

Nitro compound **3a** was prepared according to the general procedure A using nitroalkane **1b** (0.60 mmol, 91 mg), MeONa (1.3 eq., 0.780 mmol, 169 mg of 25% w/w solution in MeOH), thiophene (**5a**) (20 eq., 12 mmol, 1.01 g, 0.961 mL), CAN (2.2 eq., 1.32 mmol, 724 mg) and MeOH (3.0 mL). The crude product was purified by column chromatography (gradient eluent hexane to hexane:EtOAc 95:5 to 90:10) affording nitro compound **3a** (97 mg) as a red oil. This material was further purified by preparative HPLC yielding nitro compound **3a** (46 mg, 33%) as an orange oil.

^{1}H NMR (600 MHz, $CDCl_3$) δ 7.40 (dd, *J* = 5.2, 1.3 Hz, 1H), 7.32 – 7.20 (m, 6H), 7.01 (dd, *J* = 5.1, 3.6 Hz, 1H), 5.94 (dd, *J* = 9.1, 6.0 Hz, 1H), 3.76 (dd, *J* = 14.3, 9.1 Hz, 1H), 3.43 (dd, *J* = 14.3, 6.0 Hz, 1H).
^{13}C{^{1}H} NMR (151 MHz, $CDCl_3$) δ 136.0, 135.1, 129.0, 129.0, 128.8, 127.9, 127.8, 127.1, 87.9, 41.5.
HRMS (*m/z*): $[M+H]^+$ calcd for $C_{12}H_{12}NO_2S^+$, 236.07157; found, 236.07137.

2.2.3 2-Methyl-5-(2-phenyl-1-nitroethyl)thiophene (3b)

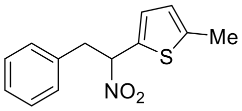

3b

Figure S3

Nitro compound **3b** was prepared according to the general procedure A using nitroalkane **1b** (0.60 mmol, 91 mg), MeONa (1.3 eq., 0.780 mmol, 169 mg of 25% w/w solution of MeONa in MeOH), 2-methylthiophene (**5b**) (20 eq., 12 mmol, 1.2 g, 1.2 mL), CAN (2.2 eq., 1.32 mmol, 724 mg) and MeOH (3.0 mL). The crude product was purified by column chromatography (gradient eluent hexane:DCM 90:10 to 85:15 to 75:25) affording nitro compound **3b** (82 mg, 55%) as a yellow solid.

^{1}H NMR (300 MHz, $CDCl_3$) δ 7.33 – 7.15 (m, 5H), 6.99 (d, *J* = 3.5 Hz, 1H), 6.65 (dq, *J* = 3.5, 1.1 Hz, 1H), 5.84 (dd, *J* = 9.1, 6.1 Hz, 1H), 3.71 (dd, *J* = 14.3, 9.1 Hz, 1H), 3.39 (dd, *J* = 14.2, 6.1 Hz, 1H), 2.48 (d, *J* = 1.1 Hz, 3H). **^{13}C{^{1}H} NMR** (151 MHz, $CDCl_3$) δ 142.9, 135.2, 133.5, 129.0, 129.0, 128.9, 127.7, 125.2, 88.3, 41.2, 15.6. **HRMS** (*m/z*): $[M+H]^+$ calcd for $C_{13}H_{14}NO_2S^+$, 248.07398; found, 248.07392.

2.2.4 2-Methyl-5-(1-nitrohexyl)thiophene (3c)

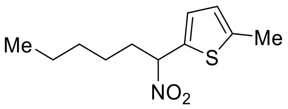

3c

Figure S4

Nitro compound **3c** was prepared according to the general procedure A using nitroalkane **1d** (0.60 mmol, 79 mg), MeONa (1.3 eq., 0.780 mmol, 169 mg of 25% w/w solution of MeONa in MeOH), 2-methylthiophene (**5b**) (20 eq., 12 mmol, 1.2 g, 1.2 mL), CAN (2.2 eq., 1.32 mmol, 724 mg) and MeOH (3.0 mL). The crude product was purified by column chromatography (gradient eluent hexane:Et_2O 99:1 to 98:2) affording nitro compound **3c** (79 mg, 58%) as a yellow oil.

^{1}H NMR (300 MHz, $CDCl_3$) δ 6.98 (d, *J* = 3.5 Hz, 1H), 6.65 (dq, *J* = 3.4, 1.1 Hz, 1H), 5.60 (dd, *J* = 8.4, 7.0 Hz, 1H), 2.50 – 2.34 (m, 4H), 2.18 – 2.00 (m, 1H), 1.40 – 1.23 (m, 6H), 0.93 – 0.84 (m, 3H). **^{13}C{^{1}H} NMR** (75 MHz, $CDCl_3$) δ 142.6, 134.3, 128.6, 125.1, 87.1, 35.2, 31.1, 25.9, 22.4, 15.6, 14.0. **HRMS** (*m/z*): $[M+H]^+$ calcd for $C_{11}H_{18}NO_2S^+$, 228.10528; found, 228.10522.

2.2.4.1 Synthesis of compound 3c on 5 mmol scale

Nitro compound **3c** was prepared according to the general procedure A using nitroalkane **1d** (5.00 mmol, 0.656 g), MeONa (1.3 eq., 6.50 mmol, 1.41 g of 25% w/w solution of MeONa in MeOH), 2-methylthiophene (**5b**) (20 eq., 100 mmol, 9.8 g, 9.7 mL), CAN (2.2 eq., 11.0 mmol, 6.03 g) and MeOH (25 mL). The crude product was purified by column chromatography (gradient eluent hexane:Et_2O 99:1 to 95:5) affording nitro compound **3c** (0.620 g, 55%) as a yellow oil.

2.2.5 2-Methyl-5-(3-methyl-1-nitrobutyl)thiophene (3d)

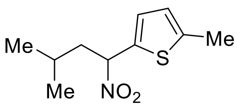

3d

Figure S5

Nitro compound **3d** was prepared according to the general procedure A using nitroalkane **1e** (0.60 mmol, 70 mg), MeONa (1.3 eq., 0.780 mmol, 169 mg of 25% w/w solution of MeONa in MeOH), 2-methylthiophene (**5b**) (20 eq., 12 mmol, 1.2 g, 1.2 mL), CAN (2.2 eq., 1.32 mmol, 724 mg) and MeOH (3.0 mL). The crude product was purified by column chromatography (gradient eluent hexane:Et_2O 99:1 to 98:2) affording nitro compound **3d** (80 mg, 63%) as a brown liquid.

^{1}H NMR (300 MHz, $CDCl_3$) δ 6.99 (d, J = 3.5 Hz, 1H), 6.65 (dq, J = 3.4, 1.1 Hz, 1H), 5.71 (dd, J = 8.5, 6.9 Hz, 1H), 2.48 (d, J = 1.1 Hz, 3H), 2.41 – 2.24 (m, 1H), 1.97 (dt, J = 14.1, 7.2 Hz, 1H), 1.66 – 1.49 (m, 1H), 0.99 (d, J = 6.6 Hz, 3H), 0.95 (d, J = 6.6 Hz, 3H). **^{13}C{^{1}H} NMR** (151 MHz, $CDCl_3$) δ 142.7, 134.3, 128.6, 125.1, 85.5, 43.8, 25.4, 22.3, 22.1, 15.5. **HRMS** (*m/z*): $[M+Na]^+$ calcd for $C_{10}H_{15}NNaO_2S^+$, 236.07157; found, 236.07137.

2.2.6 2-Methyl-5-(1-nitrobut-3-en-1-yl)thiophene (3e)

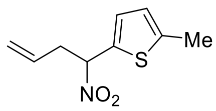

3e

Figure S6

Nitro compound **3e** was prepared according to the general procedure A using nitroalkane **1f** (0.60 mmol, 61 mg), MeONa (1.3 eq., 0.780 mmol, 169 mg of 25% w/w solution of MeONa in MeOH), 2-methylthiophene (**5b**) (20 eq., 12 mmol, 1.2 g, 1.2 mL), CAN (2.2 eq., 1.32 mmol, 724 mg) and MeOH (3.0 mL). The crude product was purified by column chromatography (gradient eluent hexane:Et_2O 99:1 to 98:2) affording nitro compound **3e** (73 mg, 62%) as a yellow liquid.

^{1}H NMR (300 MHz, $CDCl_3$) δ 7.04 – 6.95 (m, 1H), 6.66 (dq, J = 3.4, 1.1 Hz, 1H), 5.80 – 5.61 (m, 2H), 5.27 – 5.14 (m, 2H), 3.22 – 3.08 (m, 1H), 2.92 – 2.79 (m, 1H), 2.48 (d, J = 1.1 Hz, 3H). **^{13}C{^{1}H} NMR** (75 MHz, $CDCl_3$) δ 142.9, 133.4, 131.3, 128.8, 125.2, 120.1, 86.4, 39.1, 15.5. **HRMS** (*m/z*): $[M+H]^+$ calcd for $C_9H_{12}NO_2S^+$, 198.05833; found, 198.05825.

2.2.7 2-Methyl-5-(1-nitropent-3-en-1-yl)thiophene (3f)

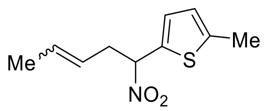

3f

Figure S7

Nitro compound **3f** was prepared according to the general procedure A using nitroalkane **1g** (0.60 mmol, 69 mg, mixture of diastereomers), MeONa (1.3 eq., 0.780 mmol, 169 mg of 25% w/w solution of MeONa in MeOH), 2-methylthiophene (**5b**) (20 eq., 12 mmol, 1.2 g, 1.2 mL), CAN (2.2 eq., 1.32 mmol, 724 mg) and MeOH (3.0 mL). The crude product was purified by column chromatography (gradient eluent hexane to hexane:Et_2O 99:1 to 98:2 to 97:3) affording nitro compound **3f** (73 mg, 58%, mixture of *E* and *Z* diastereomer d.r. 86:14) as a yellow oil.

^{1}H NMR (300 MHz, $CDCl_3$) δ 7.01 – 6.98 (m, 1H of the major diastereomer and 1H of the minor diastereomer), 5.71 – 5.57 (m, 2H of the major diastereomer and 2H of the minor diastereomer), 5.39 – 5.27 (m, 1H of the major diastereomer and 1H of the minor diastereomer), 3.29 – 3.16 (m, 1H of the minor diastereomer), 3.13 – 3.01 (m, 1H of the major diastereomer), 2.94 – 2.72 (m, 1H of the major diastereomer and 1H of the minor diastereomer). 2.48 (m, 3H of the major diastereomer and 3H of the minor diastereomer). **^{13}C{^{1}H} NMR** (151 MHz, $CDCl_3$, signals for both diastereomers reported as observed) δ 142.8, 142.7, 133.7, 131.2, 129.4, 128.8, 128.7, 125.2, 125.1, 123.8, 122.9, 87.0, 86.5, 38.2, 32.8, 18.1, 15.6, 13.1. **HRMS** (m/z): $[M+H]^+$ calcd for $C_{10}H_{13}NO_2S^+$, 212.07398; found, 212.07394.

2.2.8 2-Methyl-5-(1-nitropent-4-yn-1-yl)thiophene (3g)

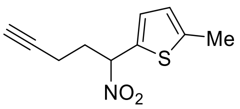

3g

Figure S8

Nitro compound **3g** was prepared according to the general procedure A using nitroalkane **1h** (0.60 mmol, 68 mg), MeONa (1.3 eq., 0.780 mmol, 169 mg of 25% w/w solution of MeONa in MeOH), 2-methylthiophene (**5b**) (20 eq., 12 mmol, 1.2 g, 1.2 mL), CAN (2.2 eq., 1.32 mmol, 724 mg) and MeOH (3.0 mL). The crude product was purified by column chromatography (gradient eluent hexane:Et_2O 99:1 to 98:2) affording nitro compound **3g** (71 mg, 57%) as a brown liquid.

^{1}H NMR (300 MHz, $CDCl_3$) δ 7.02 (bd, *J* = 3.5 Hz, 1H), 6.67 (dq, *J* = 3.4, 1.1 Hz, 1H), 5.90 – 5.79 (m, 1H), 2.72 – 2.52 (m, 1H), 2.48 (d, *J* = 1.1 Hz, 3H), 2.40 – 2.17 (m, 3H), 2.09 – 2.03 (m, 1H). **^{13}C{^{1}H} NMR** (151 MHz, $CDCl_3$) δ 143.1, 133.0, 129.2, 125.3, 85.2, 81.2, 70.7, 33.4, 15.58, 15.57. **HRMS** (*m/z*): $[M+Na]^+$ calcd for $C_{10}H_{11}NNaO_2S^+$, 232.04039; found, 232.04027.

2.2.9 2-(3-Bromophenethyl)-5-methylthiophene (3h)

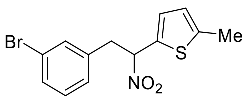

3h

Figure S9

Nitro compound **3h** was prepared according to the general procedure A using nitroalkane **1l** (0.600 mmol, 138 mg), MeONa (1.3 eq., 0.780 mmol, 169 mg of of 25% w/w solution of MeONa in MeOH), 2-methylthiophene (**5b**) (20 eq., 12 mmol, 1.2 g, 1.2 mL), CAN (2.2 eq., 1.32 mmol, 724 mg) and MeOH (3.0 mL). The crude product was purified by column chromatography (gradient eluent hexane:Et_2O 99:1 to 90:10) affording nitro compound **3h** (102 mg, 52%) as a yellow oil.

^{1}H NMR (300 MHz, $CDCl_3$) δ 7.42 – 7.33 (m, 2H), 7.20 – 7.08 (m, 2H), 7.00 (d, *J* = 3.5 Hz, 1H), 6.66 (dq, *J* = 3.4, 1.1 Hz, 1H), 5.82 (dd, *J* = 9.1, 6.1 Hz, 1H), 3.68 (dd, *J* = 14.3, 9.1 Hz, 1H), 3.36 (dd, *J* = 14.4, 6.1 Hz, 1H), 2.49 (d, *J* = 1.1 Hz, 3H). **^{13}C NMR** (151 MHz, $CDCl_3$) δ 143.1, 137.4, 133.0, 132.1, 130.9, 130.5, 129.1, 127.7, 125.3, 122.9, 87.8, 40.6, 15.6. **HRMS** (*m/z*): $[M+Na]^+$ calcd for $C_{13}H_{12}BrNNaO_2S^+$, 347.96643 and 349.96439; found, 347.96626 and 349.96409.

2.2.10 2-(2-(3-Fluorophenyl)-1-nitroethyl)-5-methylthiophene (3i)

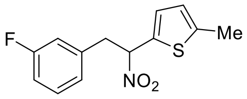

3i

Figure S10

Nitro compound **3i** was prepared according to the general procedure A using nitroalkane **1k** (0.600 mmol, 101 mg), MeONa (1.3 eq., 0.780 mmol, 169 mg of 25% w/w solution of MeONa in MeOH), 2-methylthiophene (**5b**) (20 eq., 12 mmol, 1.2 g, 1.2 mL), CAN (2.2 eq., 1.32 mmol, 724 mg) and MeOH (3.0 mL). The crude product was purified by column chromatography (gradient eluent hexane:Et_2O 99:1 to 95:5) affording nitro compound **3i** (93 mg, 58%) as a yellow liquid.

^{1}H NMR (300 MHz, $CDCl_3$) δ 7.30 – 7.22 (m, 1H), 7.02 – 6.86 (m, 4H), 6.65 (dq, *J* = 3.4, 1.1 Hz, 1H), 5.83 (dd, *J* = 9.0, 6.2 Hz, 1H), 3.71 (dd, *J* = 14.3, 9.0 Hz, 1H), 3.39 (dd, *J* = 14.3, 6.2 Hz, 1H), 2.49 (d, *J* = 1.1 Hz, 3H). **^{13}C{^{1}H} NMR** (151 MHz, $CDCl_3$) δ 163.0 (d, *J* = 246.8 Hz), 143.1, 137.5 (d, *J* = 7.5 Hz), 133.0, 130.5 (d, *J* = 8.3 Hz), 129.0, 125.3, 124.7 (d, *J* = 3.0 Hz), 116.1 (d, *J* = 21.7 Hz), 114.8 (d, *J* = 21.0 Hz), 87.9, 40.8 (d, *J* = 1.8 Hz), 15.6. **^{19}F NMR** (282 MHz, $CDCl_3$) δ 79.45 – 79.34 (m). **HRMS** (*m/z*): $[M+H]^+$ calcd for $C_{13}H_{13}FNO_2S^+$, 266.06455; found, 266.06447.

2.2.11 2-(2-(4-Fluorophenyl)-1-nitroethyl)-5-methylthiophene (3j)

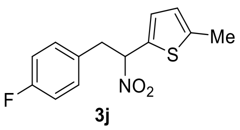

3j

Figure S11

Nitro compound **3j** was prepared according to the general procedure A using nitroalkane **1j** (0.600 mmol, 101 mg), MeONa (1.3 eq., 0.780 mmol, 169 mg of 25% w/w solution of MeONa in MeOH), 2-methylthiophene (**5b**) (20 eq., 12 mmol, 1.2 g, 1.2 mL), CAN (2.2 eq., 1.32 mmol, 724 mg) and MeOH (3.0 mL). The crude product was purified by column chromatography (gradient eluent hexane:Et_2O 99:1 to 98:2) affording nitro compound **3j** (80 mg, 50%) as an orange oil.

^{1}H NMR (300 MHz, $CDCl_3$) δ 7.18 – 7.11 (m, 2H), 7.02 – 6.93 (m, 3H), 6.65 (dq, J = 3.4, 1.1 Hz, 1H), 5.79 (dd, J = 9.0, 6.2 Hz, 1H), 3.68 (dd, J = 14.3, 9.1 Hz, 1H), 3.36 (dd, J = 14.3, 6.2 Hz, 1H), 2.49 (d, J = 1.3 Hz, 3H). **^{13}C{^{1}H} NMR** (151 MHz, $CDCl_3$) δ 162.3 (d, J = 246.3 Hz), 143.0, 133.1, 130.9 (d, J = 3.4 Hz), 130.7 (d, J = 8.1 Hz), 129.0, 125.3, 115.9 (d, J = 21.4 Hz), 88.3, 40.4, 15.6. **^{19}F NMR** (282 MHz, $CDCl_3$) δ -114.65 – -114.79 (m). **HRMS** (*m/z*): $[M+H]^+$ calcd for $C_{13}H_{13}FNO_2S^+$, 266.06455; found, 266.06445.

2.2.12 2-(2-(3-Methoxyphenyl)-1-nitroethyl)-5-methylthiophene (3k)

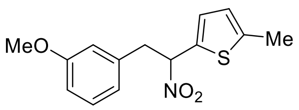

3k

Figure S12

Nitro compound **3k** was prepared according to the general procedure A using nitroalkane **1m** (1.92 mmol, 347 mg), MeONa (1.30 eq., 2.49 mmol, 538 mg of 25% w/w solution of MeONa in MeOH), 2-methylthiophene (**5b**) (20 eq., 38 mmol, 3.8 g, 3.7 mL), CAN (2.20 eq., 4.21 mmol, 2.31 g) and MeOH (9.6 mL). The crude product was purified by column chromatography (gradient eluent hexane:EtOAc 99:1 to 95:5 to 90:10) affording nitro compound **3k** (302 mg, 57%) as a yellow oil.

^{1}H NMR (300 MHz, $CDCl_3$) δ 7.24 – 7.16 (m, 1H), 7.01 – 6.98 (m, 1H), 6.82 – 6.74 (m, 2H), 6.72 – 6.70 (m, 1H), 6.65 (dq, J = 3.4, 1.1 Hz, 1H), 5.84 (dd, J = 9.1, 6.1 Hz, 1H), 3.76 (s, 3H), 3.69 (dd, J = 14.2, 9.1 Hz, 1H), 3.36 (dd, J = 14.2, 6.1 Hz, 1H), 2.48 (d, J = 1.1 Hz, 3H). **^{13}C{^{1}H} NMR** (151 MHz, $CDCl_3$) δ 159.9, 142.9, 136.7, 133.5, 130.0, 128.9, 125.2, 121.3, 114.8, 113.1, 88.1, 55.3, 41.2, 15.6. **HRMS** (*m/z*): $[M+H]^+$ calcd for $C_{14}H_{16}NO_3S^+$, 278.08454; found, 278.08437.

2.2.13 4-(5-Methylthiophen-2-yl)-4-nitrobutan-1-ol (3l)

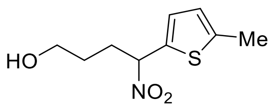

3l

Figure S13

Nitro compound **3l** was prepared according to the general procedure A using nitroalkane **1n** (0.60 mmol, 71 mg), MeONa (1.3 eq., 0.780 mmol, 169 mg of 25% w/w solution of MeONa in MeOH), 2-methylthiophene (**5b**) (20 eq., 12 mmol, 1.2 g, 1.2 mL), CAN (2.2 eq., 1.32 mmol, 724 mg) and MeOH (3.0 mL). The crude product was purified by column chromatography (gradient eluent hexane:Et_2O 85:15 to 50:50) affording nitro compound **3l** (40 mg, 31%) as a dark red oil.

^{1}H NMR (300 MHz, $CDCl_3$) δ 7.00 (bd, J = 3.6 Hz, 1H), 6.66 (dq, J = 3.4, 1.1 Hz, 1H), 5.69 (dd, J = 8.4, 6.9 Hz, 1H), 3.80 – 3.60 (m, 2H), 2.62 – 2.45 (m, 4H), 2.33 – 2.19 (m, 1H), 1.68 – 1.55 (m, 2H). **^{13}C{^{1}H} NMR** (151 MHz, $CDCl_3$) δ 142.8, 133.9, 128.8, 125.2, 86.8, 61.8, 31.8, 29.1, 15.6. **HRMS** (*m/z*): $[M+H]^+$ calcd for $C_9H_{14}NO_3S^+$, 216.06889; found, 216.06879.

2.2.14 2-(3-Methoxy-1-nitropropyl)-5-methylthiophene (3m)

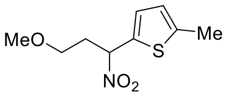

3m

Figure S14

Nitro compound **3m** was prepared according to the general procedure A using nitroalkane **1o** (0.60 mmol, 71 mg), MeONa (1.3 eq., 0.780 mmol, 169 mg of 25% w/w solution of MeONa in MeOH), 2-methylthiophene (**5b**) (20 eq., 12 mmol, 1.2 g, 1.2 mL), CAN (2.2 eq., 1.32 mmol, 724 mg) and MeOH (3.0 mL). The crude product was purified by column chromatography (gradient eluent hexane:EtOAc 95:5 to 90:10) affording nitro compound **3m** (75 mg, 58%) as a yellow liquid.

^{1}H NMR (300 MHz, $CDCl_3$) δ 7.00 (d, J = 3.5 Hz, 1H), 6.66 (dq, J = 3.4, 1.1 Hz, 1H), 5.87 (dd, J = 8.4, 6.5 Hz, 1H), 3.43 – 3.37 (m, 2H), 3.31 (s, 3H), 2.78 – 2.63 (m, 1H), 2.48 (d, J = 1.1 Hz, 3H), 2.40 – 2.27 (m, 1H). **^{13}C{^{1}H} NMR** (75 MHz, $CDCl_3$) δ 142.7, 133.8, 128.9, 125.2, 83.8, 68.2, 59.0, 35.2, 15.6. **HRMS** (*m/z*): $[M+H]^+$ calcd for $C_9H_{14}NO_3S^+$, 216.06889; found, 216.06888.

2.2.15 *tert*-Butyl(4-(5-methylthiophen-2-yl)-4-nitrobutoxy)diphenylsilane (3n)

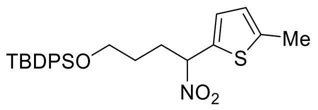

3n

Figure S15

Nitro compound **3n** was prepared according to the general procedure A using nitroalkane **1p** (0.20 mmol, 72 mg), MeONa (1.3 eq., 0.26 mmol, 56 mg of 25% w/w solution of MeONa in MeOH), 2-methylthiophene (**5b**) (20 eq., 4.00 mmol, 0.393 g, 0.387 mL), CAN (2.2 eq., 0.440 mmol, 241 mg) and MeOH (1.0 mL). The crude product was purified by column chromatography (gradient eluent hexane:Et_2O 99:1 to 98:2 to 95:5) affording nitro compound **3n** (46 mg, 51%) as a yellow oil.

^{1}H NMR (600 MHz, $CDCl_3$) δ 7.66 – 7.62 (m, 4H), 7.45 – 7.36 (m, 6H), 6.94 (d, *J* = 3.5 Hz, 1H), 6.65 (dq, *J* = 3.5, 1.2 Hz, 1H), 5.66 (t, *J* = 7.7 Hz, 1H), 3.74 – 3.63 (m, 2H), 2.57 – 2.48 (m, 4H), 2.32 – 2.23 (m, 1H), 1.64 – 1.55 (m, 2H), 1.05 (s, 9H). **^{13}C{^{1}H} NMR** (151 MHz, $CDCl_3$) δ 142.7, 135.7, 135.7, 134.1, 133.7, 129.9, 129.9, 128.7, 127.9, 127.9, 125.1, 86.75, 62.8, 31.9, 28.9, 27.0, 19.3, 15.6. **HRMS** (m/z): $[M+Na]^+$ calcd for $C_{25}H_{31}NO_3SSi^+$, 476.16861; found, 476.16842.

2.2.16 2-(2-(5-Methylthiophen-2-yl)-2-nitroethyl)-1,3-dioxolane (3o)

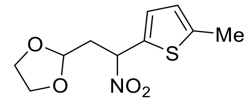

3o

Figure S16

Nitro compound **3o** was prepared according to the general procedure A using nitroalkane **1c** (0.60 mmol, 88 mg), MeONa (1.3 eq., 0.780 mmol, 169 mg of 25% w/w solution of MeONa in MeOH), 2-methylthiophene (**5b**) (20 eq., 12 mmol, 1.2 g, 1.2 mL), CAN (2.2 eq., 1.32 mmol, 724 mg) and MeOH (3.0 mL). The crude product was purified by column chromatography (gradient eluent hexane:EtOAc 95:5 to 90:10) affording nitro compound **3o** (95 mg, 65%) as a brown liquid.

^{1}H NMR (300 MHz, $CDCl_3$) δ 7.01 (bd, *J* = 3.6 Hz, 1H), 6.65 (dq, *J* = 3.4, 1.1 Hz, 1H), 5.86 (dd, *J* = 9.3, 4.8 Hz, 1H), 4.94 (t, 1H), 4.05 – 3.93 (m, 2H), 3.92 – 3.80 (m, 2H), 2.97 (ddd, *J* = 14.7, 9.3, 4.3 Hz, 1H), 2.47 (d, *J* = 1.1 Hz, 3H), 2.42 (ddd, *J* = 14.7, 4.9, 3.8 Hz, 1H). **^{13}C{^{1}H} NMR** (151 MHz, $CDCl_3$) δ 142.8, 133.9, 128.6, 125.2, 101.0, 81.9, 65.5, 65.4, 38.3, 15.5. **HRMS** (*m/z*): $[M+H]^+$ calcd for $C_{10}H_{14}NO_4S^+$, 244.06381; found, 244.06364.

2.2.17 Ethyl 4-(5-methylthiophen-2-yl)-4-nitrobutanoate (3p)

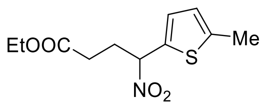

3p

Figure S17

Nitro compound **3p** was prepared according to the general procedure A using nitroalkane **1q** (0.60 mmol, 97 mg), MeONa (1.3 eq., 0.780 mmol, 169 mg of 25% w/w solution of MeONa in MeOH), 2-methylthiophene (**5b**) (20 eq., 12 mmol, 1.2 g, 1.2 mL), CAN (2.2 eq., 1.32 mmol, 724 mg) and MeOH (3.0 mL). The crude product (70% NMR yield) was purified by column chromatography (gradient eluent hexane:acetone 99:1 to 98:2) affording nitro compound **3p** (40 mg, 26%) as a yellow liquid.

^{1}H NMR (300 MHz, $CDCl_3$) δ 7.00 (d, *J* = 3.5 Hz, 1H), 6.66 (dq, *J* = 3.4, 1.1 Hz, 1H), 5.81 – 5.71 (m, 1H), 4.15 (q, *J* = 7.1 Hz, 2H), 2.79 – 2.65 (m, 1H), 2.52 – 2.35 (m, 6H), 1.26 (t, *J* = 7.1 Hz, 3H). **^{13}C{^{1}H} NMR** (151 MHz, $CDCl_3$) δ 171.8, 143.0, 133.2, 129.1, 125.3, 85.7, 61.1, 30.4, 30.0, 15.6, 14.3. **HRMS** (*m/z*): $[M+H]^+$ calcd for $C_{11}H_{16}NO_4S^+$, 258.07946; found, 258.07942.

2.2.18 1-(4-(5-Methylthiophen-2-yl)-4-nitrobutyl)pyrrolidin-2-one (3q)

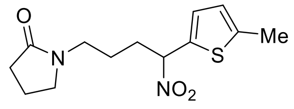

3q

Figure S18

Nitro compound **3q** was prepared according to the general procedure A using nitroalkane **1r** (0.600 mmol, 112 mg), MeONa (1.3 eq., 0.780 mmol, 169 mg of 25% w/w solution of MeONa in MeOH), 2-methylthiophene (**5b**) (20 eq., 12 mmol, 1.2 g, 1.2 mL), CAN (2.2 eq., 1.32 mmol, 724 mg) and MeOH (3.0 mL). The crude product was purified by column chromatography (gradient eluent hexane:EtOAc 90:10 to 50:50 to 15:85 to EtOAc) affording nitro compound **3q** (66 mg, 39%) as a brown oil.

^{1}H NMR (300 MHz, $CDCl_3$) δ 6.99 (d, *J* = 3.5 Hz, 1H), 6.65 (dq, *J* = 3.4, 1.1 Hz, 1H), 5.70 (dd, *J* = 8.7, 6.5 Hz, 1H), 3.43 – 3.24 (m, 4H), 2.51 – 2.35 (m, 6H), 2.18 – 1.95 (m, 3H), 1.66 – 1.51 (m, 2H). **^{13}C{^{1}H} NMR** (75 MHz, $CDCl_3$) δ 175.3, 142.8, 133.7, 128.9, 125.2, 86.4, 47.1, 41.4, 32.3, 31.0, 24.0, 18.0, 15.5. **HRMS** (*m/z*): $[M+H]^+$ calcd for $C_{13}H_{19}N_2O_3S^+$, 283.11109; found, 283.11107.

2.2.19 *tert*-Butyl-(4-(5-(hydroxymethyl)thiophen-2-yl)-4-nitrobutyl)carbamate (3r)

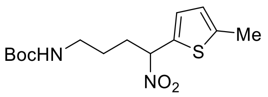

3r

Figure S19

Nitro compound **3r** was prepared according to the general procedure A using nitroalkane **1s** (0.60 mmol, 131 mg), MeONa (1.3 eq., 0.780 mmol, 169 mg of 25% w/w solution of MeONa in MeOH), 2-methylthiophene (**5b**) (20 eq., 12 mmol, 1.2 g, 1.2 mL), CAN (2.2 eq., 1.32 mmol, 724 mg) and MeOH (3.0 mL). The crude product was purified by column chromatography (gradient eluent hexane:Et_2O 90:10 to 50:50) affording nitro compound **3r** (80 mg, 42%) as a brown oil.

^{1}H NMR (600 MHz, $CDCl_3$) δ 6.98 (d, *J* = 3.5 Hz, 1H), 6.66 – 6.63 (m, 1H), 5.65 („t", *J* = 7.6 Hz, 1H), 4.57 (bs, 1H), 3.19 – 3.13 (m, 2H), 2.47 (d, *J* = 1.1 Hz, 3H), 2.47 – 2.39 (m, 1H), 2.17 – 2.11 (m, 1H), 1.58 – 1.49 (m, 2H), 1.43 (s, 9H). **^{13}C{^{1}H} NMR** (151 MHz, $CDCl_3$) δ 156.1, 142.8, 133.8, 128.8, 125.2, 86.6, 79.6, 39.6, 32.3, 28.5, 26.9, 15.5. **HRMS** (m/z): $[M+Na]^+$ calcd for $C_{14}H_{22}N_2O_4SNa^+$, 337.11925; found, 337.11950.

2.2.21 *tert*-Butyl ((*2R*)-1-((2-((4-(5-methylthiophen-2-yl)-4-nitrobutyl)amino)-2-oxoethyl)amino)-1-oxopropan-2-yl)carbamate (3s)

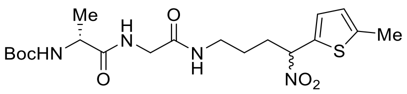

3s

Figure S20

Nitro compound **3s** was prepared according to the general procedure A using nitroalkane **1t** (0.20 mmol, 69 mg), MeONa (1.3 eq., 0.26 mmol, 56 mg of 25% w/w solution of MeONa in MeOH), 2-methylthiophene (**5b**) (20 eq., 4.00 mmol, 0.393 g, 0.387 mL), CAN (2.2 eq., 0.440 mmol, 241 mg) and MeOH (1.0 mL). The crude product was purified by column chromatography (gradient eluent hexane:EtOAc 50:50 to EtOAc to EtOAc:MeOH 90:10) affording nitro compound **3s** (53 mg, 60%) as a brown oil.

NMR data are reported as observed for both diastereomers.

^{1}H NMR (300 MHz, $CDCl_3$) δ 7.03 – 6.85 (m, 3H), 6.64 (dq, *J* = 3.4, 1.1 Hz, 1H), 5.66 (dd, *J* = 8.5, 6.8 Hz, 1H), 5.13 (d, *J* = 5.6 Hz, 1H), 4.12 – 4.00 (m, 1H), 4.00 – 3.84 (m, 2H), 3.45 – 3.13 (m, 2H), 2.56 – 2.34 (m, 4H), 2.22 – 2.03 (m, 1H), 1.66 – 1.51 (m, 2H), 1.43 (s, 9H), 1.37 (d, *J* = 7.1 Hz, 3H). **^{13}C{^{1}H} NMR** (75 MHz, $CDCl_3$) δ 173.4, 169.3, 156.2, 142.8, 133.7, 128.9, 125.2, 86.5, 80.9, 51.3, 43.3, 38.5, 32.3, 28.4, 26.2, 17.8, 15.5. **HRMS** (*m/z*): $[M+H]^+$ calcd for $C_{19}H_{31}N_4O_6S^+$, 443.19588; found, 443.19598.

2.2.20 (*2S,3S,4S,5R,6R*)-3,4,5-Trimethoxy-2-(methoxymethyl)-6-(3-(5-methylthiophen-2-yl)-3-nitropropoxy)tetrahydro-2H-pyran (3t)

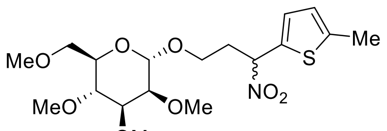

3t

Figure S21

Nitro compound **3t** was prepared according to the general procedure A using nitroalkane **1u** (0.155 mmol, 50 mg), MeONa (1.3 eq., 0.20 mmol, 43 mg of 25% w/w solution of MeONa in MeOH), 2-methylthiophene (**5b**) (20 eq., 3.09 mmol, 0.304 g, 0.299 mL), CAN (2.2 eq., 0.340 mmol, 187 mg) and MeOH (0.77 mL). The crude product was purified by column chromatography (gradient eluent hexane to hexane:EtOAc 75:25 to 65:35 to 50:50 to EtOAc) affording nitro compound **3t** (31 mg, 48%, d.r. 50:50) as a brown oil.

NMR data are reported as observed for both diastereomers.
^{1}H NMR (600 MHz, $CDCl_3$) δ 7.01 – 6.99 (m, 2H), 6.66 – 6.65 (m, 2H), 6.85 - 6.75 (m, 2H), 4.82 (d, *J* = 1.9 Hz, 1H), 4.78 (d, *J* = 1.9 Hz, 1H), 3.83 – 3.72 (m, 2H), 3.59 – 3.35 (m, 38H), 2.79- 2.77 (m, 2H), 2.47 (d, J = 1.2 Hz, 6H), 2.39- 2.32 (m, 1H). **^{13}C{^{1}H} NMR** (151 MHz, $CDCl_3$) δ 142.9, 133.44, 133.41, 129.0, 128.8, 125.27, 125.23, 97.5, 97.3, 84.2, 84.1, 81.2, 77.1, 77.0, 76.41, 76.36, 71.75, 71.72, 71.67, 63.9, 63.7, 60.79, 60.76, 60.72, 59.30, 58.0, 57.9, 34.9, 34.7, 15.5. **HRMS** (*m/z*): $[M+H]^+$ calcd for $C_{18}H_{30}NO_8S^+$, 420.16866; found, 420.16874.

2.2.22 2-(2-(5-Hexylthiophen-2-yl)-2-nitroethyl)-1,3-dioxolane (3u)

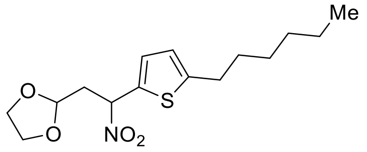

3u

Figure S22

Nitro compound **3u** was prepared according to the general procedure A using nitroalkane **1c** (0.60 mmol, 88 mg), MeONa (1.3 eq., 0.780 mmol, 169 mg of 25% w/w solution of MeONa in MeOH), 2-hexylthiophene (**5c**) (20 eq., 12 mmol, 2.0 g, 2.2 mL), CAN (2.2 eq., 1.32 mmol, 724 mg) and MeOH (3.0 mL). The crude product was purified by column chromatography (gradient eluent hexane:EtOAc 95:5 to 90:10) affording nitro compound **3u** (104 mg, 55%) as a brown liquid.

^{1}H NMR (600 MHz, $CDCl_3$) δ 7.02 (d, *J* = 3.6 Hz, 1H), 6.66 (dt, *J* = 3.6, 1.1 Hz, 1H), 5.88 (dd, *J* = 9.5, 4.6 Hz, 1H), 4.95 (t, *J* = 4.0 Hz, 1H), 4.03 – 3.94 (m, 2H), 3.90 – 3.83 (m, 2H), 2.99 (ddd, *J* = 14.7, 9.5, 4.3 Hz, 1H), 2.80 – 2.74 (m, 2H), 2.42 (ddd, *J* = 14.7, 4.7, 3.8 Hz, 1H), 1.64 (q, *J* = 7.4 Hz, 2H), 1.37 – 1.26 (m, 6H), 0.91 – 0.86 (m, 3H). **^{13}C{^{1}H} NMR** (151 MHz, $CDCl_3$) δ 149.0, 133.5, 128.4, 124.0, 101.0, 81.9, 65.5, 65.4, 38.3, 31.6, 31.6, 30.3, 28.9, 22.7, 14.2. **HRMS** (*m/z*): $[M+H]^+$ calcd for $C_{15}H_{24}NO_4S^+$, 314.14206; found, 314.14218.

2.2.23 2-(2-(5-Bromothiophen-2-yl)ethyl)-1,3-dioxolane (3v)

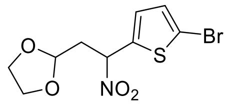

3v

Figure S23

Nitro compound **3v** was prepared according to the general procedure A using nitroalkane **1c** (0.60 mmol, 88 mg), MeONa (1.3 eq., 0.780 mmol, 169 mg of 25% w/w solution of MeONa in MeOH), 2-bromothiophene (**5d**) (20 eq., 12.0 mmol, 1.96 g, 1.16 mL), CAN (2.2 eq., 1.32 mmol, 724 mg) and MeOH (3.0 mL). The crude product was purified by column chromatography (gradient eluent hexane:EtOAc 90:10 to 85:15) affording nitro compound **3v** (73 mg, 40%) as a brown oil.

^{1}H NMR (300 MHz, $CDCl_3$) δ 7.01 – 6.95 (m, 2H), 5.86 (dd, *J* = 9.4, 4.7 Hz, 1H), 4.95 (t, *J* = 3.9 Hz, 1H), 4.05 – 3.93 (m, 2H), 3.92 – 3.82 (m, 2H), 2.97 (ddd, *J* = 14.8, 9.4, 4.2 Hz, 1H), 2.40 (ddd, *J* = 14.8, 4.8, 3.6 Hz, 1H). **^{13}C{^{1}H} NMR** (151 MHz, $CDCl_3$) δ 137.7, 129.9, 129.0, 115.2, 100.7, 81.5, 65.5, 65.4, 38.3. **HRMS** (*m/z*): $[M+H]^+$ calcd for $C_9H_{10}BrNO_4S^+$, 307.95867 and 309.95662; found, 307.95885 and 309.95684.

2.2.24 (5-(1-Nitro-2-phenylethyl)thiophen-2-yl)methanol (3w)

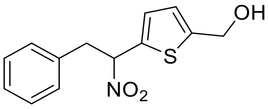

3w

Figure S24

Nitro compound **3w** was prepared according to the general procedure A using nitroalkane **1b** (0.60 mmol, 91 mg), MeONa (1.3 eq., 0.780 mmol, 169 mg of 25% w/w solution of MeONa in MeOH), 2-hydroxymethylthiophene (**5e**) (20 eq., 12.0 mmol, 1.37 g, 1.13 mL), CAN (2.2 eq., 1.32 mmol, 724 mg) and MeOH (3.0 mL). The crude product was purified by column chromatography (gradient eluent hexane to hexane:EtOAc 90:10) affording nitro compound **3w** (96 mg, 61%) as an orange oil.

^{1}H NMR (600 MHz, $CDCl_3$) δ 7.31 – 7.24 (m, 3H), 7.20 – 7.16 (m, 2H), 7.08 (bd, *J* = 3.6 Hz, 1H), 6.89 (dt, *J* = 3.6, 0.9 Hz, 1H), 5.89 (ddd, *J* = 9.0, 6.2, 0.6 Hz, 1H), 4.81 (d, *J* = 0.9 Hz, 2H), 3.74 (dd, *J* = 14.3, 9.1 Hz, 1H), 3.41 (dd, *J* = 14.3, 6.2 Hz, 1H), 1.92 (s, 1H). **^{13}C{^{1}H} NMR** (151 MHz, $CDCl_3$) δ 146.9, 135.9, 135.0, 129.0, 128.9, 128.7, 127.8, 125.0, 88.1, 60.3, 41.3. **HRMS** (*m/z*): $[M+H]^+$ calcd for $C_{13}H_{14}NO_3S^+$, 264.06889; found, 264.06887.

2.2.25 (5-(2-(1,3-Dioxolan-2-yl)-1-nitroethyl)thiophen-2-yl)methanol (3x)

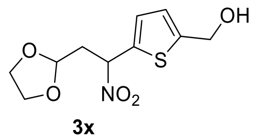

Figure S25

Nitro compound **3x** was prepared according to the general procedure A using nitroalkane **1c** (0.60 mmol, 88 mg), MeONa (1.3 eq., 0.780 mmol, 169 mg of 25% w/w solution of MeONa in MeOH), 2-hydroxymethylthiophene (**5e**) (20 eq., 12.0 mmol, 1.37 g, 1.13 mL), CAN (2.2 eq., 1.32 mmol, 724 mg) and MeOH (3.0 mL). The crude product was purified by column chromatography (gradient eluent DCM:acetone 90:10 to 50:50) affording nitro compound **3x** (63 mg, 41%) as a red oil.

^{1}H NMR (300 MHz, $CDCl_3$) δ 7.09 (d, *J* = 3.6 Hz, 1H), 6.89 (dt, *J* = 3.6, 0.9 Hz, 1H), 5.91 (dd, *J* = 9.4, 4.7 Hz, 1H), 4.95 (t, *J* = 4.0 Hz, 1H), 4.79 (d, *J* = 0.9 Hz, 2H), 4.06 – 3.93 (m, 2H), 3.93 – 3.81 (m, 2H), 3.00 (ddd, *J* = 14.7, 9.4, 4.2 Hz, 1H), 2.42 (ddd, *J* = 14.8, 4.7, 3.7 Hz, 1H), 2.04 (bs, 1H). **^{13}C{^{1}H} NMR** (151 MHz, $CDCl_3$) δ 146.9, 136.2, 128.4, 125.0, 100.8, 81.7, 65.5, 65.4, 60.1, 38.3. **HRMS** (m/z): [M-H]$^-$ calcd for $C_{10}H_{12}NO_5S^-$, 258.04417; found, 258.04330.

2.2.26 2-(2-(4-Methylthiophen-2-yl)-2-nitroethyl)-1,3-dioxolane (3y) and 2-(2-(3-methylthiophen-2-yl)-2-nitroethyl)-1,3-dioxolane (3y′)

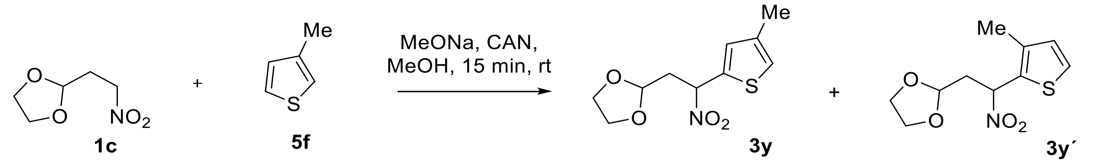

Scheme S14

To a solution of nitroalkane **1c** (0.40 mmol, 59 mg) in MeOH (2.0 mL) was added MeONa (1.30 eq., 0.520 mmol, 112 mg of 25% w/w solution of MeONa in MeOH), 3-methylthiophene (**5f**) (20 eq., 8.00 mmol, 0.785 g, 0.773 mL) and CAN (2.20 eq., 0.880 mmol, 482 mg) and the mixture was left to stir at room temperature (500 rpm). After 15 minutes, saturated solution of $Na_2S_2O_3$ (8 mL) and water (8 mL) were added and the mixture was extracted with EtOAc (2 × 8 mL). The combined organic phases were washed with brine (8 mL), dried (Na_2SO_4) and concentrated *in vacuo*. The crude mixture was purified by column chromatography (gradient eluent hexane to hexane:EtOAc 95:5 to 90:10) affording a mixture of nitro compound **3y** and **3y′** (57 mg, 70%, ratio of regioisomers 75:25) as a brown oil.

^{1}H NMR (300 MHz, $CDCl_3$) δ 7.30 – 7.27 (m, 1H of the major isomer), 7.04 – 7.03 (m, 1H of the minor isomer), 6.94 – 6.93 (m, 1H of the minor isomer), 6.84 – 6.82 (m, 1H of the major isomer), 6.05 – 6.00 (m, 1H of the major isomer), 5.89 (dd, *J* = 9.4, 4.7 Hz, 1H of the minor isomer), 4.96 – 4.93 (m, 1H of teh major isomer and 1H of the minor isomer), 4.05 – 3.82 (m, 4H of the major isomer and 4H of the minor isomer), 3.05 – 2.95 (1H of the major isomer and 1H of the minor isomer), 2.46 – 2.38 (m, 1H of the major isomer and 1H of the minor isomer), 2.34 – 2.33 (m, 3H of the major isomer), 2.23 (m, 1H of the minor isomer). **^{13}C{^{1}H} NMR** (151 MHz, $CDCl_3$, NMR data are reported as observed for both regioisomers) δ 138.8, 137.8, 136.1, 130.6, 130.5, 130.1, 126.3, 122.9, 101.0, 100.9, 81.6, 79.9, 65.5, 65.44, 65.37, 65.36, 38.5, 38.4, 15.7, 14.1. **HRMS** (m/z): [M+H]$^+$ calcd for $C_{10}H_{14}NO_4S^+$, 244.06381; found, 244.06378.

2.2.26 7-Nitro-4,5,6,7-tetrahydrobenzo[b]thiophene (3z)

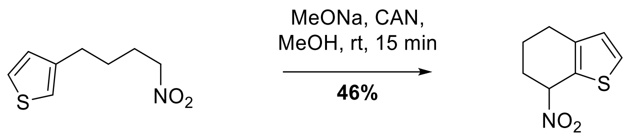

Scheme S15

To a solution of nitroalkane **5g** (0.40 mmol, 76 mg) in MeOH (2.0 mL) was added MeONa (1.30 eq., 0.520 mmol, 112 mg of 25% w/w solution of MeONa in MeOH) and CAN (2.20 eq., 0.880 mmol, 482 mg) and the mixture was left to stir at room temperature (500 rpm). After 15 minutes, saturated solution of $Na_2S_2O_3$ (8 mL) and water (8 mL) were added and the mixture was extracted with EtOAc (2 × 8 mL). The combined organic phases were washed with brine (8 mL), dried (Na_2SO_4) and concentrated *in vacuo*. The crude mixture was purified by column chromatography (gradient eluent hexane:Et_2O 95:5 to 90:10) affording nitro compound **3z** (34 mg, 46%) as a brown oil.

^{1}H NMR (300 MHz, $CDCl_3$) δ 7.35 (d, J = 5.1 Hz, 1H), 6.89 – 6.79 (m, 1H), 5.67 – 5.60 (m, 1H), 2.91 – 2.78 (m, 1H), 2.74 – 2.55 (m, 2H), 2.26 – 2.09 (m, 1H), 2.09 – 1.85 (m, 2H). **^{13}C{^{1}H} NMR** (151 MHz, $CDCl_3$) δ 141.2, 127.5, 127.4, 127.0, 80.6, 28.6, 25.4, 18.9. **HRMS** (*m/z*): $[M+H]^+$ calcd for $C_8H_{10}NO_2S^+$, 184.04268; found, 184.04274.

2.3 Synthesis and characterisation of nitroalkanes C-arylated with furan

2.3.1 General Procedure B for C-arylation of nitroalkanes with furan

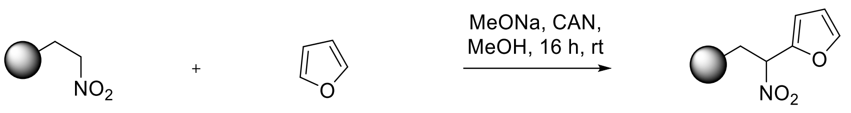

Scheme S16

To a solution of nitroalkane **1** (2.00 mmol, 1 eq.) in EtOH (10 mL) was added MeONa (1.3 eq., 2.6 mmol of 25% w/w solution of MeONa in MeOH), furan (**5h**) (20 eq., 40 mmol) and CAN (2.0 eq., 4.00 mmol) and the mixture was left to stir at room temperature (500 rpm). After 16 h, saturated solution of $Na_2S_2O_3$ (20 mL) and water (20 mL) were added and the mixture was extracted with EtOAc (2 × 20 mL). The combined organic phases were washed with brine (20 mL), dried (Na_2SO_4) and concentrated *in vacuo*. The crude mixture was purified by column chromatography.

2.3.2 2-(3-Methyl-1-nitrobutyl)furan (3aa)

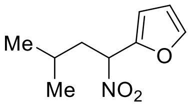

3aa

Figure S26

Nitro compound **3aa** was prepared according to the general procedure B using nitroalkane **1e** (2.000 mmol, 0.234 g), MeONa (1.3 eq., 2.600 mmol, 0.562 g of 25% w/w solution in MeOH), furan (**5h**) (20 eq., 40 mmol, 2.7 g, 2.9 mL), CAN (2.0 eq., 4.000 mmol, 2.183 g) and EtOH (10 mL). The crude product was purified by column chromatography (gradient eluent hexane to hexane:toluene 90:10 to 85:15) affording nitro compound **3aa** (198 mg, 53%) as a pale-yellow oil.

^{1}H NMR (600 MHz, $CDCl_3$) δ 7.45 (dd, *J* = 1.8, 0.8 Hz, 1H), 6.54 (d, *J* = 3.3 Hz, 1H), 6.41 (dd, *J* = 3.3, 1.8 Hz, 1H), 5.62 (dd, *J* = 8.4, 7.2 Hz, 1H), 2.35 (ddd, *J* = 13.9, 8.4, 6.6 Hz, 1H), 2.06 („dt“, *J* = 14.0, 7.4 Hz, 1H), 1.57 (m, 1H), 0.99 (d, *J* = 6.6 Hz, 3H), 0.96 (d, *J* = 6.7 Hz, 3H). **^{13}C{^{1}H} NMR** (151 MHz, $CDCl_3$) δ 147.6, 144.0, 111.2, 111.0, 82.9, 40.2, 25.3, 22.4, 22.1. **HRMS** (*m/z*): $[M+H]^+$ calcd for $C_9H_{14}NO_3^+$, 184.09682; found, 184.09575.

2.3.3 2-(1-Nitrobut-3-en-1-yl)furan (3ab)

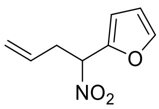

3ab

Figure S27

Nitro compound **3ab** was prepared according to the general procedure B using nitroalkane **1f** (2.00 mmol, 0.202 g), MeONa (1.3 eq., 2.60 mmol, 0.562 g of 25% w/w solution in MeOH), furan (**5h**) (20 eq., 40 mmol, 2.7 g, 2.9 mL), CAN (2.0 eq., 4.00 mmol, 2.19 g) and EtOH (10 mL). The crude product was purified by column chromatography (gradient eluent pentane to pentane:toluene 90:10 to 85:15) affording nitro compound **3ab** (0.144 g, 43%) as a colorless liquid.

^{1}H NMR (300 MHz, $CDCl_3$) δ 7.47 (dd, *J* = 1.9, 0.8 Hz, 1H), 6.56 (dt, *J* = 3.3, 0.7 Hz, 1H), 6.42 (ddd, *J* = 3.4, 1.9, 0.4 Hz, 1H), 5.84 – 5.63 (m, 1H), 5.57 (dd, *J* = 8.4, 6.9 Hz, 1H), 5.29 – 5.17 (m, 1H), 5.22 – 5.12 (m, 1H), 3.17 (m, 1H), 3.05 – 2.88 (m, 1H). **^{13}C{^{1}H} NMR** (151 MHz, $CDCl_3$) δ 146.9, 144.1, 131.0, 120.2, 111.6, 111.0, 83.7, 35.7. **HRMS** (*m/z*): [M+H]$^+$ calcd for $C_8H_{10}NO_3^+$, 168.06552; found, 168.06552.

2.3.4 2-(1-Nitropent-3-yn-1-yl)furan (3ac)

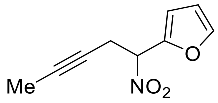

3ac

Figure S28

Nitro compound **3ac** was prepared according to the general procedure B using nitroalkane **1i** (2.00 mmol, 0.226 g), MeONa (1.3 eq., 2.60 mmol, 0.562 g of 25% w/w solution in MeOH), furan (**5h**) (20 eq., 40 mmol, 2.7 g, 2.9 mL), CAN (2.0 eq., 4.00 mmol, 2.19 g) and EtOH (10 mL). The crude product was purified by column chromatography (gradient eluent pentane to pentane:toluene 90:10 to 85:15 to 80:20) affording nitro compound **3ac** (0.150 g, 42%) as a pale-orange oil.

^{1}H NMR (300 MHz, $CDCl_3$) δ 7.46 (dd, *J* = 1.9, 0.8 Hz, 1H), 6.60 (dt, *J* = 3.4, 0.7 Hz, 1H), 6.42 (ddd, *J* = 3.4, 1.9, 0.4 Hz, 1H), 5.62 (ddt, *J* = 8.4, 6.5, 0.5 Hz, 1H), 3.30 (ddq, *J* = 16.9, 8.4, 2.5 Hz, 1H), 3.02 (ddq, *J* = 16.9, 6.5, 2.5 Hz, 1H), 1.74 (t, *J* = 2.5 Hz, 3H). **^{13}C{^{1}H} NMR** (151 MHz, $CDCl_3$) δ 146.3, 144.2, 111.6, 111.1, 82.9, 80.0, 71.9, 22.3, 3.6. **HRMS** (*m/z*): [M+H]$^+$ calcd for $C_9H_{10}NO_3^+$, 180.06552; found, 180.06556.

2.3.5 2-(1-Nitro-2-phenylethyl)furan (3ad)

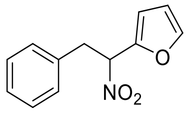

3ad

Figure S29

Nitro compound **3ad** was prepared according to the general procedure B using nitroalkane **1b** (0.400 mmol, 0.0600 g), MeONa (1.3 eq., 0.520 mmol, 0.112 g of 25% w/w solution in MeOH), furan (**5h**) (20 eq., 8.00 mmol, 0.545 g, 0.582 mL), CAN (2.0 eq., 0.800 mmol, 0.439 g) and EtOH (2 mL). The crude product was purified by column chromatography (gradient eluent pentane to pentane:toluene 90:10) affording nitro compound **3ad** (0.044 g, 51%) as a pale-yellow oil.

^{1}H NMR (300 MHz, $CDCl_3$) δ 7.48 (dd, *J* = 1.9, 0.8 Hz, 1H), 7.35 – 7.10 (m, 5H), 6.55 (dt, *J* = 3.4, 0.7 Hz, 1H), 6.40 (dd, *J* = 3.3, 1.9 Hz, 1H), 5.74 (dd, *J* = 8.4, 7.2 Hz, 1H), 3.72 (dd, *J* = 14.2, 8.4 Hz, 1H), 3.53 (dd, *J* = 14.1, 7.2 Hz, 1H). **^{13}C{^{1}H} NMR** (75 MHz, $CDCl_3$) δ 146.8, 144.1, 135.0, 129.0, 129.0, 127.7, 111.9, 111.1, 85.4, 37.7. **HRMS** (m/z): $[M+H]^+$ calcd for $C_{12}H_{12}NO_3^+$, 218.08117; found, 218.08113.

2.3.6 2-(2-(4-Fluorophenyl)-1-nitroethyl)furan (3ae)

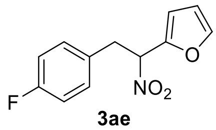

3ae

Figure S30

Nitro compound **3ae** was prepared according to the general procedure B using nitroalkane **1j** (2.00 mmol, 0.338 g), MeONa (1.3 eq., 2.60 mmol, 0.562 g of 25% w/w solution in MeOH), furan (**5h**) (20 eq., 40 mmol, 2.7 g, 2.9 mL), CAN (2.0 eq., 4.00 mmol, 2.19 g) and EtOH (10 mL). The crude product was purified by column chromatography (gradient eluent hexane to hexane:DCM 90:10 to 80:20) affording nitro compound **3ae** (0.240 g, 52%) as a pale-yellow oil.

^{1}H NMR (600 MHz, CDCl3) δ 7.49 (dd, *J* = 1.8, 0.8 Hz, 1H), 7.16 – 7.10 (m, 2H), 7.01 – 6.94 (m, 2H), 6.55 (dt, *J* = 3.3, 0.7 Hz, 1H), 6.41 (dd, *J* = 3.4, 1.8 Hz, 1H), 5.70 (dd, *J* = 8.5, 7.1 Hz, 1H), 3.70 (dd, *J* = 14.3, 8.4 Hz, 1H), 3.51 (dd, *J* = 14.3, 7.2 Hz, 1H). **^{13}C{^{1}H} NMR** (151 MHz, $CDCl_3$) δ 162.32 (d, *J* = 246.4 Hz), 146.51, 144.22, 130.65 (d, *J* = 8.1 Hz), 130.65 (d, *J* = 3.3 Hz), 115.93 (d, *J* = 21.5 Hz), 112.0, 111.2, 85.4, 36.9. **^{19}F NMR** (282 MHz, CD_3CD_2OD) δ -117.09 – -117.19 (m). **HRMS** (m/z): $[M+H]^+$ calcd for $C_{12}H_{11}FNO_3^+$, 236.07175; found, 236.07175.

2.3.7 2-(3-Methoxy-1-nitropropyl)furan (3af)

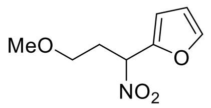

3af

Figure S31

Nitro compound **3af** was prepared according to the general procedure B using nitroalkane **1o** (1.00 mmol, 0.119 g), MeONa (1.3 eq., 1.30 mmol, 0.281 g of 25% w/w solution in MeOH), furan (**5h**) (20 eq., 20 mmol, 1.4 g, 1.5 mL), CAN (2.0 eq., 2.00 mmol, 1.096 g) and EtOH (5 mL). The crude product was purified by column chromatography (gradient eluent hexane to hexane:Et_2O 95:5 to 90:10) affording nitro compound **3af** (0.114 g, 62%) as an orange oil.

^{1}H NMR (300 MHz, $CDCl_3$) δ 7.46 (dd, *J* = 1.9, 0.8 Hz, 1H), 6.55 (ddd, *J* = 3.4, 0.8, 0.5 Hz, 1H), 6.41 (ddd, *J* = 3.3, 1.9, 0.4 Hz, 1H), 5.78 (dd, *J* = 8.3, 6.7 Hz, 1H), 3.52 – 3.27 (m, 2H), 3.31 (s, 3H), 2.73 (dddd, J = 14.5, 8.3, 6.6, 4.6 Hz, 1H), 2.42 (dtd, *J* = 14.5, 6.9, 4.7 Hz, 1H). **^{13}C{^{1}H} NMR** (151 MHz, $CDCl_3$) δ 147.1, 144.1, 111.5, 111.0, 81.1, 68.0, 59.0, 31.7. **HRMS** (*m/z*): $[M+H]^+$ calcd for $C_8H_{12}NO_4^+$, 186.07608; found, 186.07609.

2.3.8 Ethyl 4-(furan-2-yl)-4-nitrobutanoate (3ag)

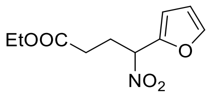

3ag

Figure S32

Nitro compound **3ag** was prepared according to the general procedure B using nitroalkane **1q** (2.00 mmol, 0.322 g), MeONa (1.4 eq., 2.80 mmol, 0.605 g of 25% w/w solution in MeOH), furan (**5h**) (20 eq., 40 mmol, 2.7 g, 2.9 mL), CAN (2.0 eq., 4.00 mmol, 2.193 g) and EtOH (10 mL). The crude product was purified by column chromatography (gradient eluent hexane to hexane:toluene 50:50 to toluene) affording nitro compound **3ag** (0.194 g, 43%) as a pale-yellow oil.

^{1}H NMR (300 MHz, $CDCl_3$) δ 7.46 (dd, *J* = 1.9, 0.8 Hz, 1H), 6.57 (ddd, *J* = 3.4, 0.9, 0.5 Hz, 1H), 6.47 – 6.37 (m, 1H), 5.74 – 5.63 (m, 1H), 4.15 (q, *J* = 7.1 Hz, 2H), 2.83 – 2.65 (m, 1H), 2.55 (dddd, J = 14.1, 7.9, 7.1, 6.5 Hz, 1H), 2.47 – 2.28 (m, 2H), 1.28 (t, *J* = 7.1 Hz, 3H). **^{13}C{^{1}H} NMR** (151 MHz, $CDCl_3$) δ 171.8, 146.7, 144.3, 111.8, 111.1, 82.9, 61.1, 30.2, 26.6, 14.3. **HRMS** (m/z): $[M+Na]^+$ calcd for $C_{10}H_{13}NO_5Na^+$, 250.06859; found, 250.06873.

2.3.9 *tert*-Butyl ((2R)-1-((2-((4-(furan-2-yl)-4-nitrobutyl)amino)-2-oxoethyl)amino)-1-oxopropan-2-yl)carbamate (3ah)

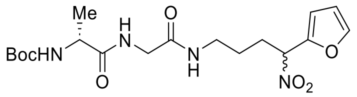

3ah

Figure S33

Nitro compound **3ah** was prepared according to the general procedure B using nitroalkane **1t** (0.200 mmol, 69.3 mg), MeONa (1.3 eq., 0.260 mmol, 56.2 mg of 25% w/w solution in MeOH), furan (**5h**) (20 eq., 4.0 mmol, 272 mg, 291 µL), CAN (2.0 eq., 0.400 mmol, 219 mg) and EtOH (2.0 mL). The crude product was purified by column chromatography (gradient eluent toluene to toluene:MeOH 90:10) affording nitro compound **3ah** (44 mg, 53%, d.r. ~50:50) as a pale-yellow oil.

Signals for both diastereomers reported as observed.
^{1}H NMR (600 MHz, $CDCl_3$) δ 7.46 – 7.42 (m, 1H), 7.01 (bs, 1H), 6.85 (bs, 1H), 6.55 (d, J = 3.3 Hz, 1H), 6.40 (dd, J = 3.4, 1.8 Hz, 1H), 5.57 („t“, J = 7.7 Hz, 1H), 5.09 (d, J = 5.4 Hz, 1H), 4.08 – 4.00 (m, 1H), 3.98 – 3.87 (m, 2H), 3.40 – 3.34 (m, 1H), 3.23 (bs, 1H), 2.49 – 2.40 (m, 1H), 2.28 – 2.18 (m, 1H), 1.58 (m, 2H), 1.42 (s, 9H), 1.36 (d, J = 7.1 Hz, 3H). **^{13}C{^{1}H} NMR** (151 MHz, $CDCl_3$) δ 173.4, 169.3, 156.3, 147.1, 144.1, 144.1, 111.6, 111.1, 83.8, 83.8, 80.9, 51.3, 43.3, 38.5, 28.7, 28.4, 26.0, 26.0, 17.7. **HRMS** (*m/z*): $[M+Na]^+$ calcd for $C_{18}H_{28}N_4NaO_7^+$, 435.18502; found, 435.18518.

2.3.10 (2*S*,3*S*,4*S*,5*R*,6*R*)-2-(3-(furan-2-yl)-3-nitropropoxy)-3,4,5-trimethoxy-6-(methoxymethyl)tetrahydro-2H-pyran (3ai)

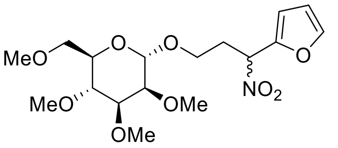

3ai

Figure S34

Nitro compound **3ai** was prepared according to the general procedure A using nitroalkane **1u** (0.20 mmol, 65 mg), MeONa (1.3 eq., 0.26 mmol, 56 mg of 25% w/w solution in MeOH), furan (**5h**) (20 eq., 4.00 mmol, 0.272 g, 0.291 mL), CAN (2.0 eq., 0.400 mmol, 0.219 g) and EtOH (2.0 mL). The crude product was purified by column chromatography (gradient eluent hexane:EtOAc 75:25 to 50:50 to EtOAc) affording impure nitro compound **3ai** as a yellow oil. This material was purified by preparative HPLC yielding nitro compound **3ai** (29 mg, 37%) as a colorless oil.

NMR data are reported as observed for both diastereomers.

^{1}H NMR (600 MHz, $CDCl_3$) δ 7.47 – 7.46 (m, 2H), 6.57 – 6.56 (m, 2H), 6.43 – 6.41 (m, 2H), 5.73 (dd, J = 8.4, 6.6 Hz, 1H), 5.69 (dd, J = 8.2, 6.6 Hz, 1H), 4.83 (d, J = 1.8 Hz, 1H), 4.79 (d, J = 1.8 Hz, 1H), 3.83 (ddd, J = 10.4, 6.7, 4.5 Hz, 1H), 3.76 (ddd, J = 10.5, 7.3, 4.4 Hz, 1H), 3.58 – 3.35 (m, 38H), 2.81 – 2.72 (m, 2H), 2.47 (m, 2H). **^{13}C{^{1}H} NMR** (151 MHz, $CDCl_3$) δ 147.1, 147.0, 144.2, 111.7, 111.5, 111.1, 111.1, 97.5, 97.4, 81.7, 81.5, 81.27, 81.25, 77.1, 77.0, 76.43, 76.40, 71.84, 71.82, 71.76, 71.72, 63.8, 63.5, 60.78, 60.72, 59.3, 59.16, 59.15, 57.97, 57.90, 31.6, 31.5. **HRMS** (*m/z*): $[M+Na]^+$ calcd for $C_{17}H_{27}NNaO_9^+$, 412.15780; found, 412.15796.

2.4 Regioselectivity determination

2.4.1 Independent synthesis and characterisation of 3-(1-nitro-2-phenylethyl)thiophene 3a′

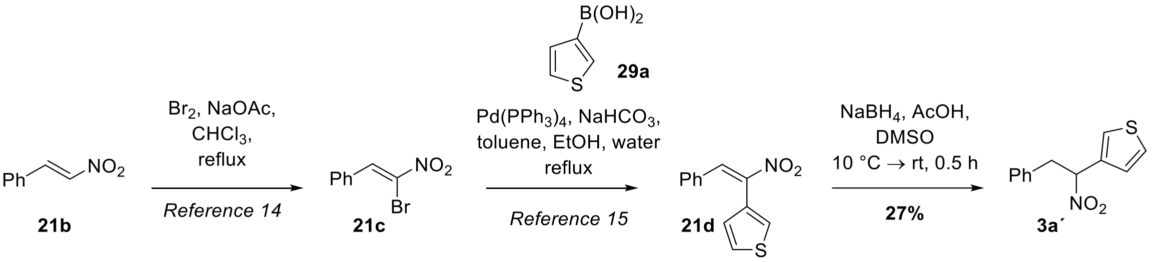

Scheme S17

2.4.1.1 3-(1-Nitro-2-phenylethyl)thiophene (3a′)

To a mixture of nitrostyrene **21d** (0.400 mmol, 0.093 g) and DMSO (2.0 mL) was added AcOH (2.5 eq., 1.0 mmol, 60 mg, 57 μL) under argon. The mixture was cooled to 10 °C (water bath) and $NaBH_4$ (1.5 eq., 0.60 mmol, 23 mg) was added portion wise . The resulting mixture was stirred at rt. After 20 min the mixture was diluted with water (10 mL) and extracted with Et_2O (2 × 10 mL). The combined organics were washed with brine (10 mL), dried (Na_2SO_4) and concentrated *in vacuo* yielding pale-orange oil (0.093 g). This residue was by column chromatography (gradient eluent hexane to hexane:Et_2O 97:3) affording impure nitro compound **3a′** (0.031 g) as a pale-yellow oil. This material was further purified by preparative HPLC yielding nitro compound **3a′** (0.025 g, 27%) as a pale-yellow oil.

^{1}H NMR (300 MHz, $CDCl_3$) δ 7.44 (ddd, *J* = 3.0, 1.4, 0.5 Hz, 1H), 7.37 (ddd, *J* = 5.2, 3.0, 0.4 Hz, 1H), 7.34 – 7.18 (m, 4H), 7.24 – 7.09 (m, 2H), 5.80 (ddd, *J* = 9.2, 5.9, 0.5 Hz, 1H), 3.74 (dd, *J* = 14.2, 9.2 Hz, 1H), 3.37 (dd, *J* = 14.2, 6.0 Hz, 1H). **^{13}C{^{1}H} NMR** (75 MHz, $CDCl_3$) δ 135.4, 135.0, 129.0, 129.0, 127.6, 127.2, 126.3, 125.6, 88.1, 40.5. **HRMS** (*m/z*): $[M+H]^+$ calcd for $C_{12}H_{12}NO_2S^+$, 234.05833; found, 234.05824.

2.4.2 Independent synthesis and characterisation of 2-methyl-3-(1-nitro-2-phenylethyl)thiophene (3b′)

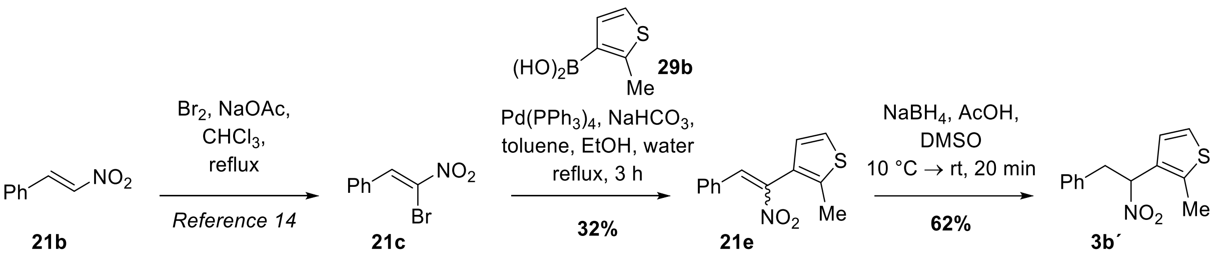

Scheme S18

2.4.2.1 2-Methyl-3-(1-nitro-2-phenylvinyl)thiophene (21e)

To a mixture of nitrostyrene **21c** (1.00 mmol, 0.228 g), boronic acid **29b** (1.5 eq., 1.50 mmol, 0.213 g) and $NaHCO_3$ (3 eq., 3.00 mmol, 0.252 g) was added toluene (9 mL), EtOH (1 mL) and water (1 mL). The resulting mixture was degassed and filled with argon. $Pd(PPh_3)_4$ (0.2 eq., 0.200 mmol, 0.231 g) was added, the mixture was degassed, filled with argon and stirred at reflux (HEAT SOURCE: metal heating block). After 3 h the mixture was cooled to rt, diluted with water (10 mL) and extracted with EtOAc (2 × 10 mL). The combined organics were washed with brine (10 mL), dried (Na_2SO_4) and concentrated *in vacuo* yielding a brown oil. This residue was purified by column chromatography (gradient eluent hexane to hexane:Et_2O 99:1) affording nitroalkene **21e** (78 mg, 32%) as a yellow solid.

^{1}H NMR (400 MHz, $CDCl_3$) δ 8.27 (s, 1H), 7.41 – 7.32 (m, 1H), 7.31 – 7.24 (m, 3H), 7.15 – 7.11 (m, 2H), 6.92 (d, J = 5.3 Hz, 1H) 2.16 (s, 3H). **^{13}C{^{1}H} NMR** (101 MHz, $CDCl_3$) δ 144.5, 141.3, 136.1, 131.5, 131.1, 130.8, 129.1, 128.8, 127.1, 123.7, 13.8. **HRMS** (*m/z*): $[M+H]^+$ calcd for $C_{13}H_{12}NO_2S^+$, 246.05833; found, 246.05842.

2.4.2.2 2-Methyl-3-(1-nitro-2-phenylethyl)thiophene (3b′)

To a mixture of nitrostyrene **21e** (0.18 mmol, 43 mg) and DMSO (0.92 mL) was added AcOH (2.5 eq., 0.45 mmol, 28 mg, 26 μL) under argon. The mixture was cooled to 10 °C (water bath) and $NaBH_4$ (1.5 eq., 0.27 mmol, 10 mg) was added portion wise. The resulting mixture was stirred at rt. After 20 min the mixture was diluted with water (10 mL) and extracted with Et_2O (2 × 10 mL). The combined organics were washed with brine (10 mL), dried (Na_2SO_4) and concentrated *in vacuo* yielding pale-orange oil (61 mg). This residue was purified by column chromatography (gradient eluent hexane to hexane:Et_2O 99:1) affording nitro compound **3b′** (28 mg , 62%) as a pale-yellow oil.

^{1}H NMR (400 MHz, $CDCl_3$) δ 7.32 – 7.19 (m, 4H), 7.15 – 7.07 (m, 3H), 5.73 (dd, J = 8.4, 6.7 Hz, 1H), 3.75 (dd, J = 14.0, 8.4 Hz, 1H), 3.30 (dd, J = 14.1, 6.7 Hz, 1H), 2.33 (s, 3H). **^{13}C{^{1}H} NMR** (101 MHz, $CDCl_3$) δ 139.6, 135.5, 130.4, 129.1, 128.9, 127.6, 125.8, 123.1, 85.9, 40.3, 12.9. **HRMS** (*m/z*): $[M+H]^+$ calcd for $C_{13}H_{14}NO_2S^+$, 248.07398; found, 248.07340.

2.4.3 Independent synthesis and characterisation of 2-methyl-4-(1-nitro-2-phenylethyl)thiophene (3b′′)

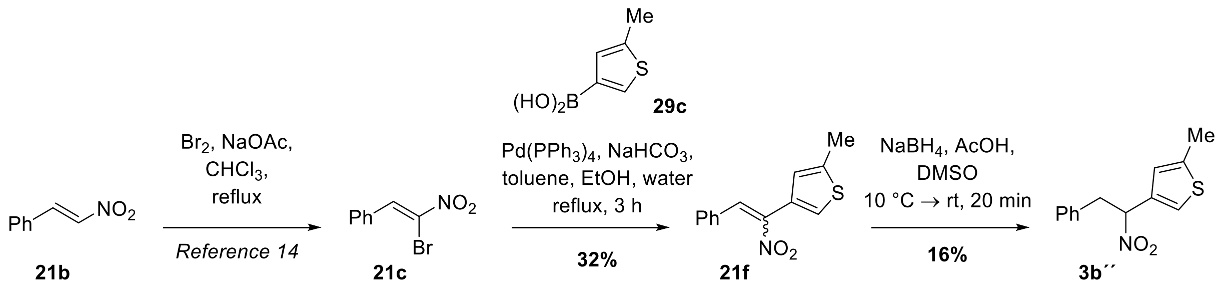

Scheme S19

2.4.3.1 2-Methyl-4-(1-nitro-2-phenylvinyl)thiophene (21f)

To a mixture of nitrostyrene **21c** (1.00 mmol, 0.228 g), boronic acid **29c** (1.5 eq., 1.50 mmol, 0.213 g) and $NaHCO_3$ (3 eq., 3.00 mmol, 0.252 g) was added toluene (9 mL), EtOH (1 mL) and water (1 mL). The resulting mixture was degassed and filled with argon. $Pd(PPh_3)_4$ (0.2 eq., 0.200 mmol, 0.231 g) was added, the mixture was degassed, filled with argon and stirred at reflux (HEAT SOURCE: metal heating block). After 3 h the mixture was cooled to rt, diluted with water (10 mL) and extracted with EtOAc (2 × 10 mL). The combined organics were washed with brine (10 mL), dried (Na_2SO_4) and concentrated *in vacuo* yielding a brown oil. This residue was purified by column chromatography (gradient eluent hexane to hexane:Et_2O 99:1) affording impure nitroalkene **21f** (90 mg) as a yellow solid. This material was purified by preparative HPLC yielding nitroalkene **21f** (40 mg, 32%) as a pale-yellow solid.

^{1}H NMR (600 MHz, $CDCl_3$) δ 8.15 (s, 1H), 7.38 – 7.32 (m, 1H), 7.31 – 7.26 (m, 2H), 7.23 – 7.15 (m, 2H), 7.13 (d, *J* = 1.4 Hz, 1H), 6.72 (quinted, *J* = 1.2 Hz, 1H), 2.54 (d, *J* = 1.1 Hz, 3H). **^{13}C{^{1}H} NMR** (151 MHz, $CDCl_3$) δ 145.4, 141.5, 135.2, 131.7, 131.1, 130.9, 129.7, 128.9, 126.6, 126.5, 15.5. **HRMS** (*m/z*): $[M+H]^+$ calcd for $C_{13}H_{12}NO_2S^+$, 246.05833; found, 246.05844.

2.4.3.2 2-Methyl-4-(1-nitro-2-phenylethyl)thiophene (3b´´)

To a mixture of nitrostyrene **21f** (0.20 mmol, 49 mg) and DMSO (1.0 mL) was added AcOH (2.5 eq., 0.50 mmol, 30 mg, 29 µL) under argon. The mixture was cooled to 10 °C (water bath) and $NaBH_4$ (1.5 eq., 0.30 mmol, 11 mg) was added portion wise. The resulting mixture was stirred at rt. After 20 min the mixture was diluted with water (10 mL) and extracted with Et_2O (2 × 10 mL). The combined organics were washed with brine (10 mL), dried (Na_2SO_4) and concentrated *in vacuo* yielding a pale-orange oil (51 mg). This residue was purified by column chromatography (gradient eluent hexane to hexane:Et_2O 99:1) affording nitro compound **3b´´** (28 mg , 16%) as a pale-yellow solid.

^{1}H NMR (300 MHz, $CDCl_3$) δ 7.35 – 7.10 (m, 6H), 6.96 – 6.88 (m, 1H), 5.69 (dd, *J* = 9.4, 5.8 Hz, 1H), 3.69 (dd, *J* = 14.3, 9.4 Hz, 1H), 3.33 (dd, *J* = 14.3, 5.8 Hz, 1H), 2.48 (d, *J* = 1.1 Hz, 3H). **^{13}C{^{1}H} NMR** (101 MHz, $CDCl_3$) δ 141.7, 135.5, 134.6, 129.0, 128.9, 127.6, 124.3, 123.4, 88.4, 40.3, 15.5. **HRMS** (*m/z*): $[M+H]^+$ calcd for $C_{13}H_{14}NO_2S^+$, 248.07398; found, 248.07383.

2.4.4 Comparison of ^{1}H NMR spectra of 3a´ and crude 3a

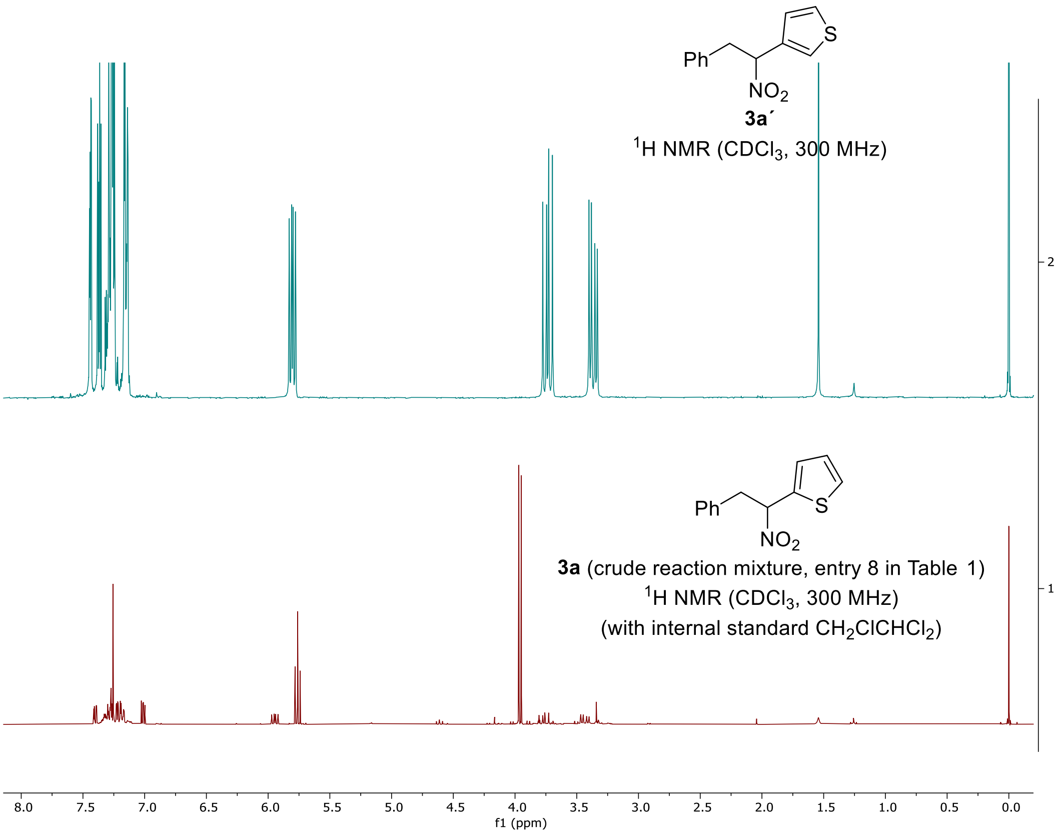

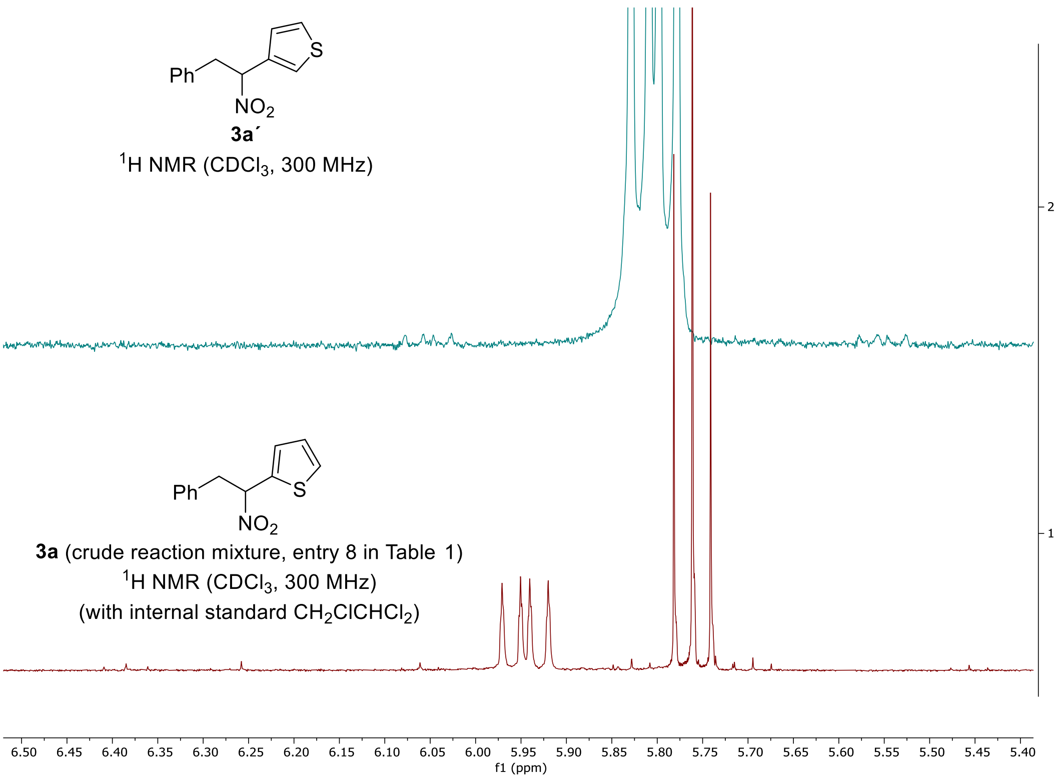

Figure S35

2.4.5 Comparison of ^{1}H NMR spectra of 3b´, 3b´´ and crude 3b

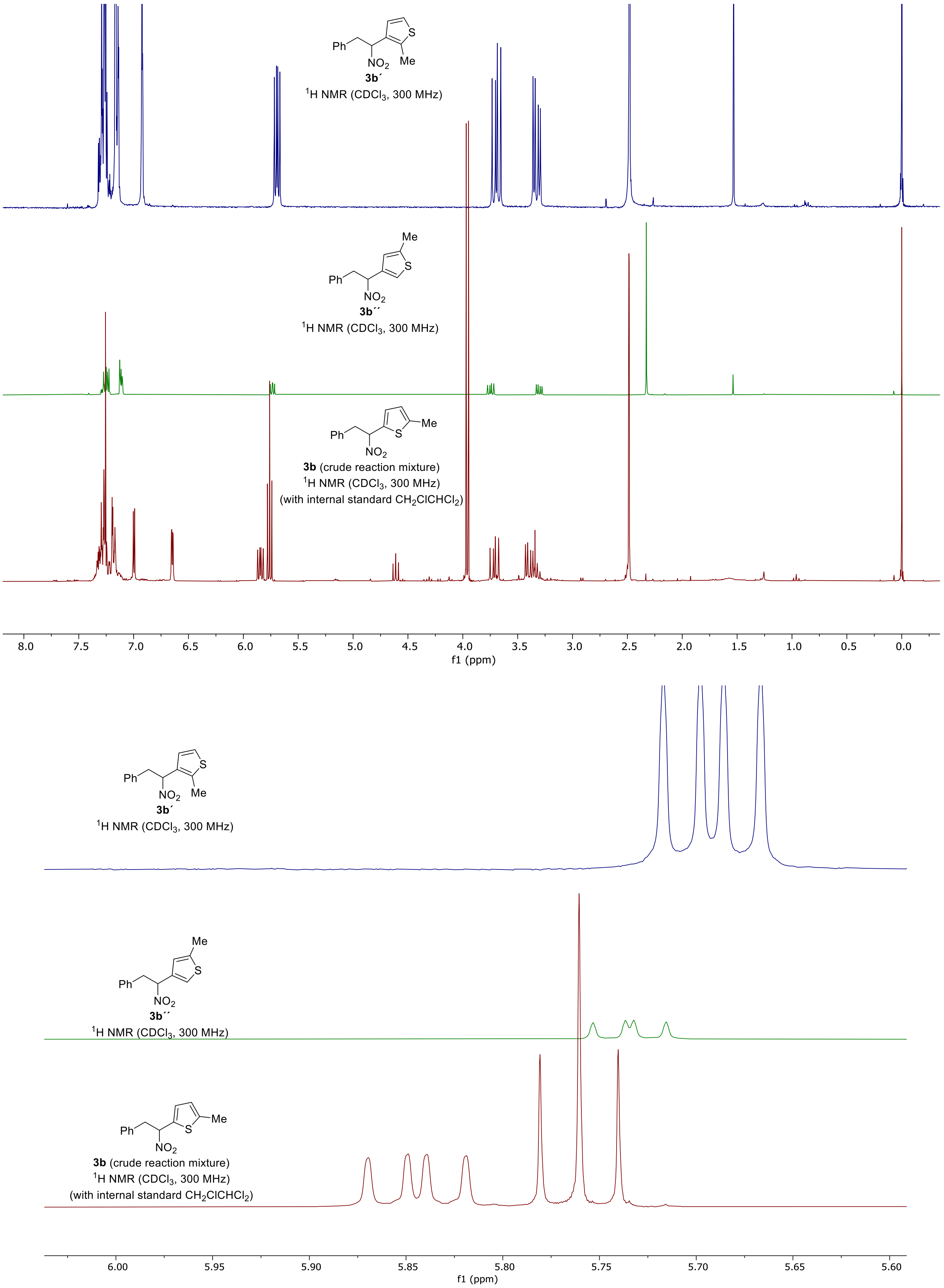

Figure S36

2.5 Derivatisation of C-arylated products

2.5.1 1-(5-Methylthiophen-2-yl)hexan-1-amine acetate (9a)

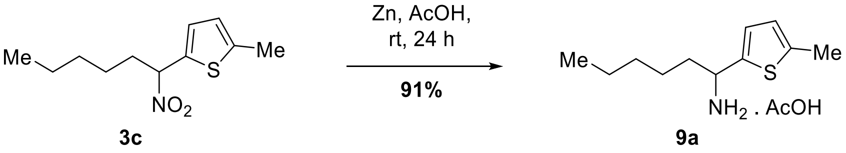

Scheme S20

To a mixture of nitroalkane **3c** (0.20 mmol, 45 mg) and acetic acid (4 mL) was over 20 min portion wise added zinc (4.00 mmol, 0.262 g) The resulting mixture was vigorously stirred (500 rpm) at rt. After 24 h the mixture was filtered throupgh a pad of Celite, the insoluble solid was washed with AcOH (2 times 4 mL) and the filtrate was concentrated *in vacuo*. This residue was purified by column chromatography (gradient eluent hexane:EtOAc to EtOAc to EtOAc:MeOH 90:10 yielding ammonium salt **9a** (47 mg, 91%) as a white solid.

^{1}H NMR (300 MHz, $CDCl_3$) δ 6.74 (d, *J* = 3.4 Hz, 1H), 6.57 (dq, *J* = 3.4, 1.1 Hz, 1H), 4.97 (s, 3H), 4.11 (t, *J* = 7.0 Hz, 1H), 2.44 (d, *J* = 1.1 Hz, 3H), 1.84 – 1.74 (m, 5H), 1.38 – 1.20 (m, 6H), 0.92 – 0.80 (m, 3H). **^{13}C{^{1}H} NMR** (75 MHz, $CDCl_3$) δ 177.4, 145.5, 138.8, 124.8, 124.2, 51.8, 38.6, 31.6, 26.0, 23.2, 22.6, 15.5, 14.1. **HRMS** (*m/z*): $[M+H]^+$ calcd for $C_{11}H_{20}NS^+$, 198.13110; found, 198.13111.

2.5.3 (*E*)-N-benzylidene-1-(5-methylthiophen-2-yl)hexan-1-amine oxide (9b)

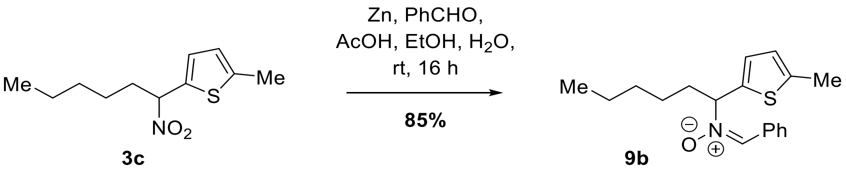

Scheme S21

A mixture of nitroalkane **3c** (2 eq., 0.400 mmol, 91 mg) and EtOH (3 mL) and water (0.15 mL) was cooled to 0-5 °C (ice-bath) under argon. Benzaldehyde (0.20 mmol, 21 mg, 21 µL) , zinc (3 eq., 0.60 mmol, 39 mg) and acetic acid (6 eq., 1.2 mmol, 72 mg, 69 µL) were added and the mixture (hetereogeneous mixture) was stirred at 0-5 °C under argon. After 16 h the mixture was filtered through a pad of Celite, the isoluble solid was washed with EtOH (2 times 4 mL) and the filtrate was concentrated *in vacuo*. The residue was purified by column chromatography (gradient eluent hexane to hexane:EtOAc 90:10 to 85:15) affording nitrone **9b** (51 mg, 85%) as as pale-yellow oil.

^{1}H NMR (300 MHz, $CDCl_3$) δ 8.30 – 8.18 (m, 2H), 7.49 (s, 1H), 7.46 – 7.34 (m, 3H), 6.92 (d, *J* = 3.5 Hz, 1H), 6.62 (dq, *J* = 3.4, 1.1 Hz, 1H), 5.08 (dd, *J* = 8.2, 6.5 Hz, 1H), 2.50 – 2.43 (m, 4H), 2.09 – 1.87 (m, 1H), 1.36 – 1.27 (m, 6H), 0.89 – 0.87 (m, 3H). **^{13}C{^{1}H} NMR (**101 MHz, $CDCl_3$) δ 141.1, 137.6, 132.8, 130.5, 130.3, 128.7, 128.4, 126.4, 124.3, 76.3, 34.6, 31.3, 26.1, 22.4, 15.3, 14.0. **HRMS** (*m/z*): $[M+Na]^+$ calcd for $C_{18}H_{23}NNaOS^+$, 324.13926; found, 324.13951.

2.5.2 1-(5-Methylthiophen-2-yl)hexan-1-one (9c)

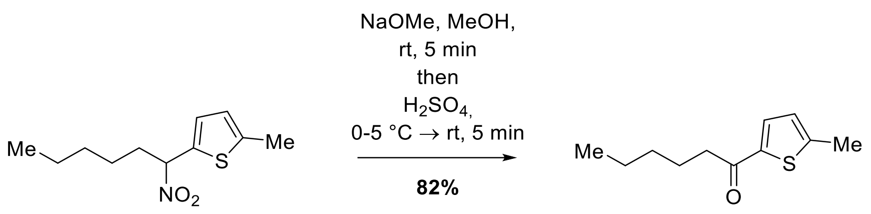

3c **9c**

Scheme S22

To a mixture of nitroalkane **3c** (0.20 mmol, 45 mg) and MeOH (4 mL) was added sodium methoxide (3 eq., 0.600 mmol, 130 mg of 25% w/w solution in MeOH) and the resulting mixture was stirred at rt. After 5 min the mixture (orange solution) was added drop wise over 10 min to sulfuric acid (6 mL of 12M aqueous solution) at 0-5 °C. The resulting mixture was warmed to rt and stirred at rt. After 5 min the mixture was diluted with water (24 mL) and extracted with Et_2O (2 times 15 mL). The combined organics were washed with brine (15 mL), dried (Na_2SO_4) and concentrated *in vacuo* yielding brown oil (0.051 g). This residue was purified by column chromatography (gradient eluent hexane to hexane:EtOAc 97:3) yielding ketone **9c** (0.032 g, 82%) as a pale-yellow oil.

^{1}H NMR (600 MHz, $CDCl_3$) δ 7.53 – 7.49 (m, 1H), 6.78 (dq, J = 3.7, 1.0 Hz, 1H), 2.84 – 2.79 (m, 2H), 2.52 (d, J = 1.0 Hz, 3H), 1.77 – 1.68 (m, 2H), 1.40 – 1.30 (m, 4H), 0.93 – 0.85 (m, 3H). **^{13}C{^{1}H} NMR** (151 MHz, $CDCl_3$) δ 193.4, 149.4, 142.5, 132.3, 126.8, 39.1, 31.7, 24.8, 22.6, 16.1, 14.1. **HRMS** (*m/z*): $[M+H]^+$ calcd for $C_{11}H_{17}OS^+$, 199.09946; found, 199.09932.

2.5.4 *N*-Ethylundecan-6-amine (9d)

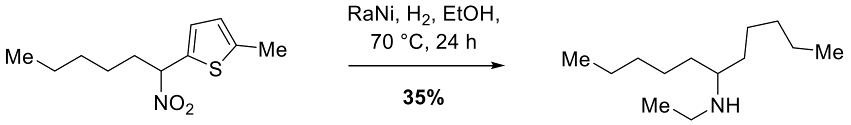

3c **9d**

Scheme S23

To a mixture of nitroalkane **3c** (0.800 mmol, 182 mg) and EtOH (16 mL) was added RaNi (20 eq, 16 mmol, 0.94 g). The resulting mixture was degassed, filled with hydrogen and stirred at 70 °C (HEAT SOURCE: metal heating block) in hydrogen atmosphere (balloon). After 24 h the mixture was cooled to rt, filtered through a pad of Celite, the insoluble solid was washed with EtOH (4 mL) and water (4 mL) and the filtrate was concentrated *in vacuo*. The residue was purified by column chromatography (gradient eluent EtOAc to EtOAc:MeOH 90:10 to 75:25) affording amine **9d** (56 mg, 35%) as as pale-yellow oil.

^{1}H NMR (300 MHz, CD_3OD) δ 3.19 – 3.03 (m, 3H), 1.80 – 1.59 (m, 4H), 1.47 – 1.28 (m, 16H), 0.94 (m, 6H). **^{13}C NMR{^{1}H}** (75 MHz, CD_3OD) δ 58.6, 42.1, 34.5, 33.3, 26.5, 23.7, 15.0, 14.4. **HRMS** (*m/z*): $[M+H]^+$ calcd for $C_{13}H_{30}N^+$, 200.23728; found, 200.23718.

2.6 Experiments to support the mechanistic proposal

2.6.1 Independent synthesis of the dimer 7

2.6.1.1 (2,3-Dinitrobutane-1,4-diyl)dibenzene (7)

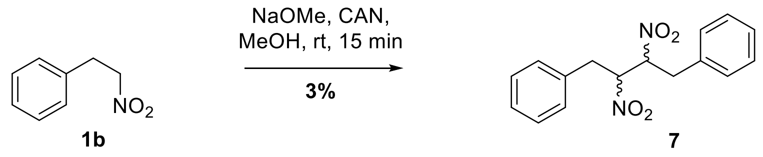

Scheme S24

To a solution of nitroalkane **1b** (1.99 mmol, 0.300 g) in MeOH (3.0 ml) were added MeONa (2.58 mmol, 558 mg of 25% w/w solution in MeOH) and CAN (4.37 mmol, 2.39 g) and the resulting mixture was stirred at room temperature. After 15 minutes, to the reaction mixture was added saturated solution of $Na_2S_2O_3$ (15 ml), then water (15 mL) and the mixture was extracted with EtOAc (2 × 20 mL). The combined organic layers were washed with brine (20 mL), dried (Na_2SO_4) and concentrated *in vacuo*. The crude product was purified by flash column chromatography (gradient eluent hexane:Et_2O 99:1 to 98:2) affording impure dimer **7**. This material was then purified by recrystallisation from *i*PrOH (2 ml). Desired dimer **7** (16 mg, 3%) was isolated as a pale-yellow solid.

^{1}H NMR (300 MHz, $CDCl_3$) δ 7.41 – 7.24 (m, 6H), 7.21 – 7.06 (m, 4H), 5.22 – 5.08 (m, 2H), 3.45 – 3.27 (m, 2H), 3.15 (dd, J = 14.6, 3.4 Hz, 2H). **^{13}C{^{1}H} NMR** (151 MHz, $CDCl_3$) δ 133.4, 129.3, 128.9, 128.3, 89.2, 36.8. **EA** Anal. Calcd for $C_{16}H_{16}N_2O_4$: C, 63.99; H, 5.37; N, 9.33. Found: C, 64.04; H, 5.09; N, 9.40.

2.6.2 Synthesis and characterisation of the non-aromatic intermediate (8a)

2.6.2.1 2-Ethoxy-5-(1-nitro-2-phenylethyl)-2,5-dihydrofuran (8a)

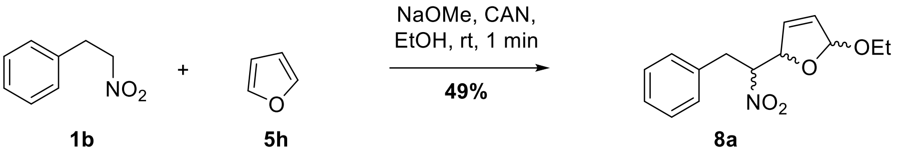

Scheme S25

Nitro compound **8a** was prepared according to general procedure B using nitroalkane **1b** (0.60 mmol, 91 mg), MeONa (0.720 mmol, 156 mg of 25% w/w solution in MeOH), furan (**5h**) (12 mmol, 0.87 ml), CAN (1.20 mmol, 658 g) and EtOH (3.0 ml). The reaction time was 1 min. The crude product was purified by flash column chromatography (gradient eluent hexane:EtOAc 95:5 to 75:25) and yielded nitro compound **8a** (77 mg, 49%, mixture of diastereomers) as a yellow oil.

^{1}H NMR (600 MHz, $CDCl_3$) δ 7.38 – 7.10 (m, 5H, all stereoisomers), 6.24 – 5.93 (m, 2H, all stereoisomers), 5.89 – 5.64 (m, 1H, all stereoisomers), 5.30 – 5.02 (m, 1H, all stereoisomers), 4.83 – 4.67 (m, 1H, all stereoisomers), 3.90 – 3.75 (m, 1H), 3.67 – 3.52 (m, 1H), 3.39 – 3.08 (m, 2H, all stereoisomers), 1.31 – 1.20 (m, 3H, all stereoisomers). **^{13}C{^{1}H} NMR** (151 MHz, $CDCl_3$) δ 135.7, 135.3, 135.2, 135.1, 130.73, 130.69, 130.67, 130.50, 130.45, 130.09, 130.01, 129.98, 129.05, 129.04, 129.03, 128.97, 128.95, 128.93, 128.92, 128.91, 127.68, 127.66, 127.63, 127.48, 108.85, 108.83, 108.6, 108.2, 93.6, 92.2, 91.77, 91.76, 85.0, 84.80, 84.80, 84.47, 64.43, 64.39, 63.73, 63.69, 36.0, 35.85, 35.80, 35.7, 15.6, 15.5, 15.44, 15.43. **HRMS** (*m/z*): $[M+Na]^+$ calcd for $C_{14}H_{17}NNaO_4S^+$, 286.10498; found, 286.10495.

2.6.3 Experiment in the presence of radical scavenger TEMPO

2.6.3.1 Synthesis and characterisation of 2,2,6,6-tetramethyl-1-(1-nitro-2-phenylethoxy)piperidine (31)

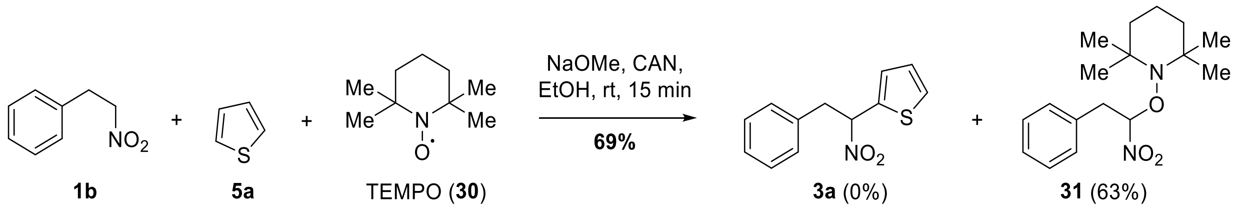

Scheme S26

To a mixture of nitro compound **1b** (0.40 mmol, 0.060 g) and MeOH (1 mL) was subsequently added MeONa (1.3 eq., 0.520 mmol, 0.112 g of 25% w/w solution in MeOH), thiophene (**5a**) (20 eq., 8.00 mmol, 0.673 g, 0.641 mL), TEMPO (**30**) (1 eq., 0.400 mmol, 0.062 g) and CAN (2.2 eq., 0.880 mmol, 0.482 g) and the resulting mixture was stirred at rt. After 15 minutes, to the reaction mixture was added saturated solution of $Na_2S_2O_3$ (8 ml), then water (8 mL) and the mixture was extracted with EtOAc (2 × 8 mL). The combined organic layers were washed with brine (8 mL), dried (Na_2SO_4) and concentrated *in vacuo*. The crude product was purified by flash column chromatography (gradient eluent hexane:EtOAc 95:5 to 75:25) and yielded nitro compound **31** (77 mg, 63%) as a yellow oil.

^{1}H NMR (300 MHz, $CDCl_3$) δ 7.41 – 7.20 (m, 3H), 7.25 – 7.10 (m, 2H), 5.80 (dd, *J* = 8.9, 4.8 Hz, 1H), 3.35 (dd, *J* = 14.2, 4.7 Hz, 1H), 3.23 (dd, *J* = 14.2, 8.9 Hz, 1H), 1.54 – 1.02 (m, 18H). **^{13}C NMR** (75 MHz, $CDCl_3$) δ 133.1, 129.5, 129.0, 127.9, 117.6, 61.8, 60.0, 40.5, 40.3, 39.0, 33.2, 32.0, 20.5, 20.2, 17.0. **HRMS** (m/z): $[M+H]^+$ calcd for $C_{17}H_{26}N_2O_3^+$, 307.20162; found, 307.20197.

3. Theoretical mechanistic investigation

3.1 Computational details

Density Functional Theory (DFT) was used throughout the study with the ωB97X-D4 functional[16] – a modified version of ωB97X-V[17] with D4 dispersion correction – together with the triple-zeta quality basis set def2-TZVPP.[18] The ωB97X-D4 functional has demonstrated strong performance in recent benchmark studies concerning reaction kinetics and thermodynamics.

Solvent effects were included via implicit solvation using the SMD model.[19] Ethanol was used as the solvent for reactions involving the furan substrate, while methanol was used for thiophene-based reactions, in accordance with the experimental setup. The reaction products and intermediates have multiple possible conformations.

Given the presence of multiple conformers for the reaction products and intermediates, the Global Optimizer Algorithm (GOAT)[20] was used to identify the global minimum. Due to the time and computational demands of this process, a two-step approach was applied. First, a tight-binding method was used to identify the global minimum and a set of low-energy structures.[21] These were subsequently re-optimized at the ωB97X-D4/def2-TZVPP/SMD(alcohol) level of theory and global minimum was selected from the optimized structures. For the final reaction step, explicit solvent molecules were necessary to model interactions with the leaving groups (Figures S3.3 and S3.7). Initial structures were generated by adding more than 100 implicit solvent molecules using stochastic SOLVATOR algorithm. Solvent molecules distant from the reactive sites were then removed, and the structure was re-optimized.

For all located minima, vibrational frequency (Hessian) calculations were performed. Transition state searches were initiated from nearby structures obtained by scanning a key reaction coordinate. All transition states were confirmed by the presence of a single imaginary frequency.

All calculations were performed using the ORCA software package, version 6.0.0,[22] with defgrid2 integration grid and tight optimization criteria.

In all energy calculations the value of 1.61 V was used as the standard reduction potential for CAN (v.s. SHE).[23]

3.2 Computational analysis

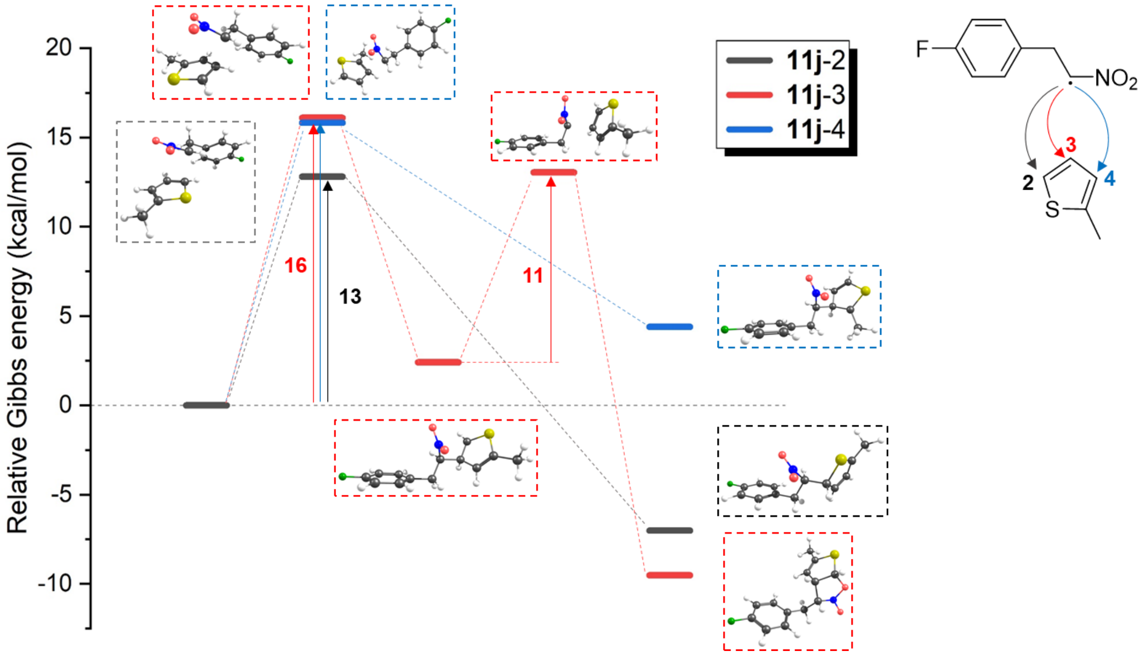

Figure S37

Theoretical Gibbs energy profile (in kcal/mol) of radical addition of **10j** to different positions on **5b** leading towards three regioisomers **11j**, which is a critical step for the regioselectivity of the whole reaction cascade. Note that the cyclized regioisomer **11j**-3 is formed in two steps, the first one corresponding to the formation of an open form, which is followed by the cyclization. All values were obtained at the ωB97X-D4/def2-TZVPP/SMD(methanol) level of theory.

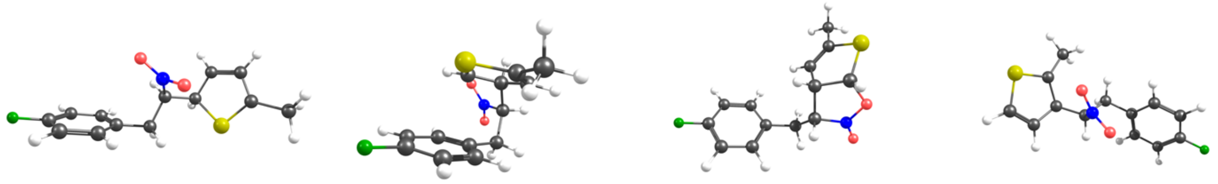

12j-2 (0.0) | **12j**-3 (open) (10.9) | **12j**-3 (cyclic) (14.0) | **12j**-4 (9.5)

Figure S38

Regioisomers of **12j** and their relative Gibbs energies (in kcal/mol) obtained at the ωB97X-D4/def2-TZVPP/SMD(methanol) level of theory.

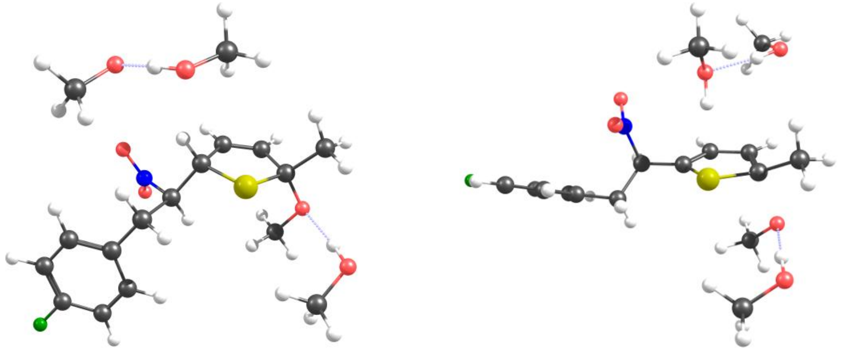

13j (0.0) | **13j**-TS3 (19.0)

Figure S39

Optimized structures of **13j** and **13j**-TS involving assisting explicit solvent molecules with their relative Gibbs energies obtained at the ωB97X-D4/def2-TZVPP/SMD(methanol) level of theory.

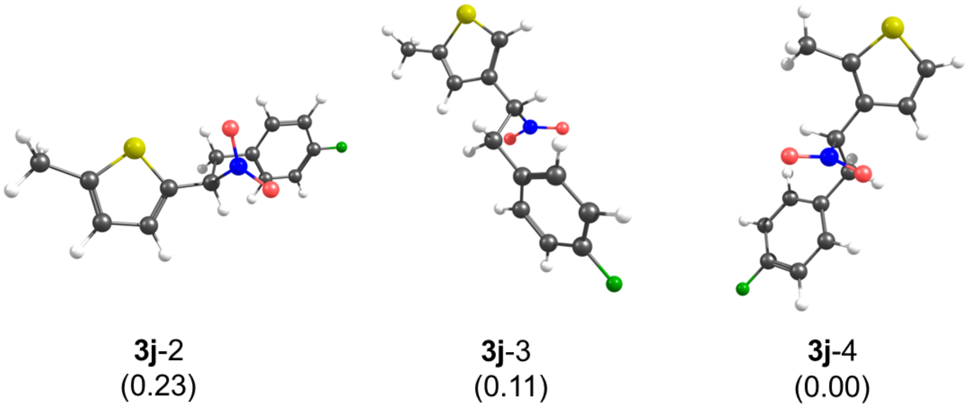

Figure S40

Regioisomers of **3j** and their relative Gibbs energies (in kcal/mol) obtained at the ωB97X-D4/def2-TZVPP/SMD(methanol) level of theory.

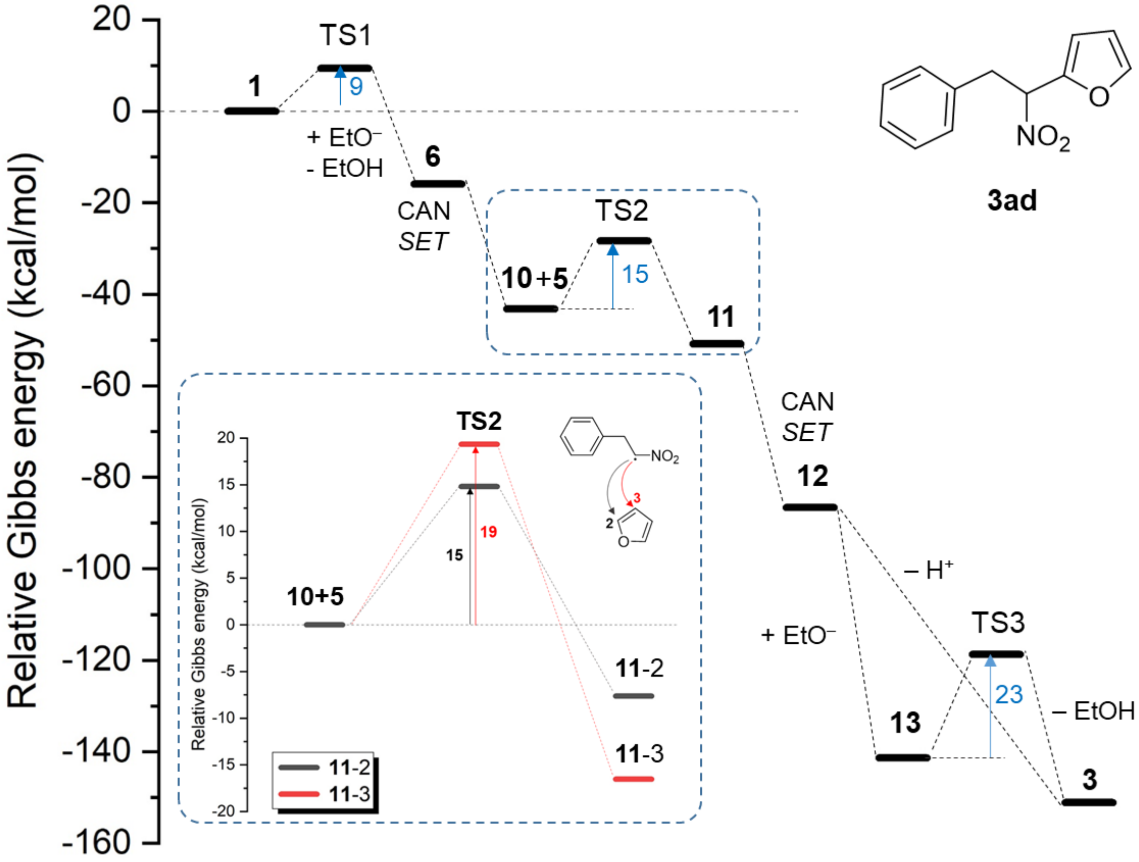

Figure S41

Theoretical Gibbs energy profile (in kcal/mol) of the formation of **3ad** from **1b** and **5d** following the mechanism shown in Scheme 5. Inset: Comparison of activation barriers of the formation of regioisomers **11ad** via radical addition of **10b** to different positions on **5d**, which is a critical step for the regioselectivity of the whole reaction cascade. All values were obtained at the ωB97X-D4/def2-TZVPP/SMD(ethanol) level of theory.

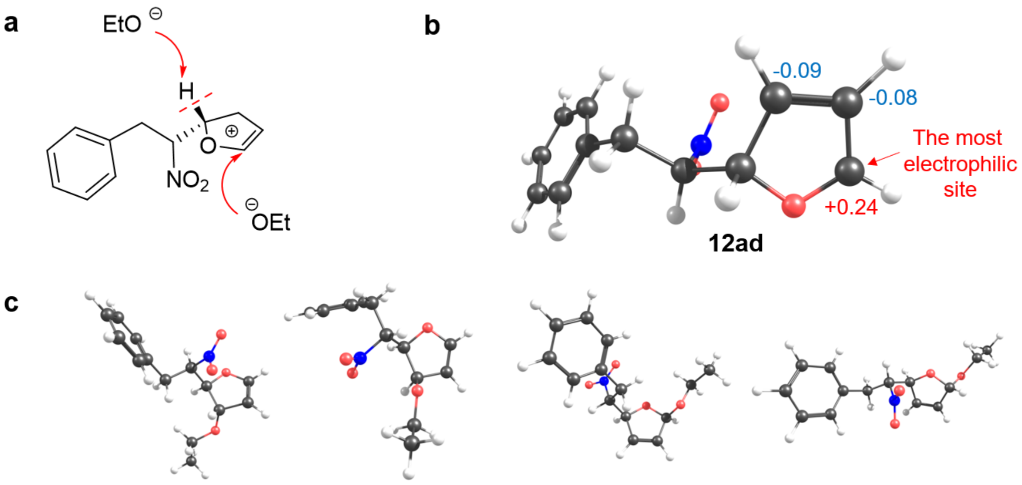

2,3-*anti*-**12ad**	2,3-*syn*-**12ad**	2,5-*syn*-**12ad**	2,5-*anti*-**12ad**
(5.91)	(5.75)	(0.06)	(0.00)

Figure S42

(a) Two alternative attacks of a methoxide anion on cationic intermediate **12ad** involving either barrierless deprotonation from the position #2 of the heteroaromatic ring or the formation of adduct **13ad**. (b) The optimized structure of **12ad** with Mulliken atomic charges on relevant carbons. (c) Isomers of **13ad** and their relative Gibbs energies (in kcal/mol). All values were obtained at the ωB97X-D4/def2-TZVPP/SMD(ethanol) level of theory.

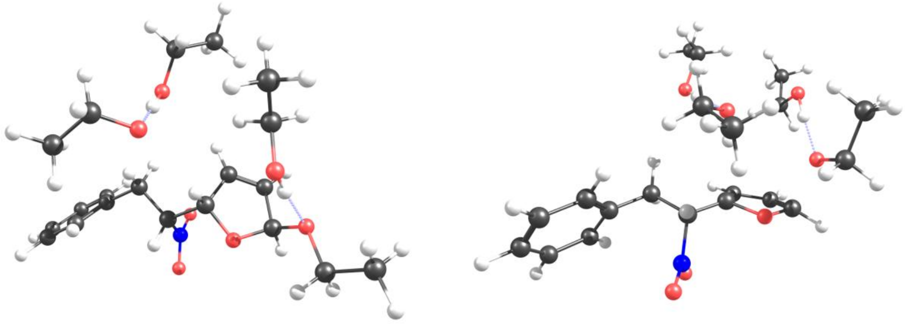

**13ad**	**13ad**-TS3
(0.0)	(23.0)

Figure S43

Optimized structures of **13ad** and **13ad**-TS involving assisting explicit solvent molecules with their relative Gibbs energies (in kcal/mol) obtained at the ωB97X-D4/def2-TZVPP/SMD(ethanol) level of theory.

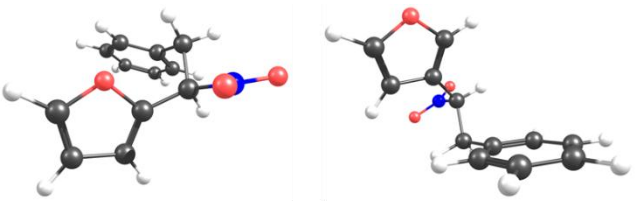

**3ad**-2	**3ad**-3
(0.00)	(0.67)

Figure S44

Regioisomers of **3ad** and their relative Gibbs energies (in kcal/mol) obtained at the ωB97X-D4/def2-TZVPP/SMD(ethanol) level of theory.

4. NMR spectra

4.1 NMR spectra of 1g

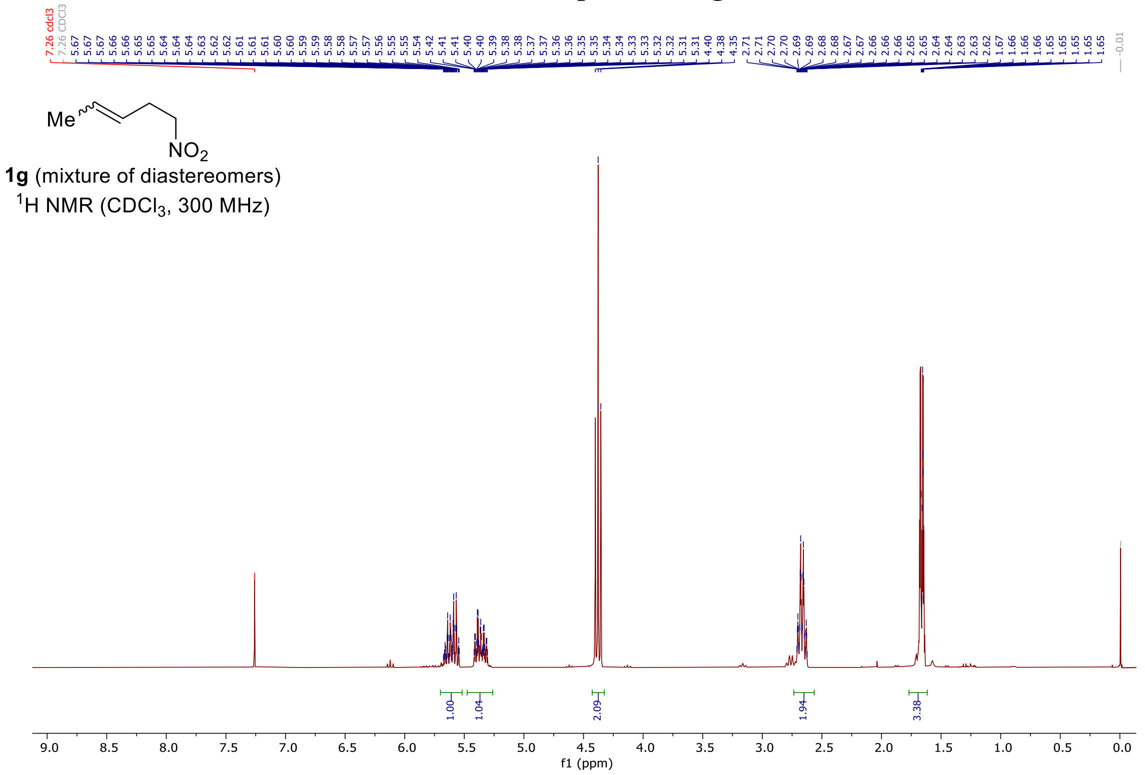

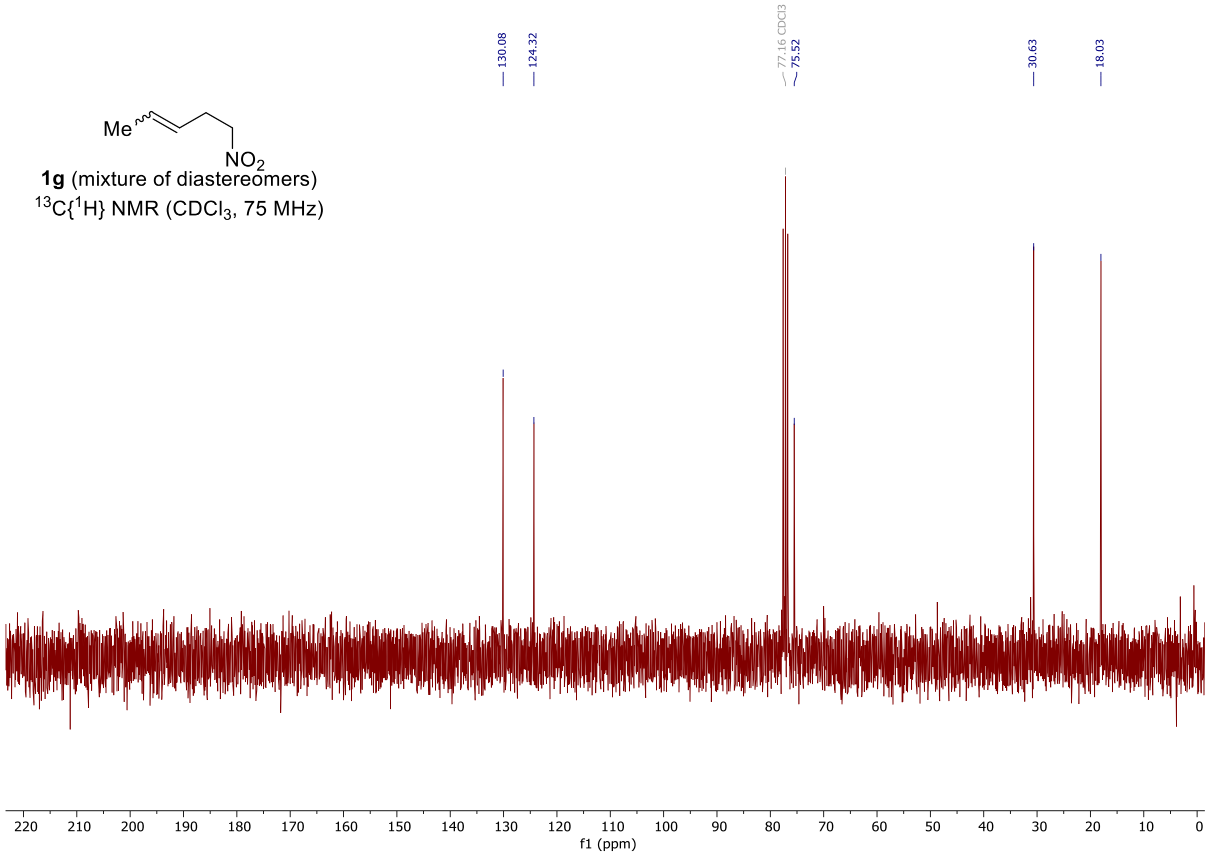

Figure S45

4.2 NMR spectra of 1l

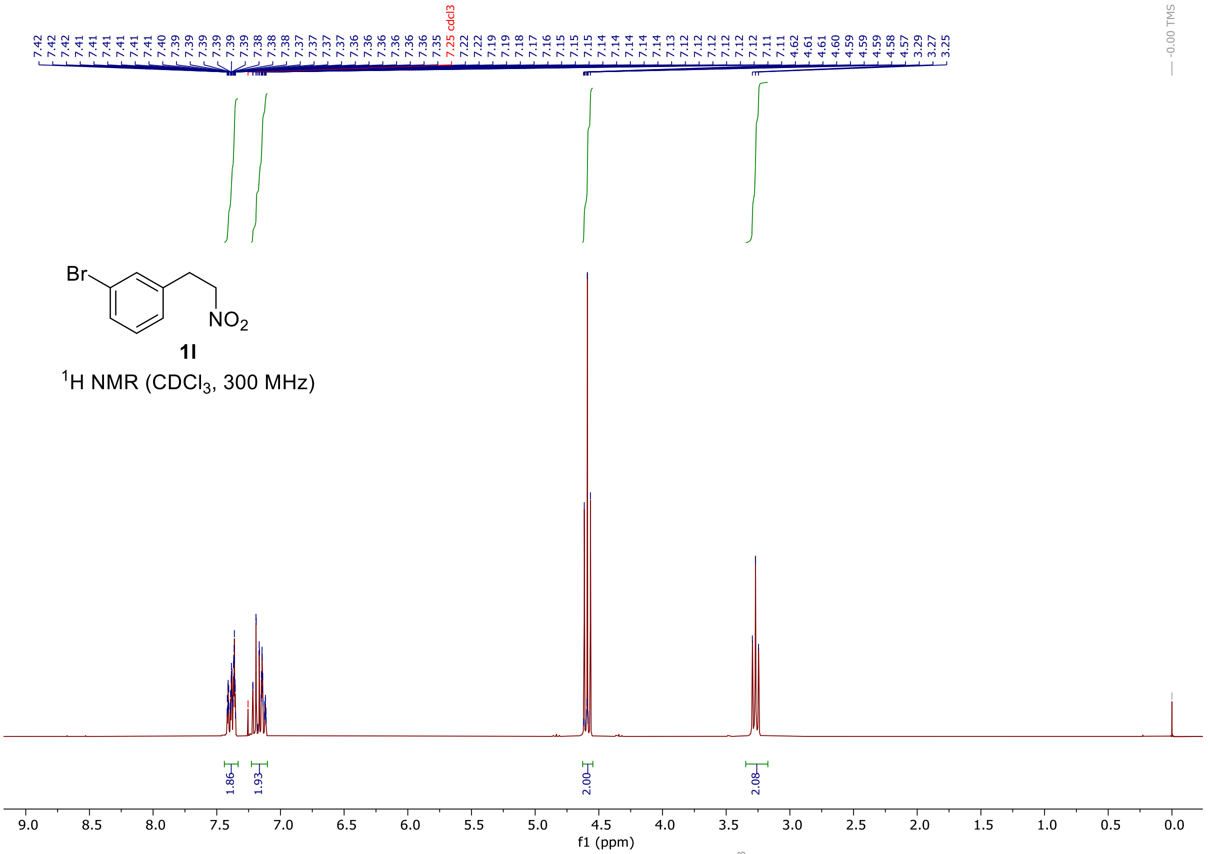

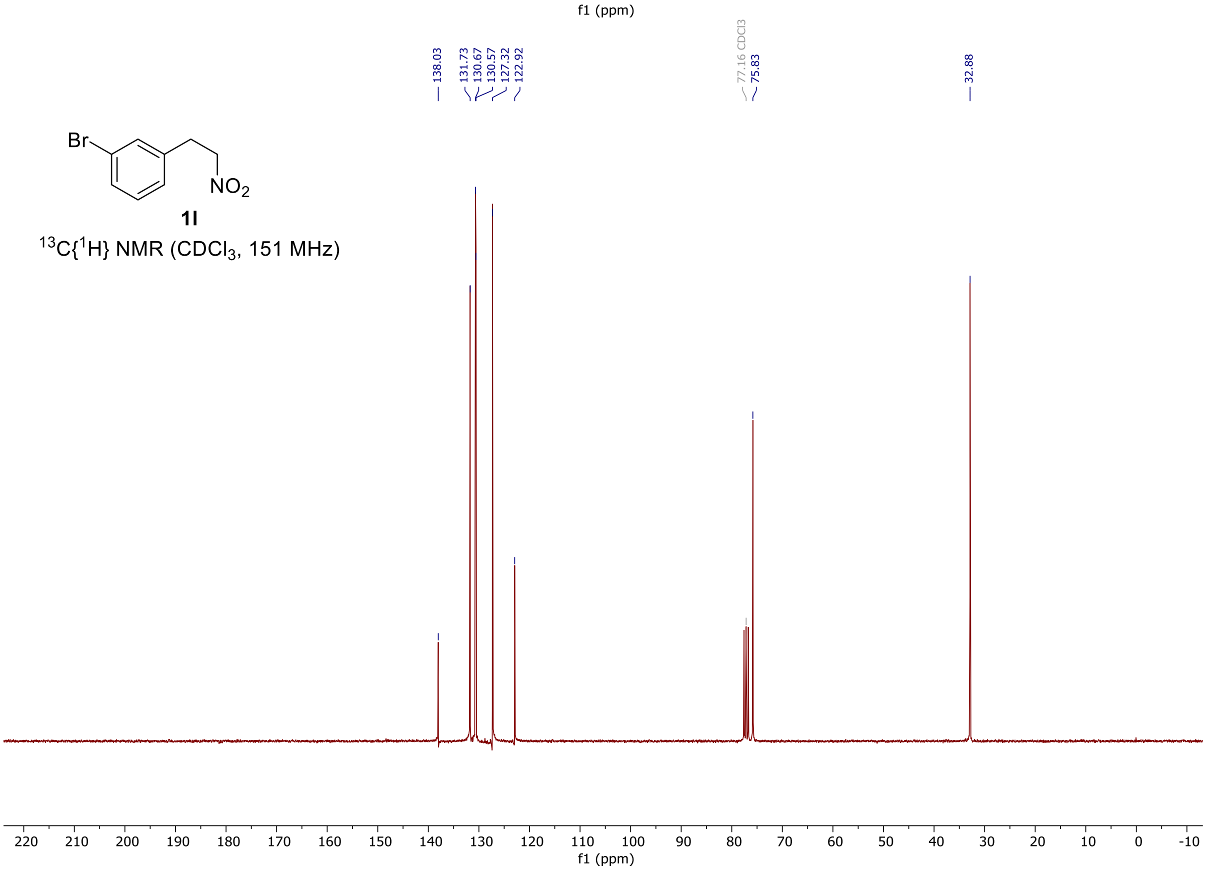

Figure S46

4.3 NMR spectra of 1n

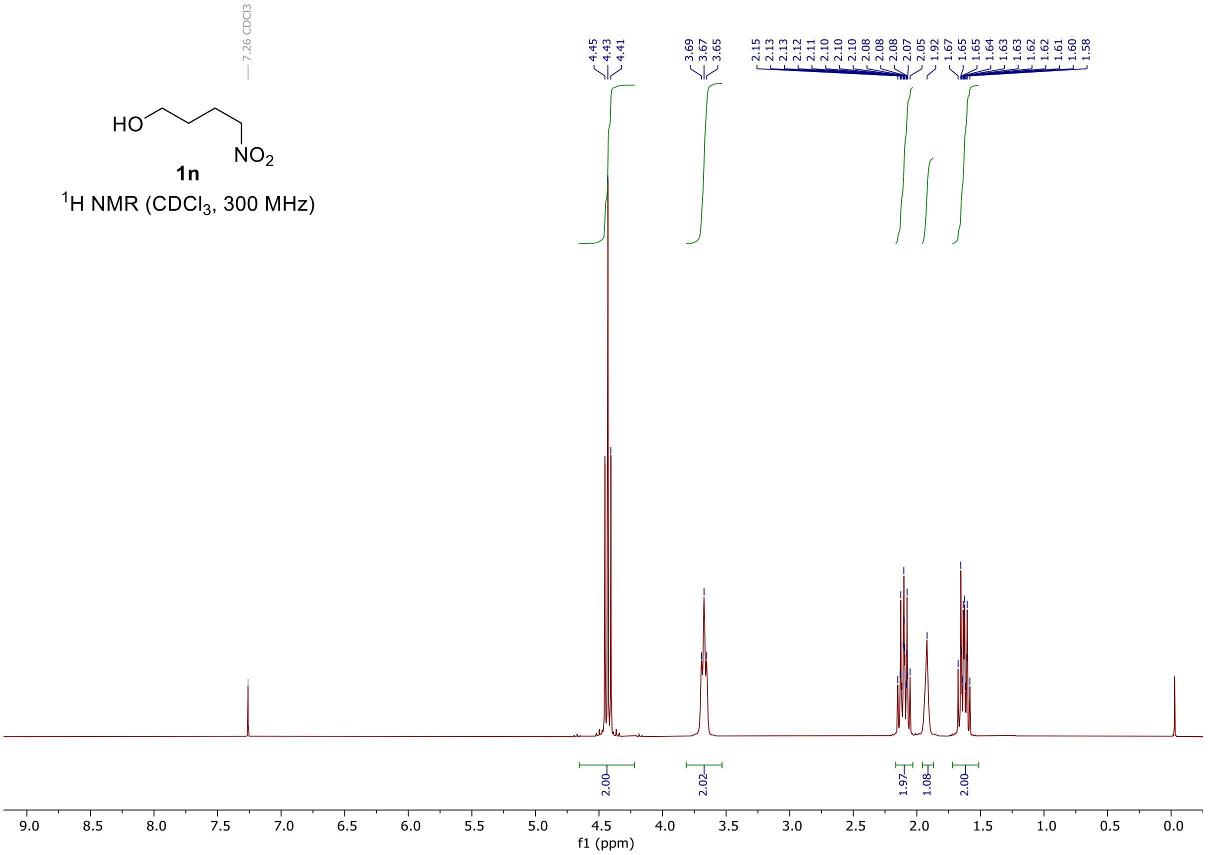

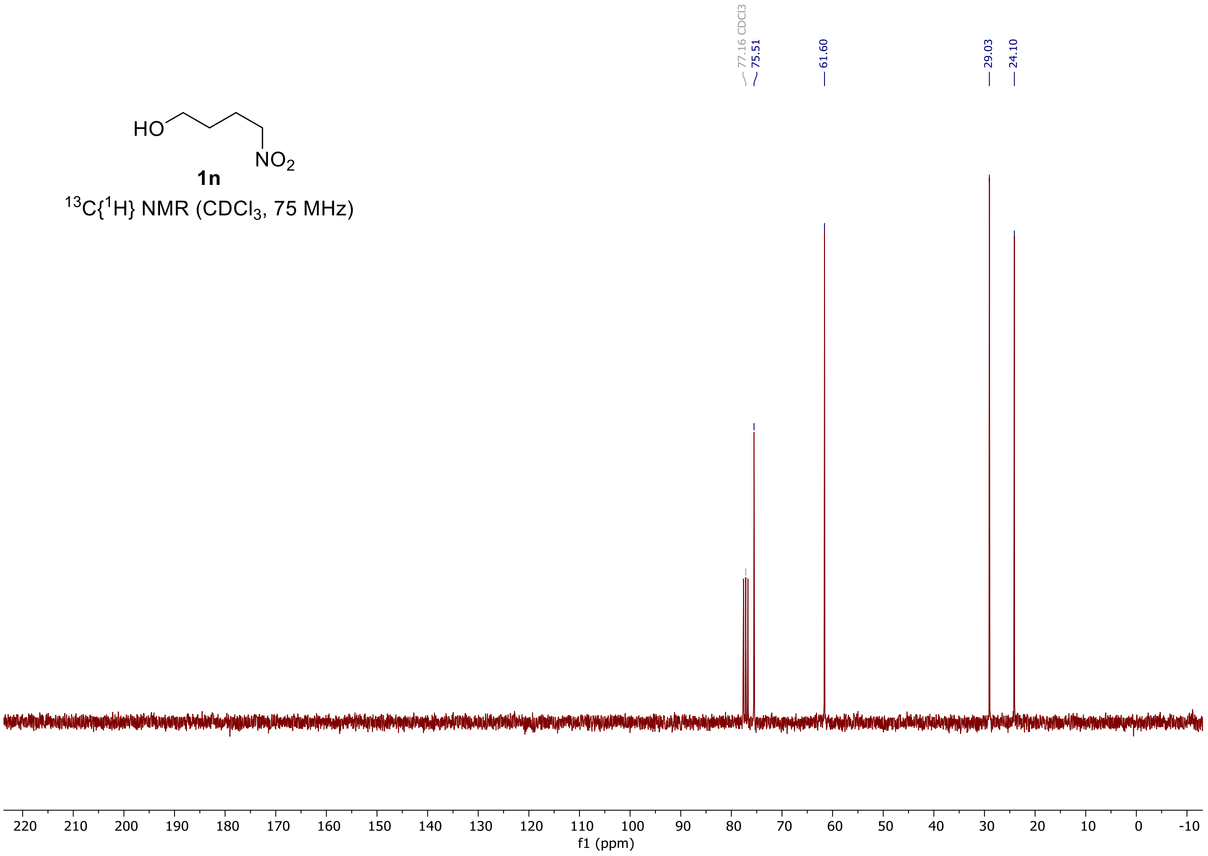

Figure S47

4.4 NMR spectra of 1o

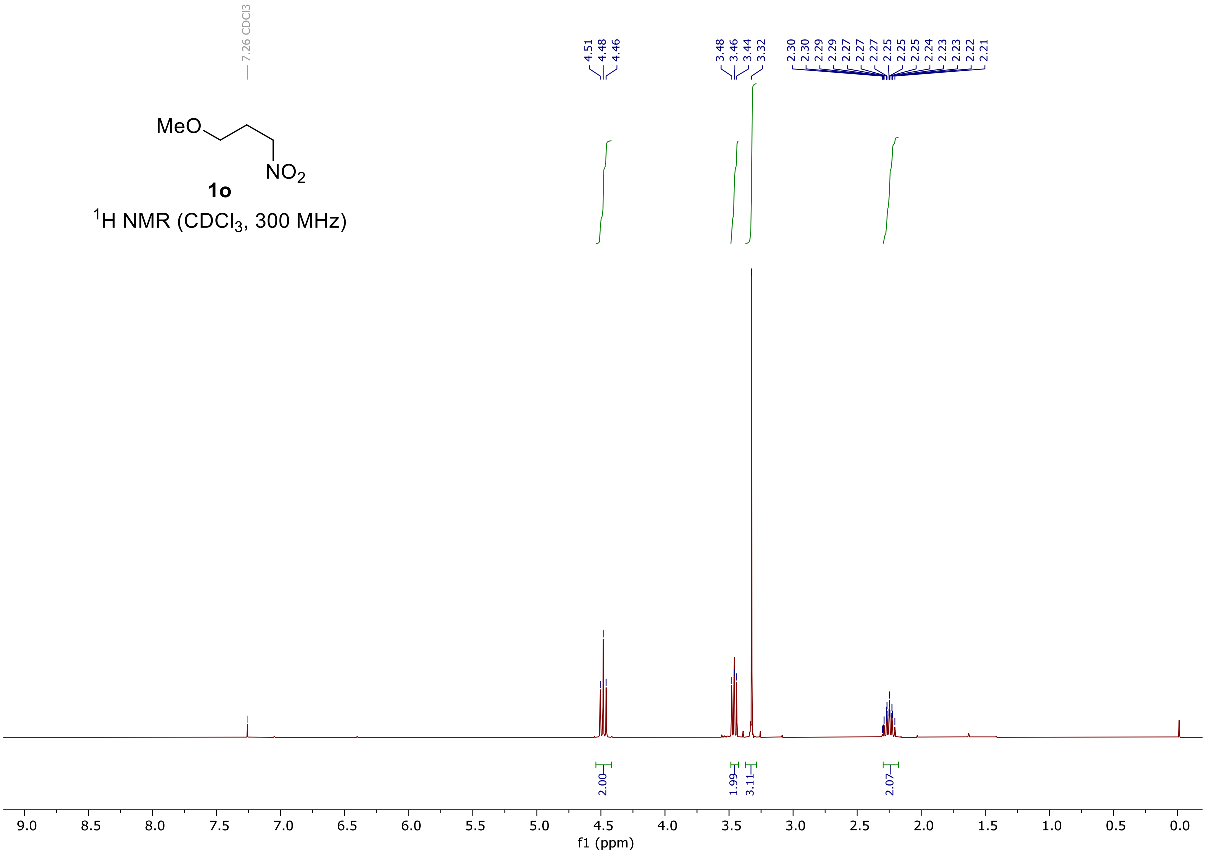

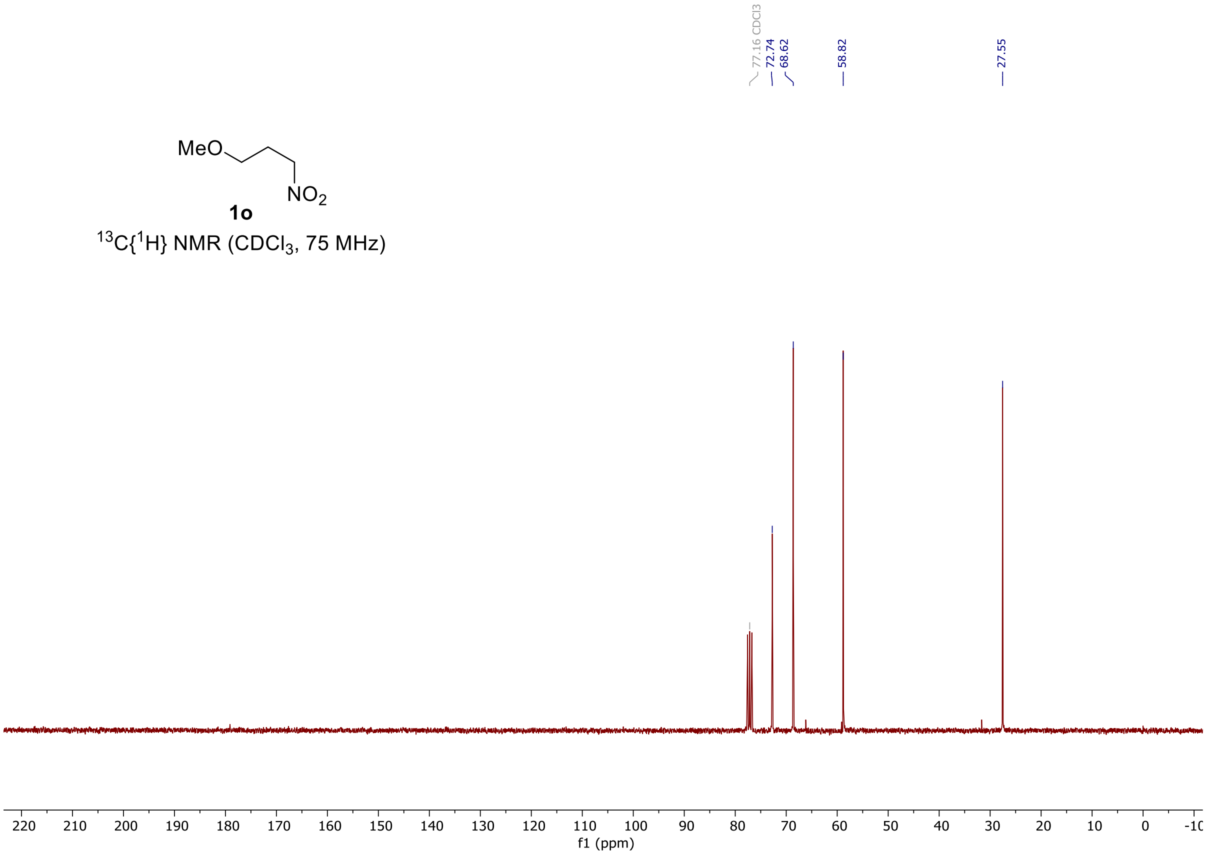

Figure S48

4.5 NMR spectra of 1p

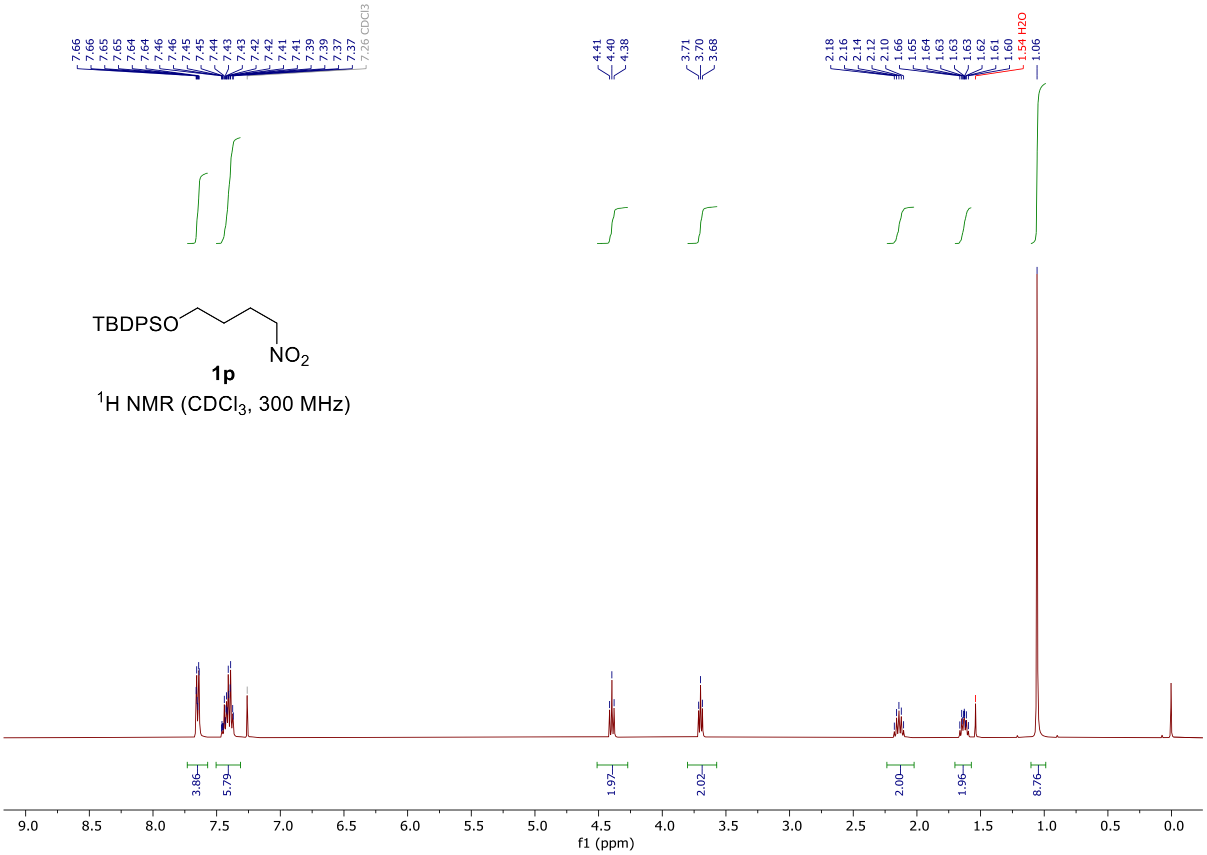

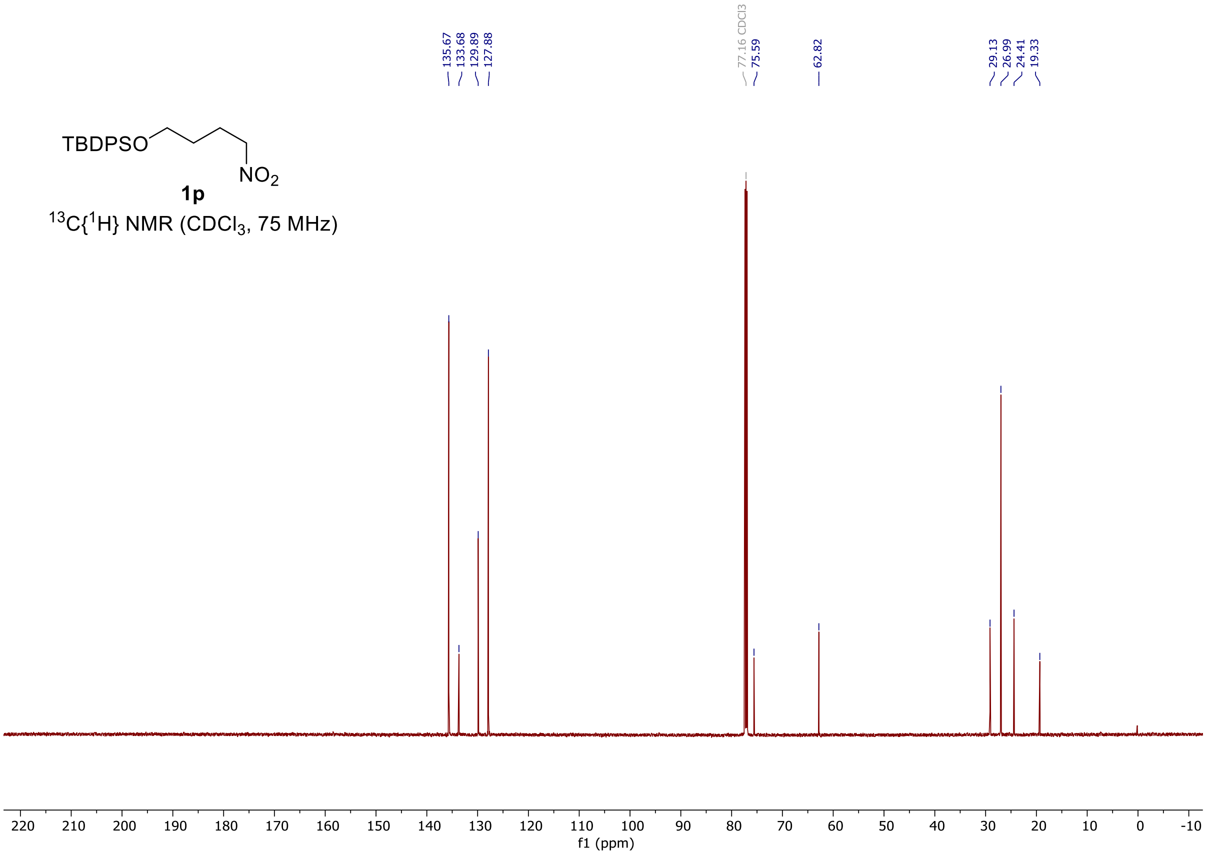

Figure S49

4.6 NMR spectra of 1q

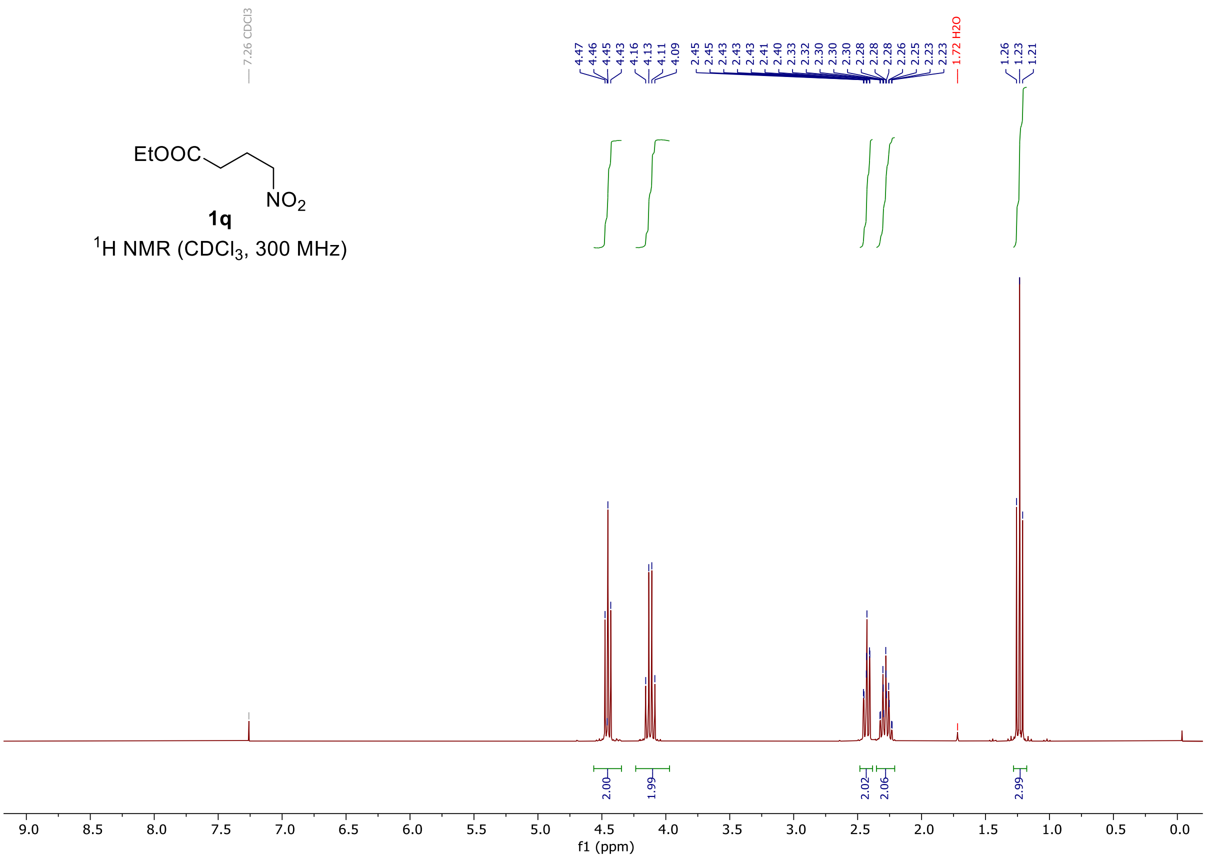

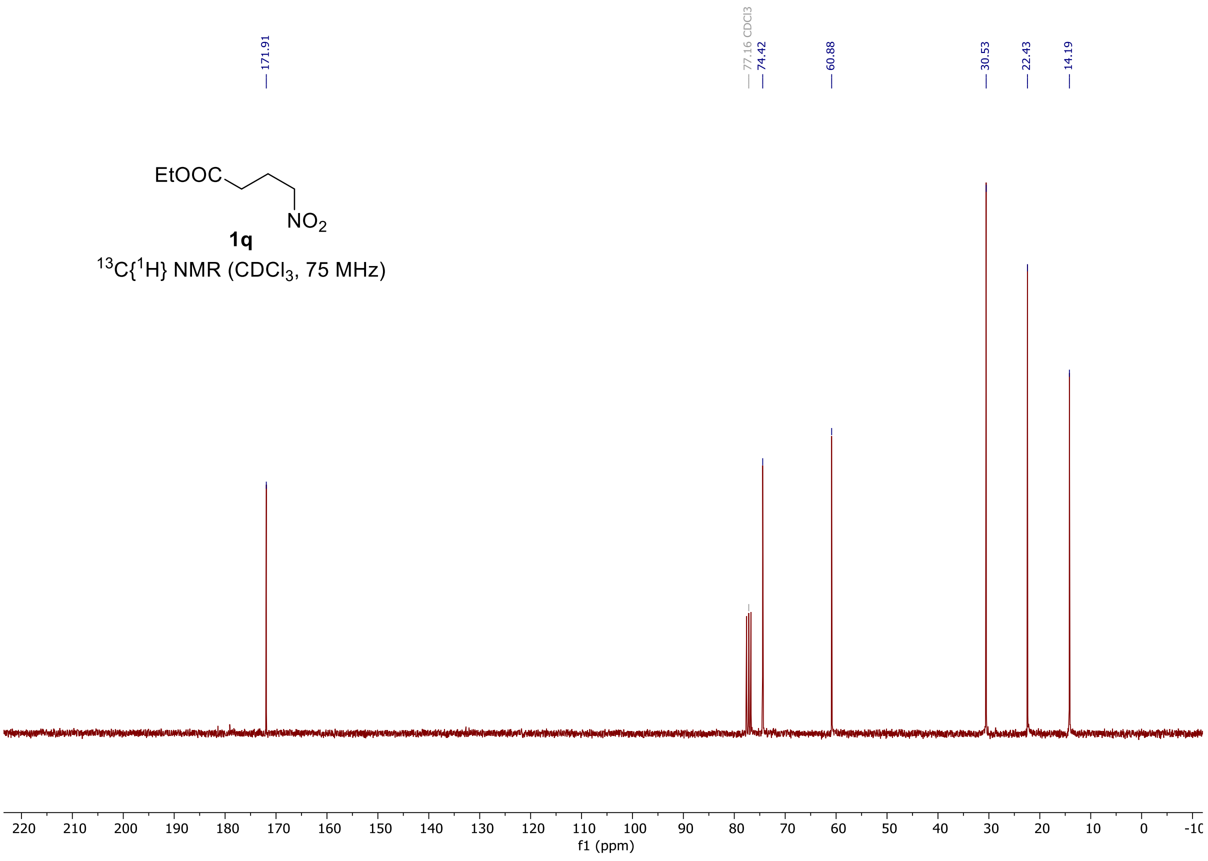

Figure S50

4.7 NMR spectra of 24

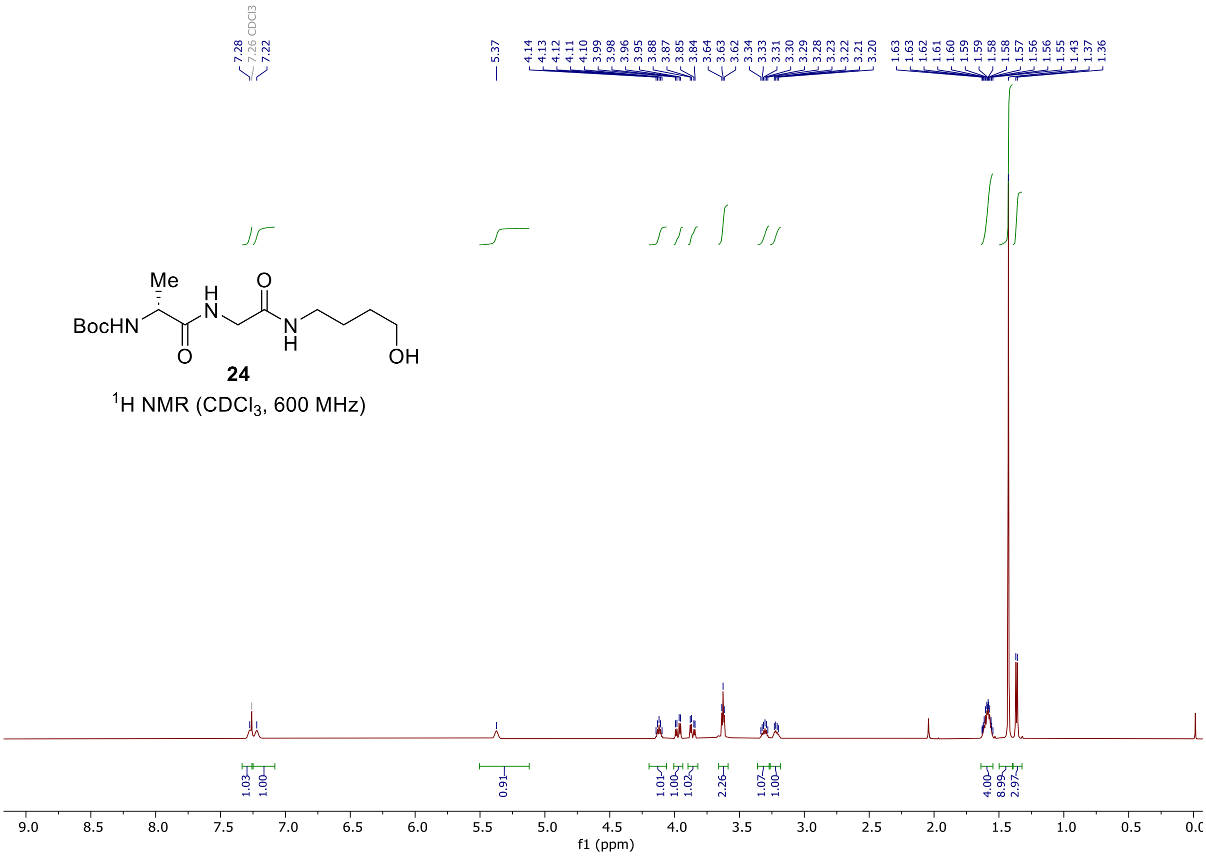

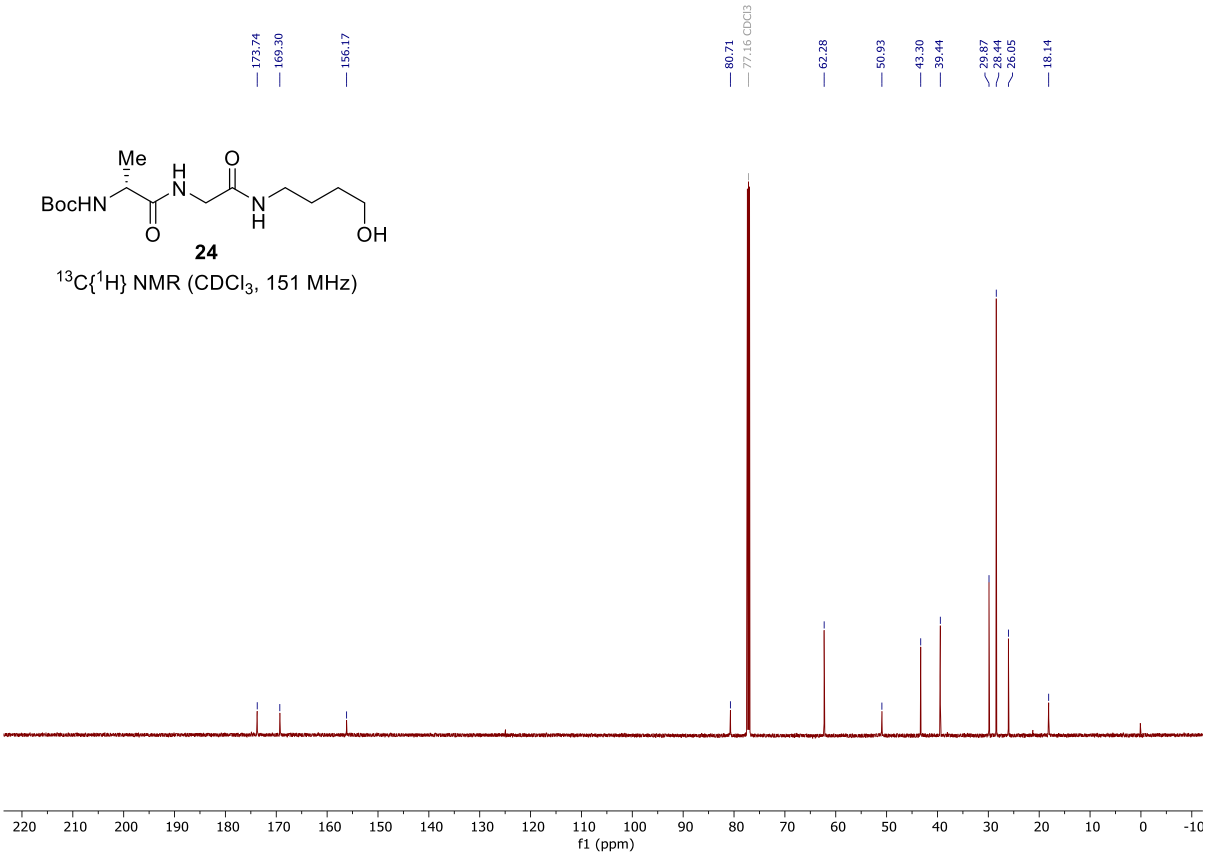

Figure S51

4.8 NMR spectra of 25

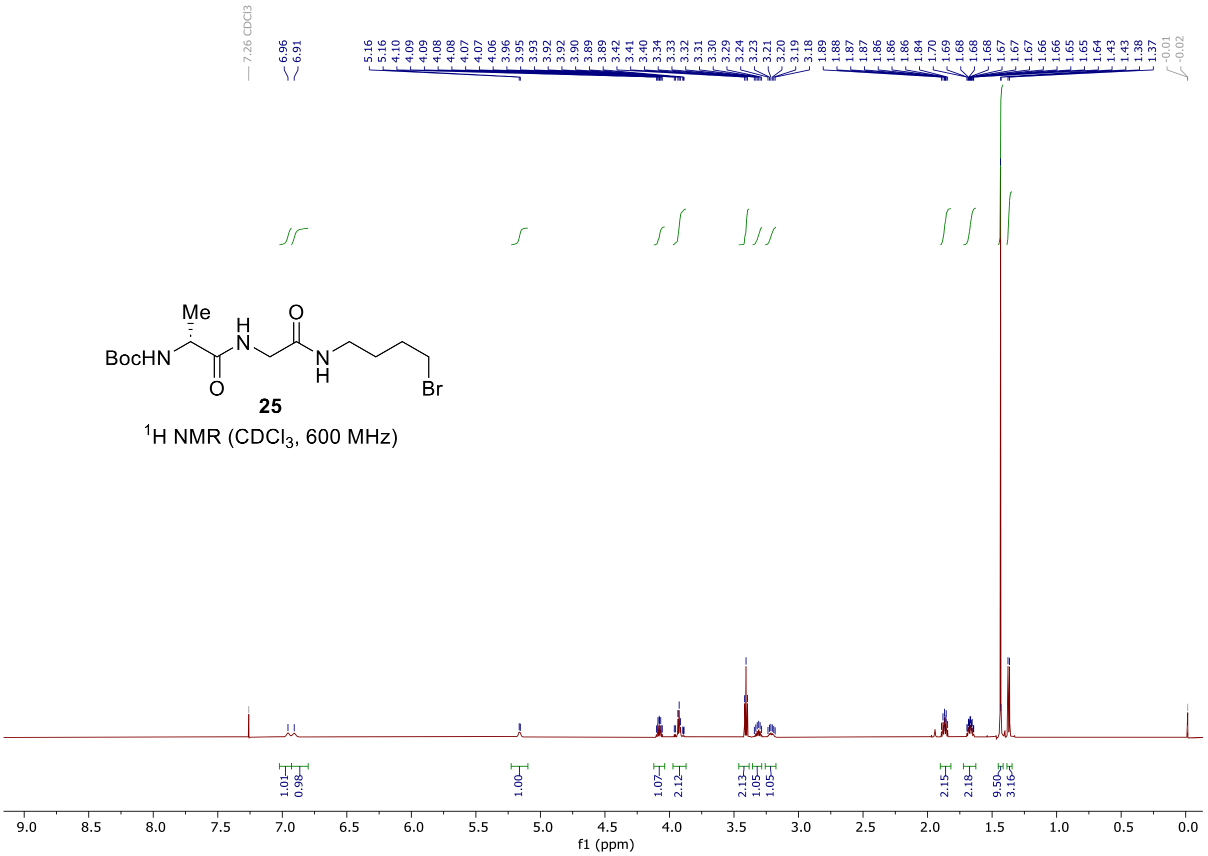

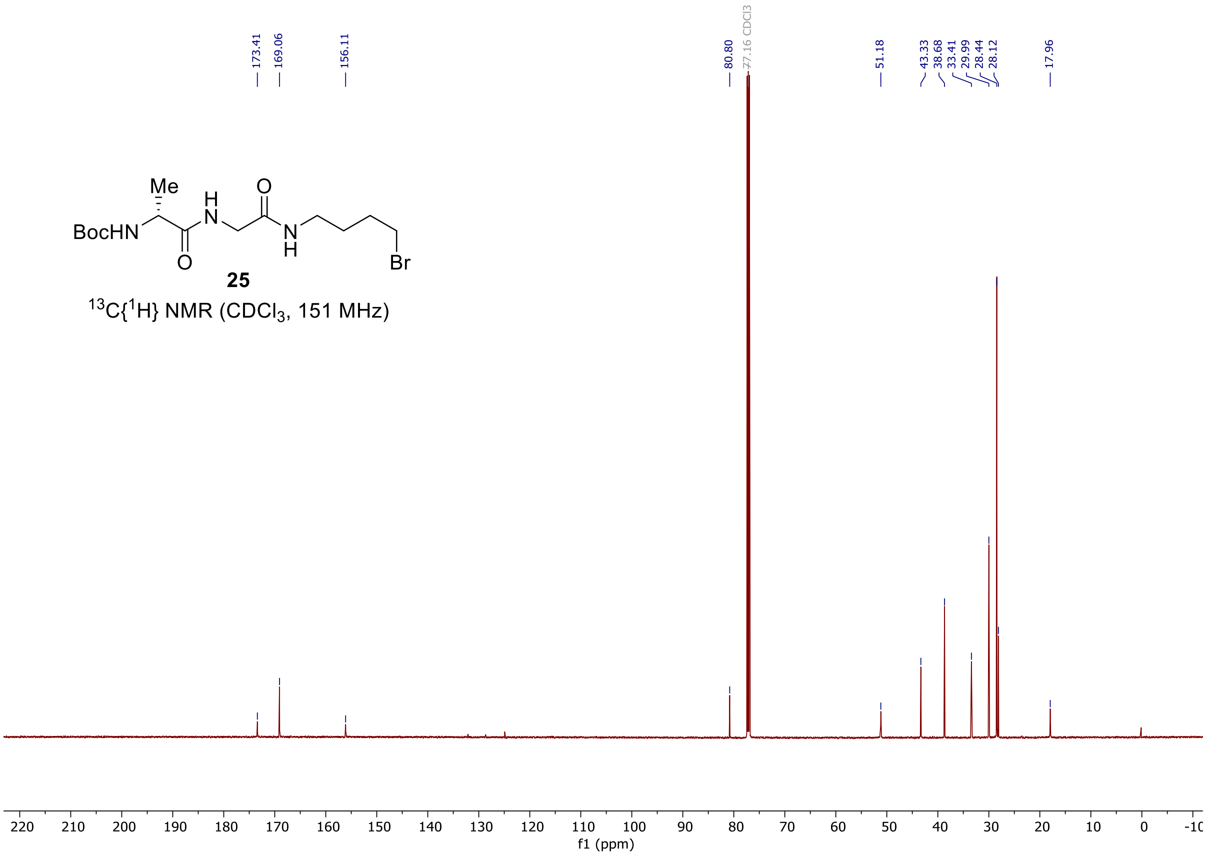

Figure S52

4.9 NMR spectra of 1t

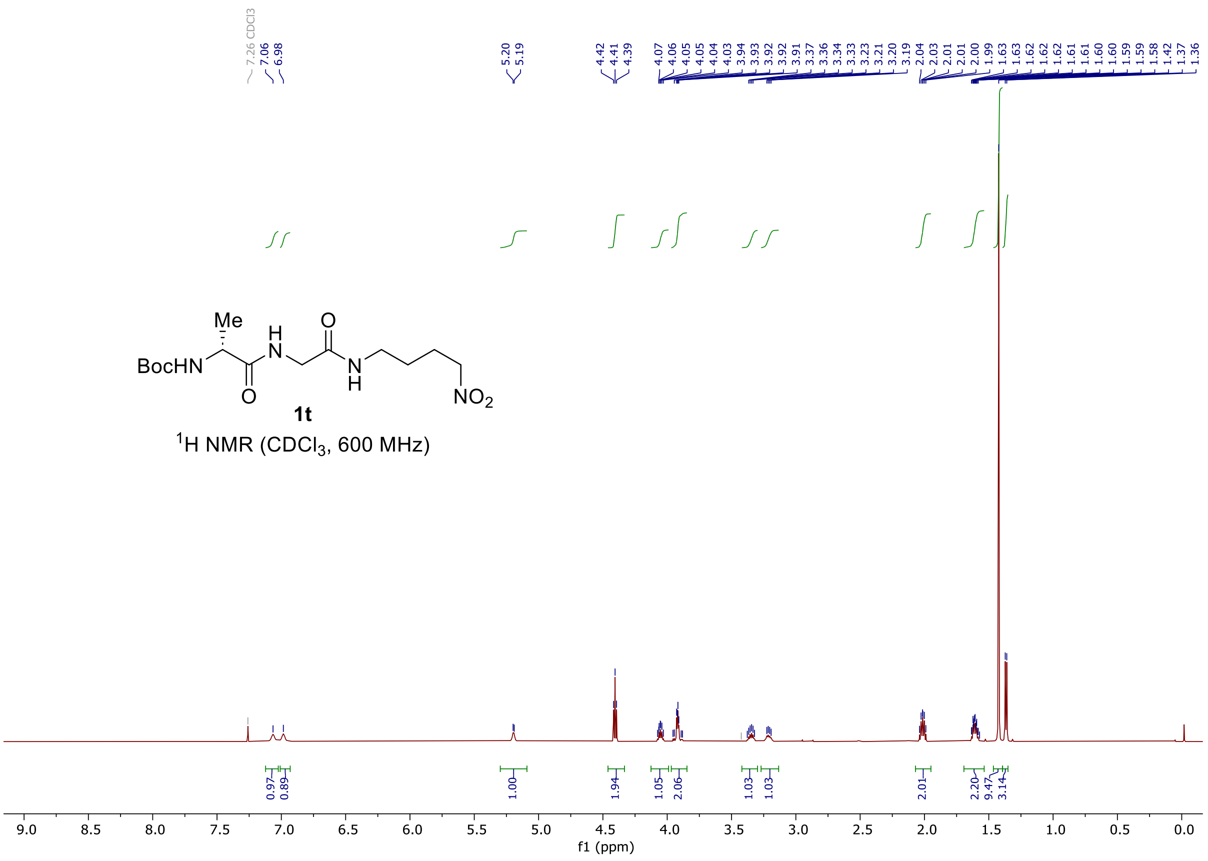

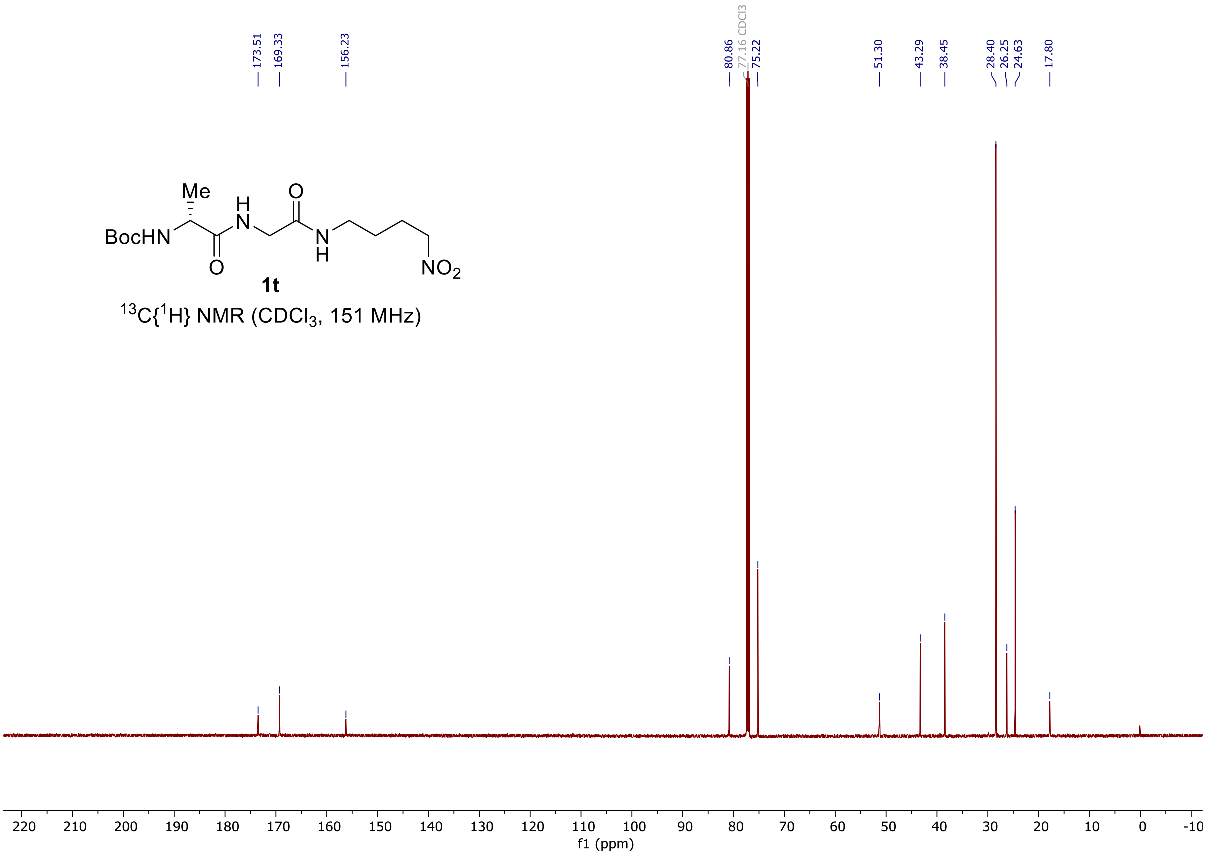

Figure S53

4.10 NMR spectra of 28

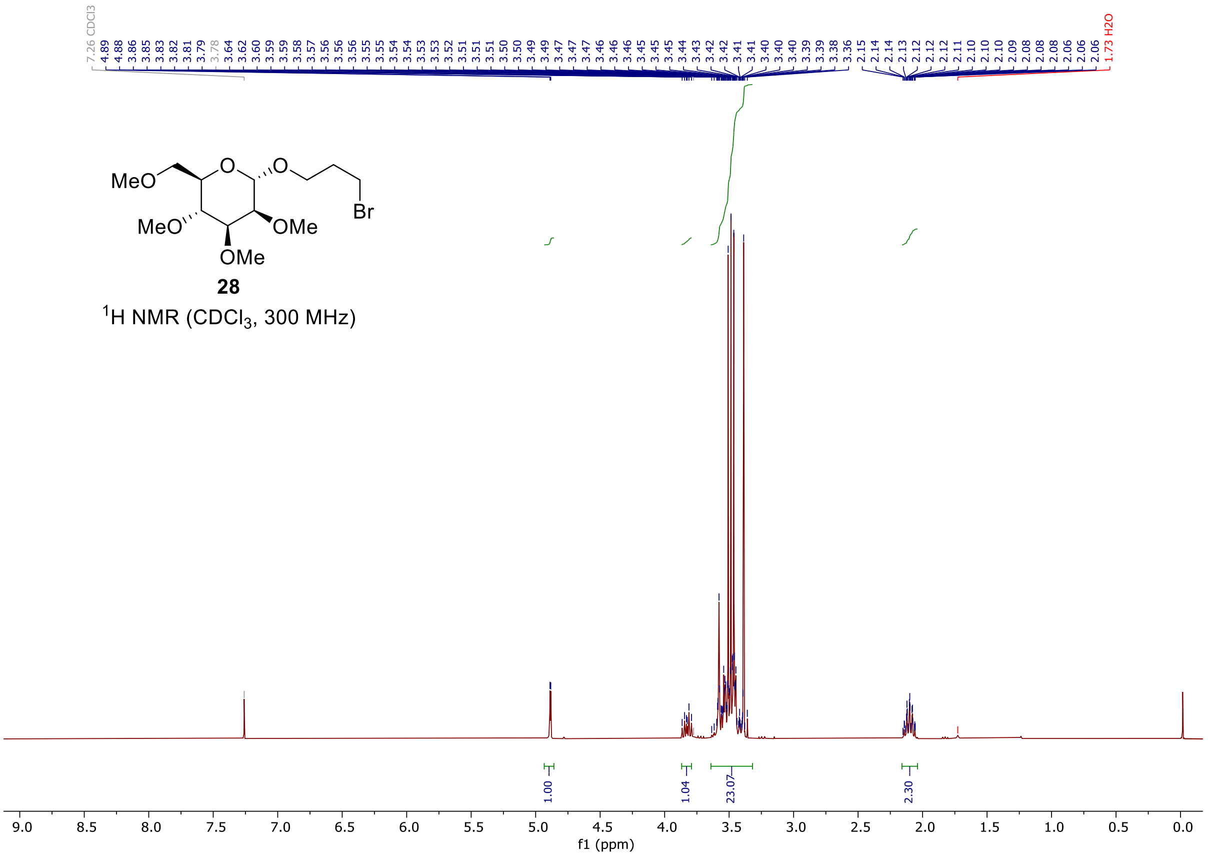

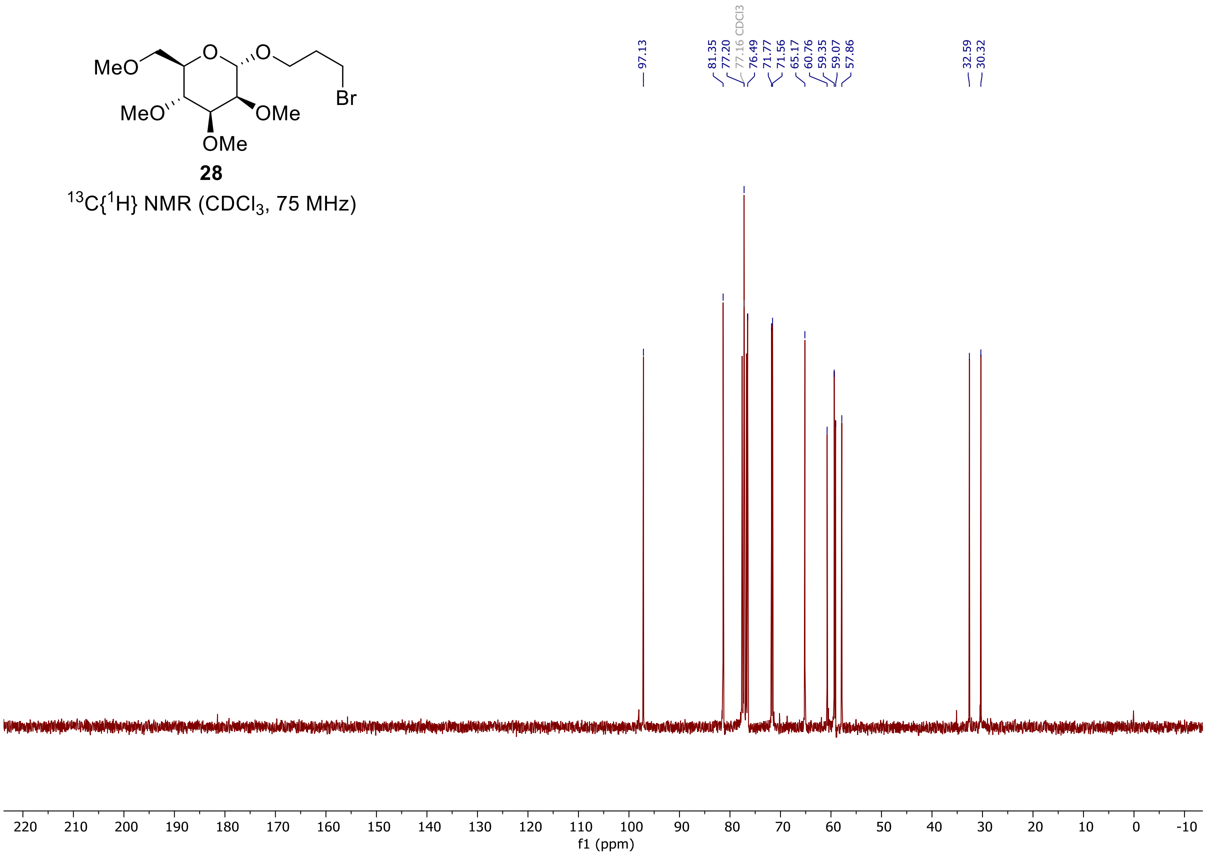

Figure S54

4.11 NMR spectra of 1u

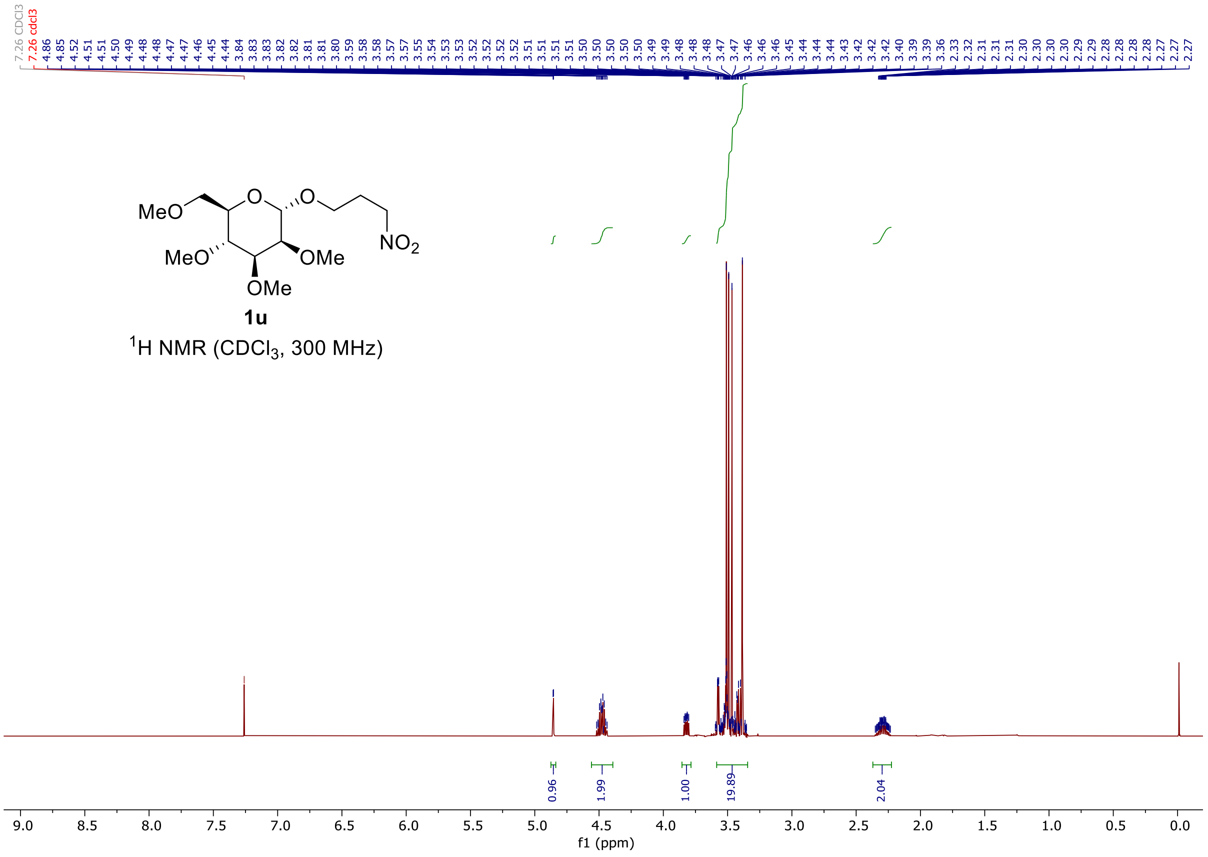

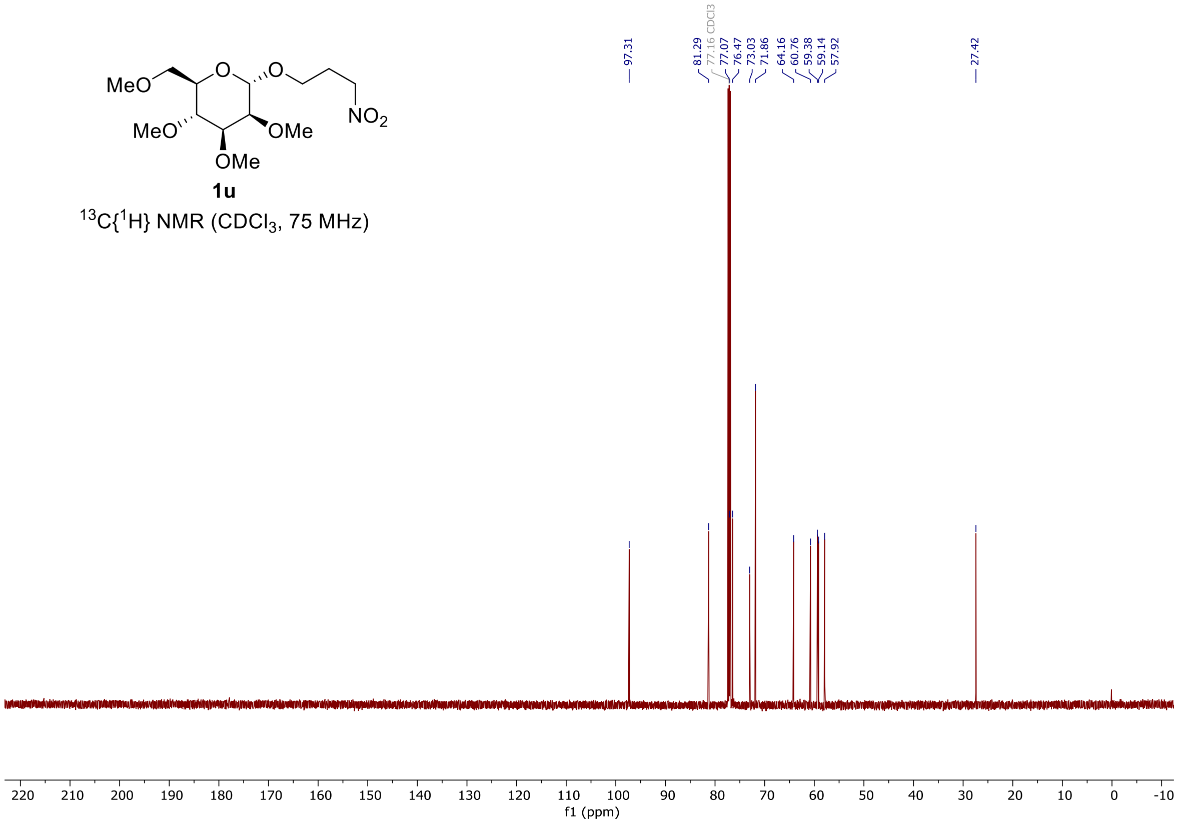

Figure S55

4.12 NMR spectra of 3a

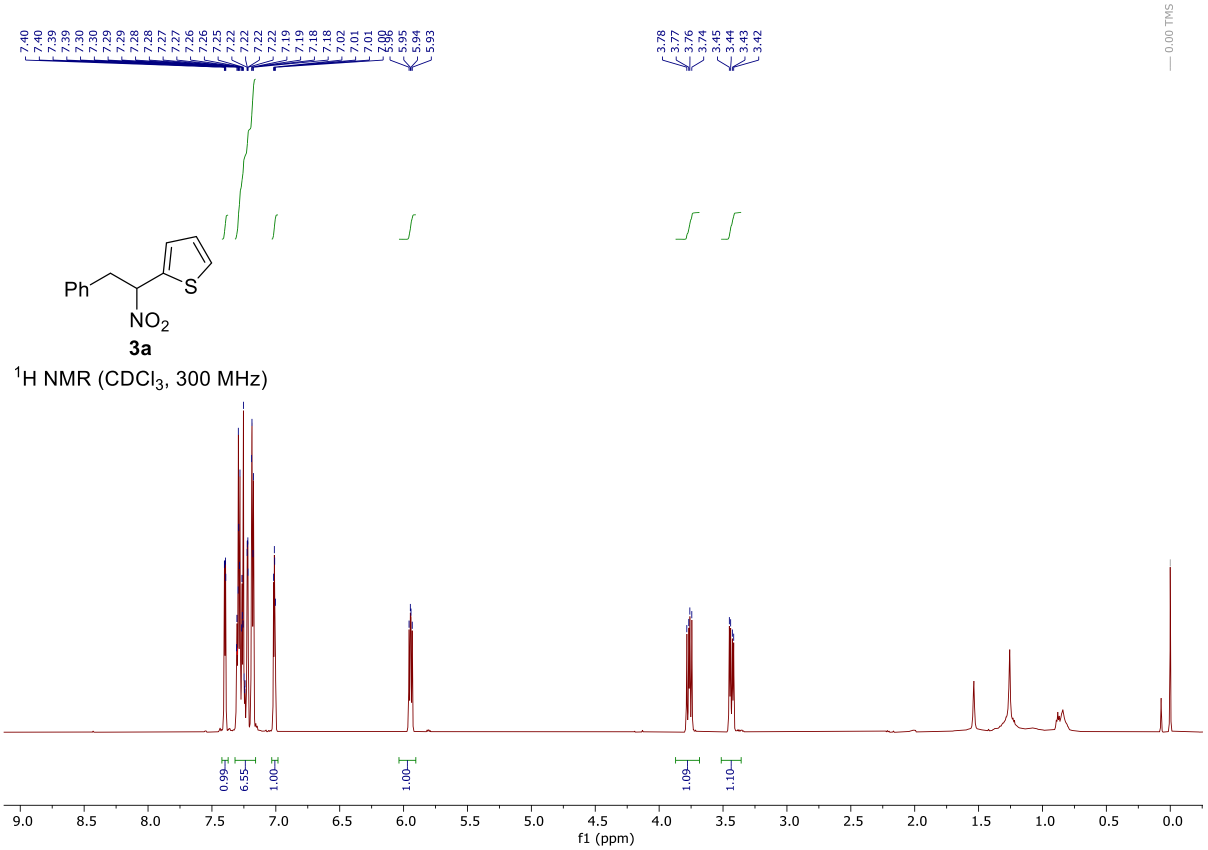

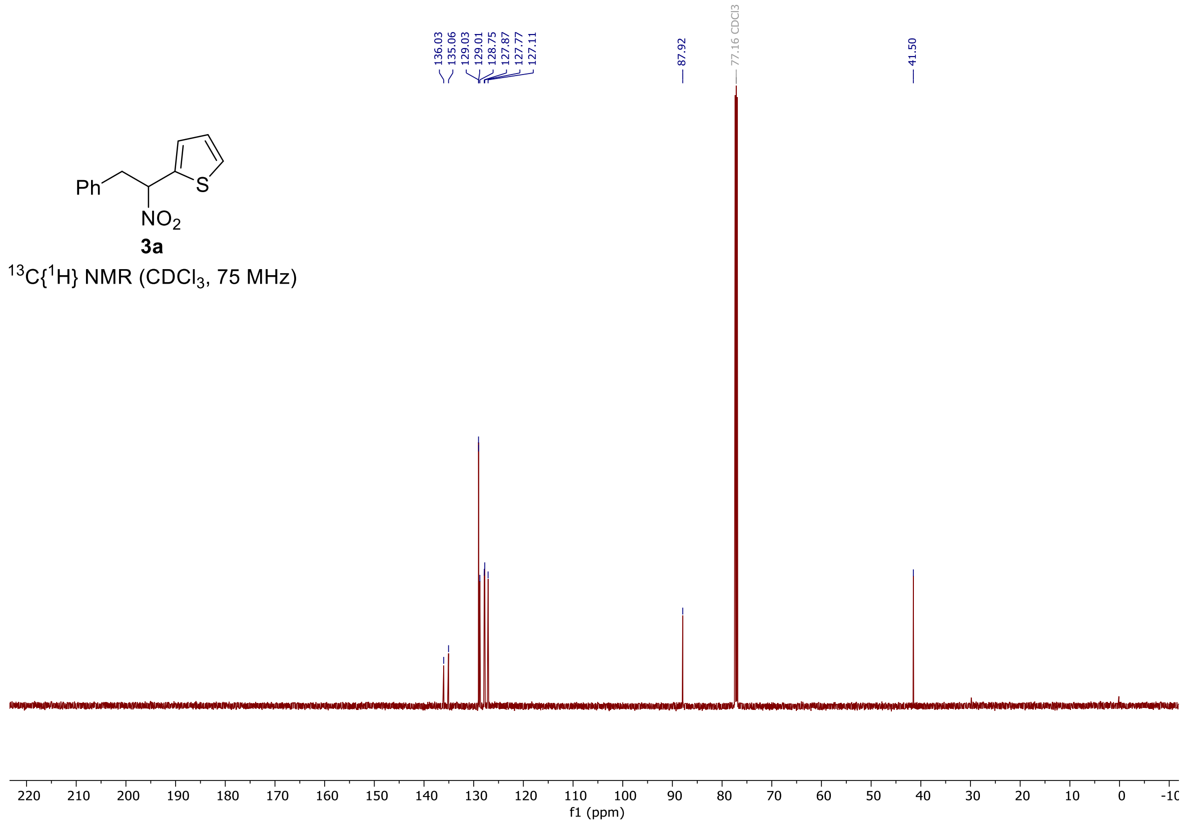

Figure S56

4.13 NMR spectra of 3b

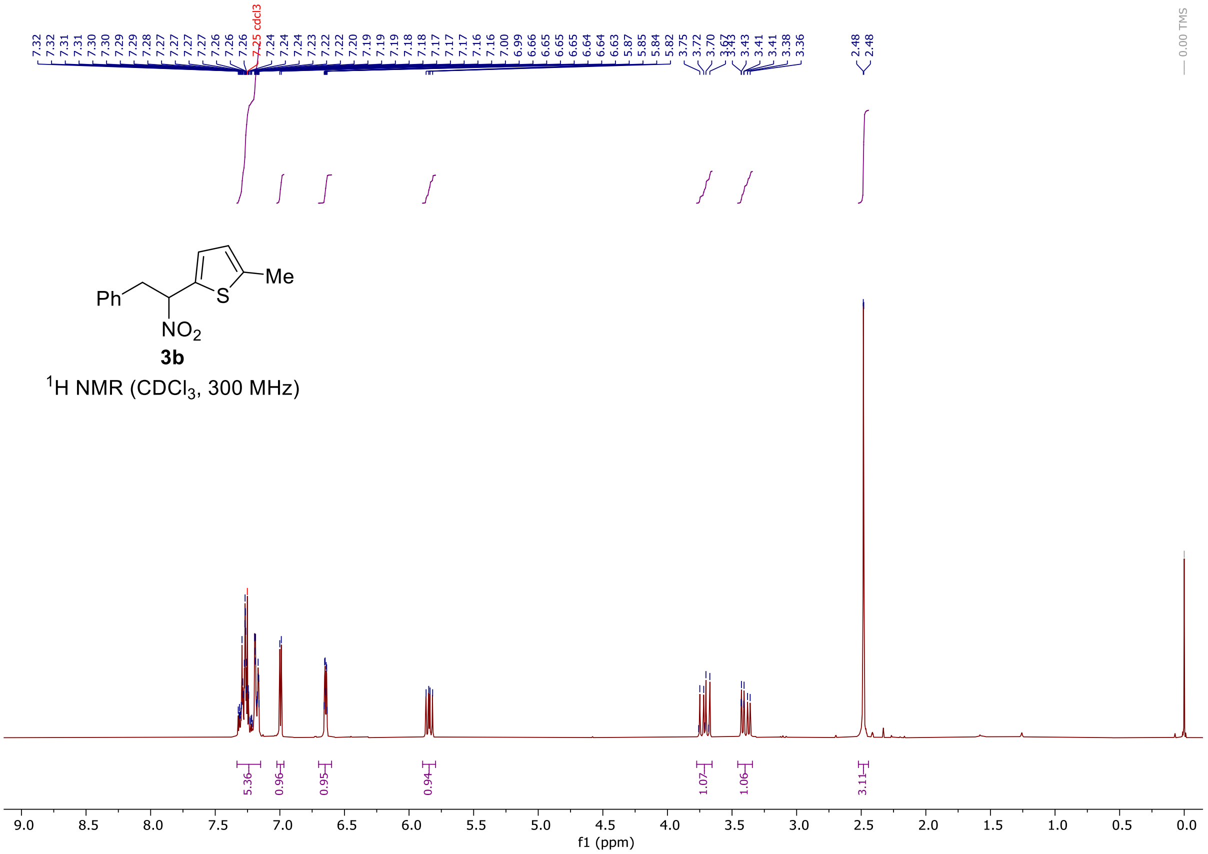

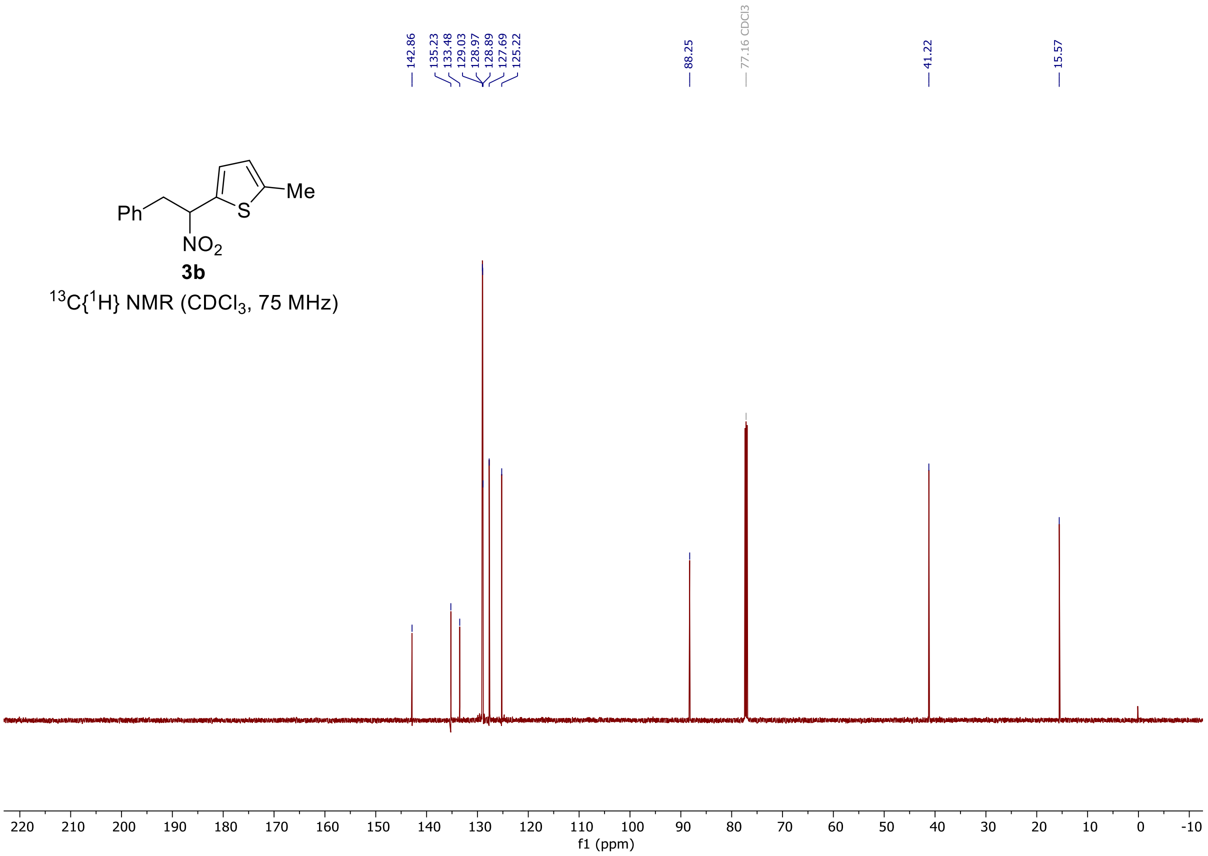

Figure S57

4.14 NMR spectra of 3c

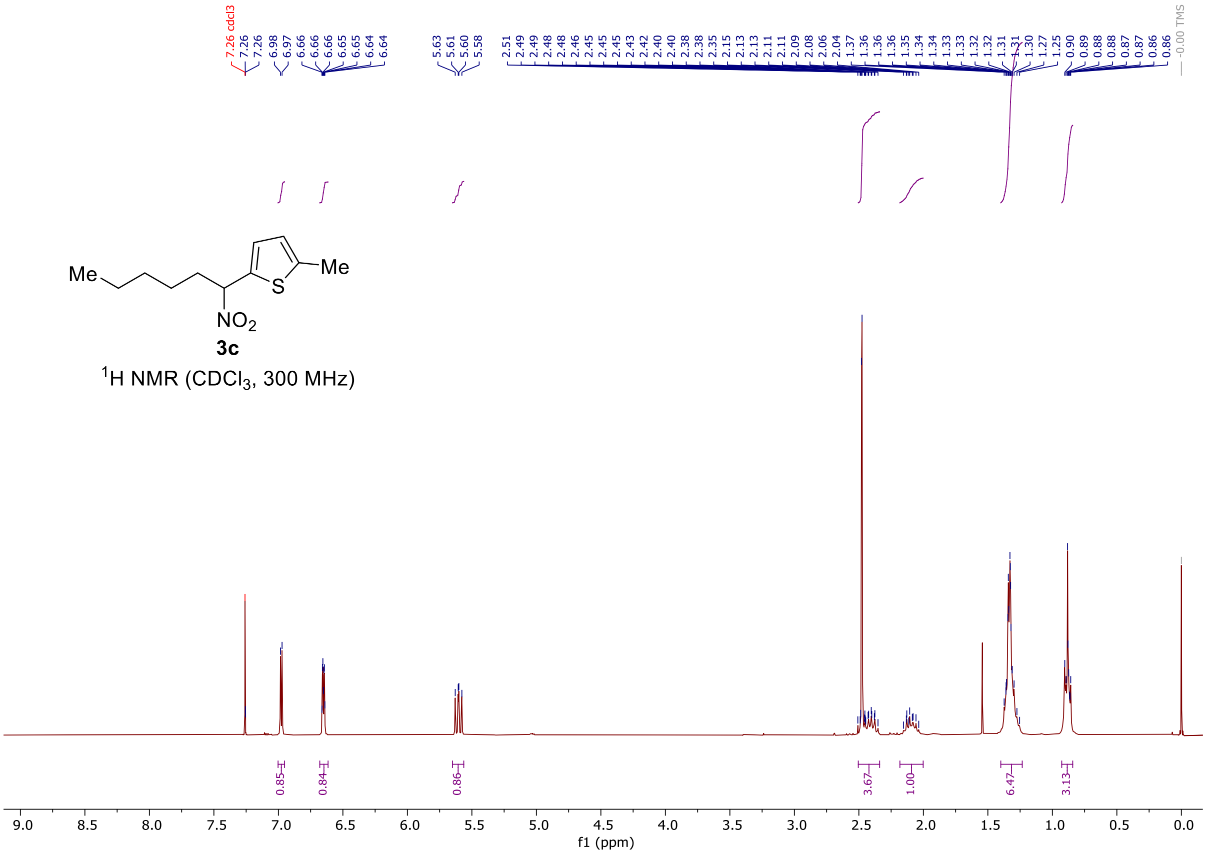

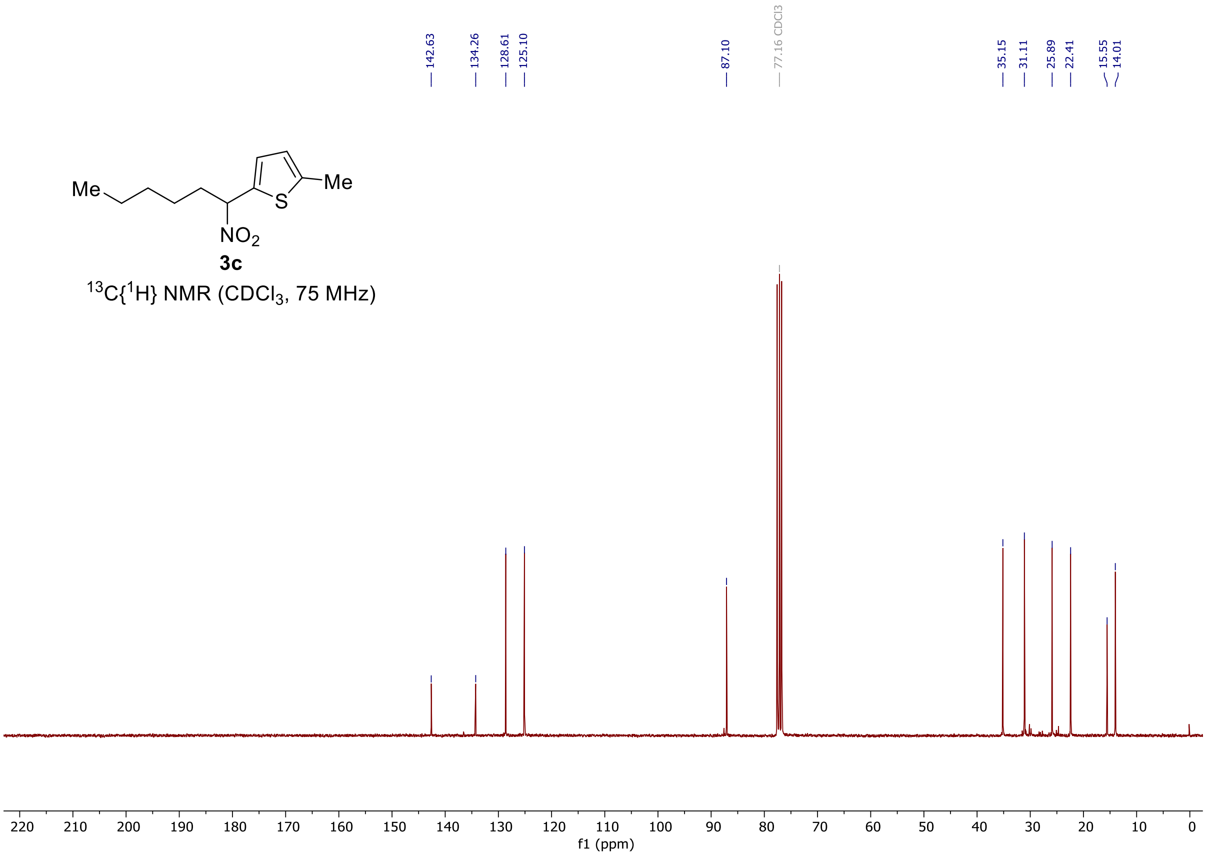

Figure S58

4.15 NMR spectra of 3d

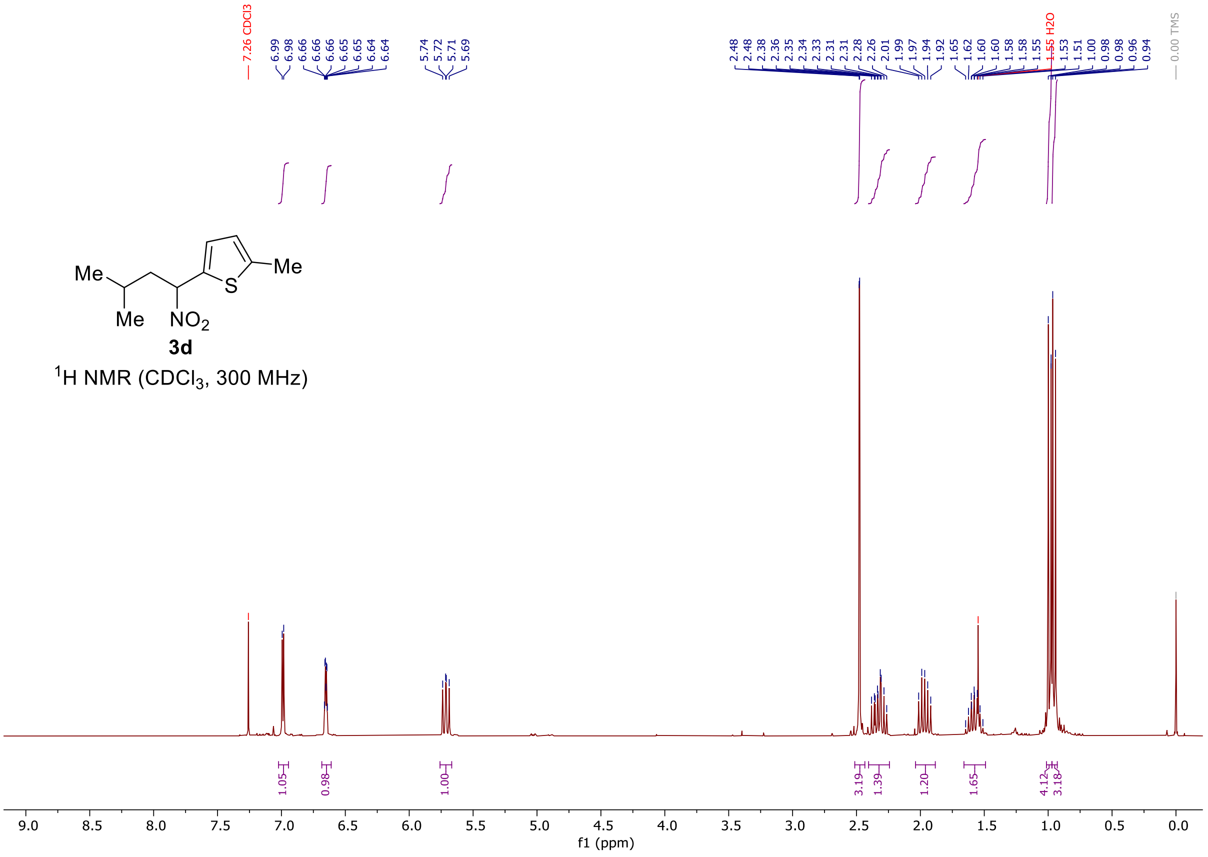

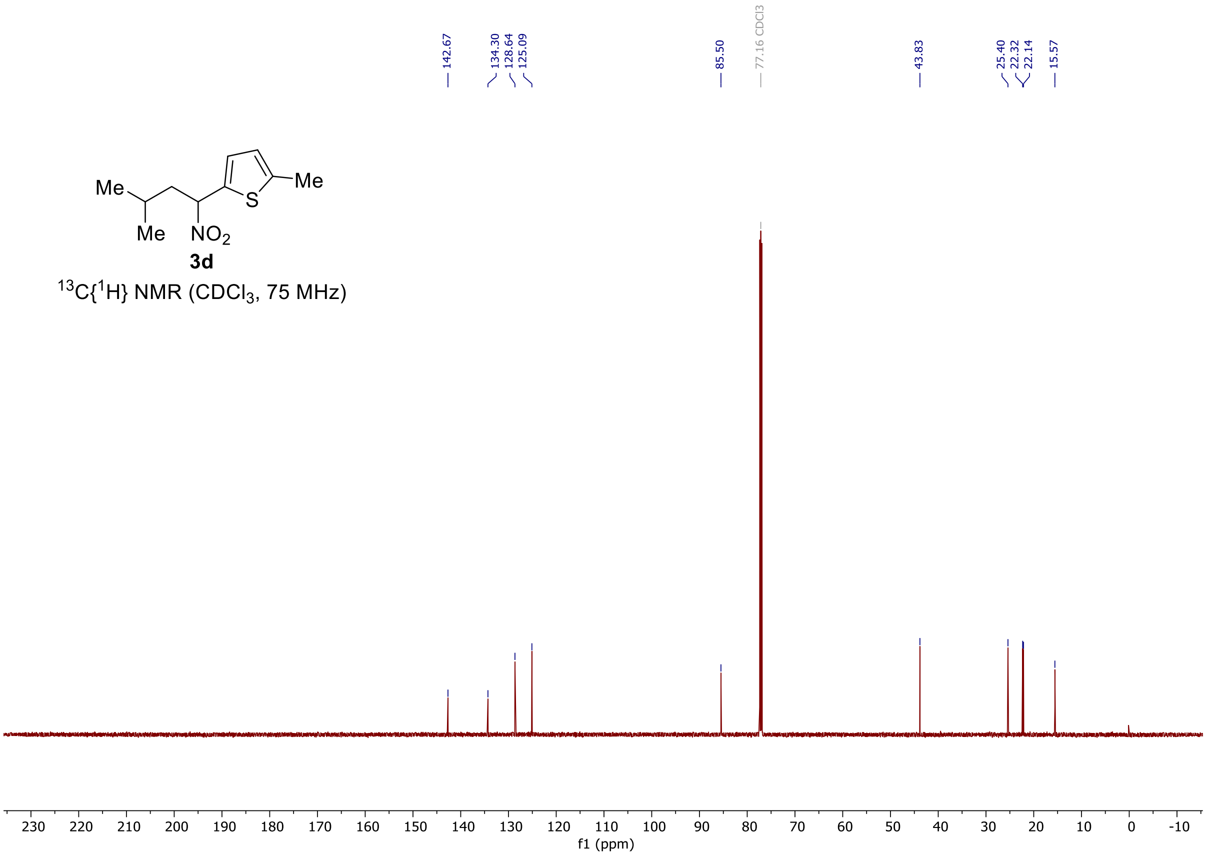

Figure S59

4.16 NMR spectra of 3e

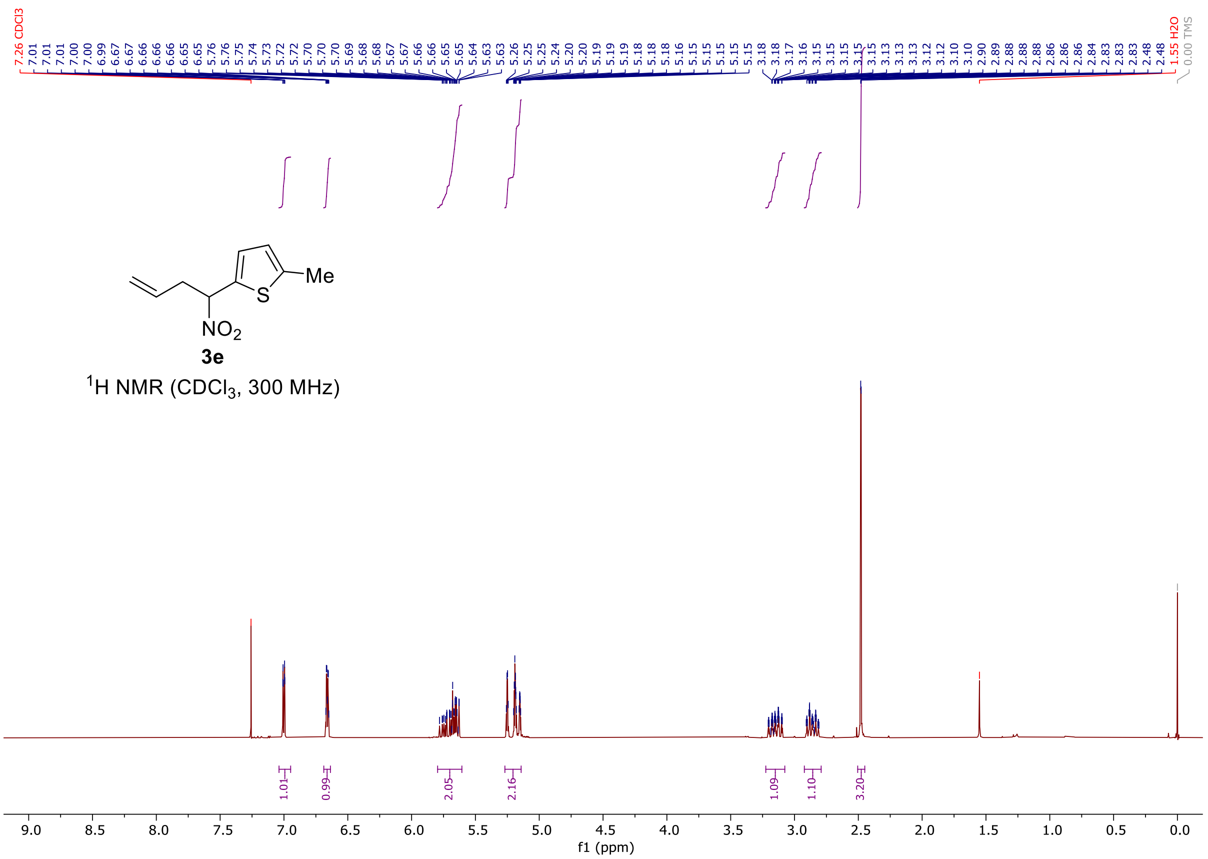

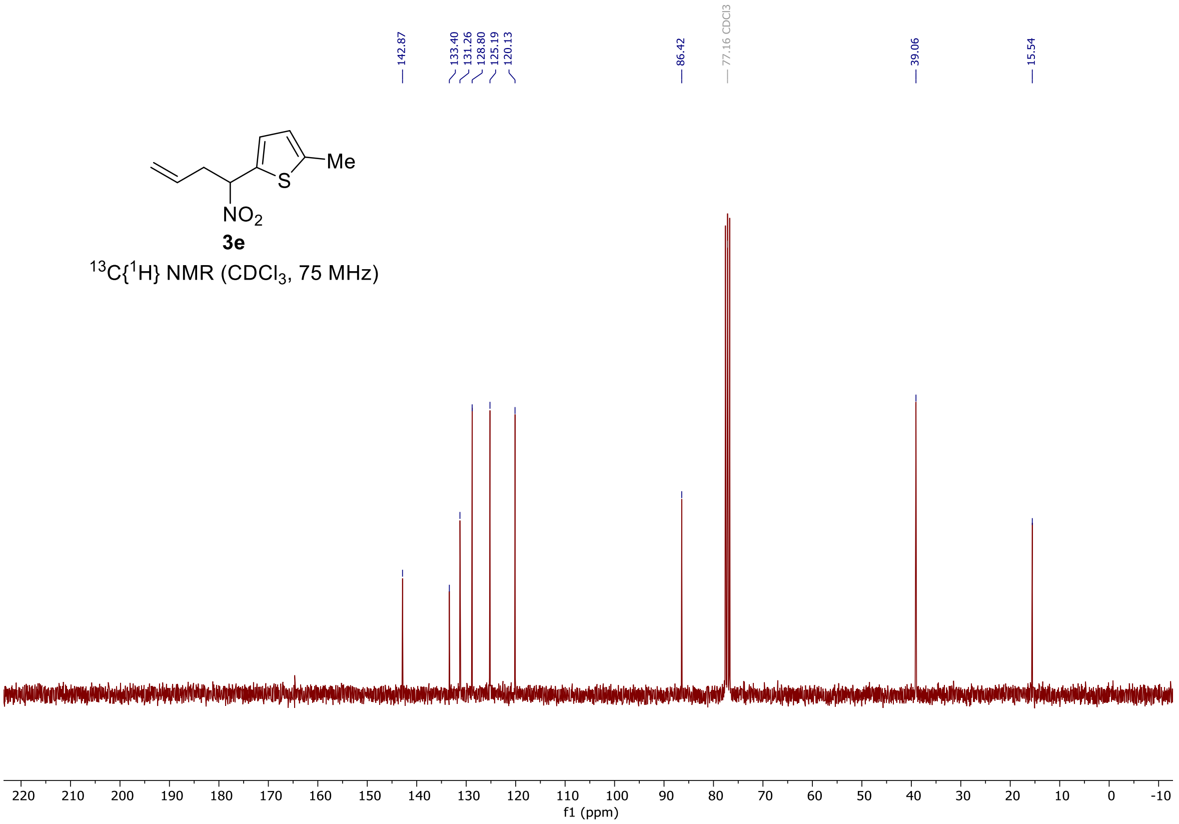

Figure S60

4.17 NMR spectra of 3f

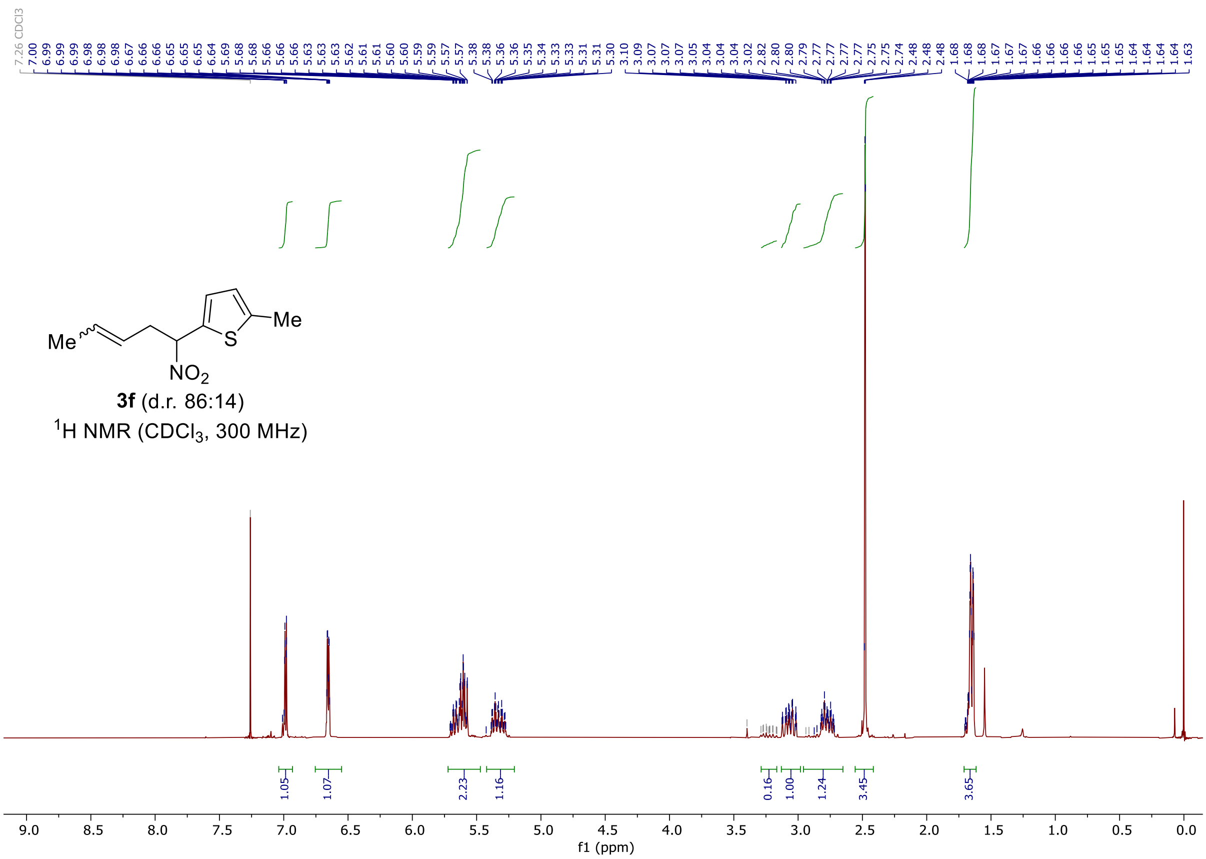

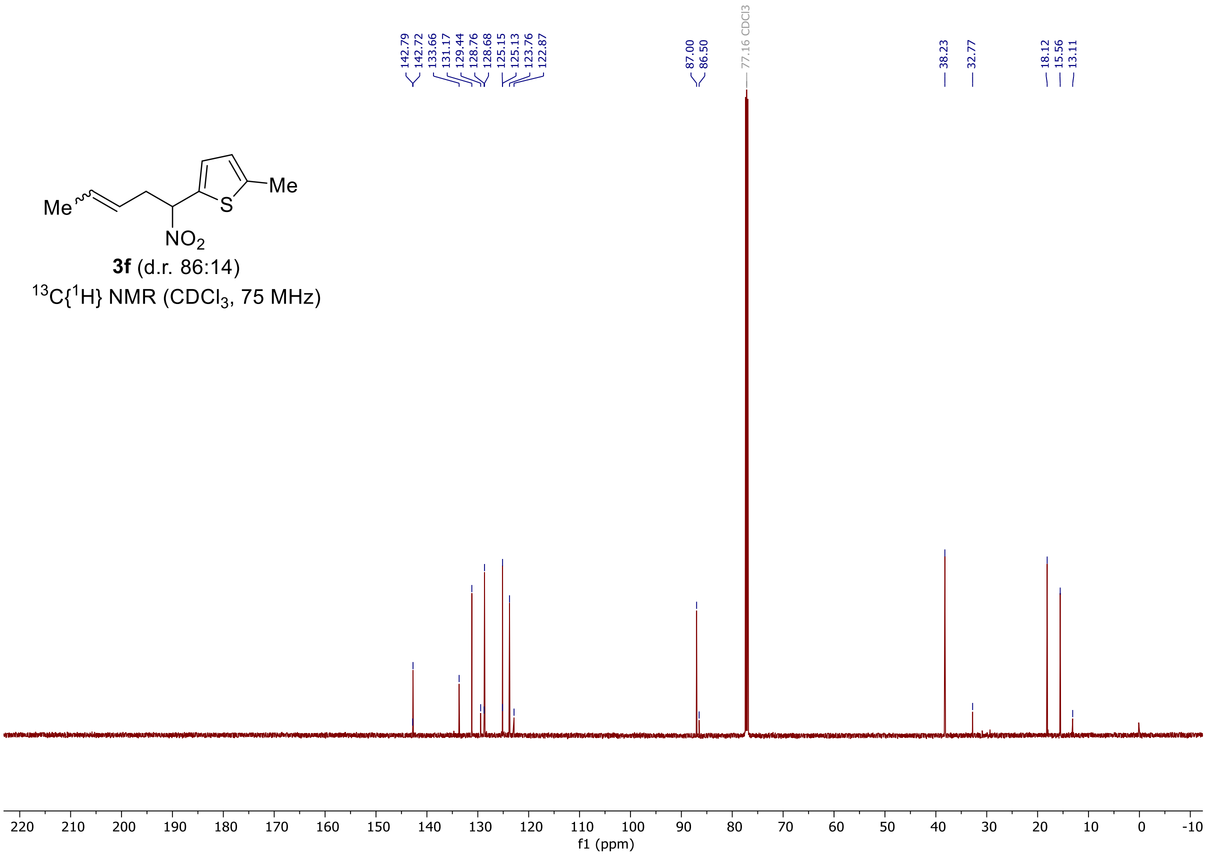

Figure S61

4.18 NMR spectra of 3g

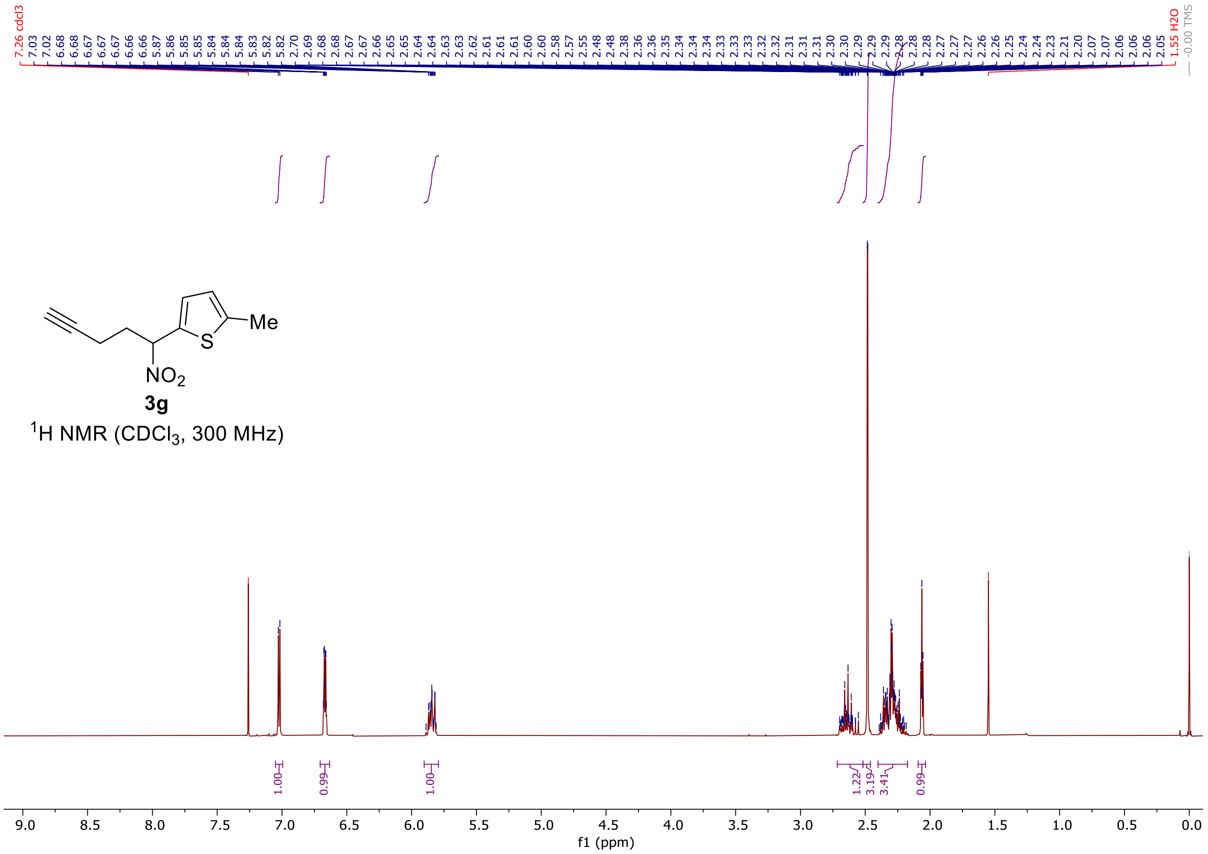

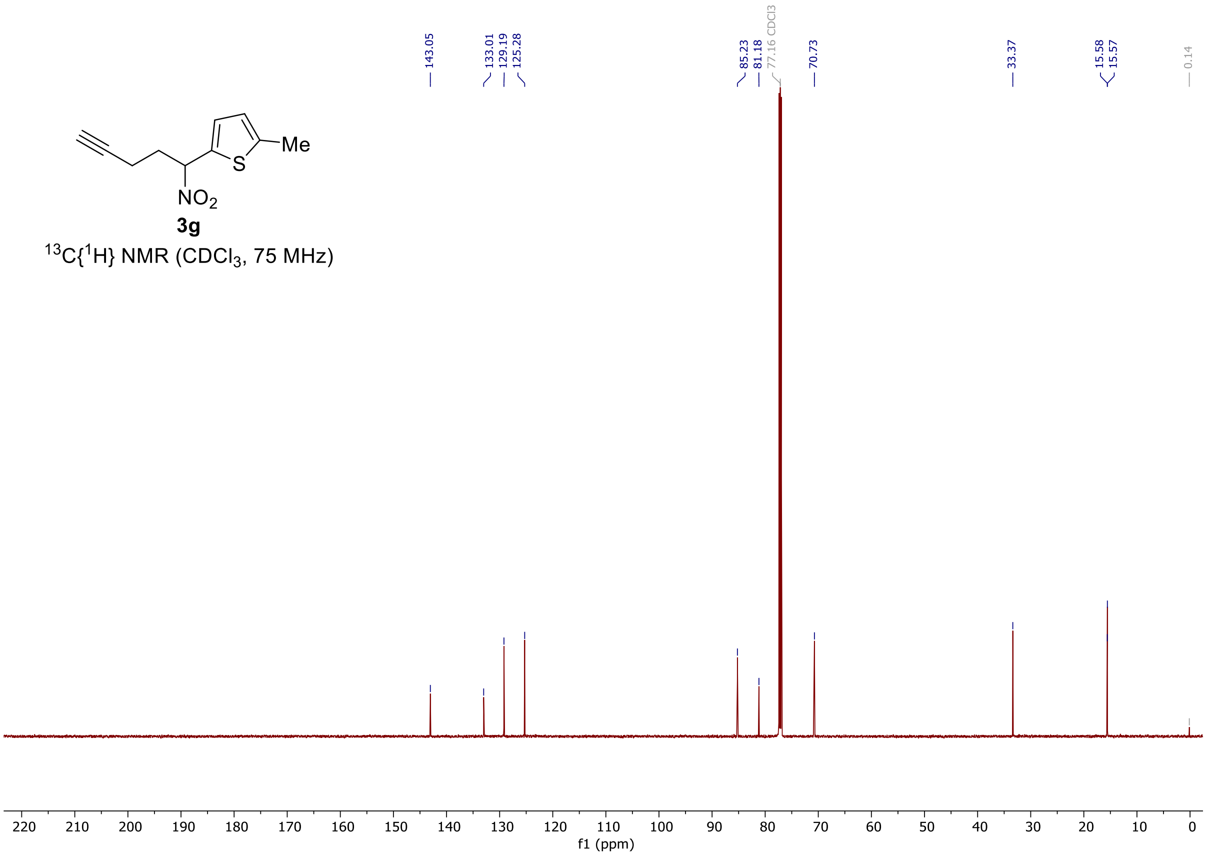

Figure S62

4.19 NMR spectra of 3h

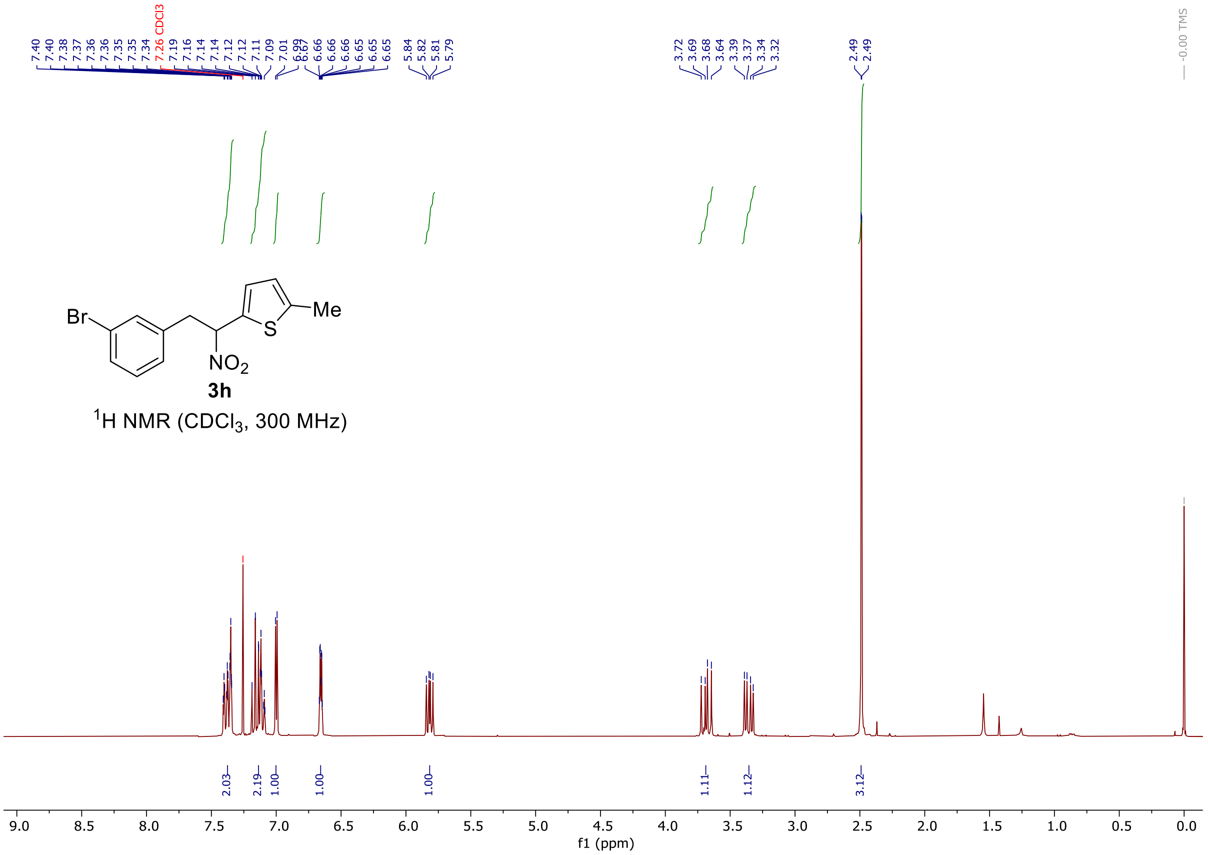

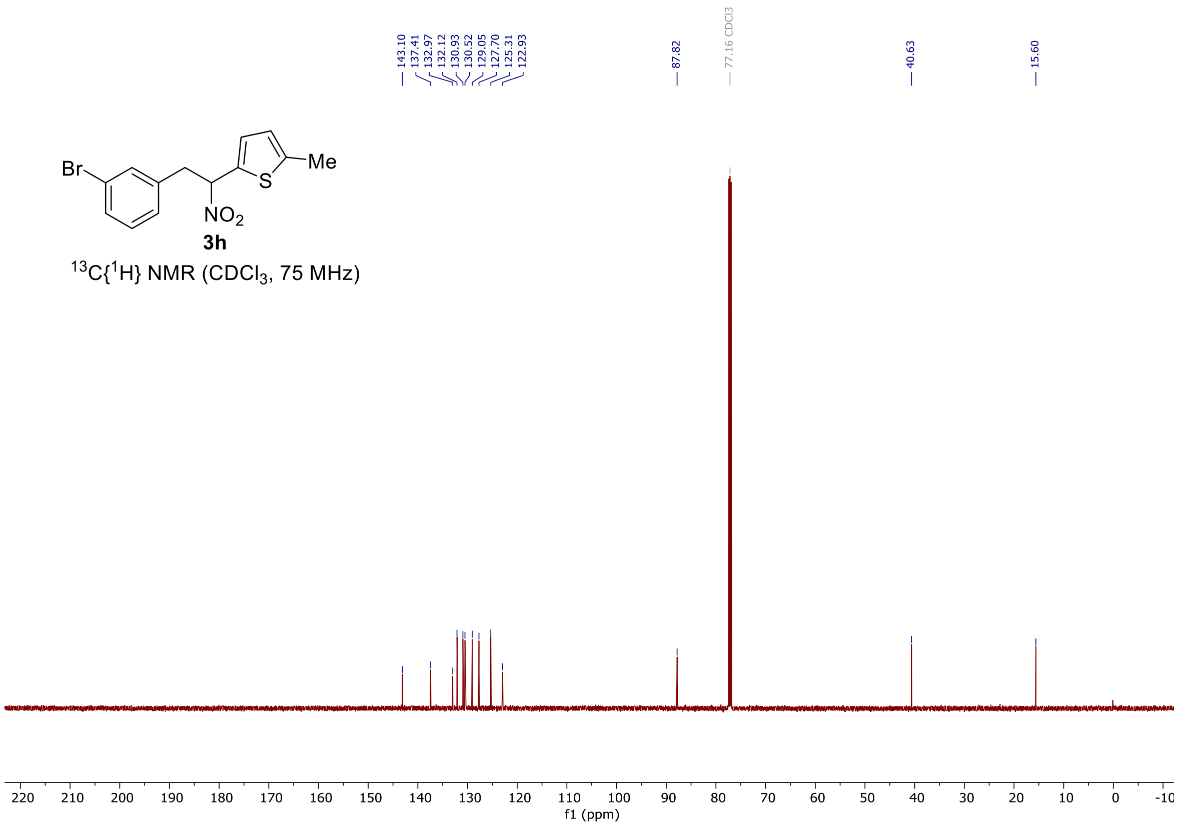

Figure S63

4.20 NMR spectra of 3i

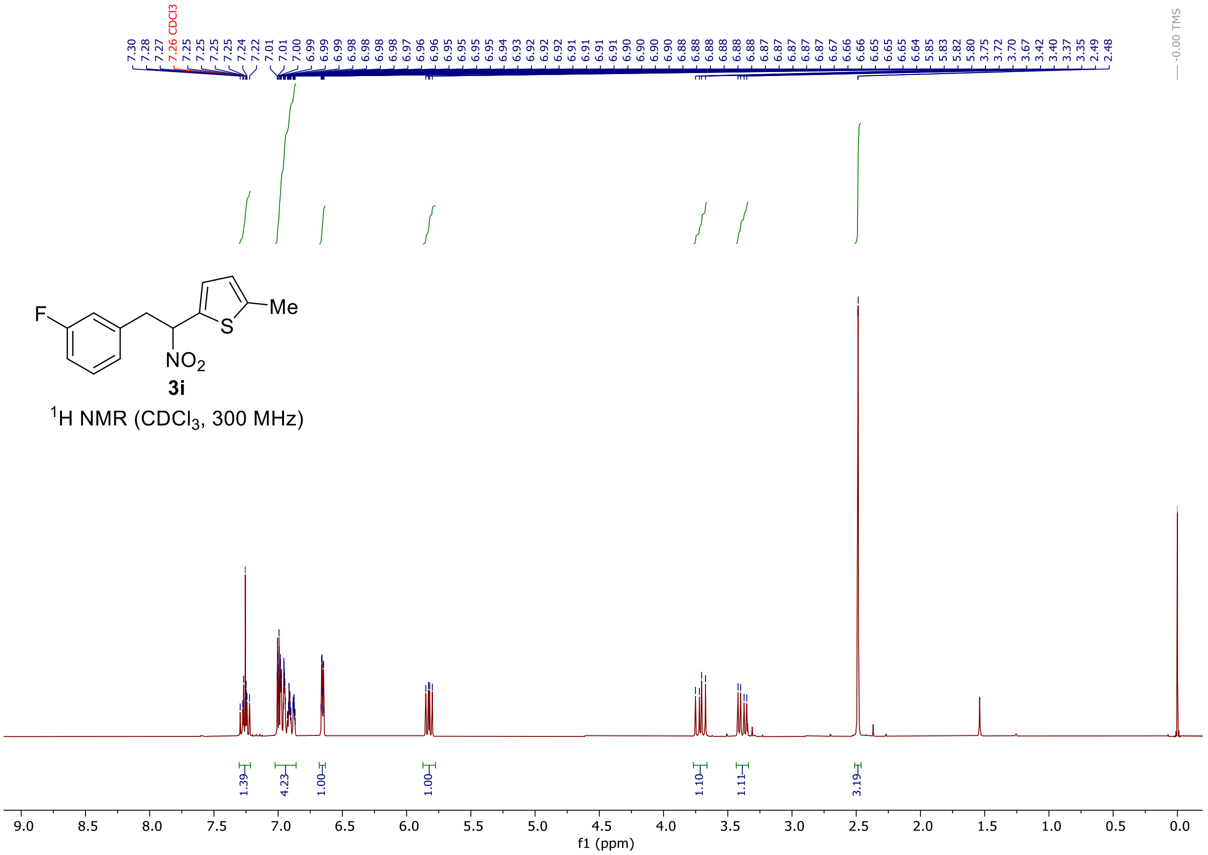

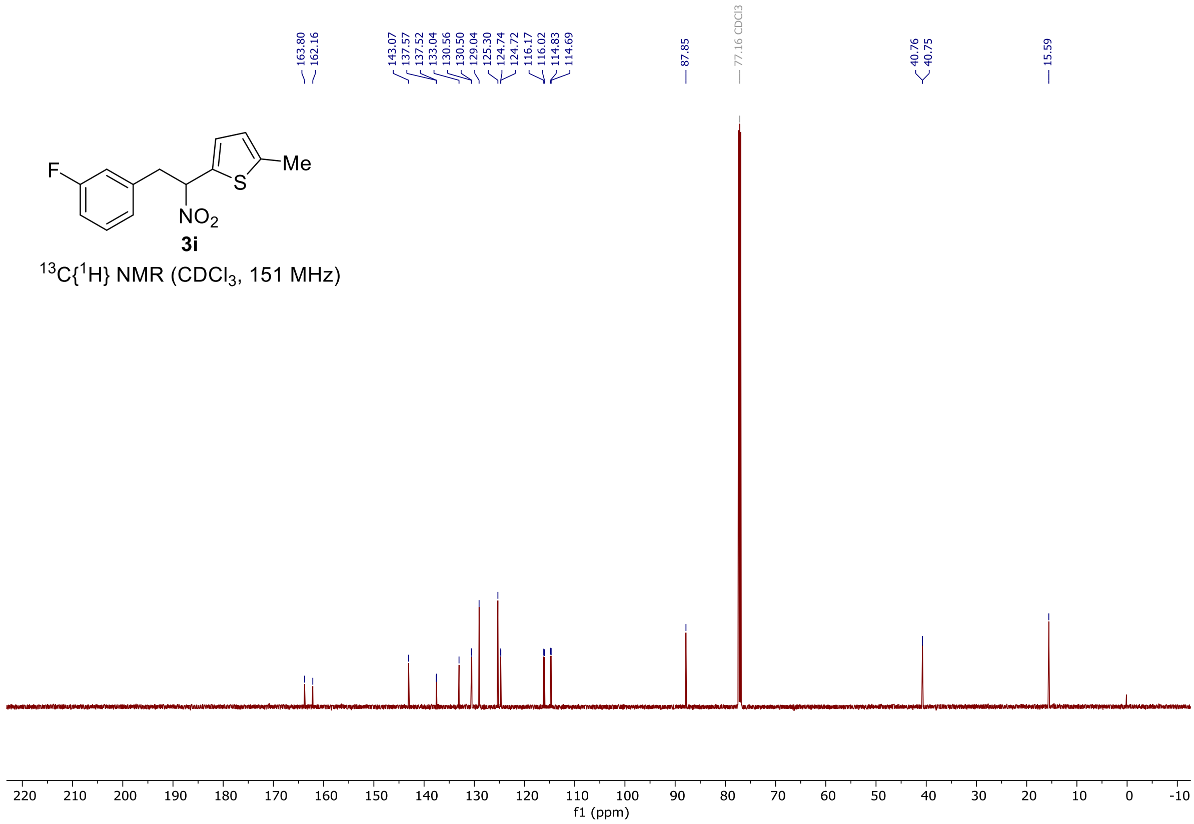

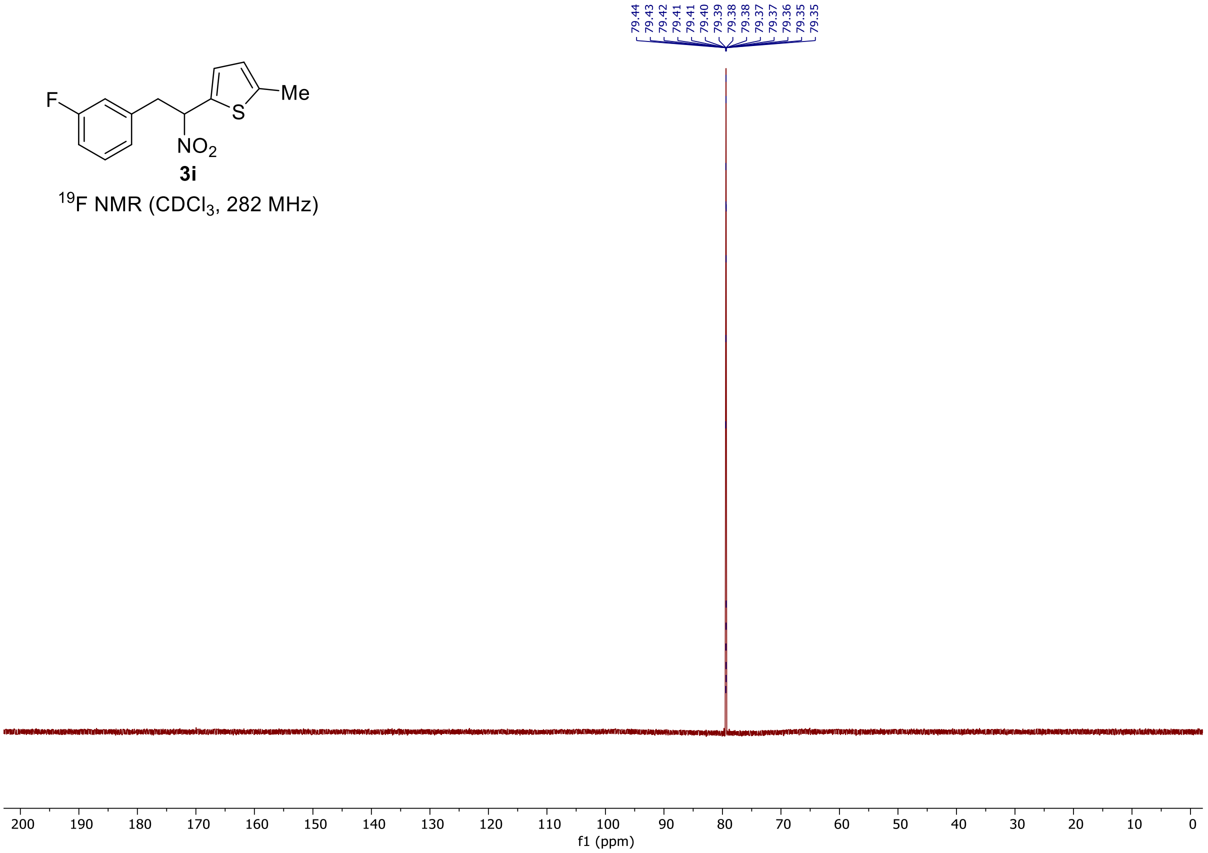

3i

^{19}F NMR ($CDCl_3$, 282 MHz)

Figure S64

4.21 NMR spectra of 3j

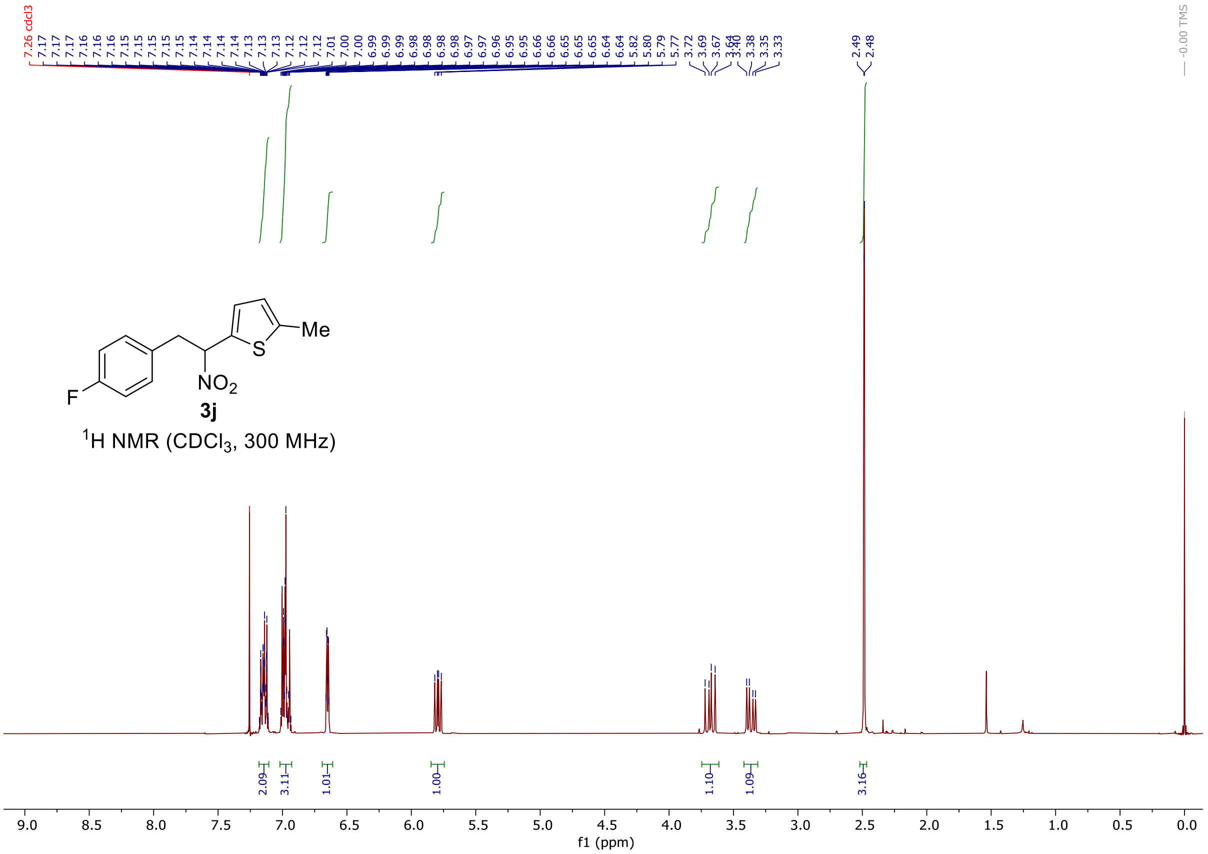

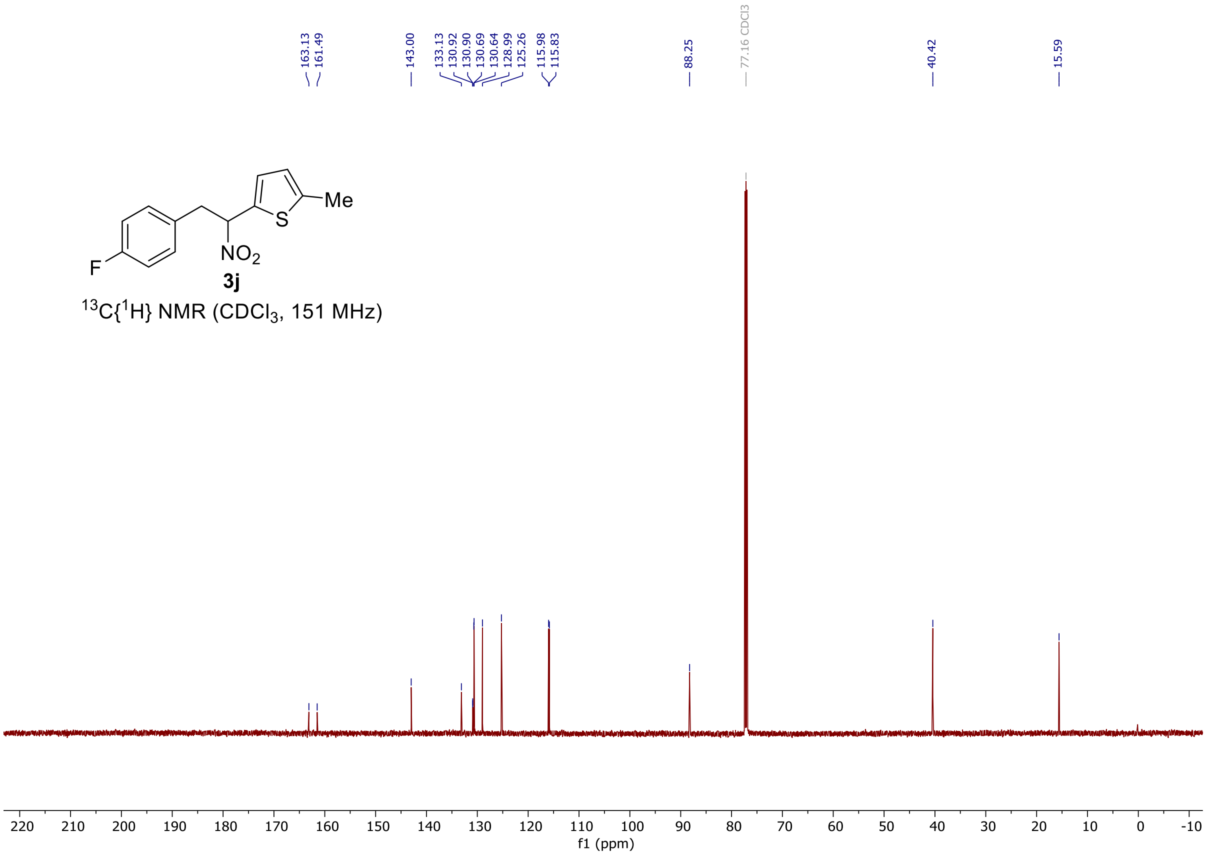

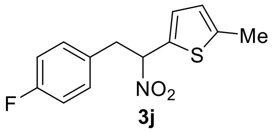

3j

^{19}F NMR ($CDCl_3$, 282 MHz)

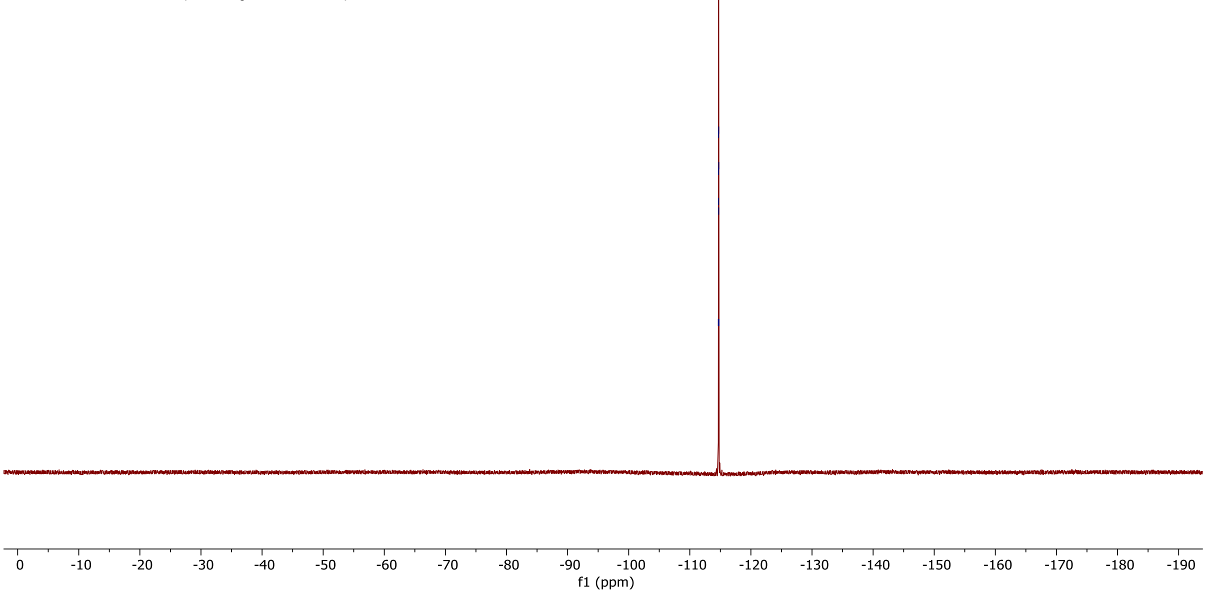

Figure S65

4.22 NMR spectra of 3k

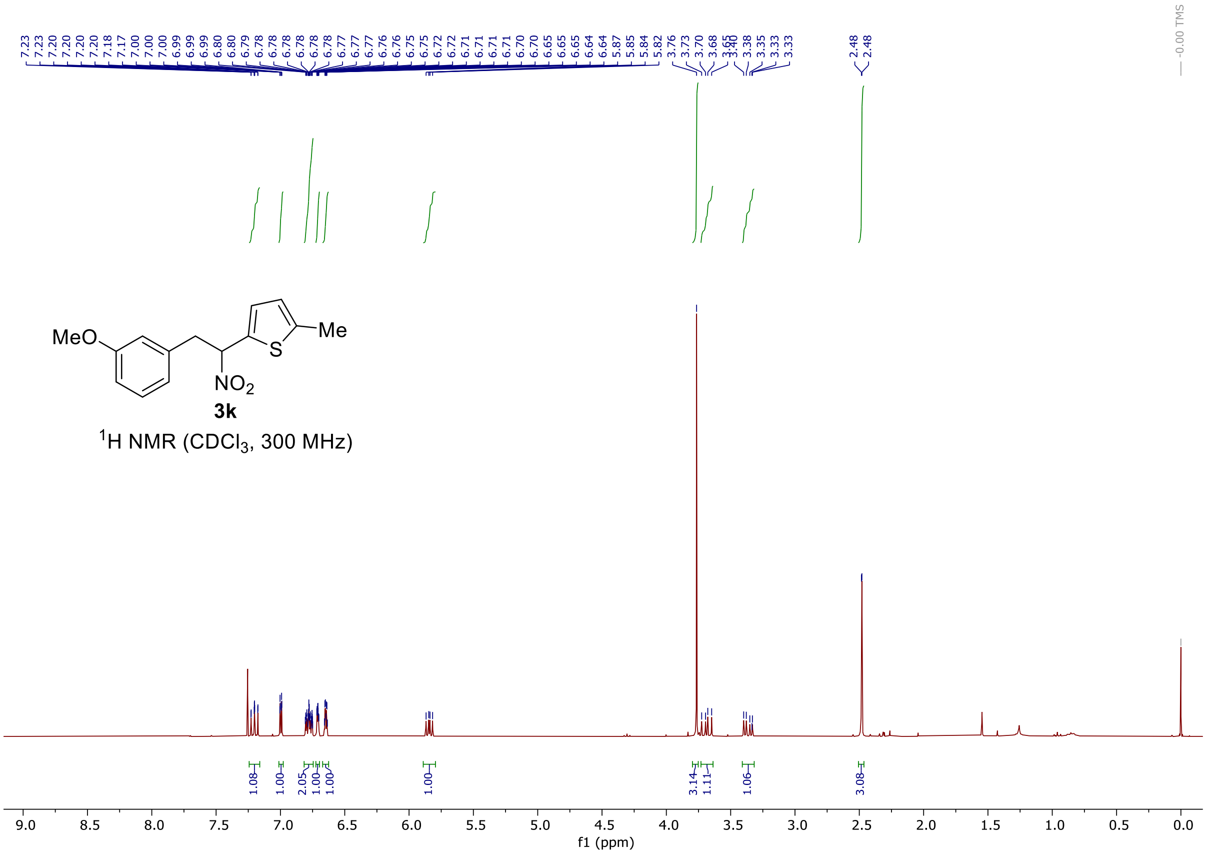

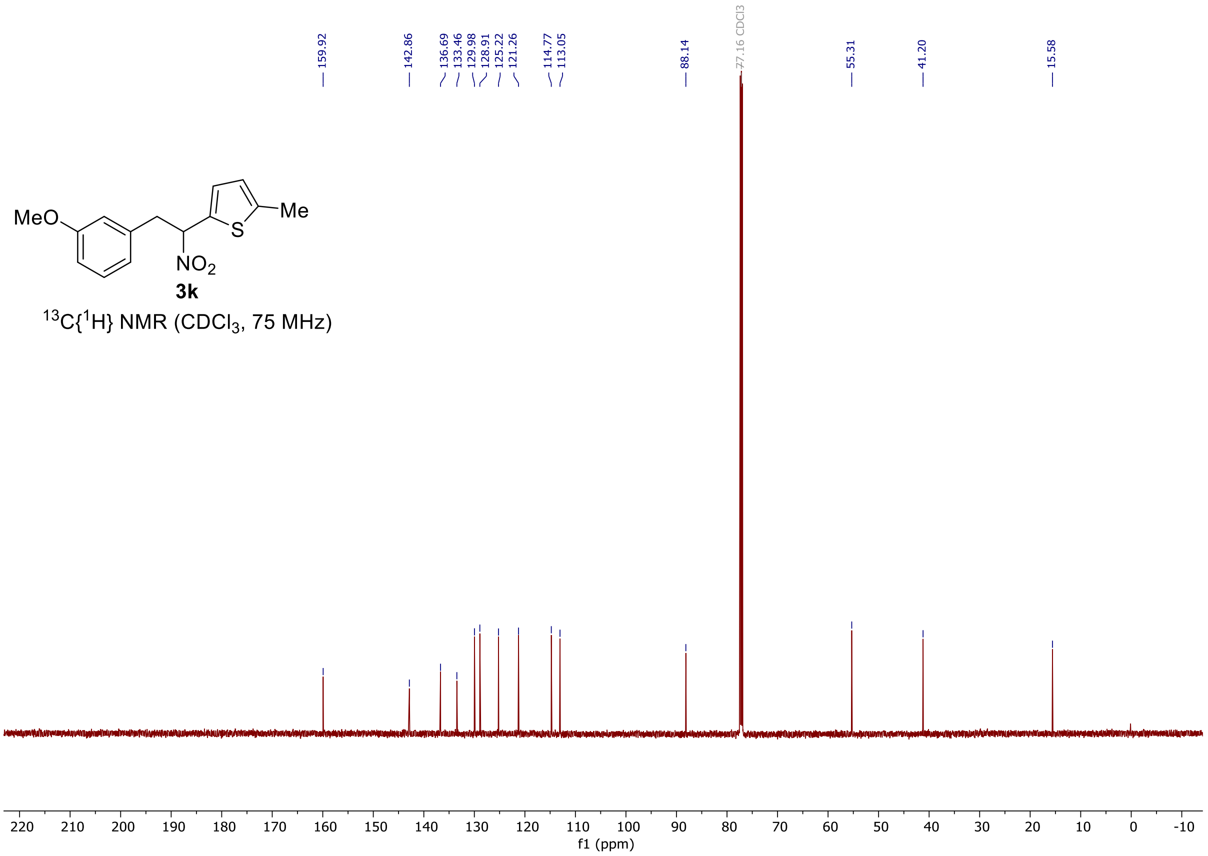

Figure S66

4.23 NMR spectra of 3l

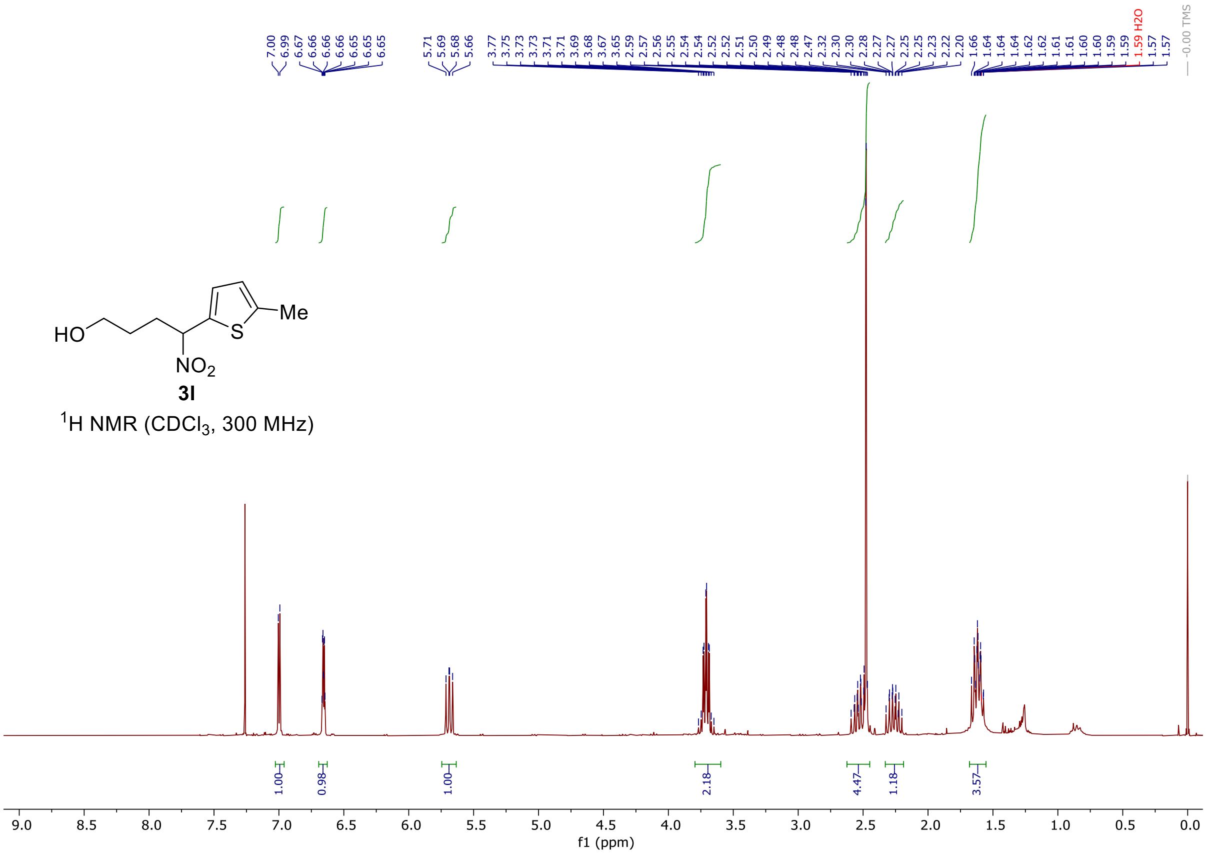

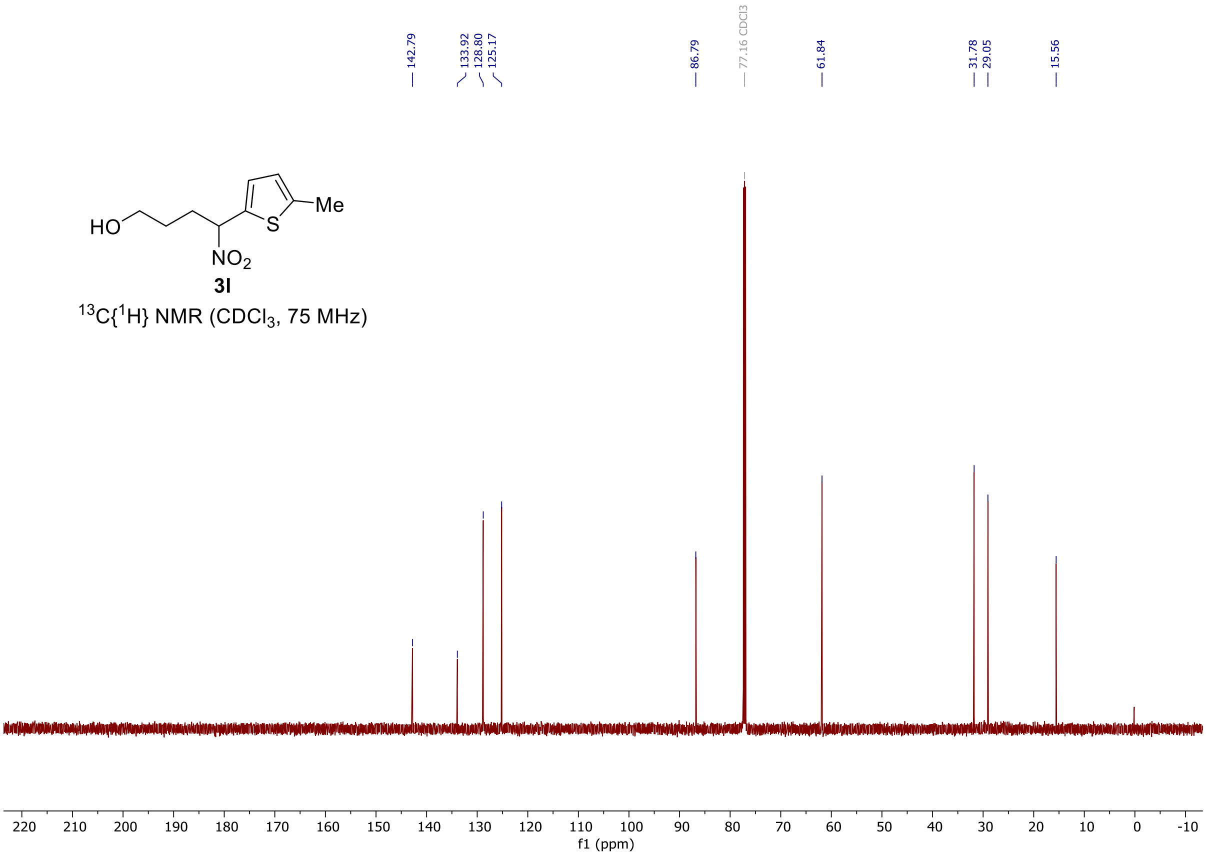

Figure S67

4.24 NMR spectra of 3m

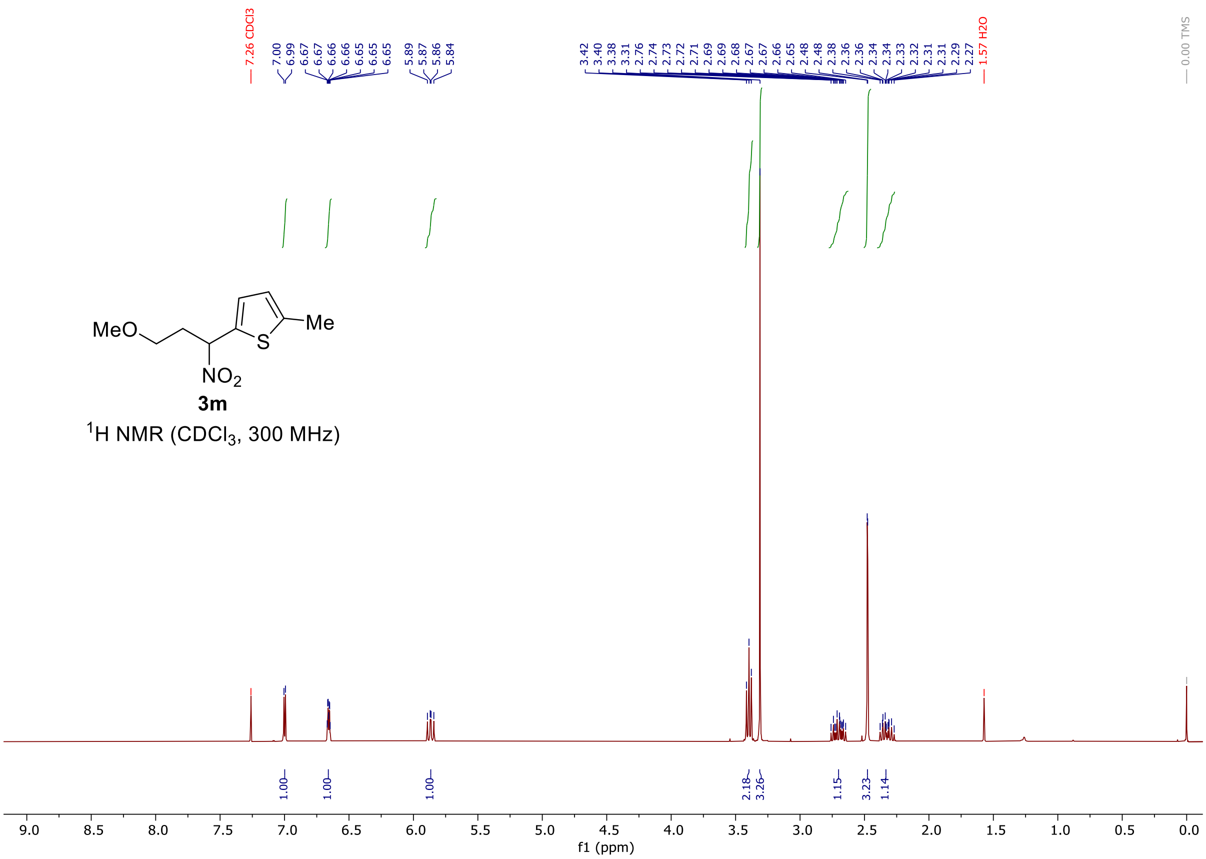

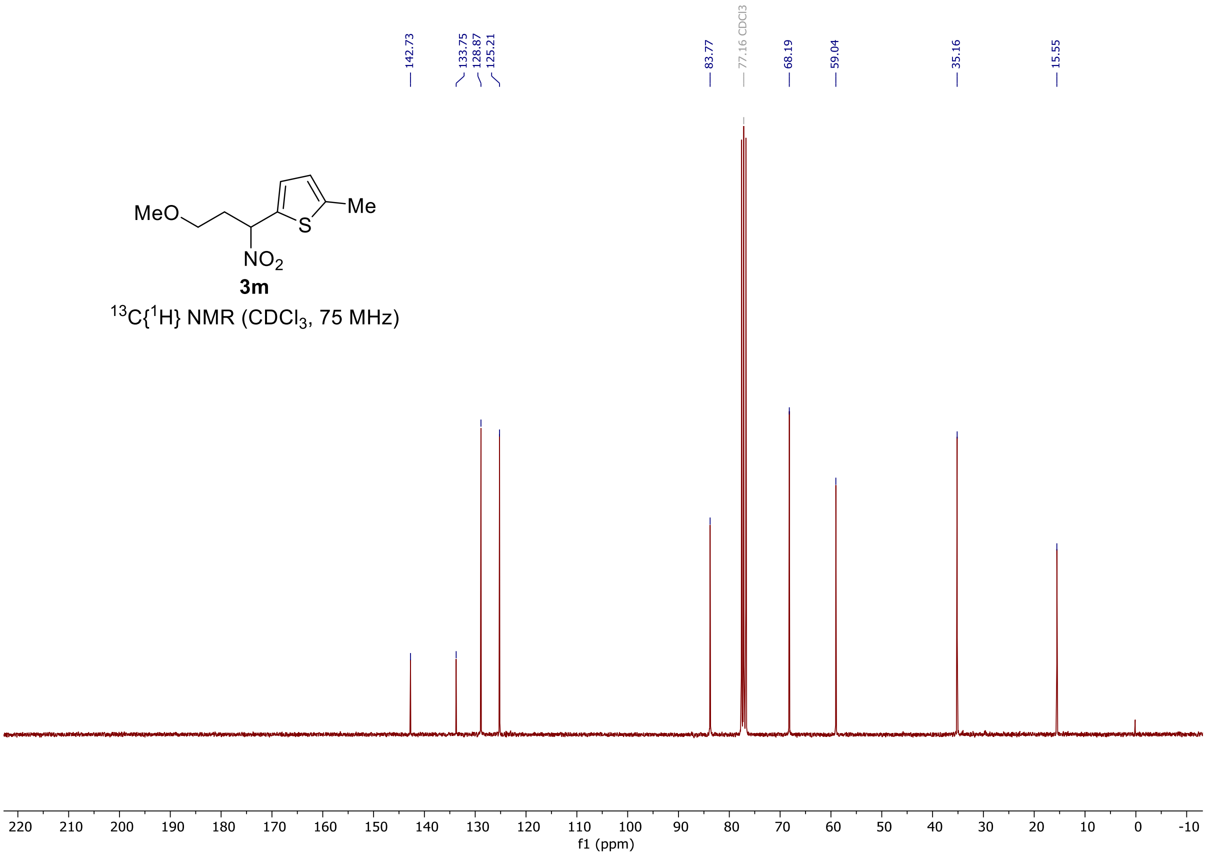

Figure S68

4.25 NMR spectra of 3n

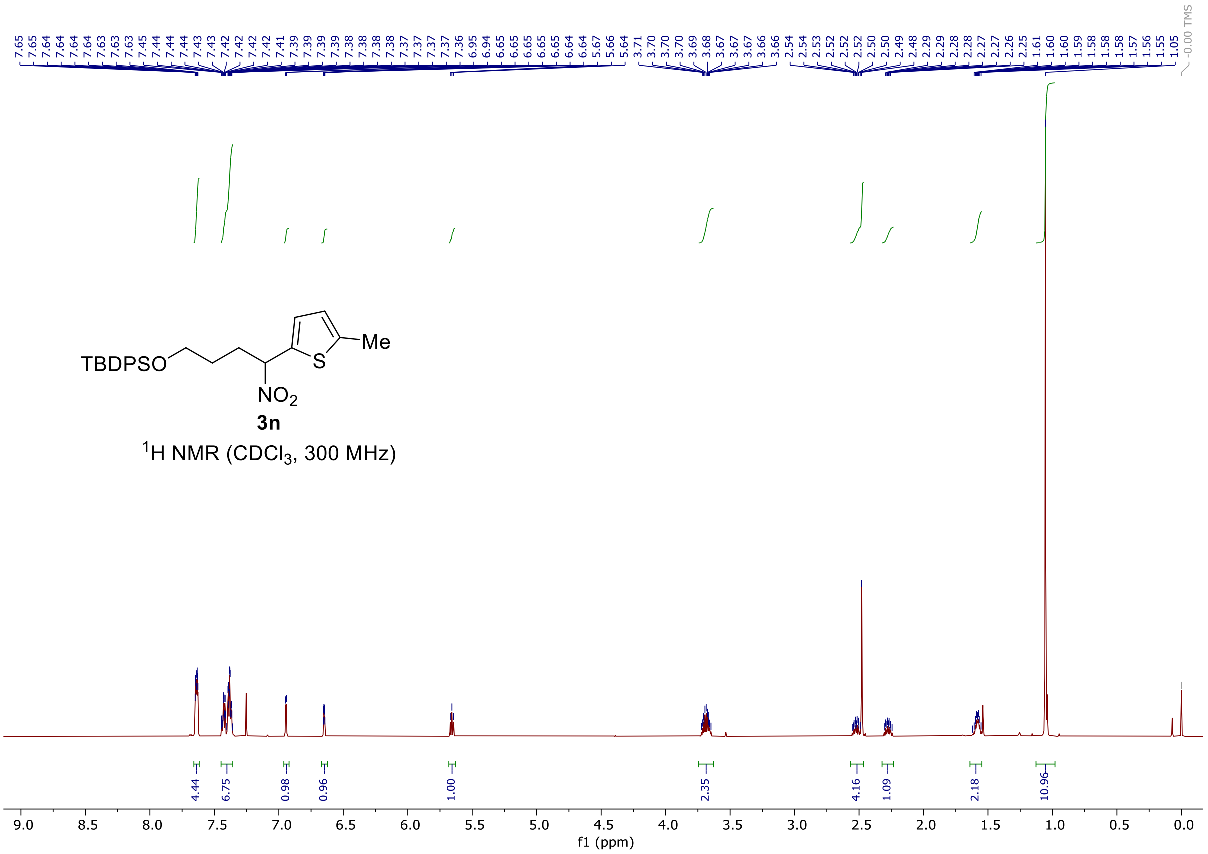

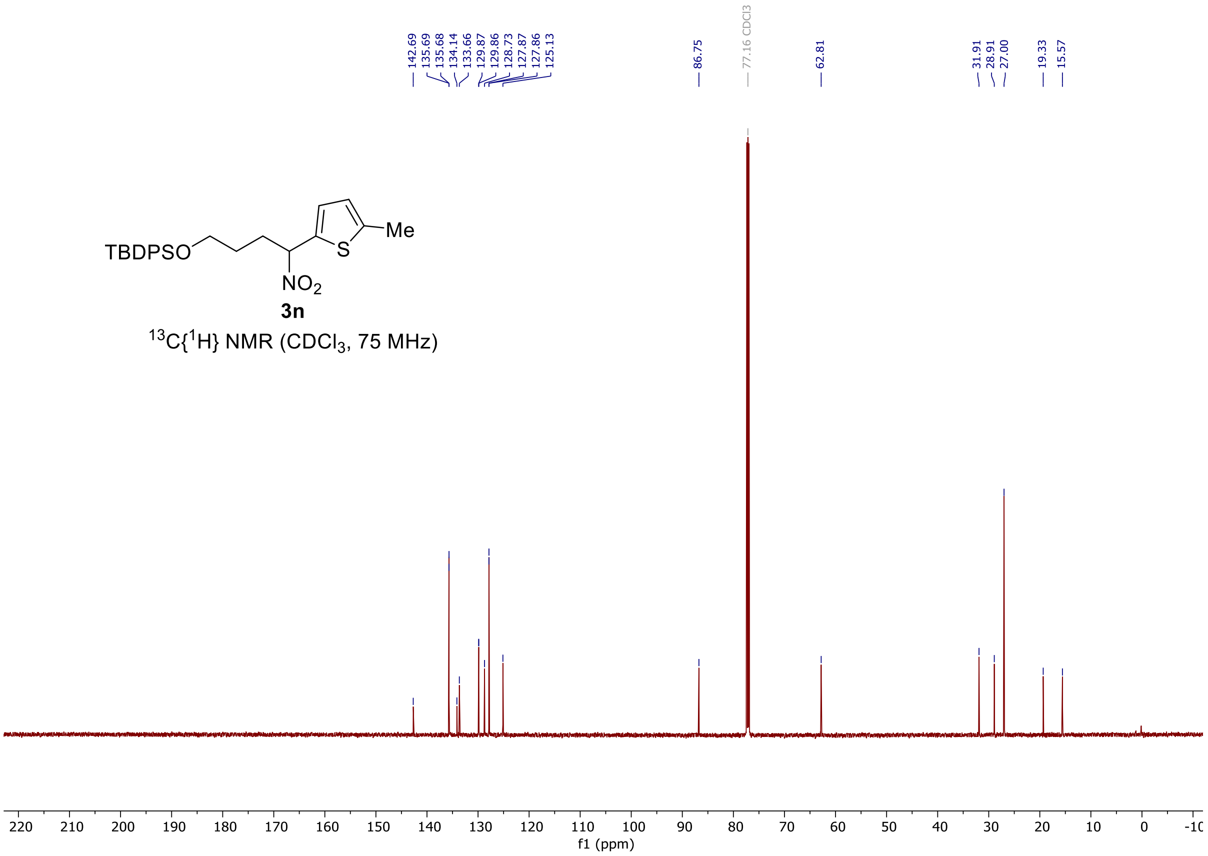

Figure S69

4.26 NMR spectra of 3o

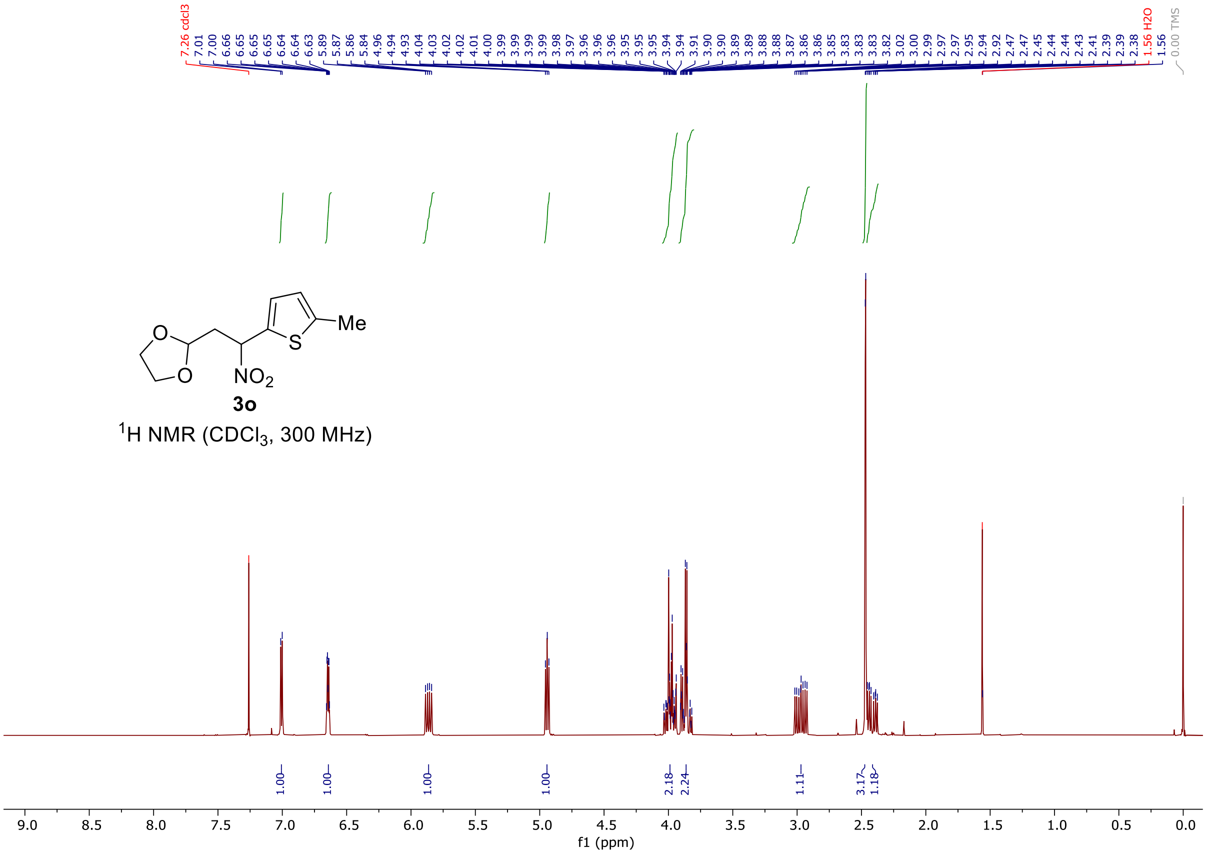

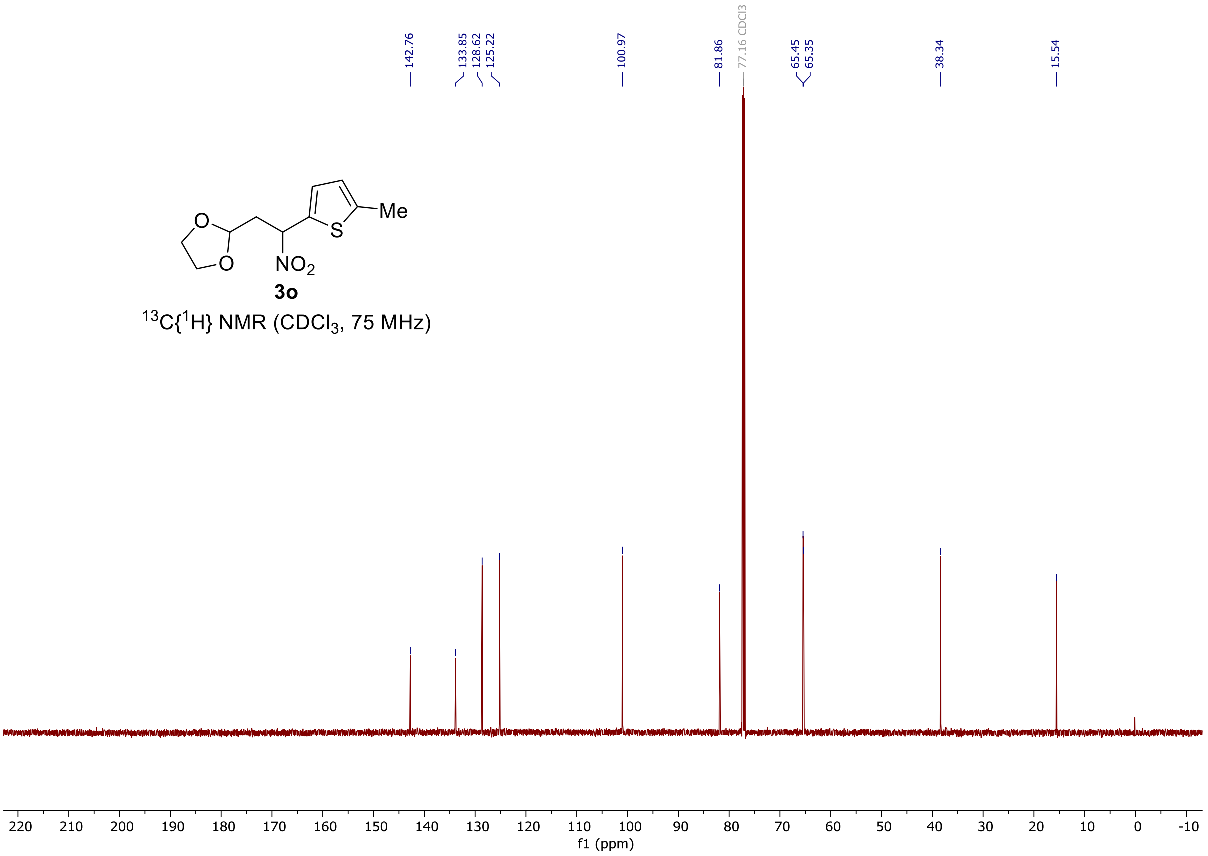

Figure S70

4.27 NMRspectra of 3p

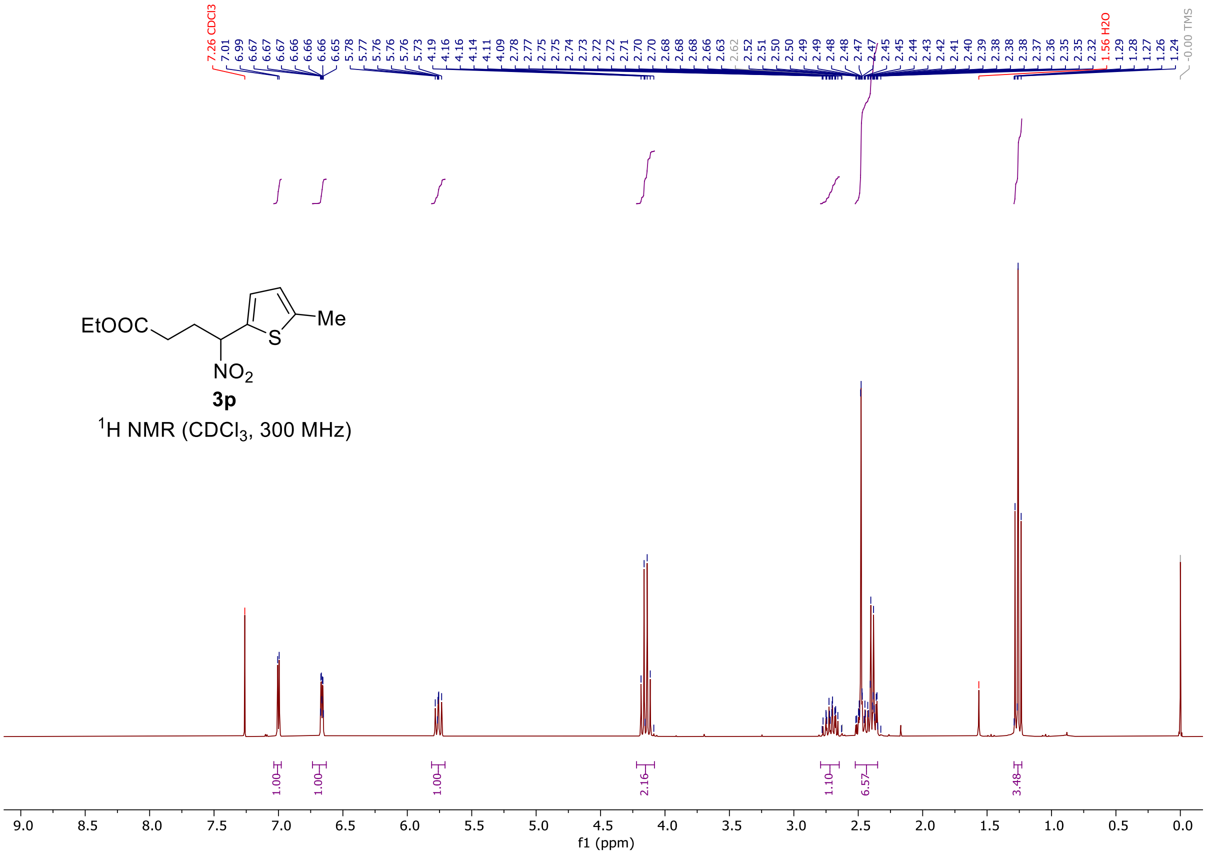

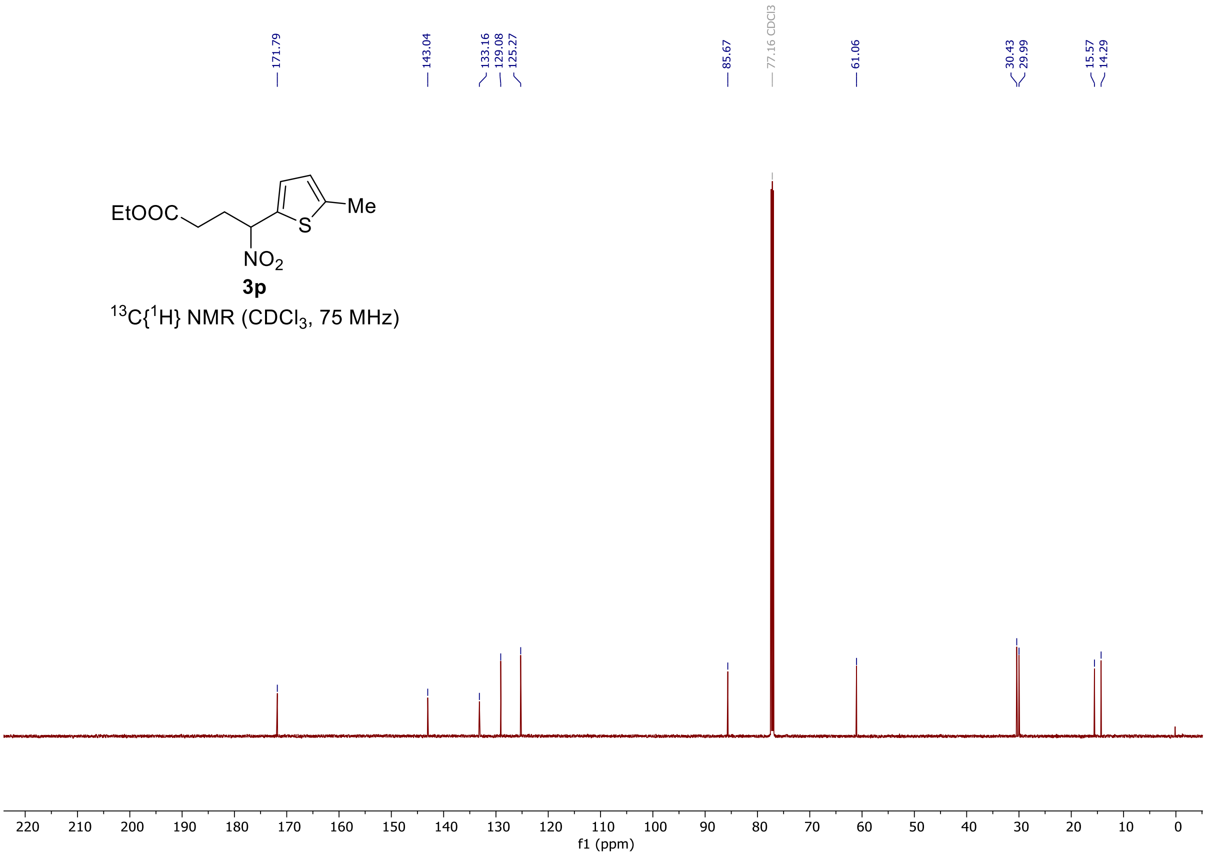

Figure S71

4.28 NMR spectra of 3q

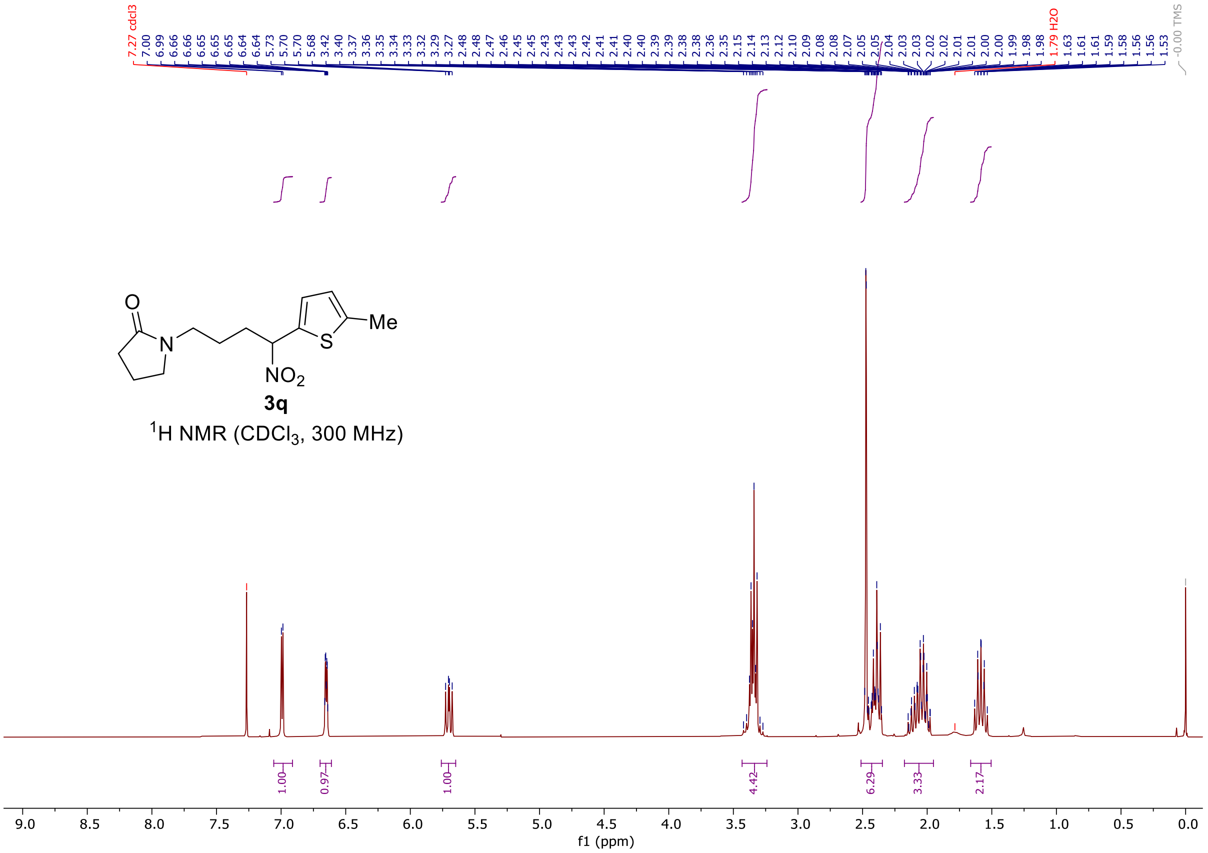

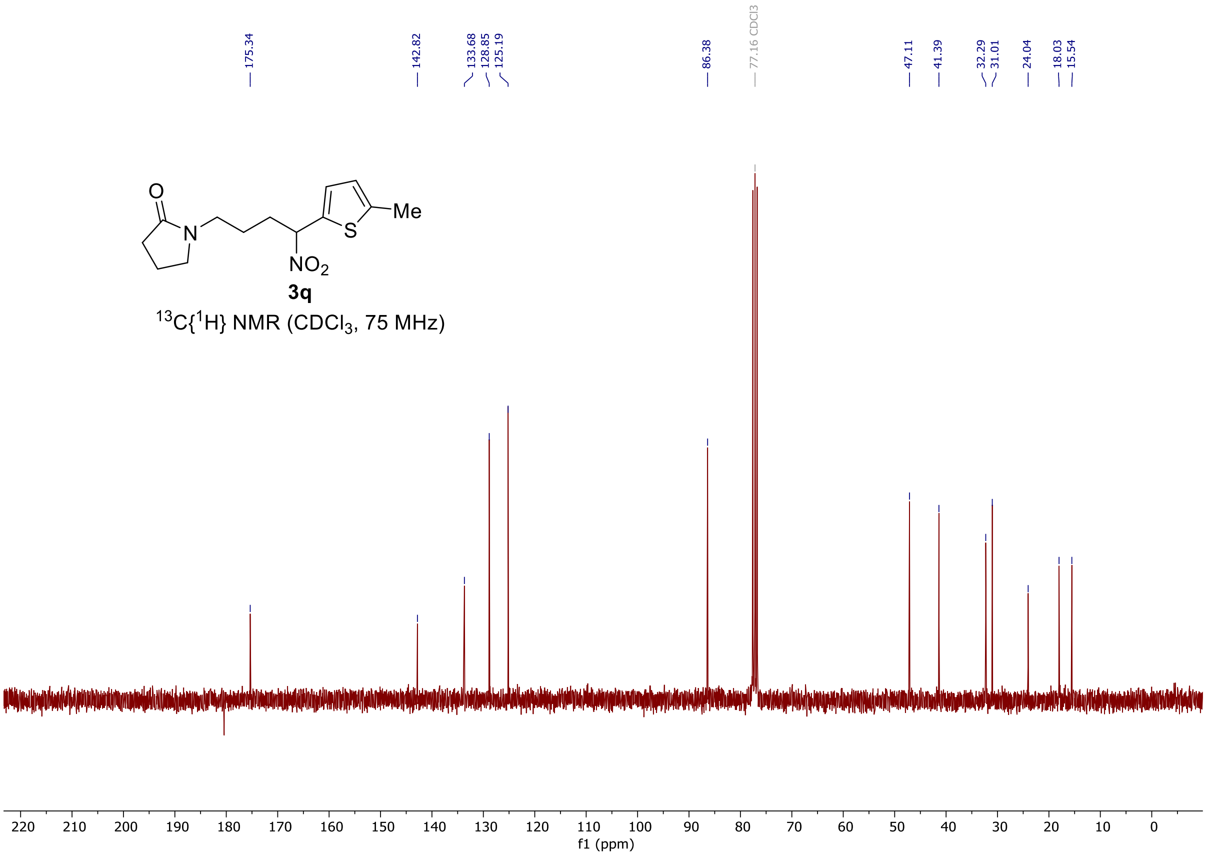

Figure S72

4.29 NMR spectra of 3r

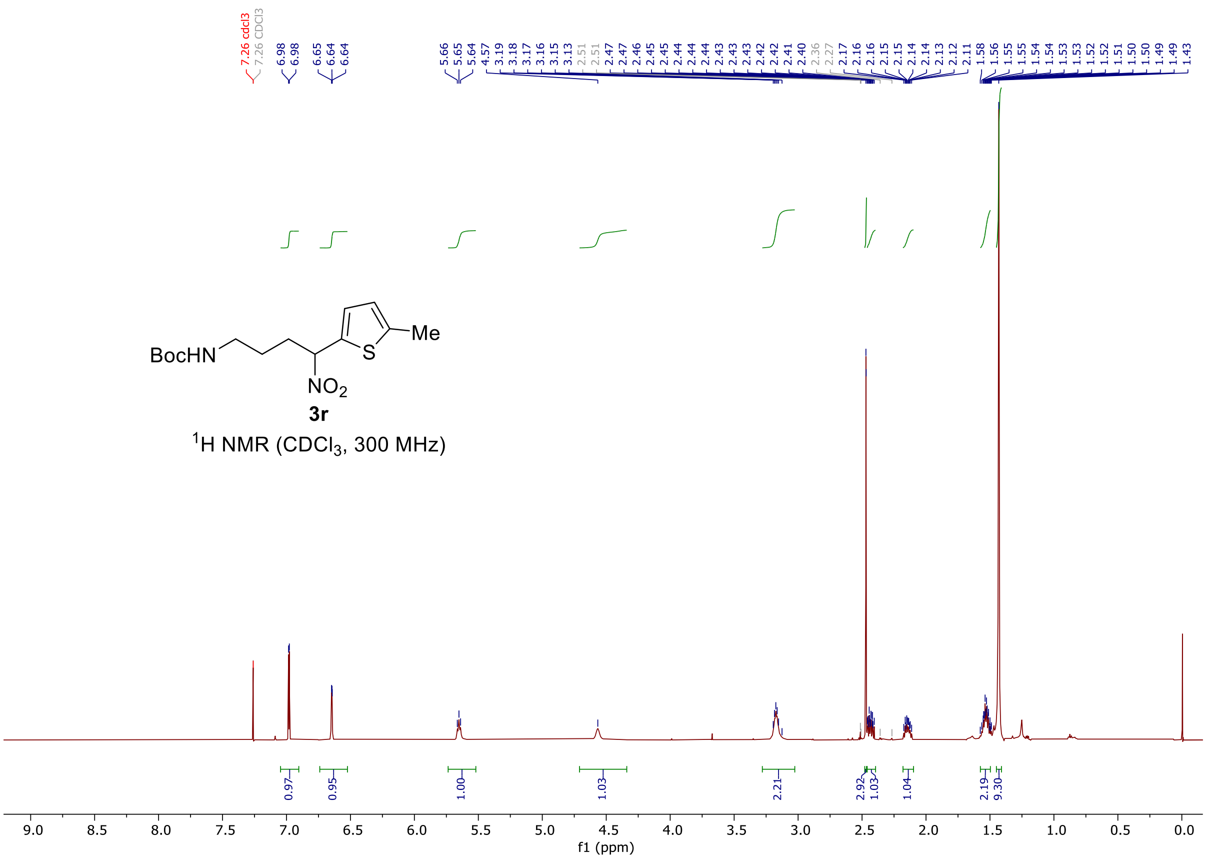

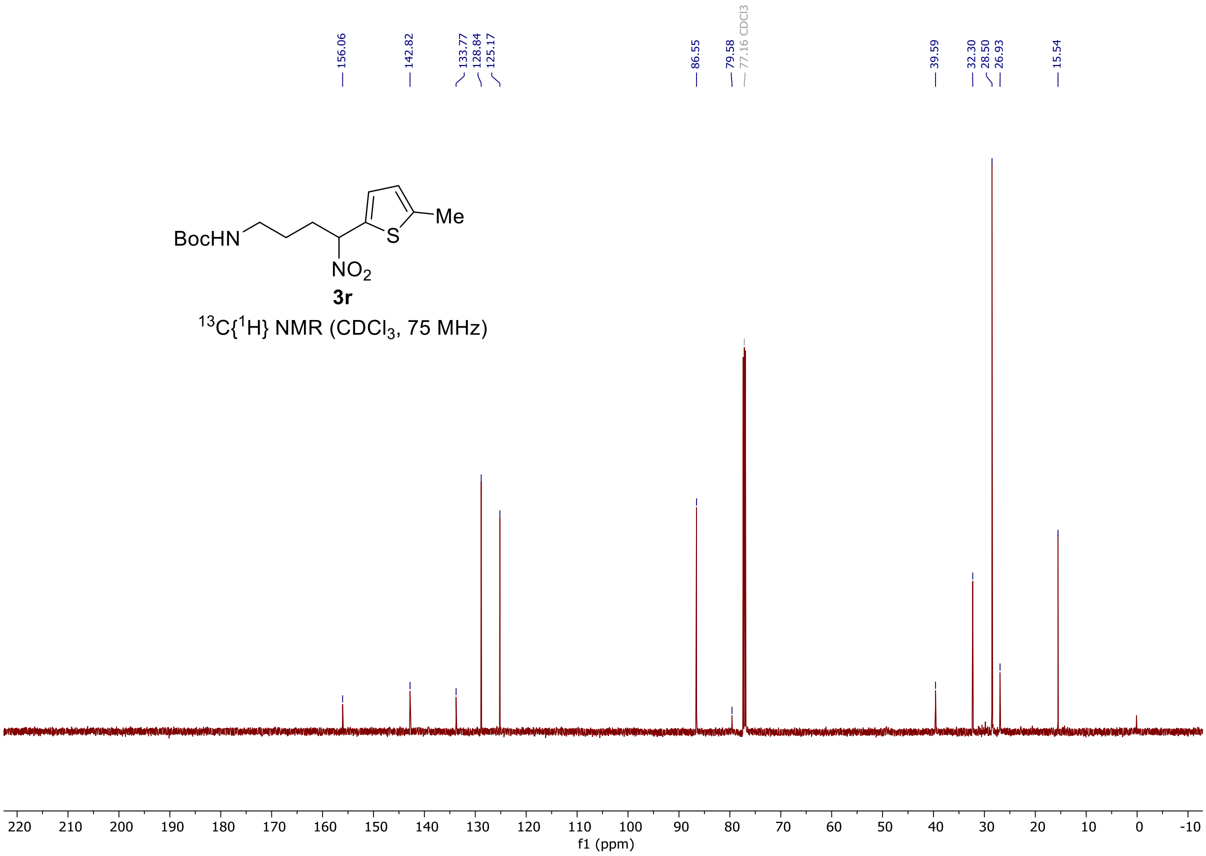

Figure S73

4.30 NMR spectra of 3s

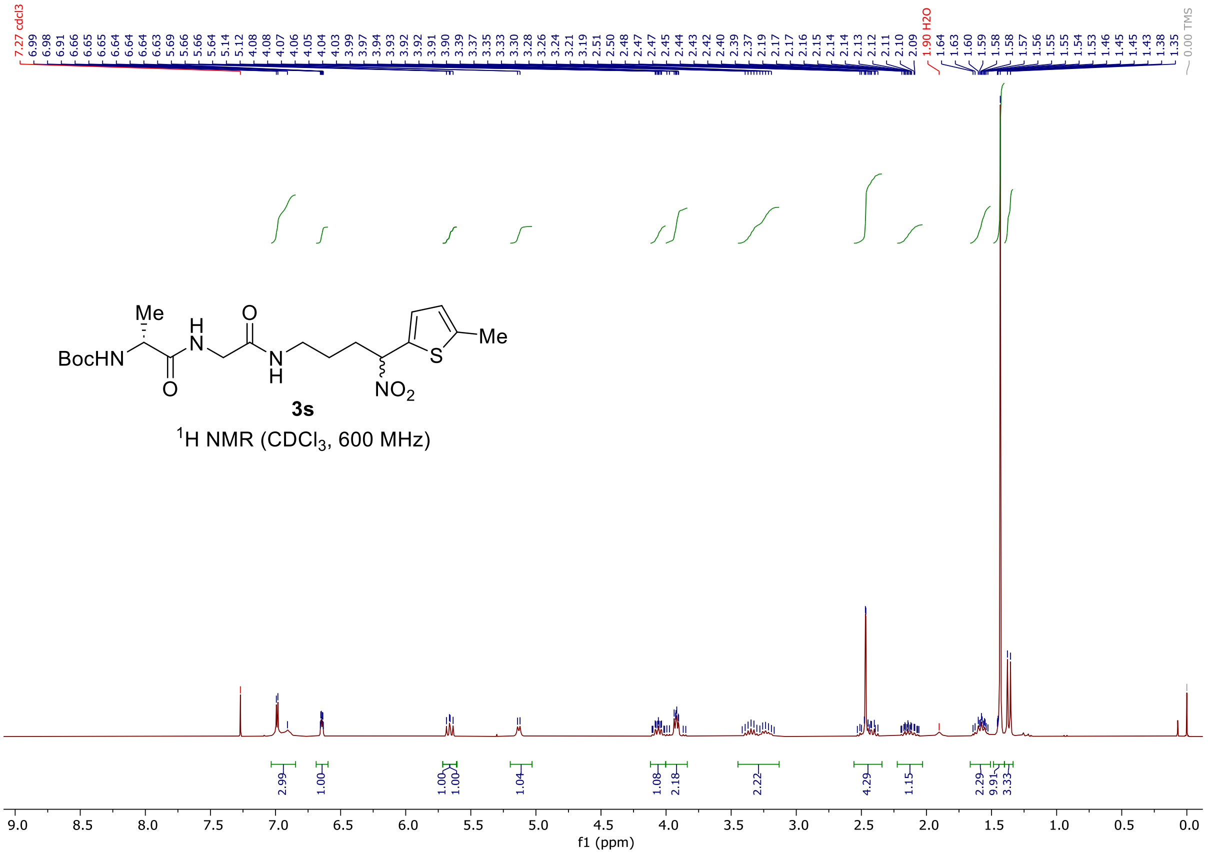

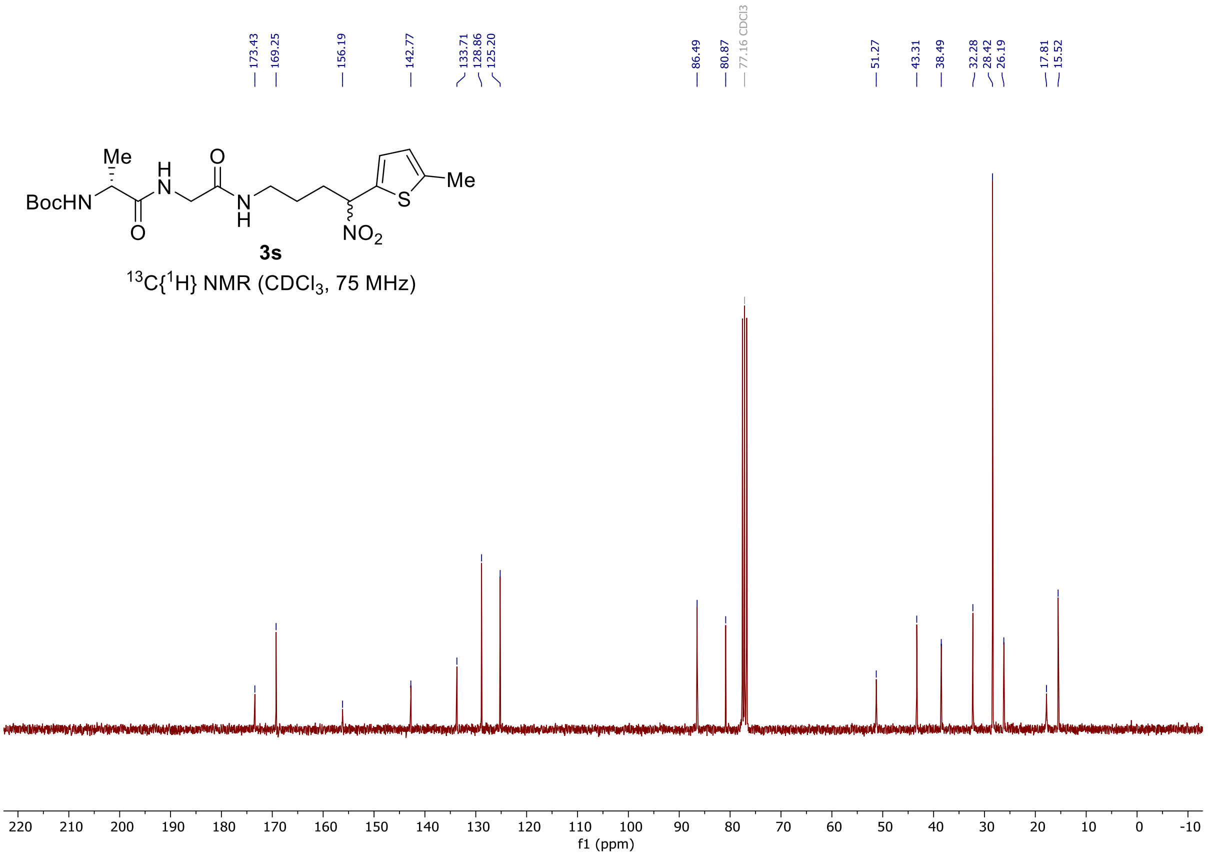

Figure S74

4.31 NMR spectra of 3t

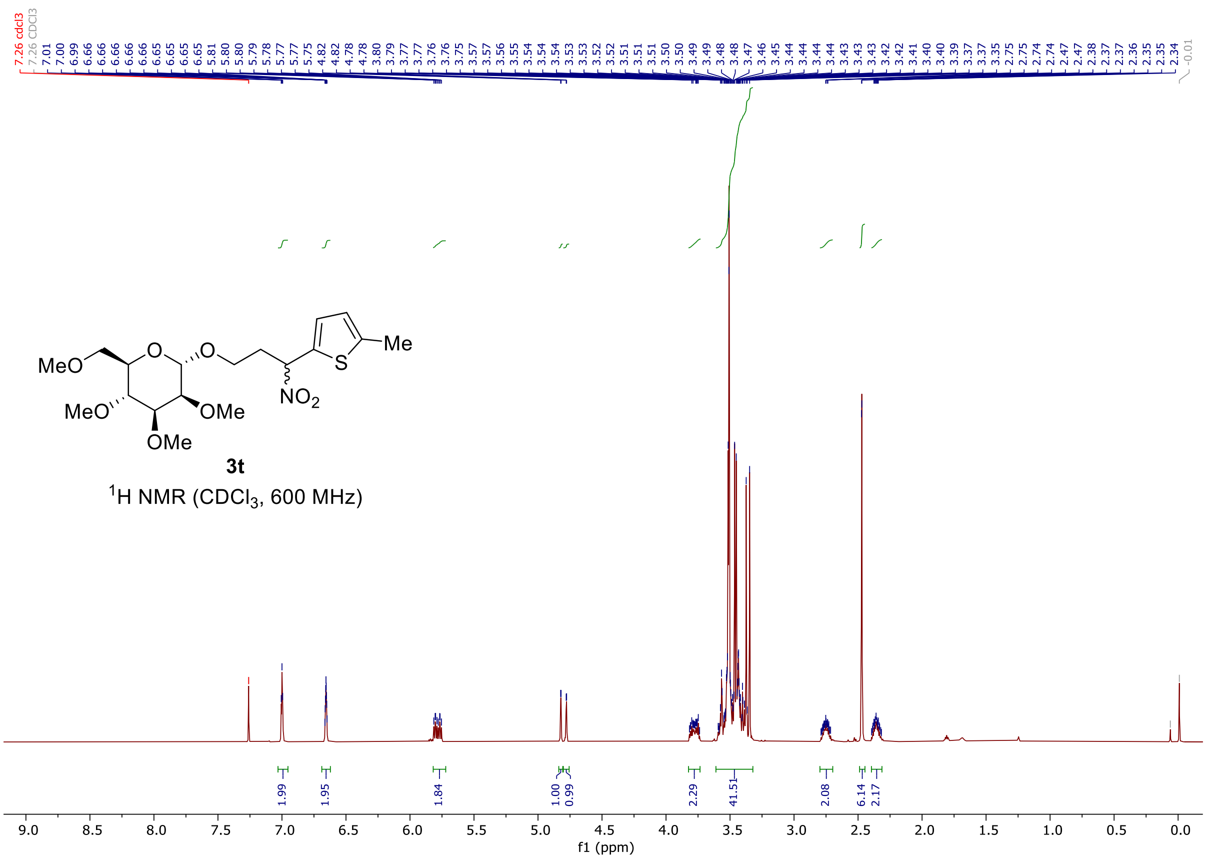

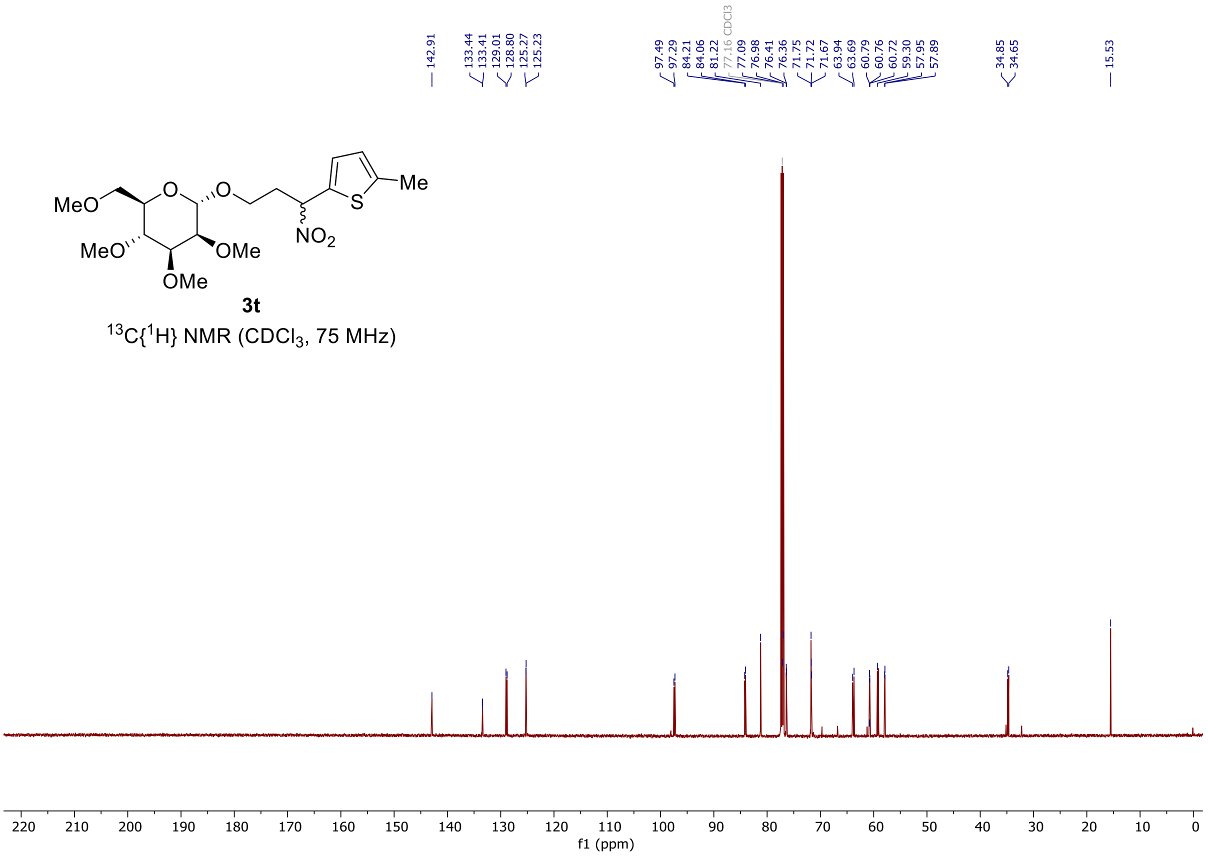

Figure S75

4.32 NMR spectra of 3u

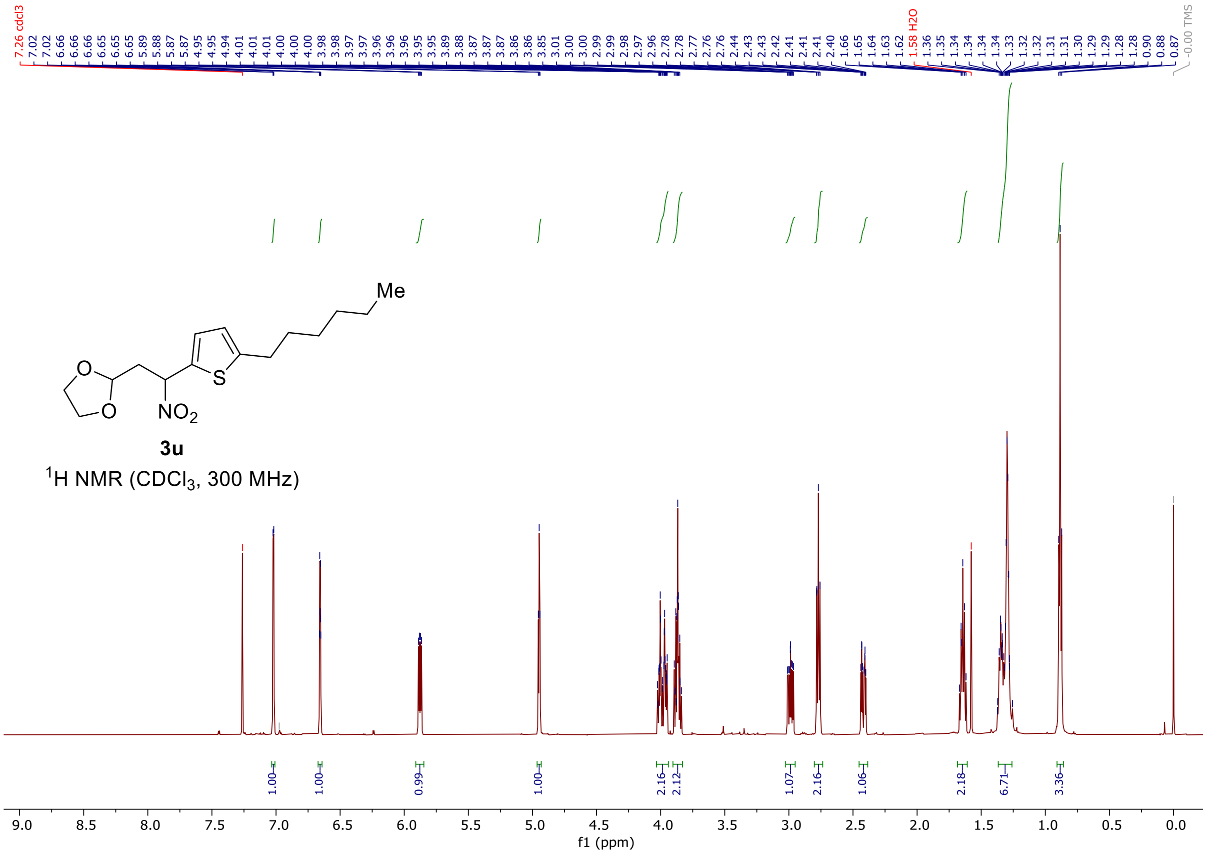

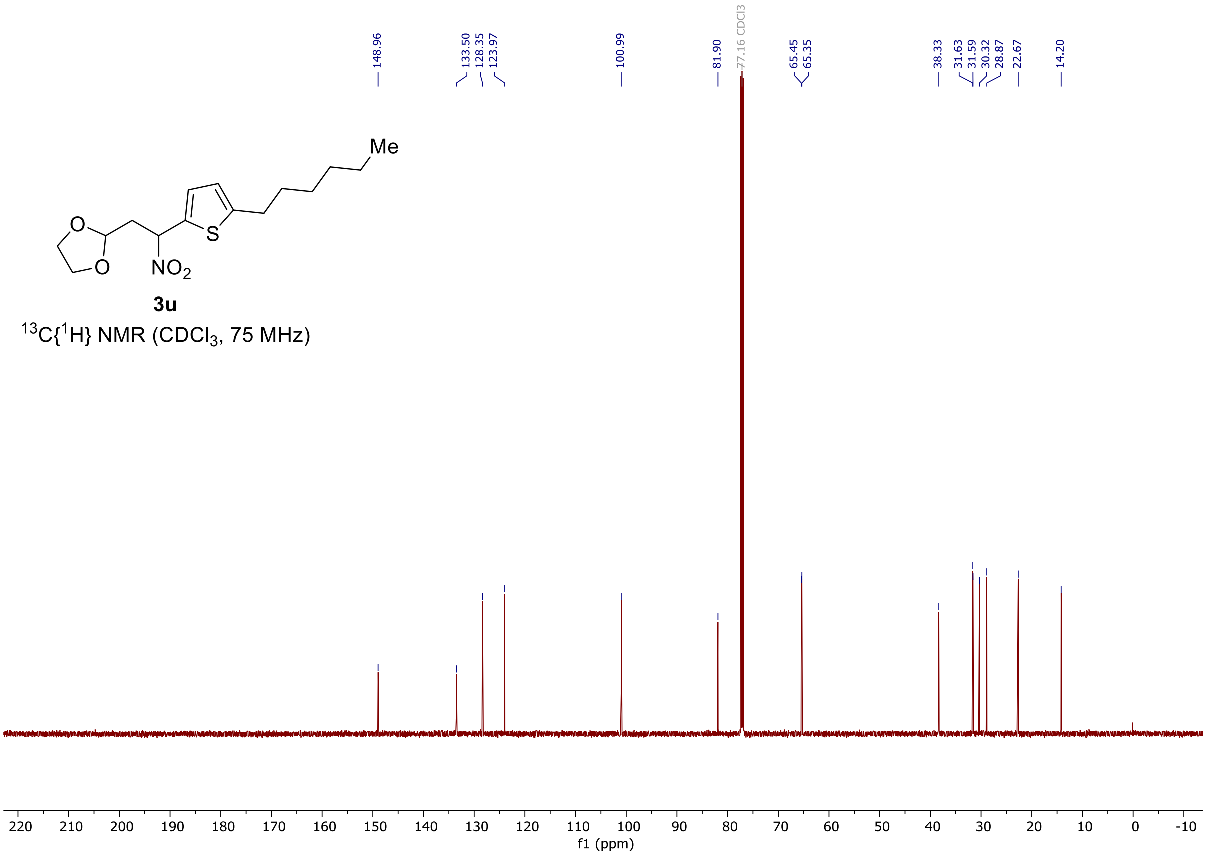

Figure S76

4.33 NMR spectra of 3v

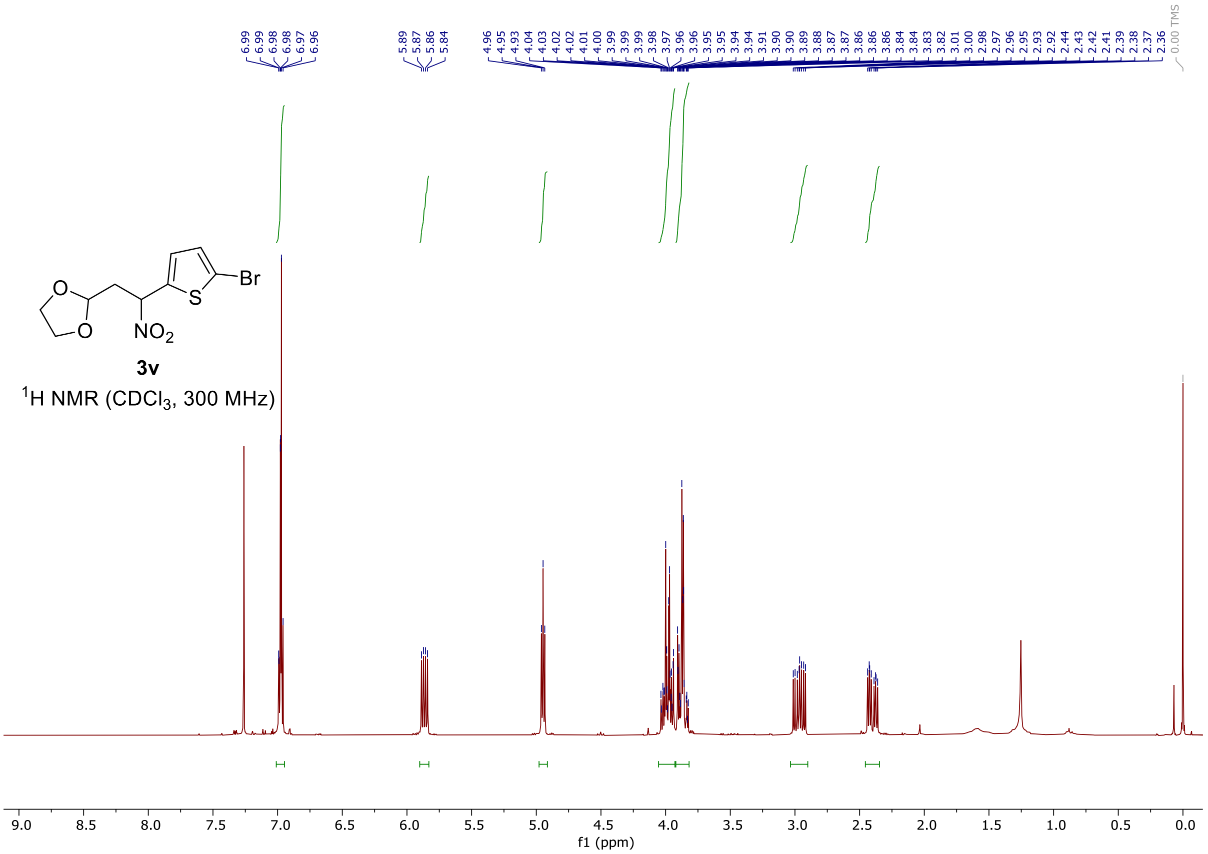

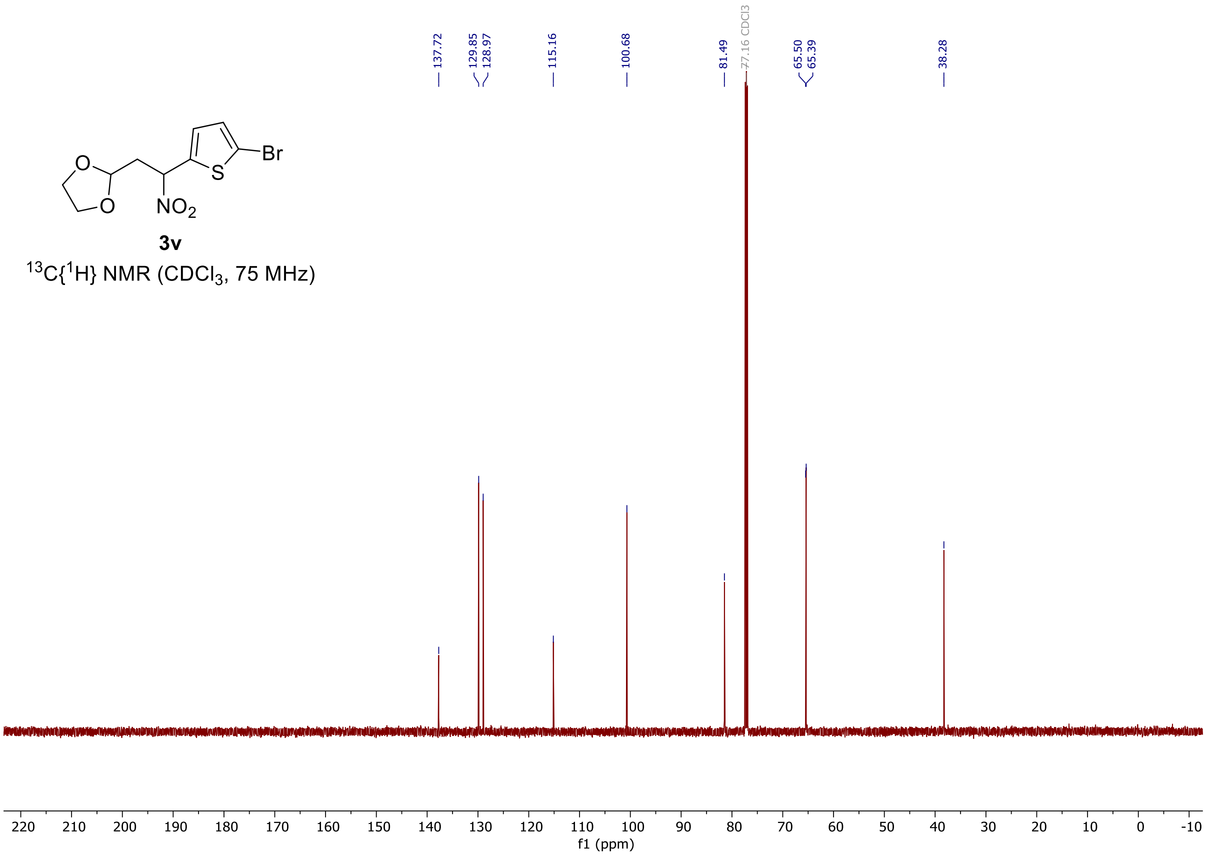

Figure S77

4.34 NMR spectra of 3w

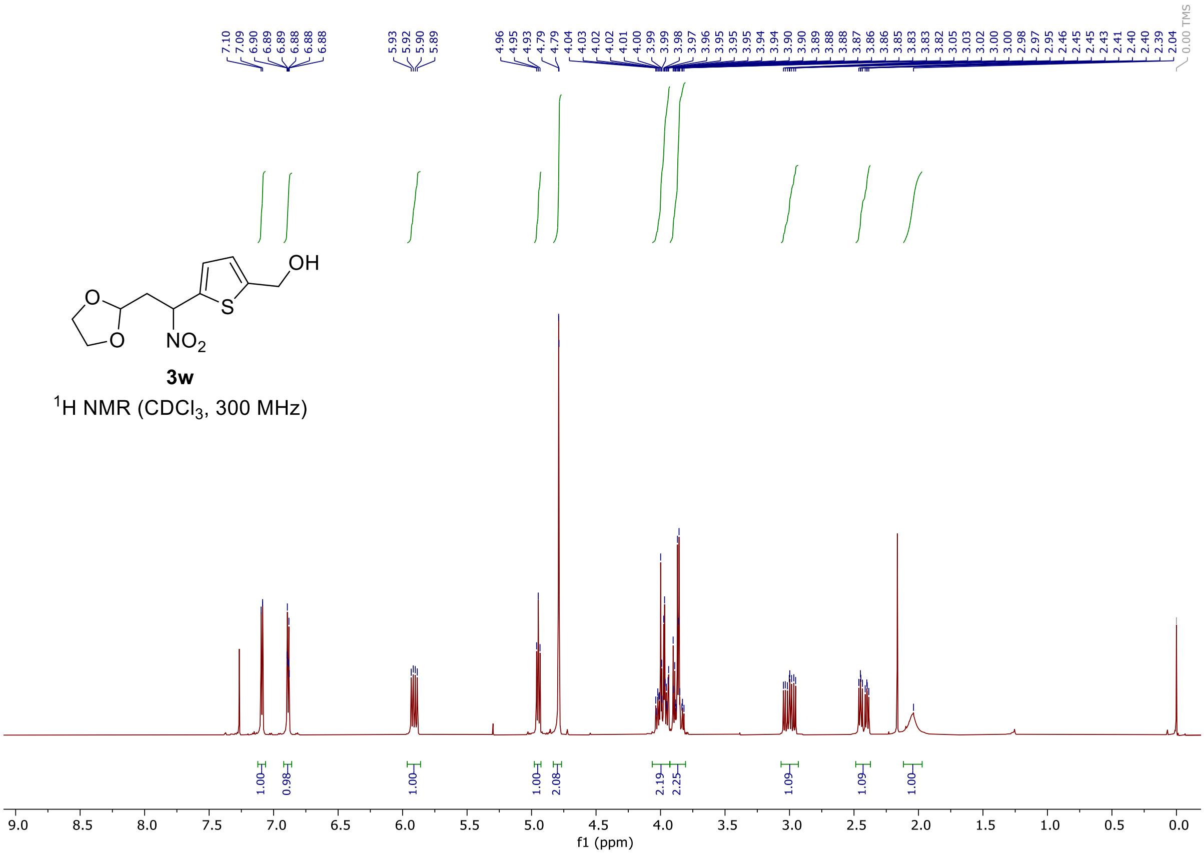

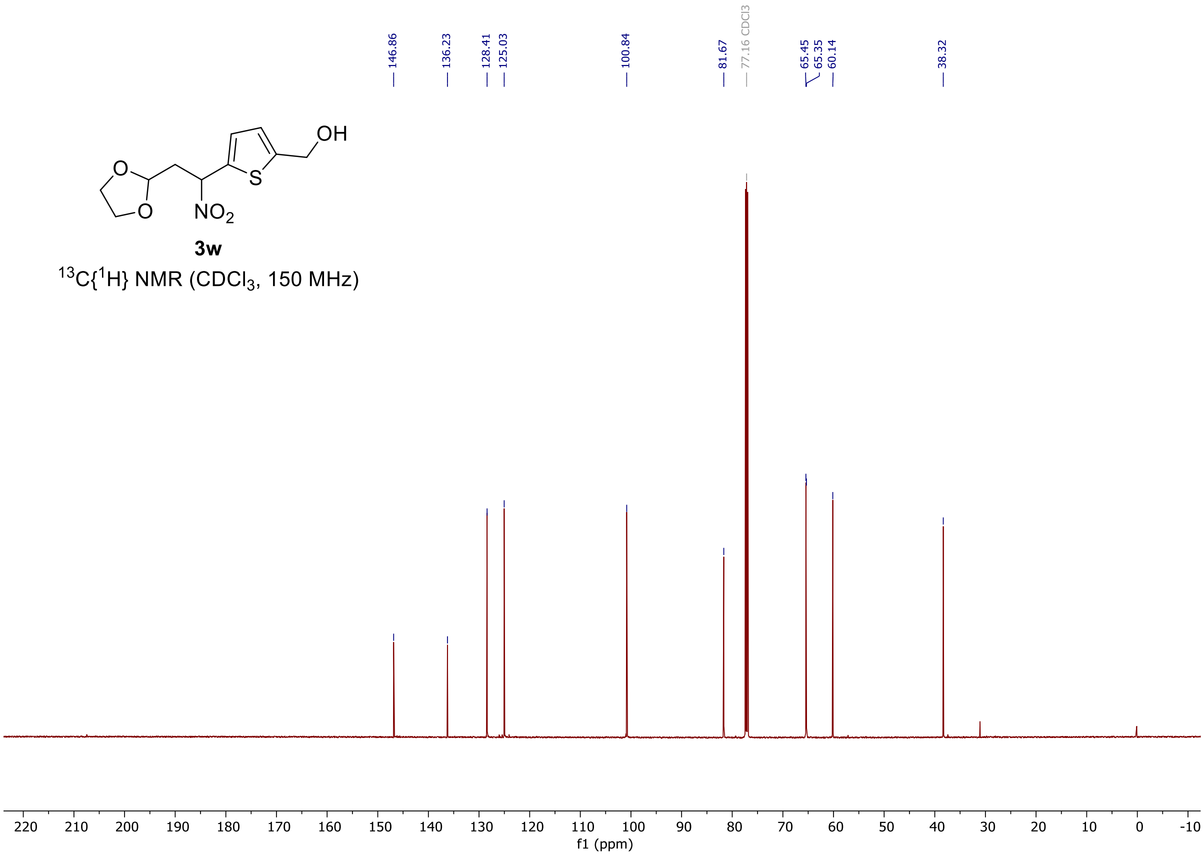

Figure S78

4.35 NMR spectra of 3x

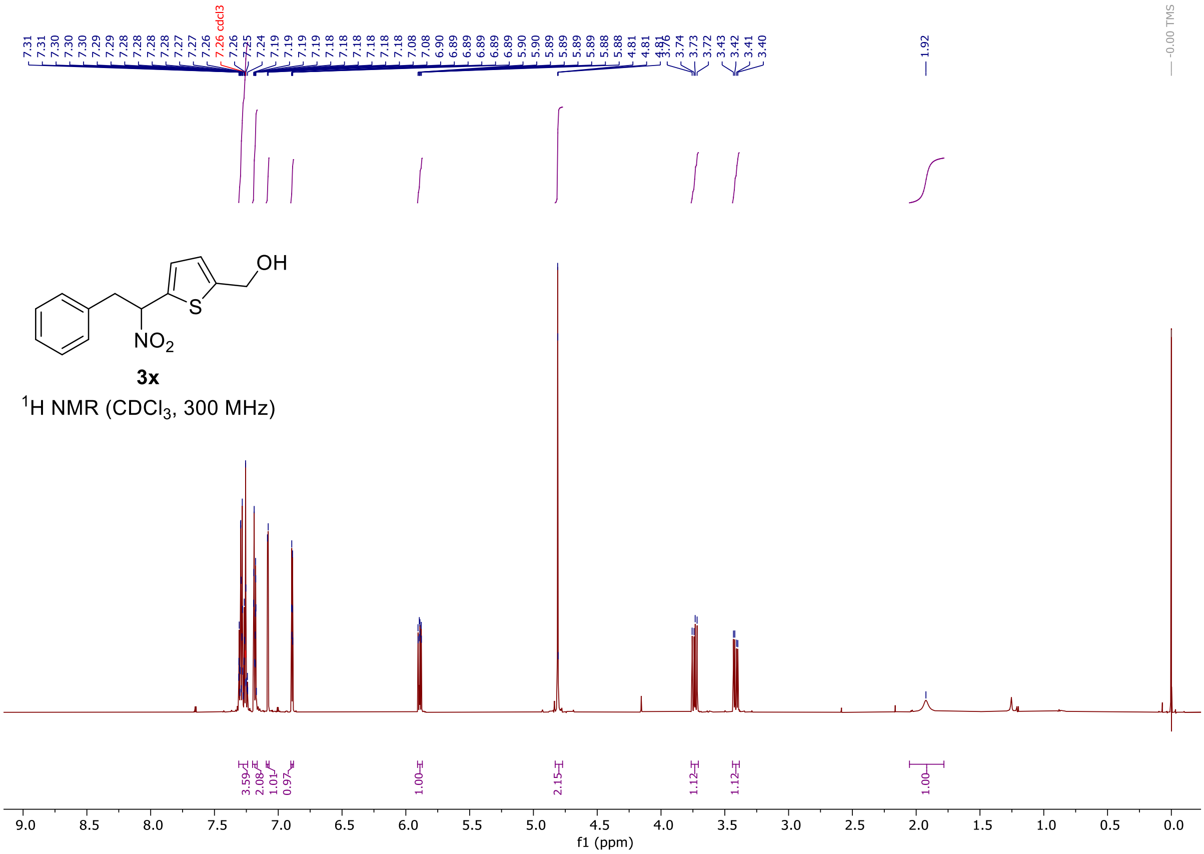

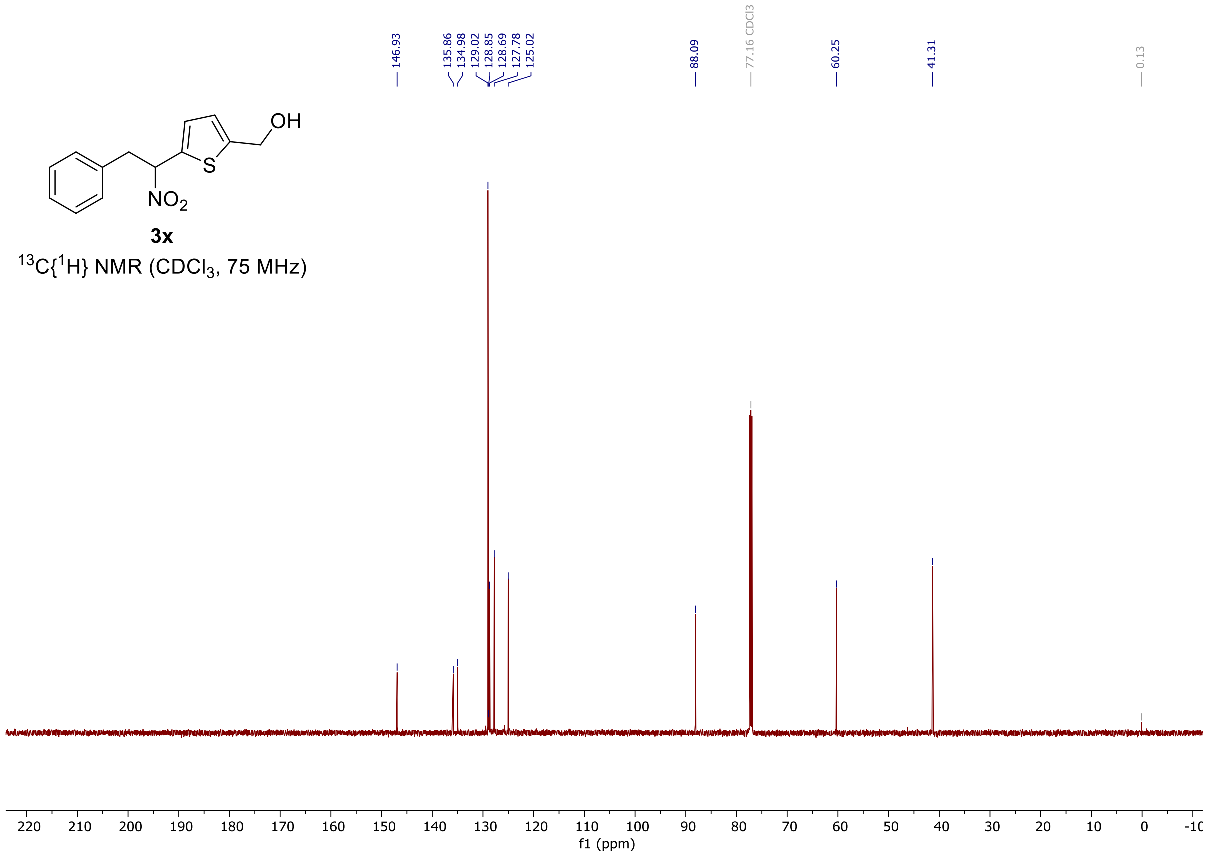

Figure S79

4.36 NMR spectra of 3y and 3y´

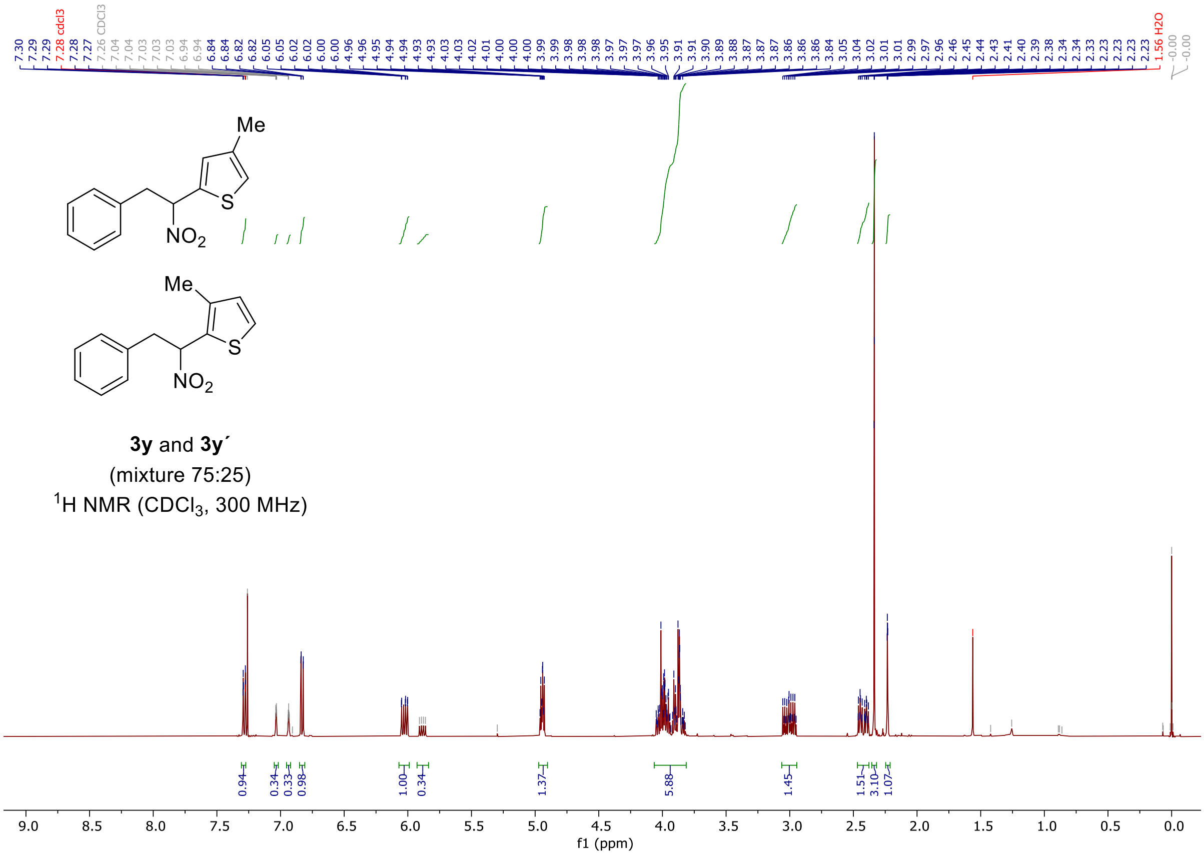

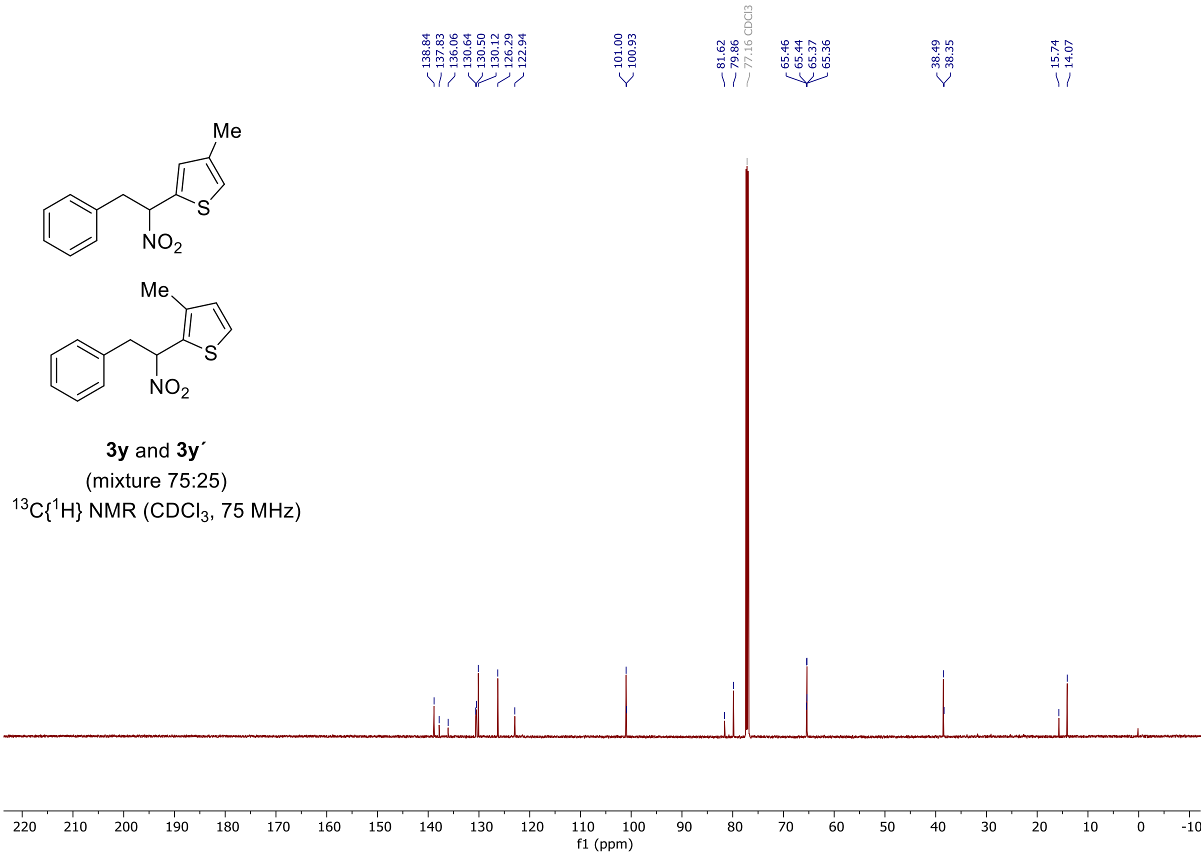

Figure S80

4.37 NMR spectra of 3z

3z

^1H NMR ($CDCl_3$, 300 MHz)

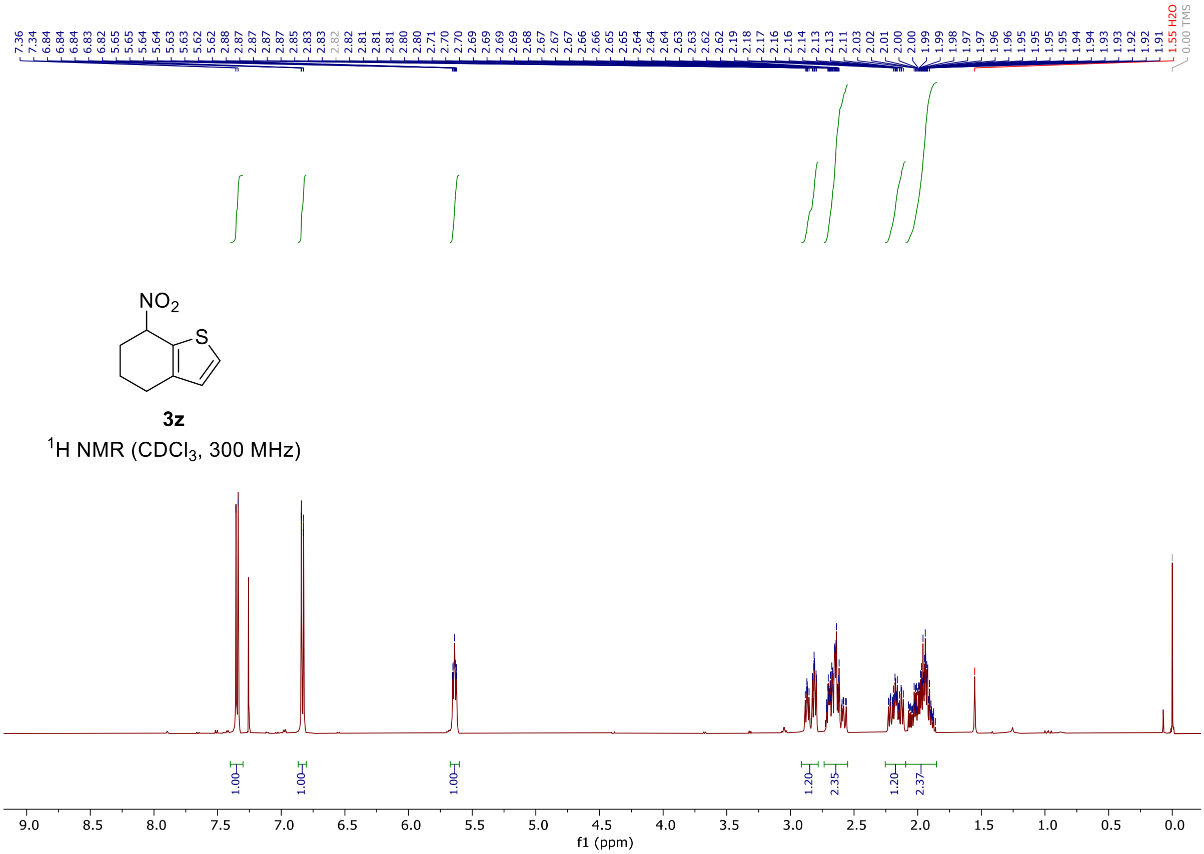

3z

$^{13}C\{^1H\}$ NMR ($CDCl_3$, 75 MHz)

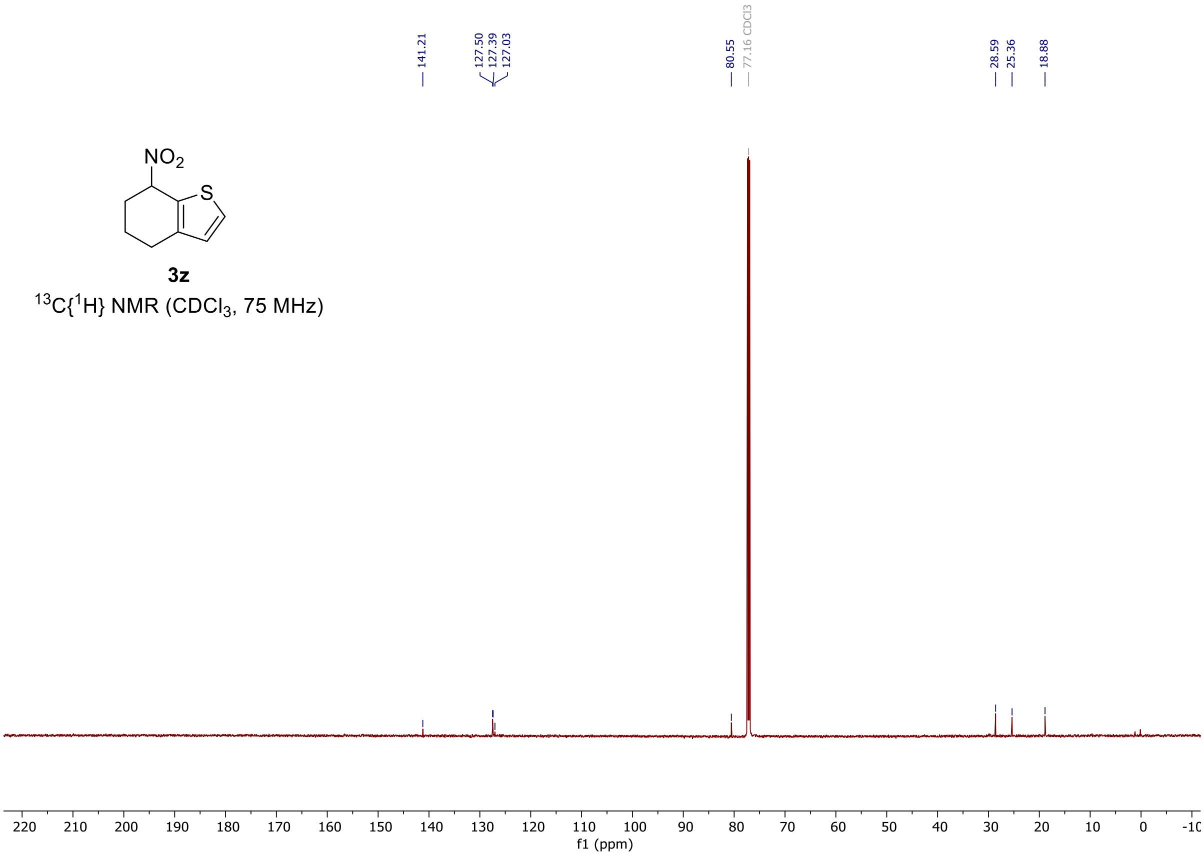

Figure S81

4.38 NMR spectra of 3aa

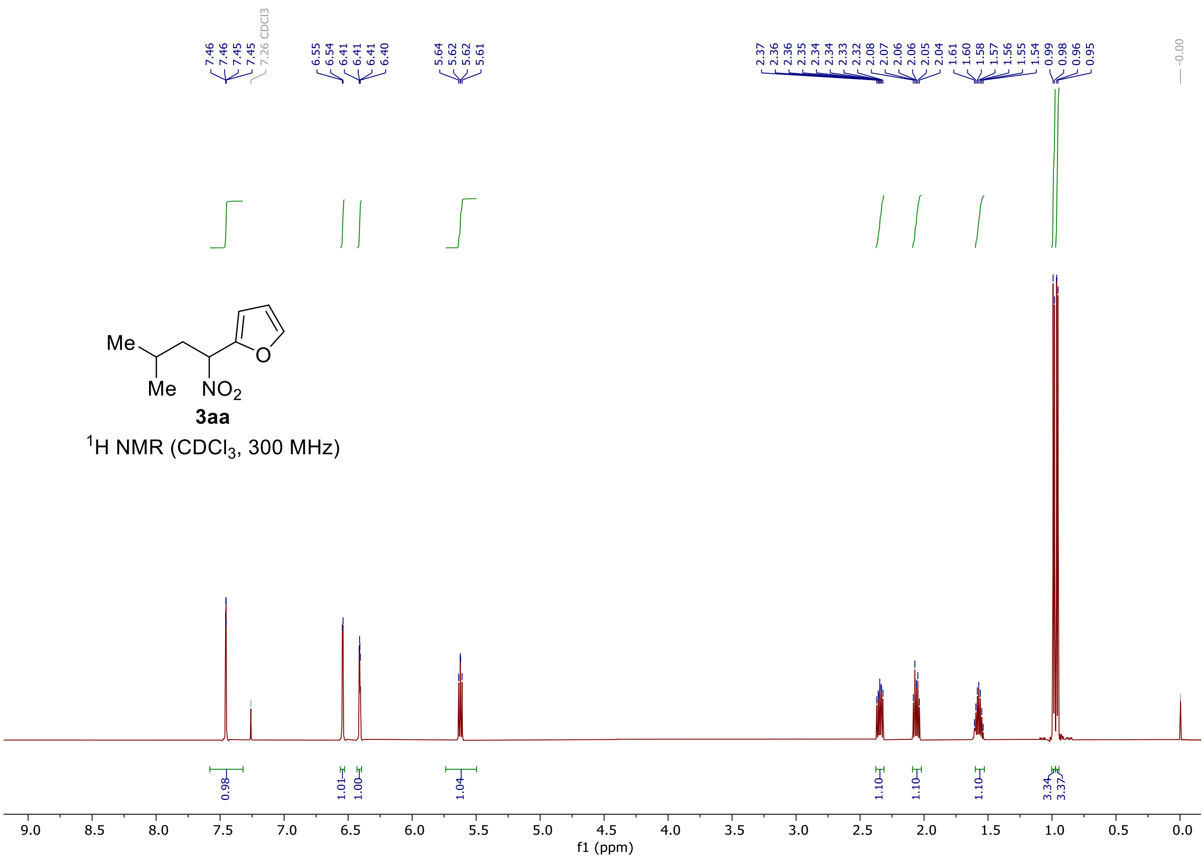

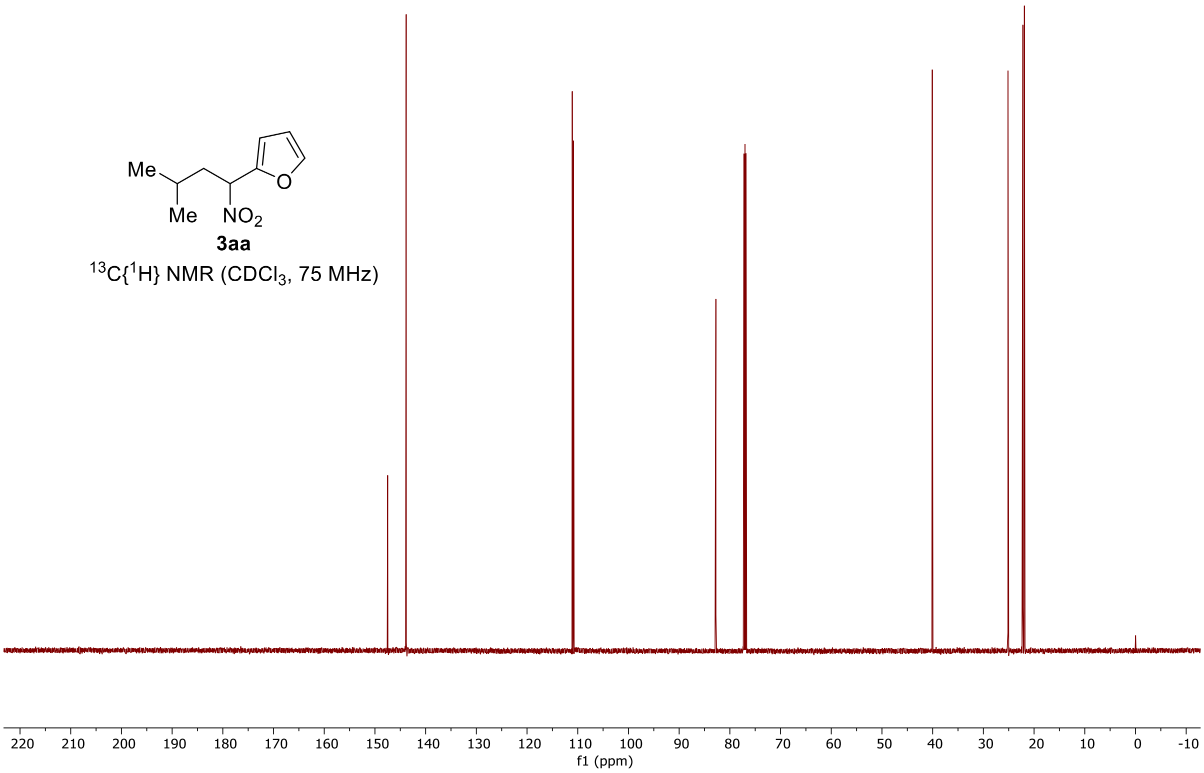

Figure S82

4.39 NMR spectra of 3ab

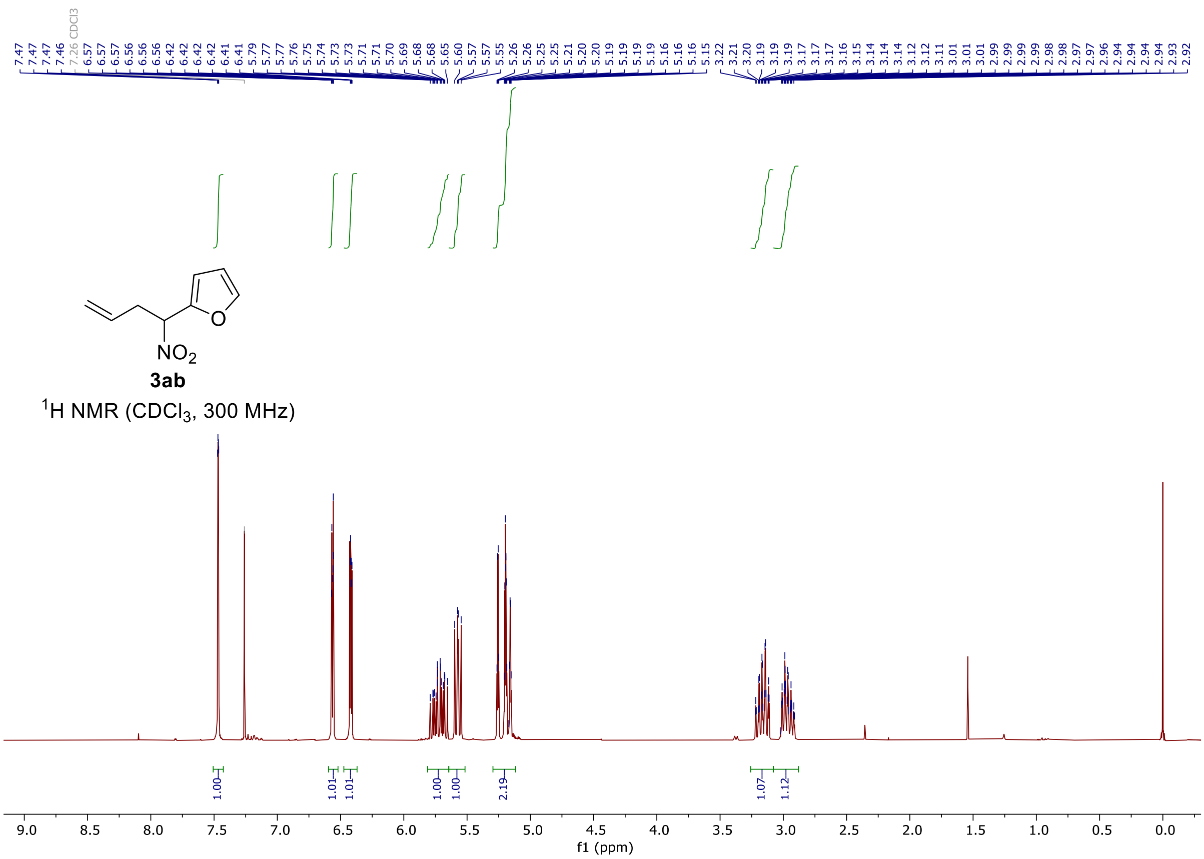

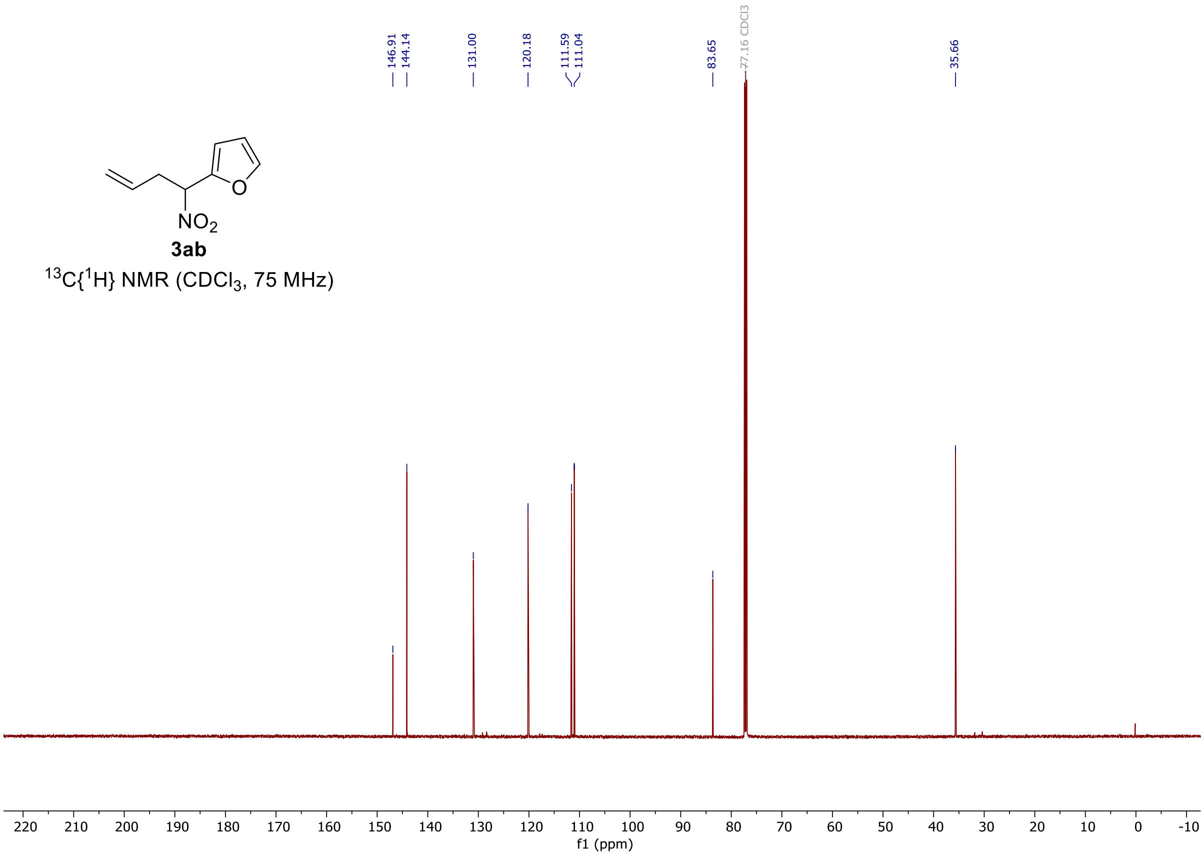

Figure S83

4.40 NMR spectra of 3ac

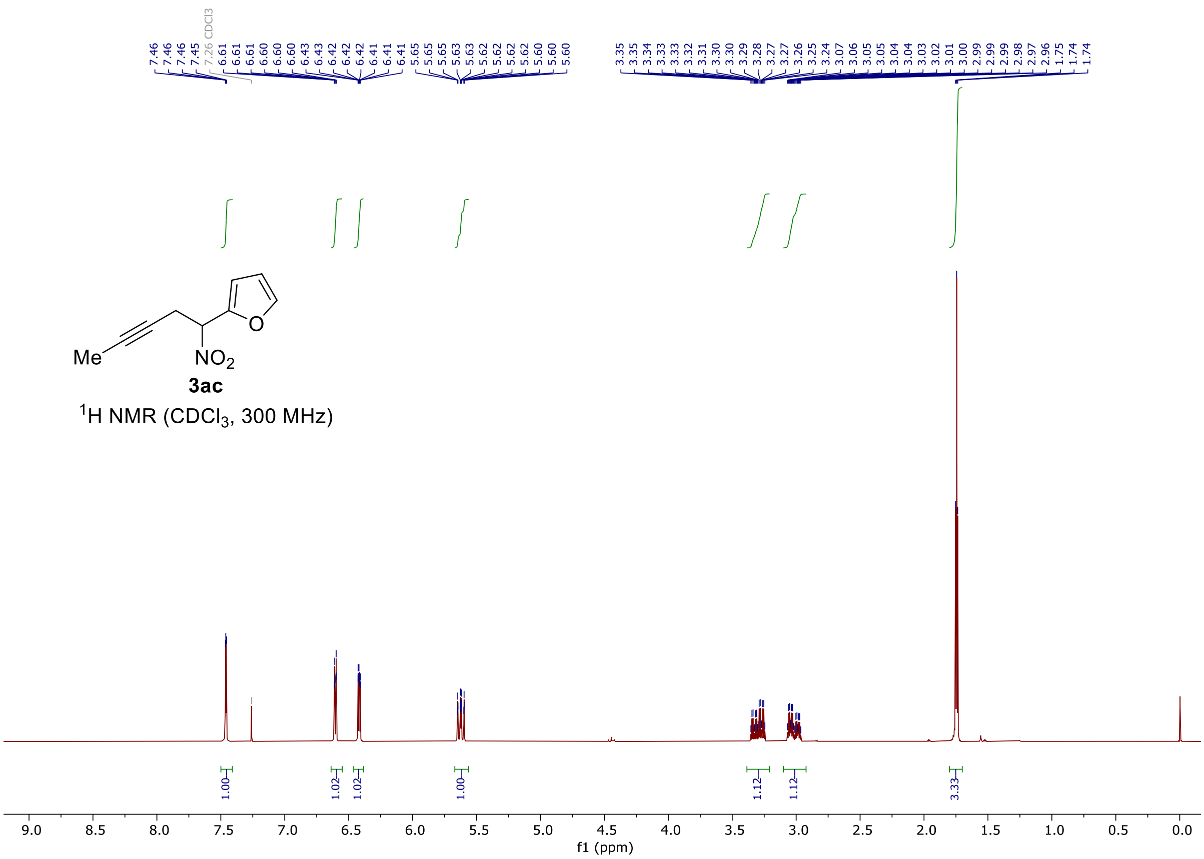

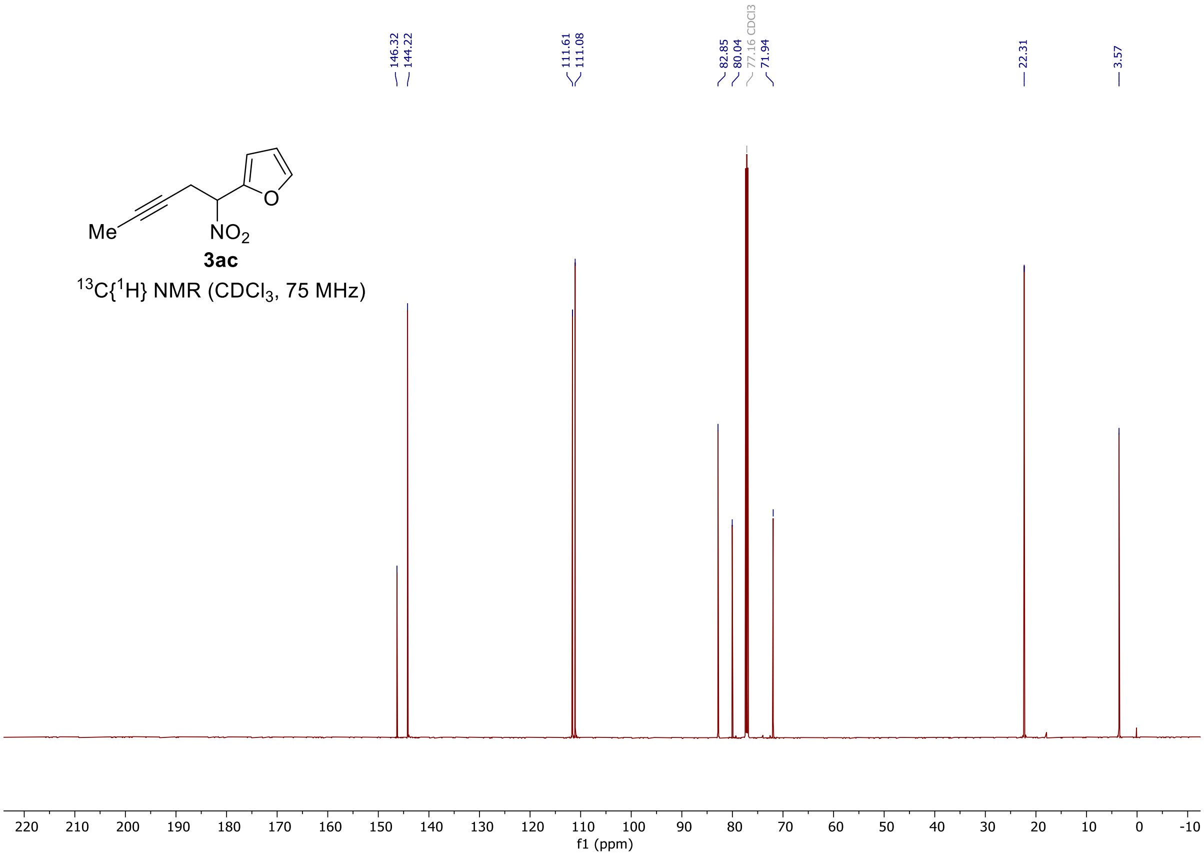

Figure S84

4.41 NMR spectra of 3ad

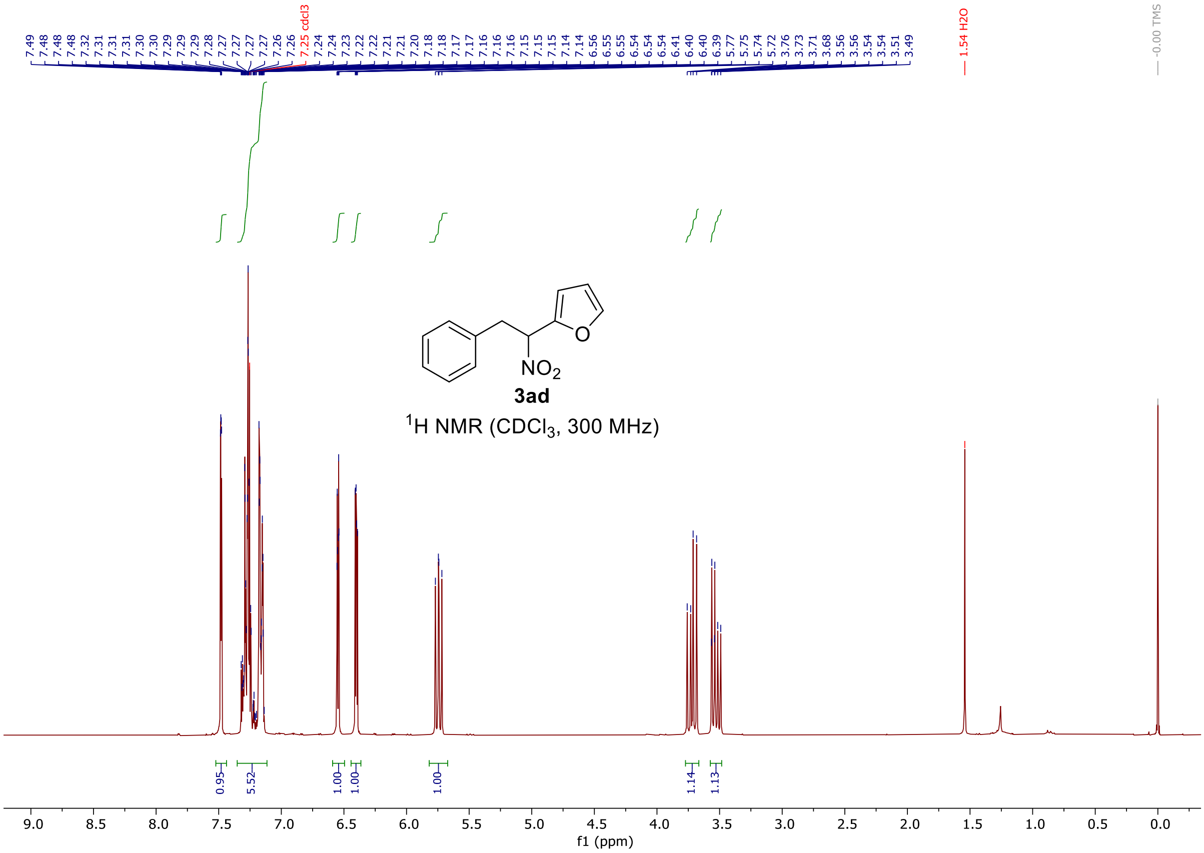

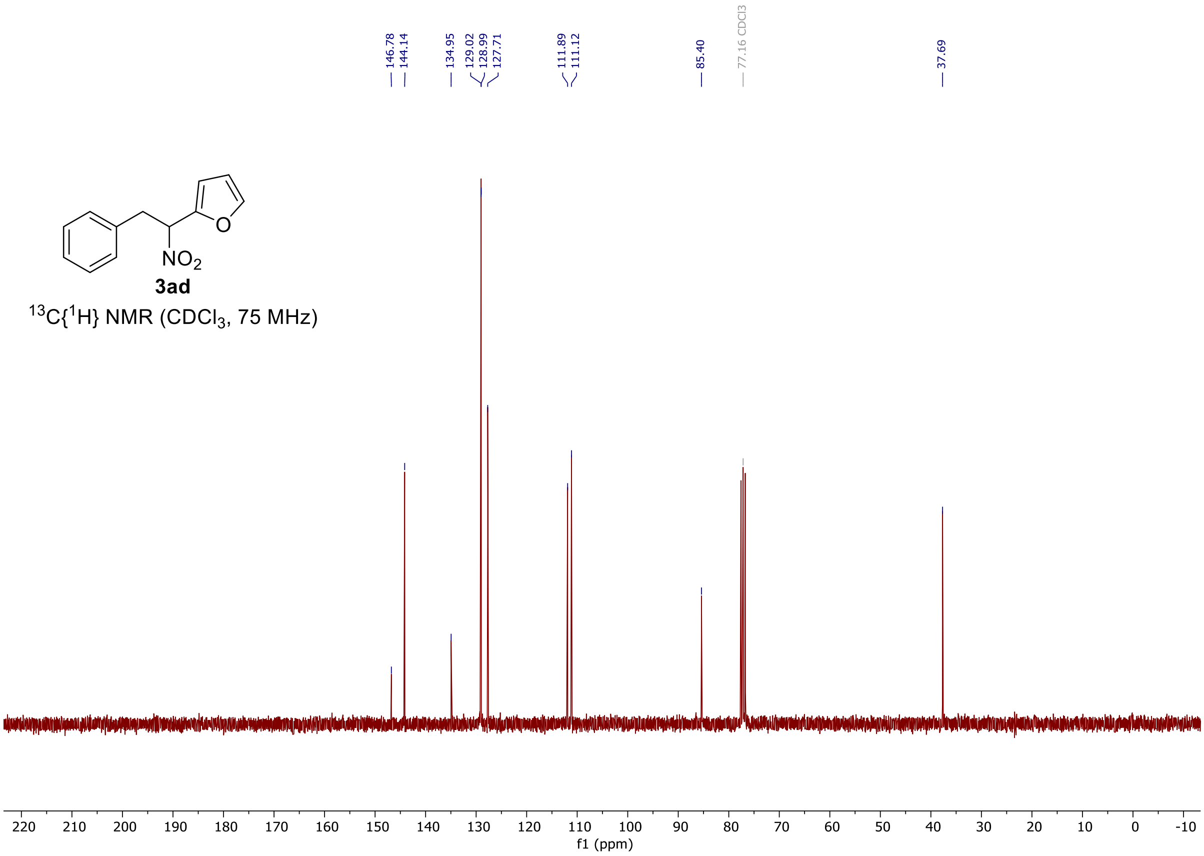

Figure S85

4.42. NMR spectra of 3ae

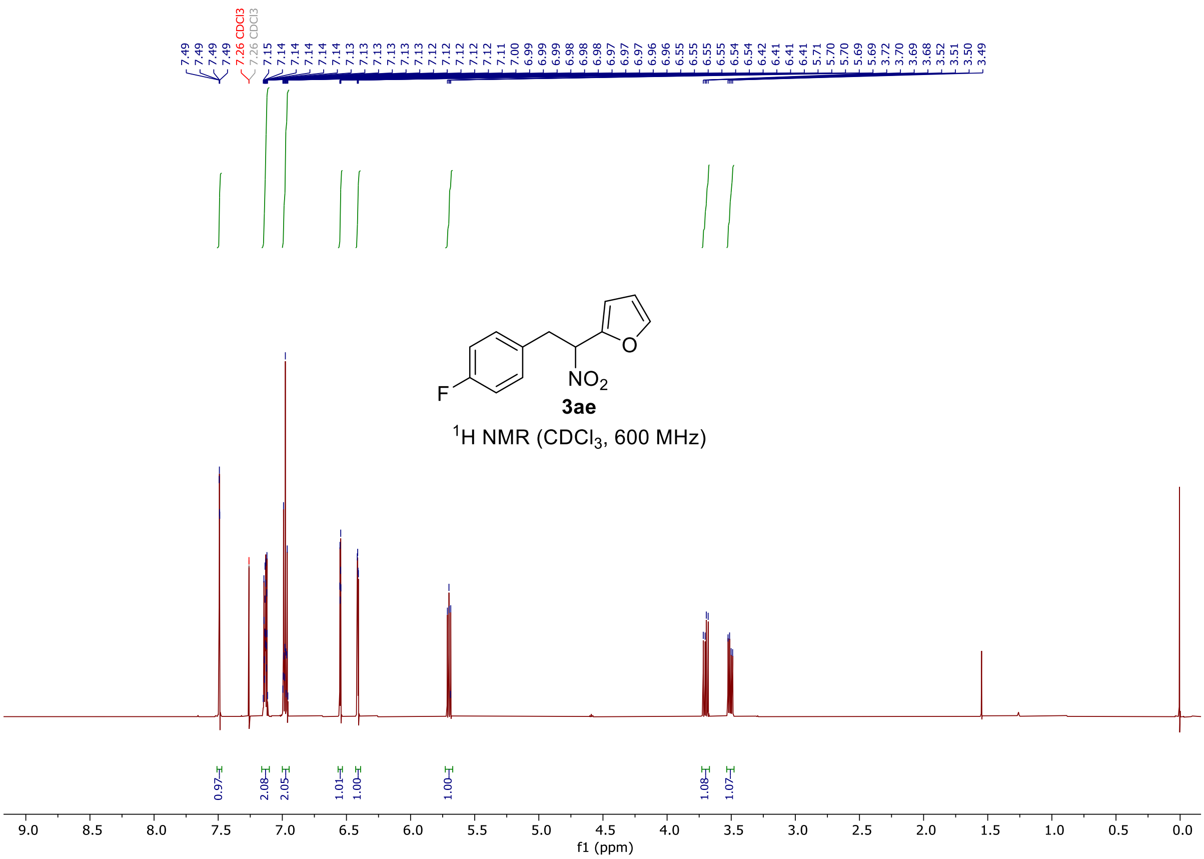

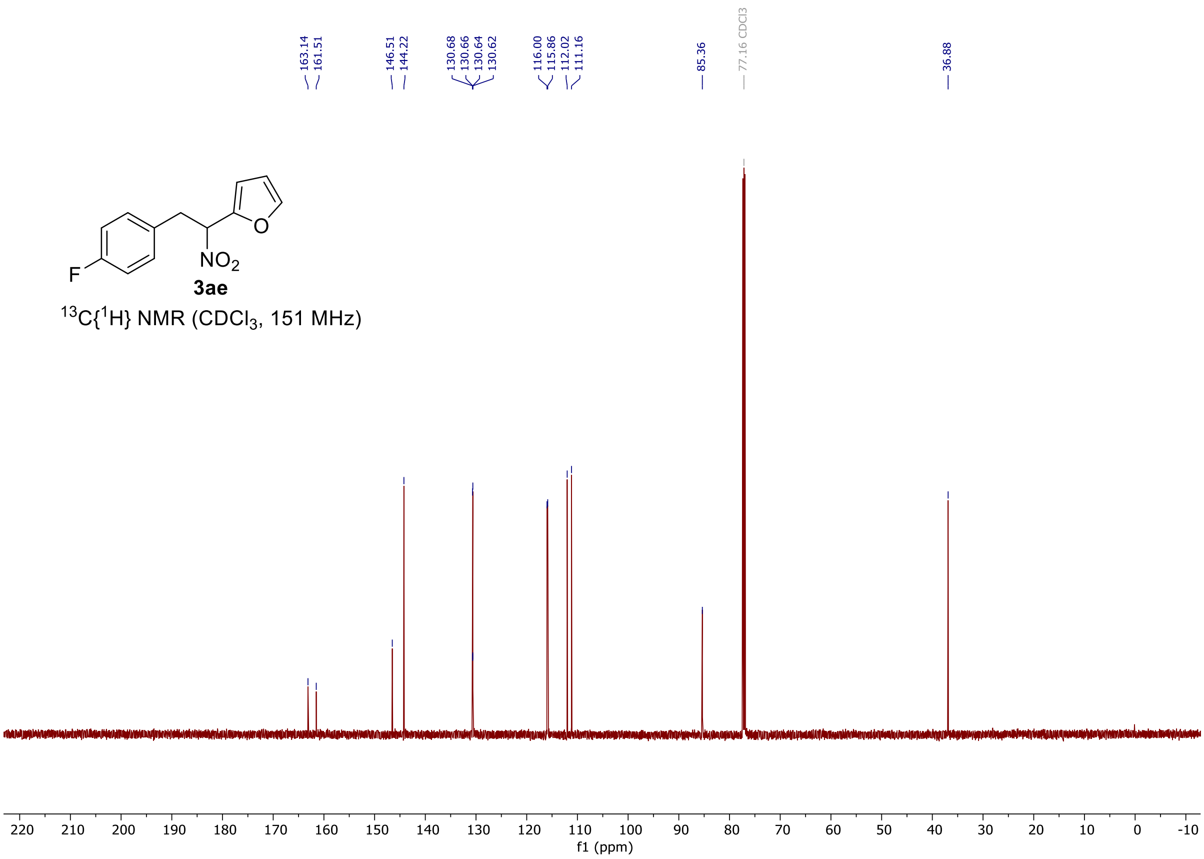

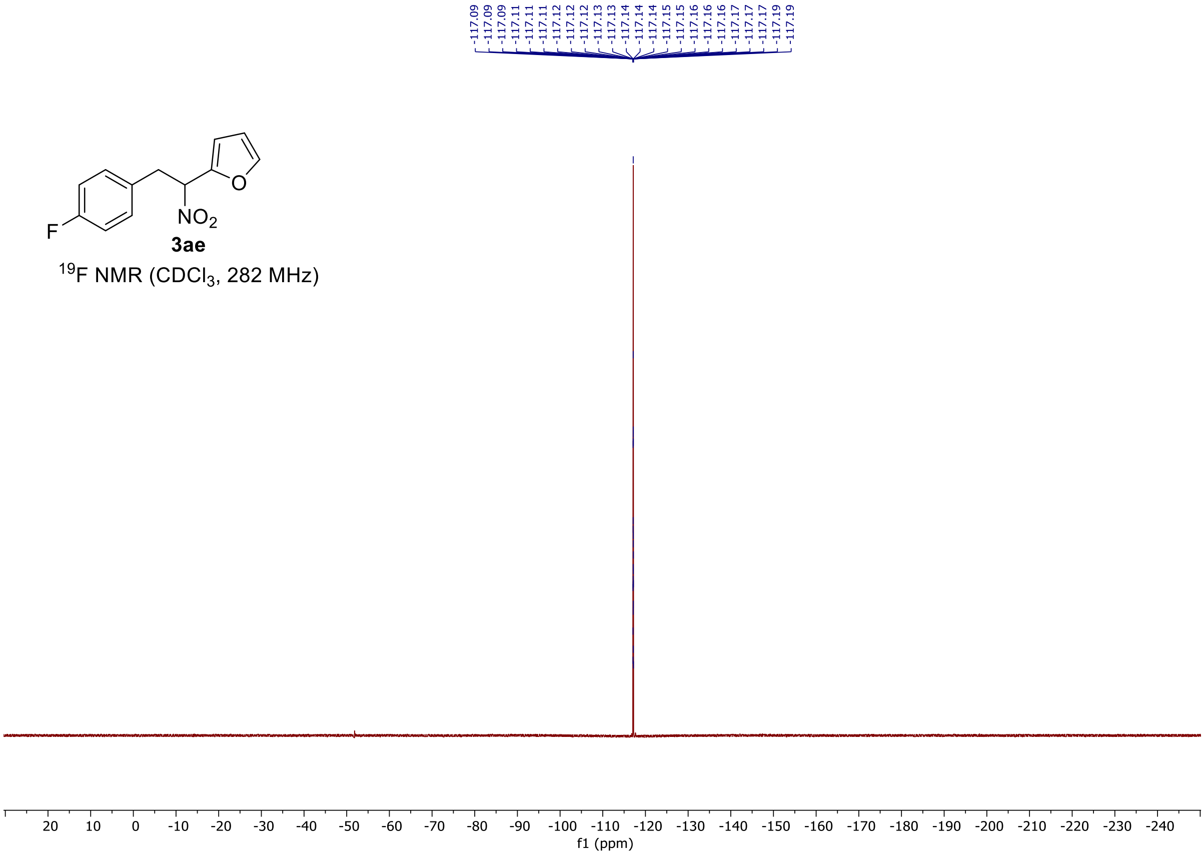
-117.09
-117.09
-117.09
-117.11
-117.11
-117.11
-117.12
-117.12
-117.12
-117.13
-117.13
-117.14
-117.14
-117.14
-117.15
-117.15
-117.16
-117.16
-117.17
-117.17
-117.17
-117.19
-117.19
F
NO2
O
3ae
19F NMR (CDCl3, 282 MHz)
20 10 0 -10 -20 -30 -40 -50 -60 -70 -80 -90 -100 -110 -120 -130 -140 -150 -160 -170 -180 -190 -200 -210 -220 -230 -240
f1 (ppm)

Figure S86

4.43 NMR spectra of 3ef

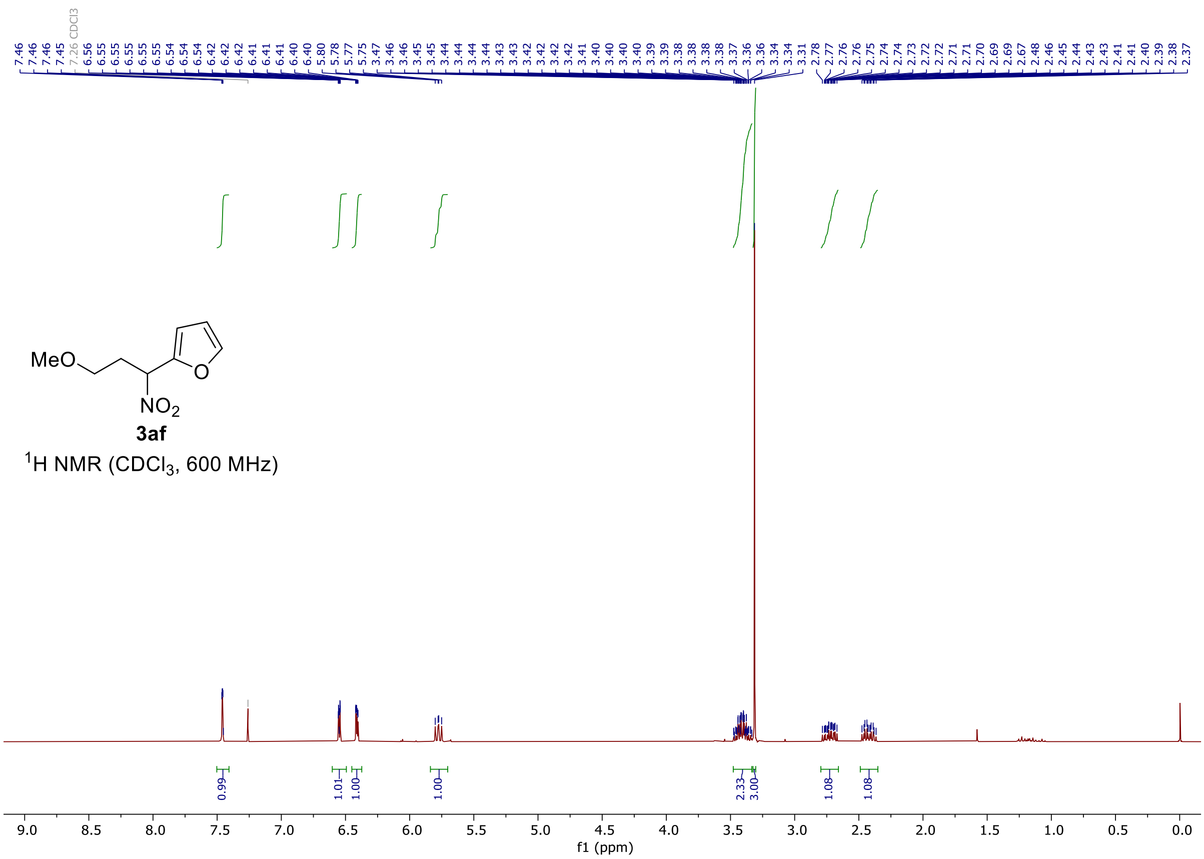

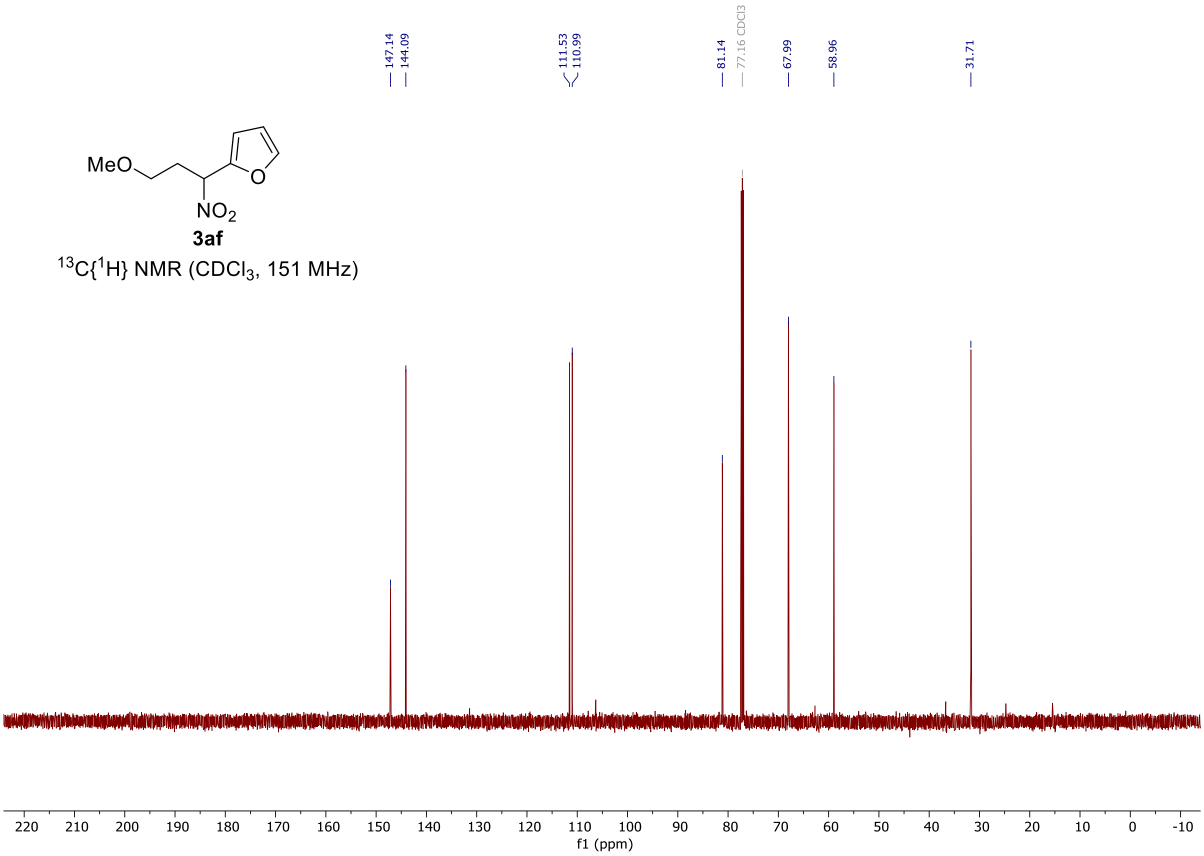

Figure S87

4.44 NMR spectra of 3eg

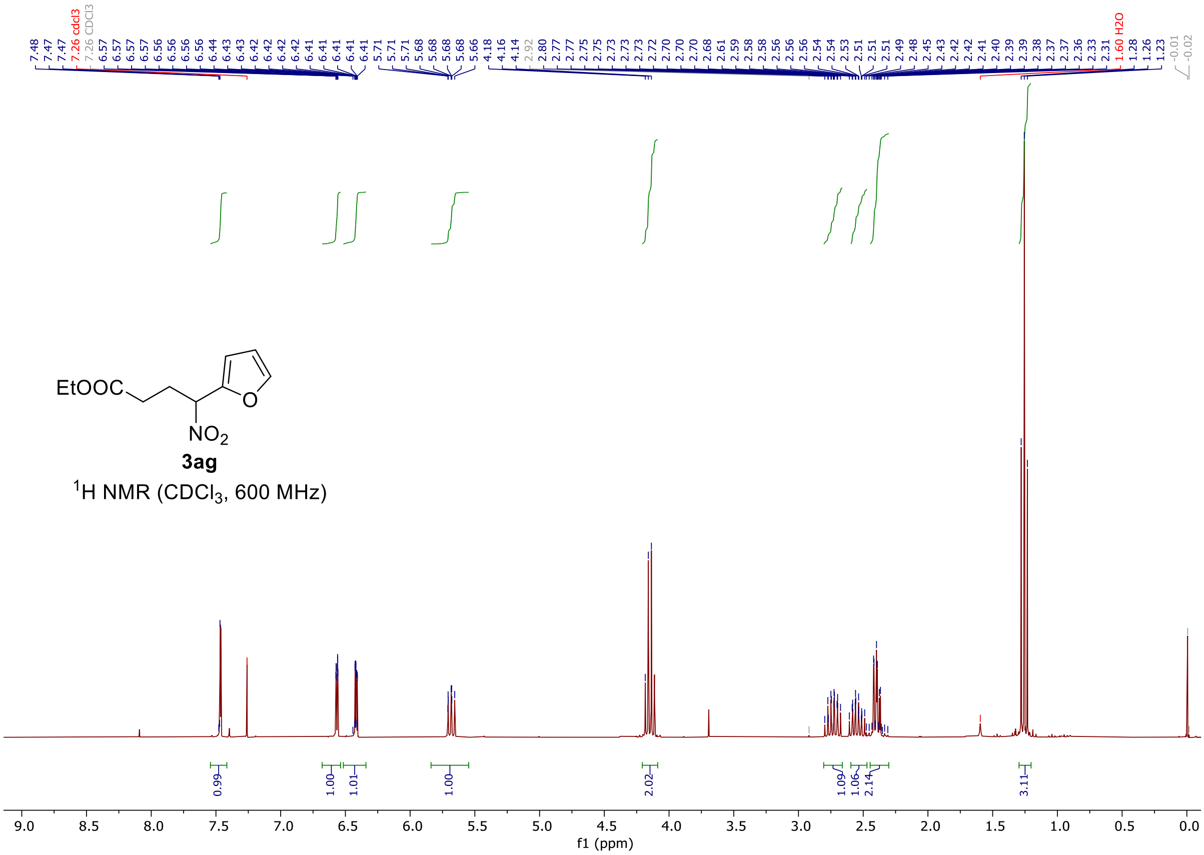

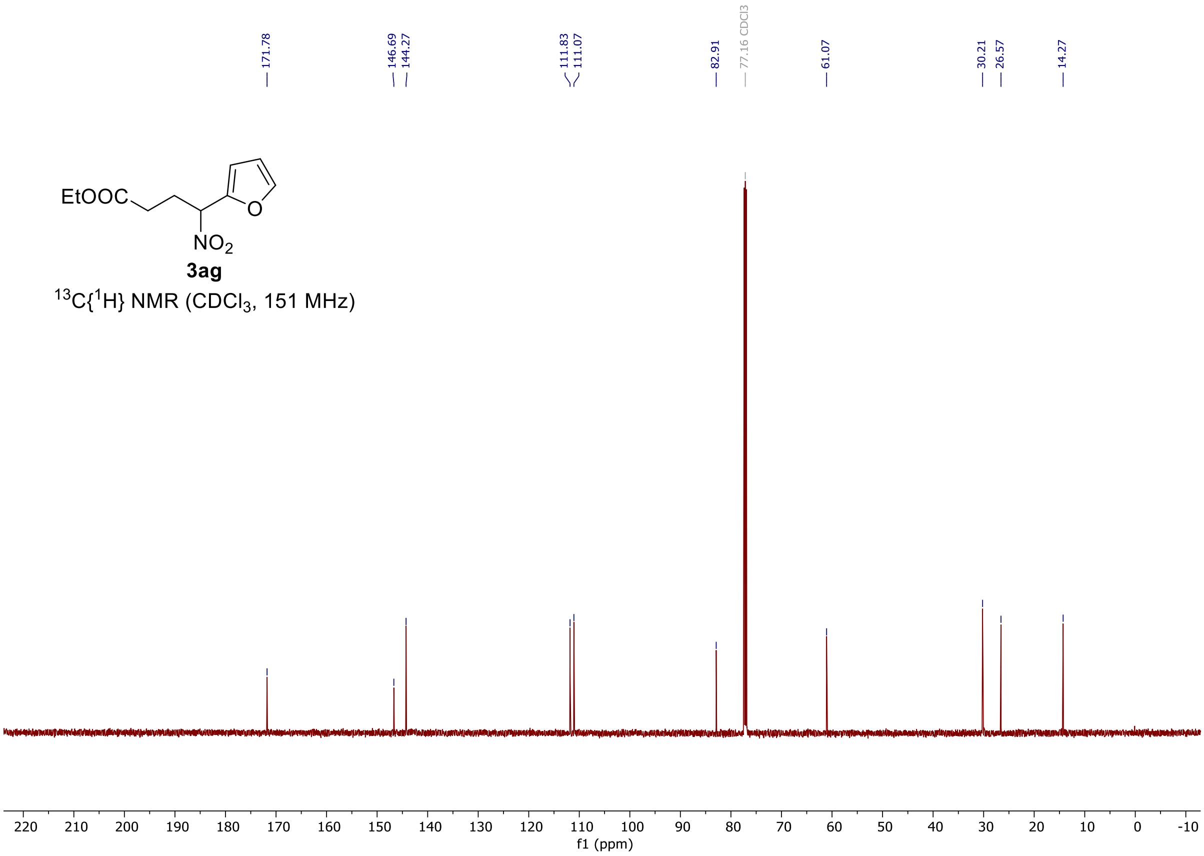

Figure S88

4.45 NMR spectra of 3ah

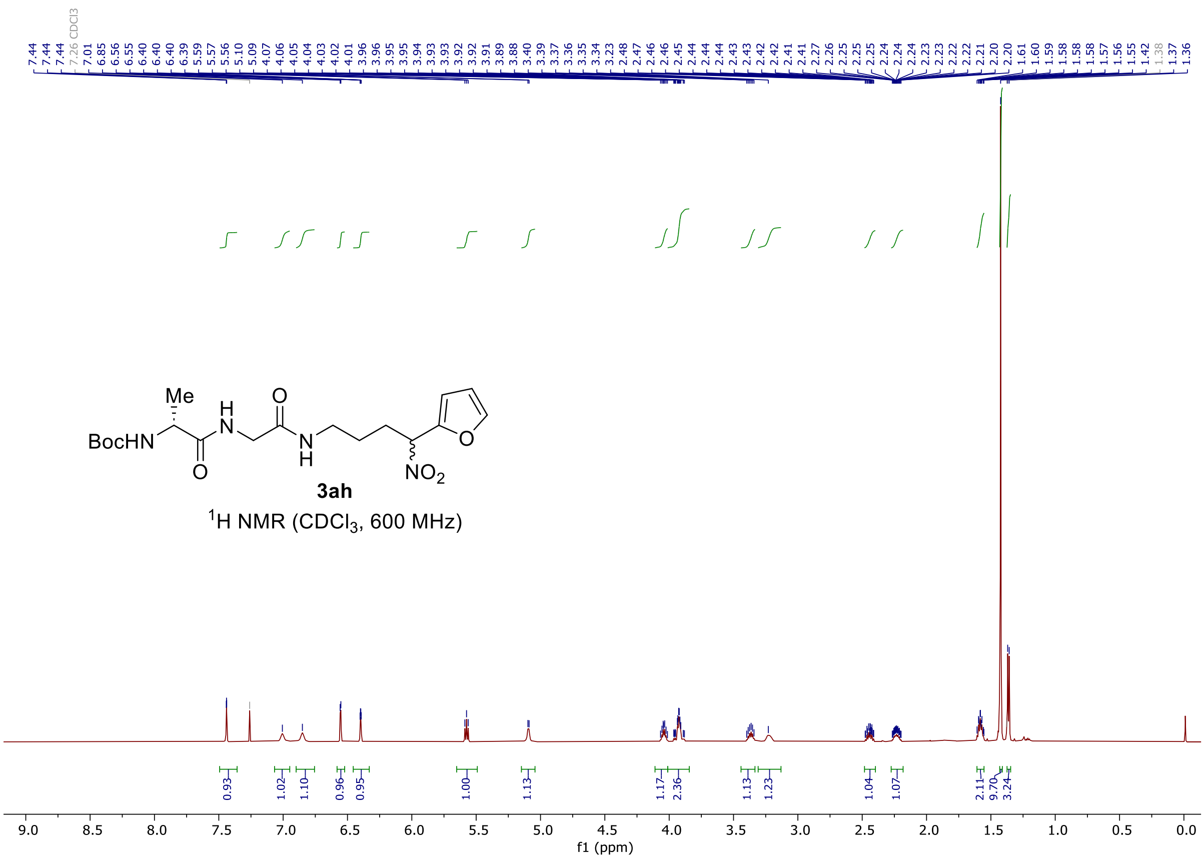

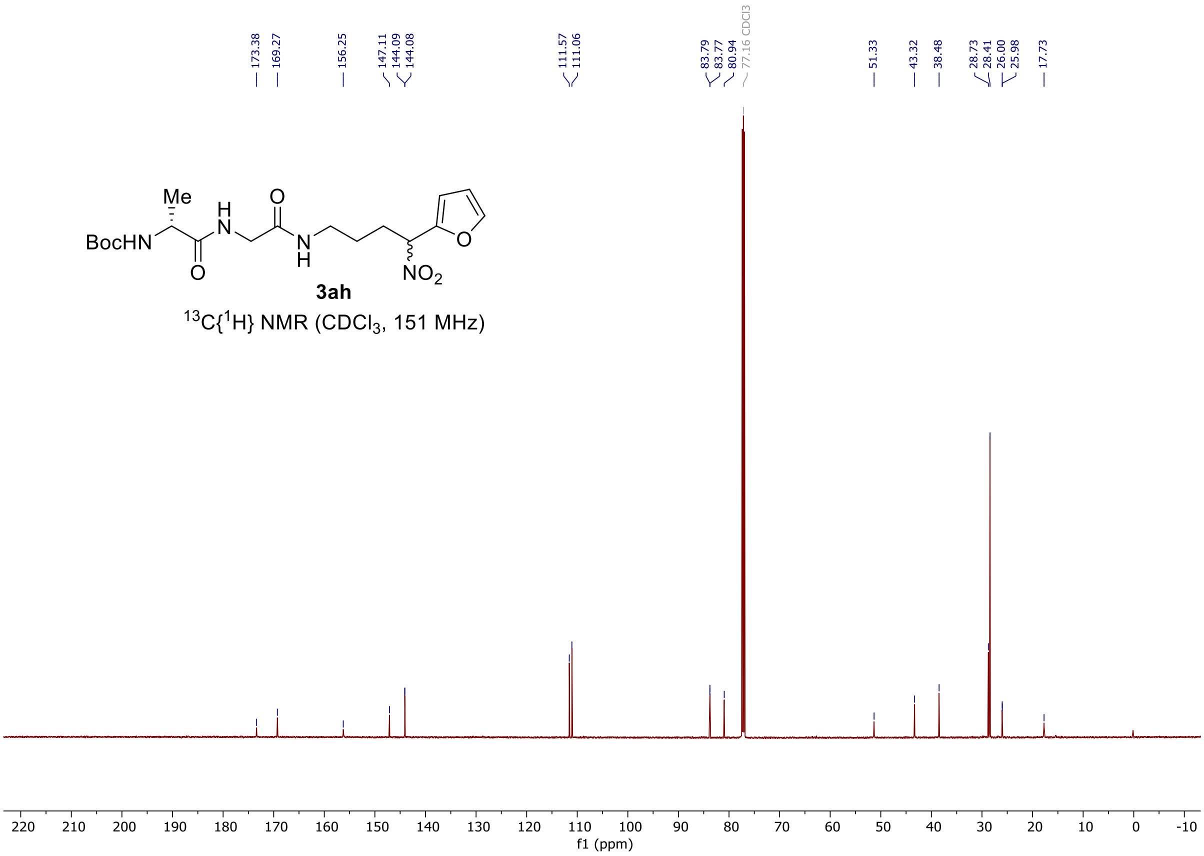

Figure S89

4.46 NMR spectra of 3i

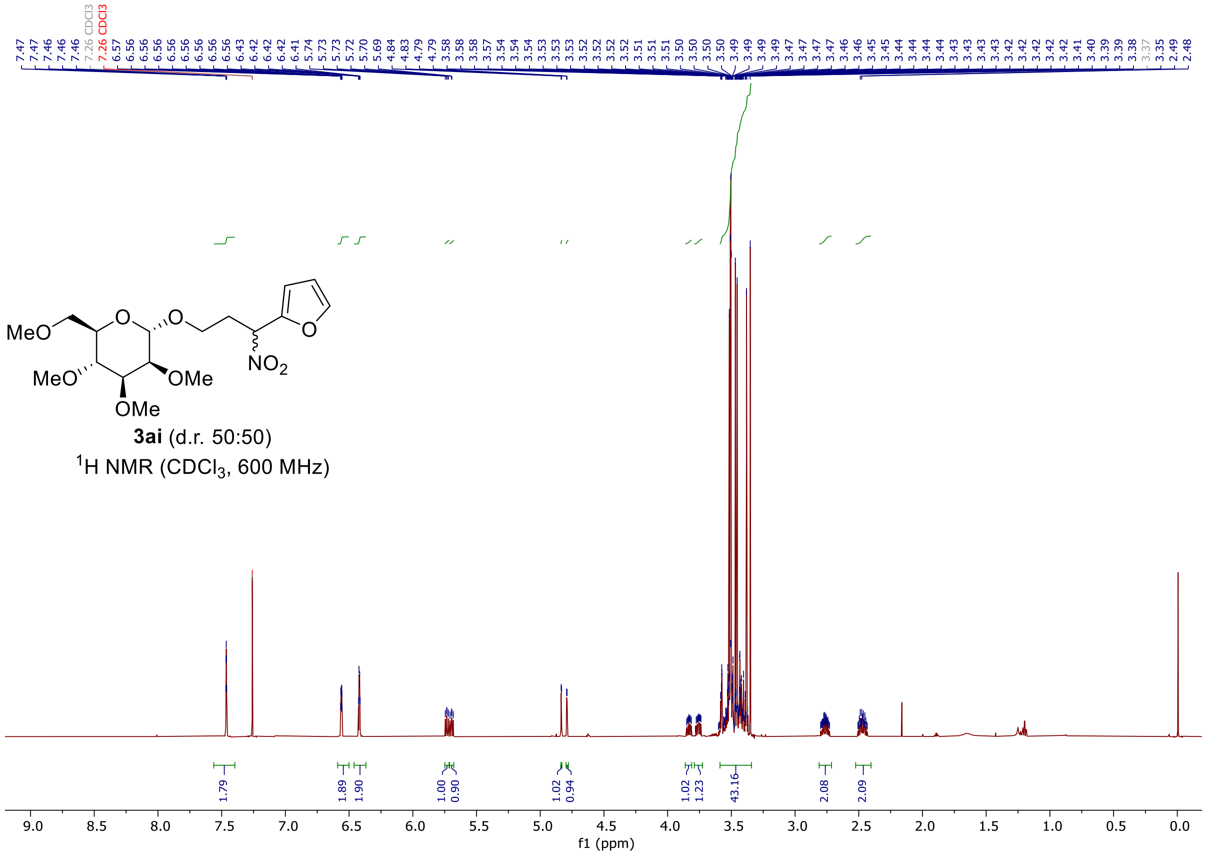

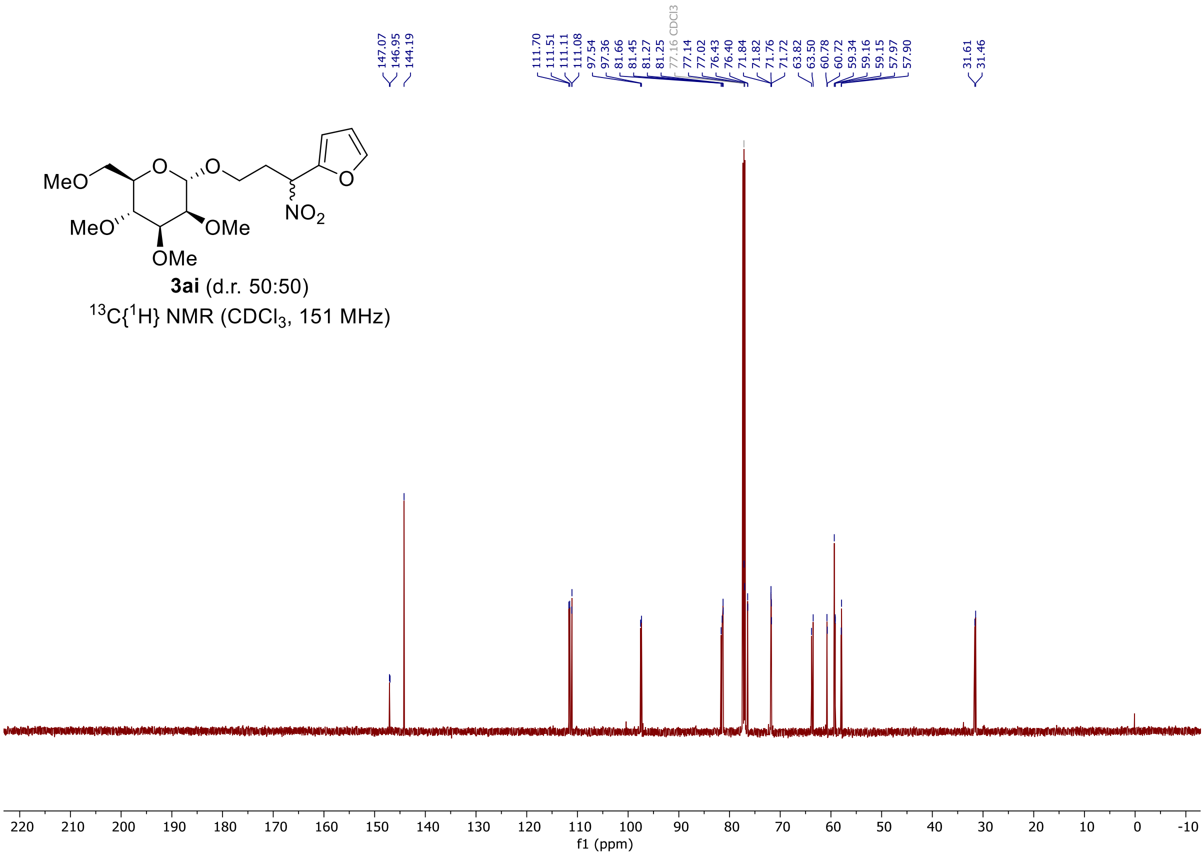

Figure S90

4.47 NMR spectra of 9a

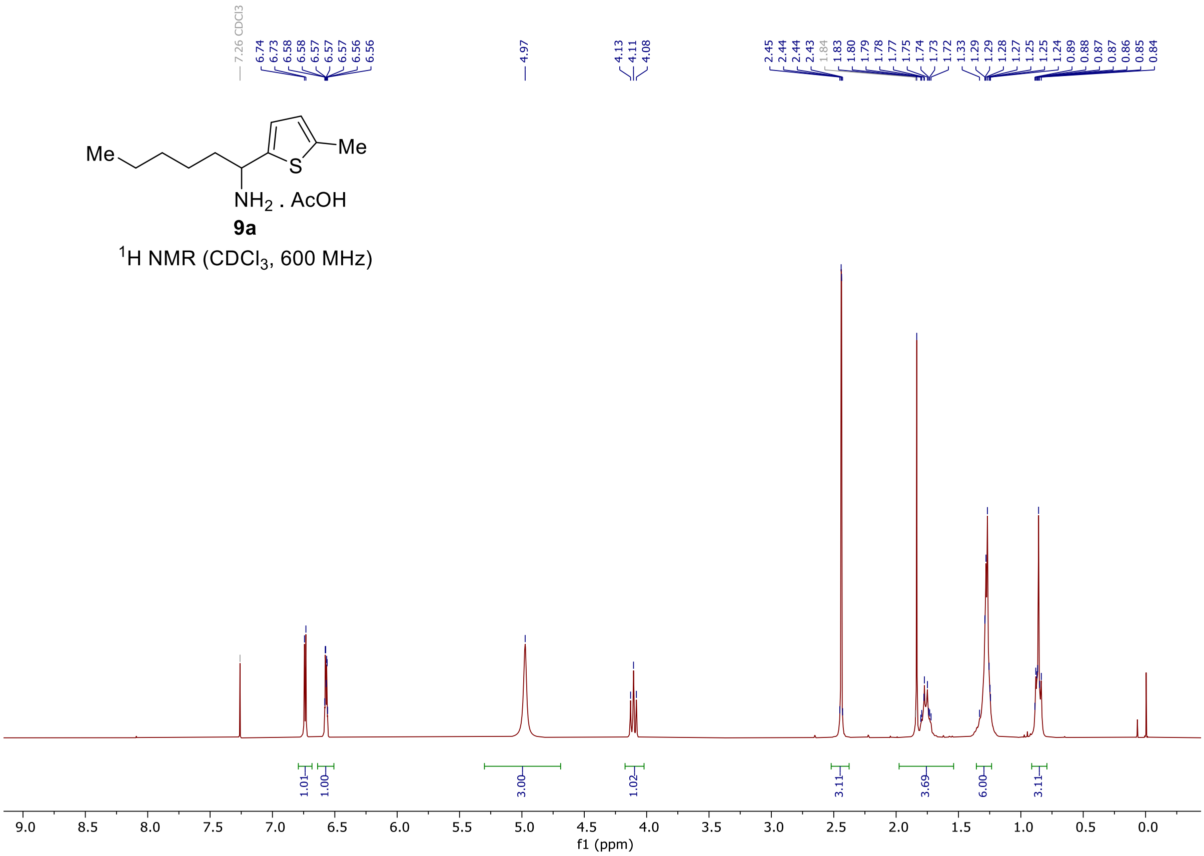

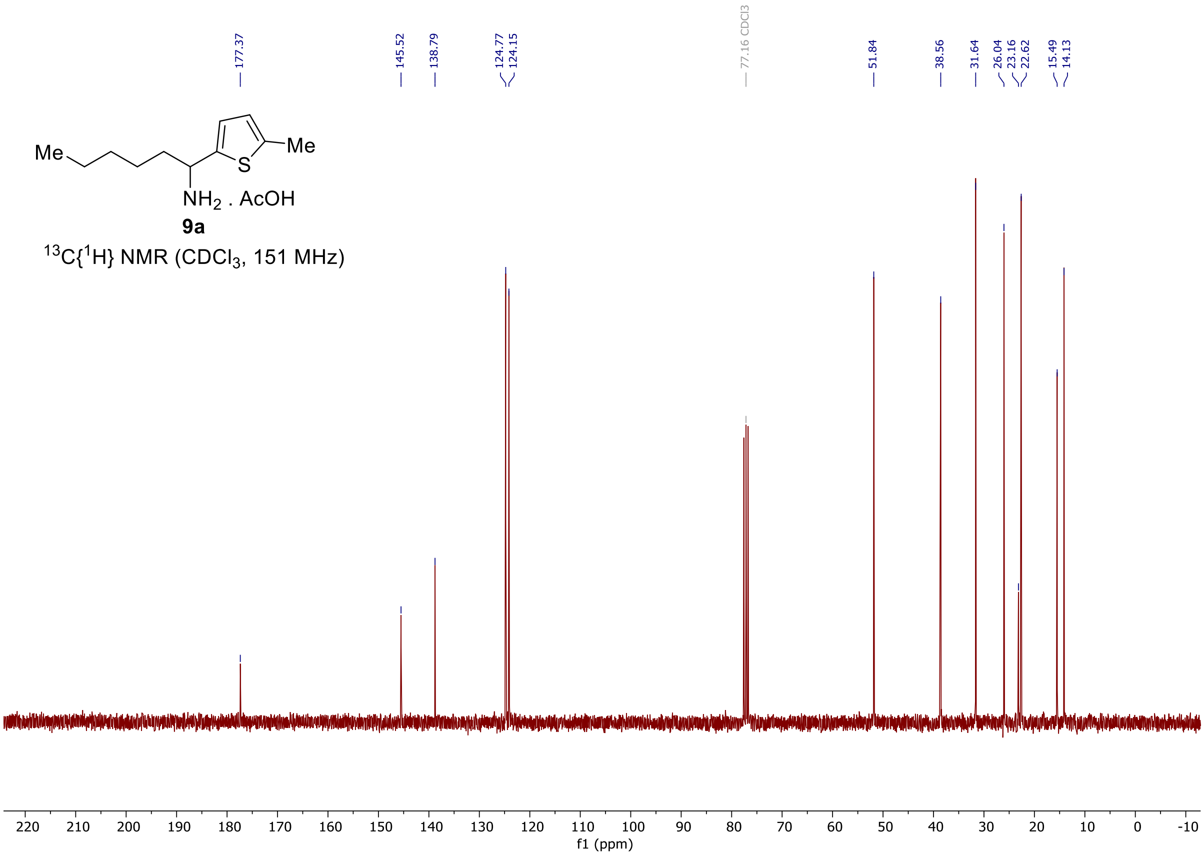

Figure S91

4.48 NMR spectra of 9b

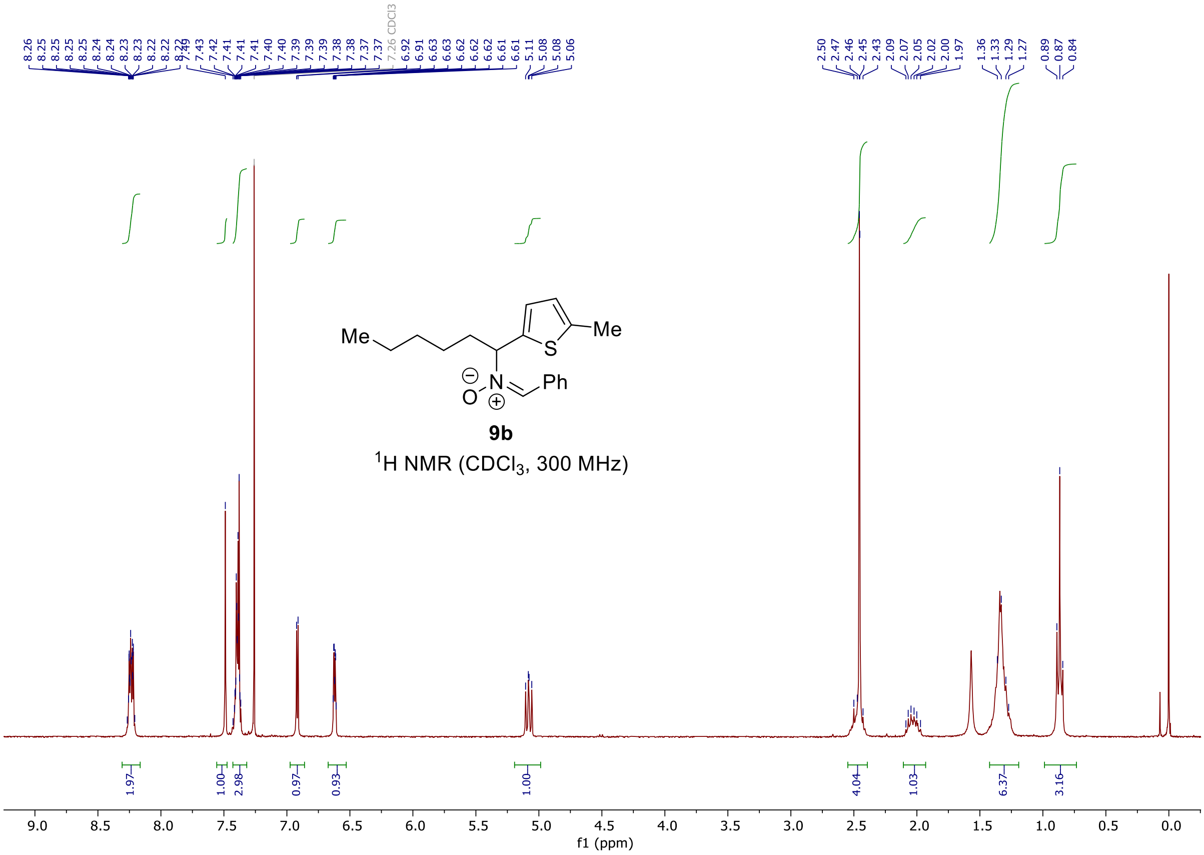

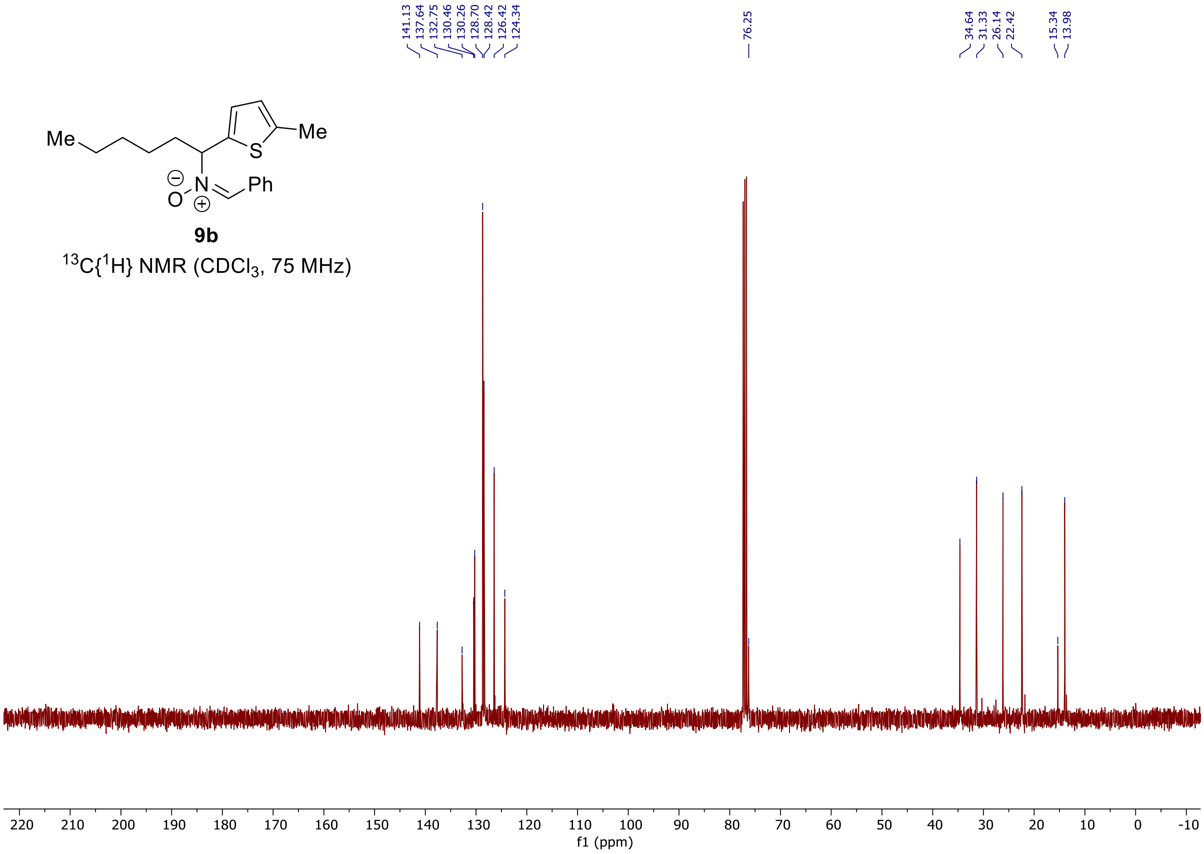

Figure S92

4.49 NMR spectra of 9c

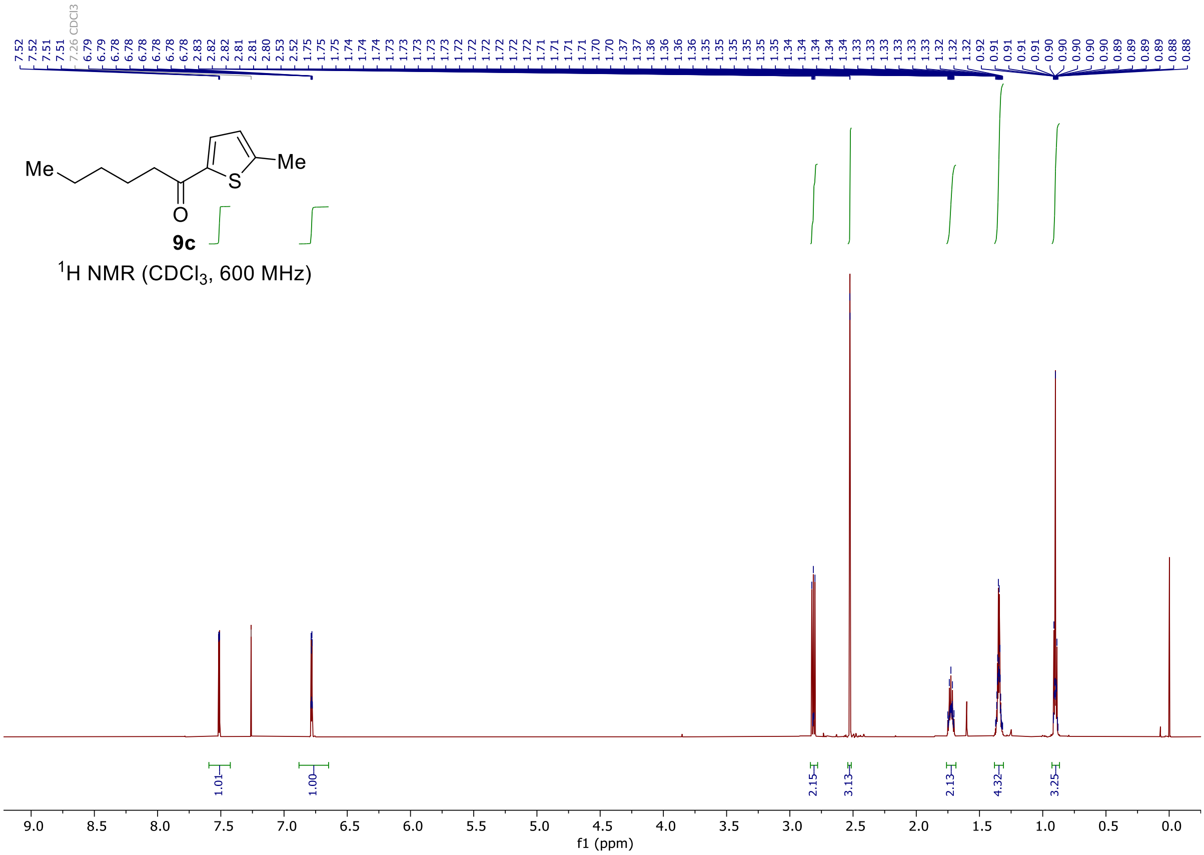

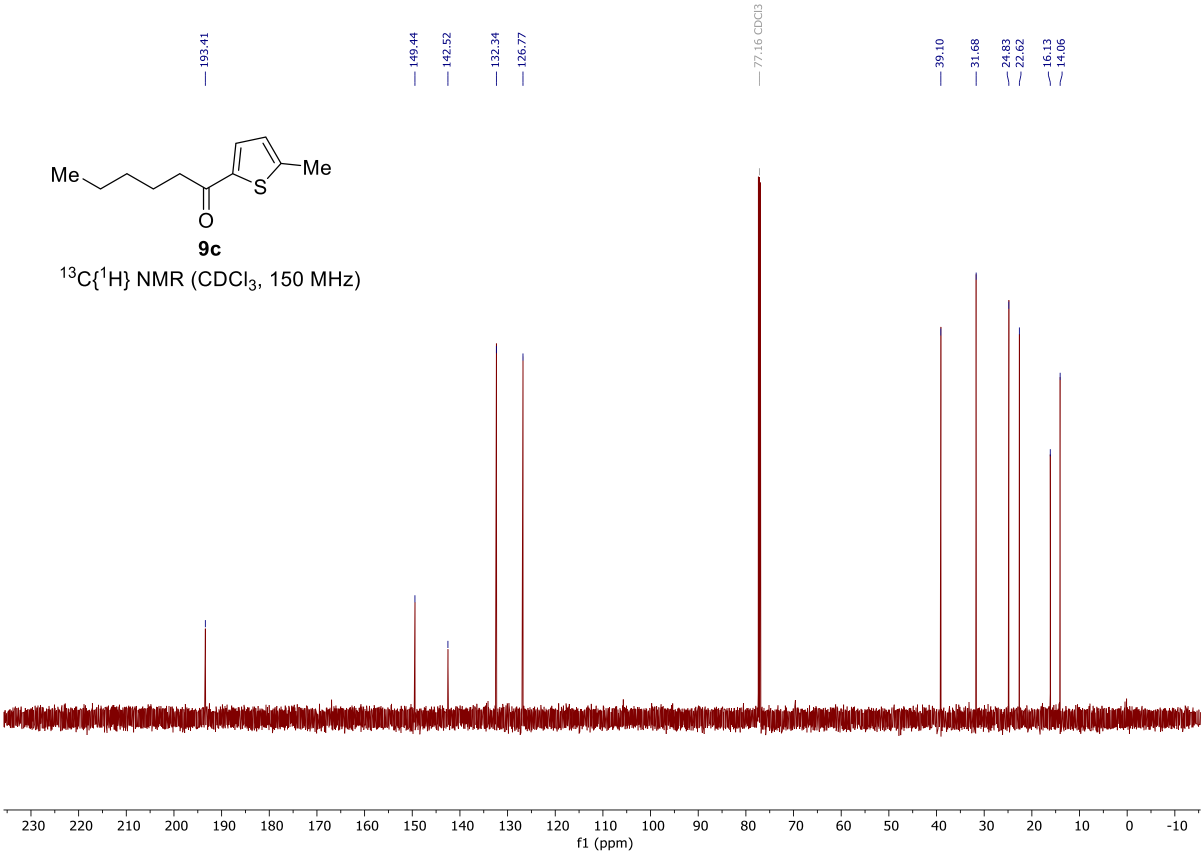

Figure S93

4.50 NMR spectra of 9d

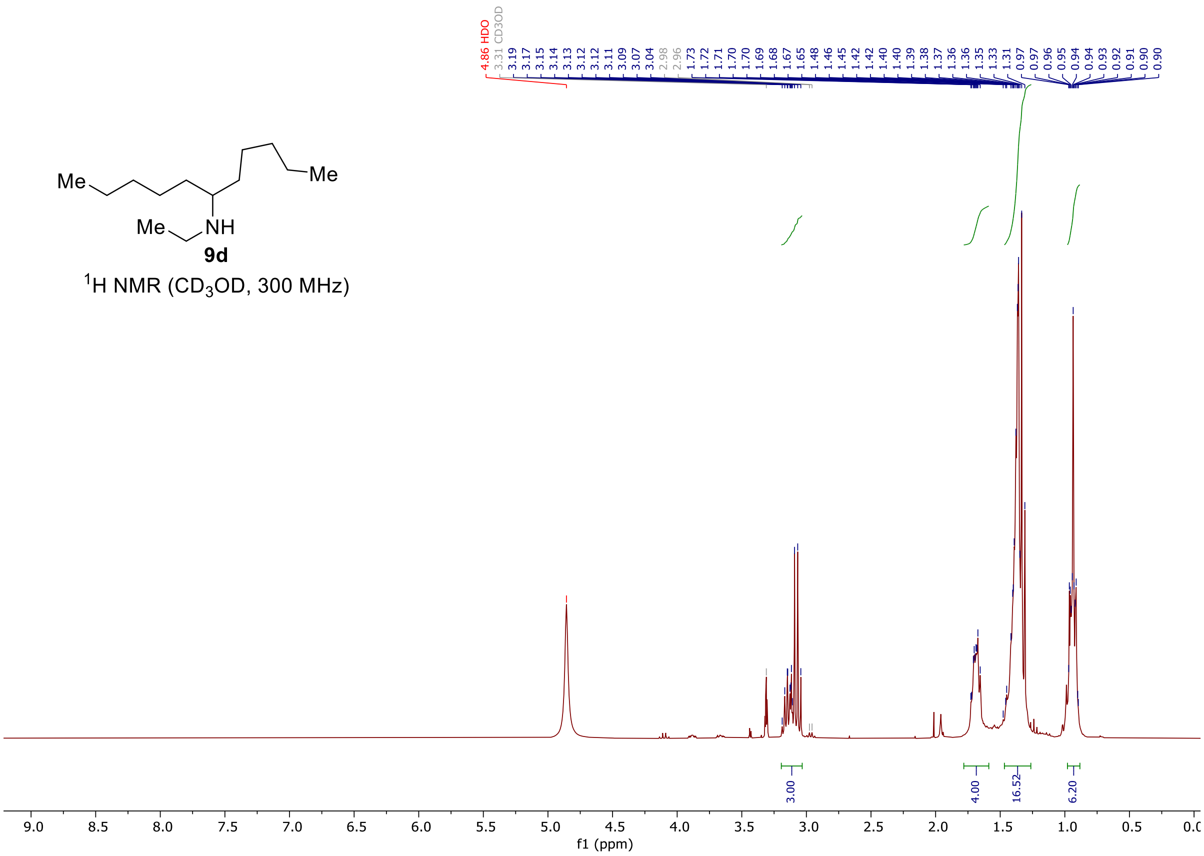

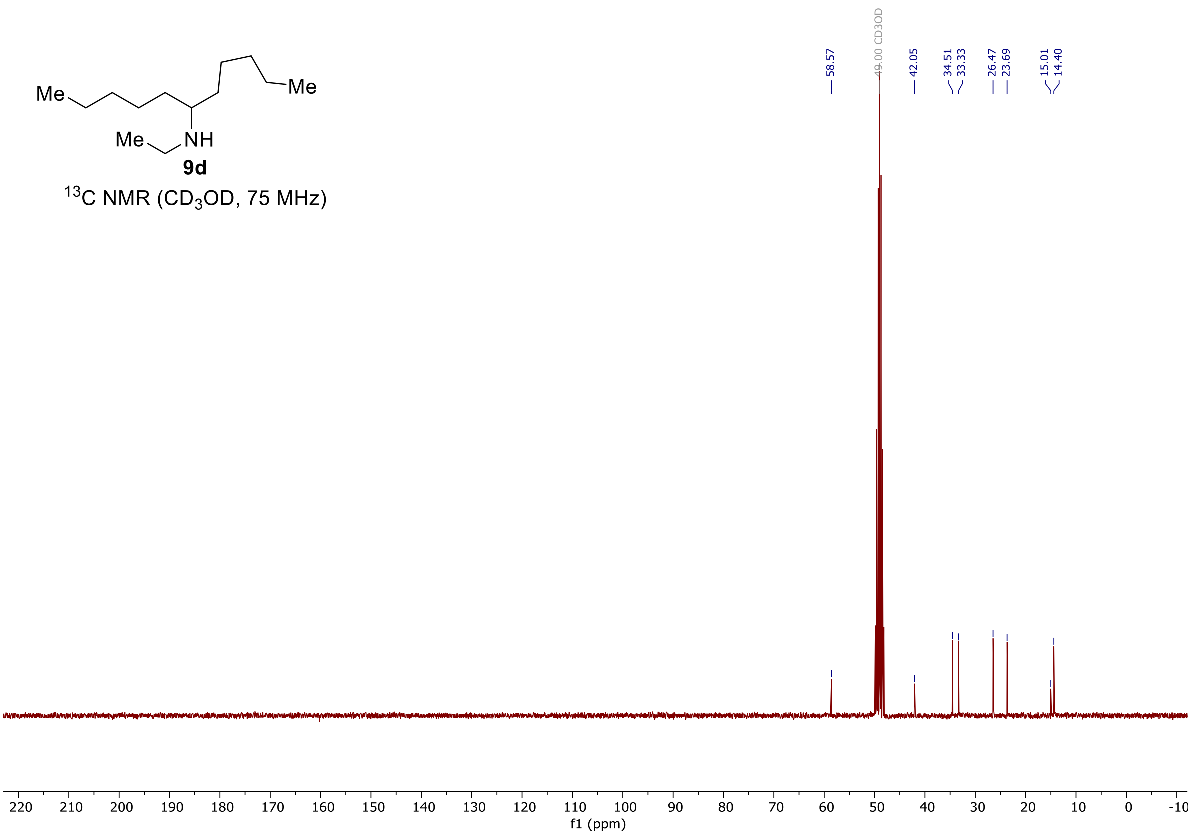

Figure S94

4.51 NMR spectra of 3a´

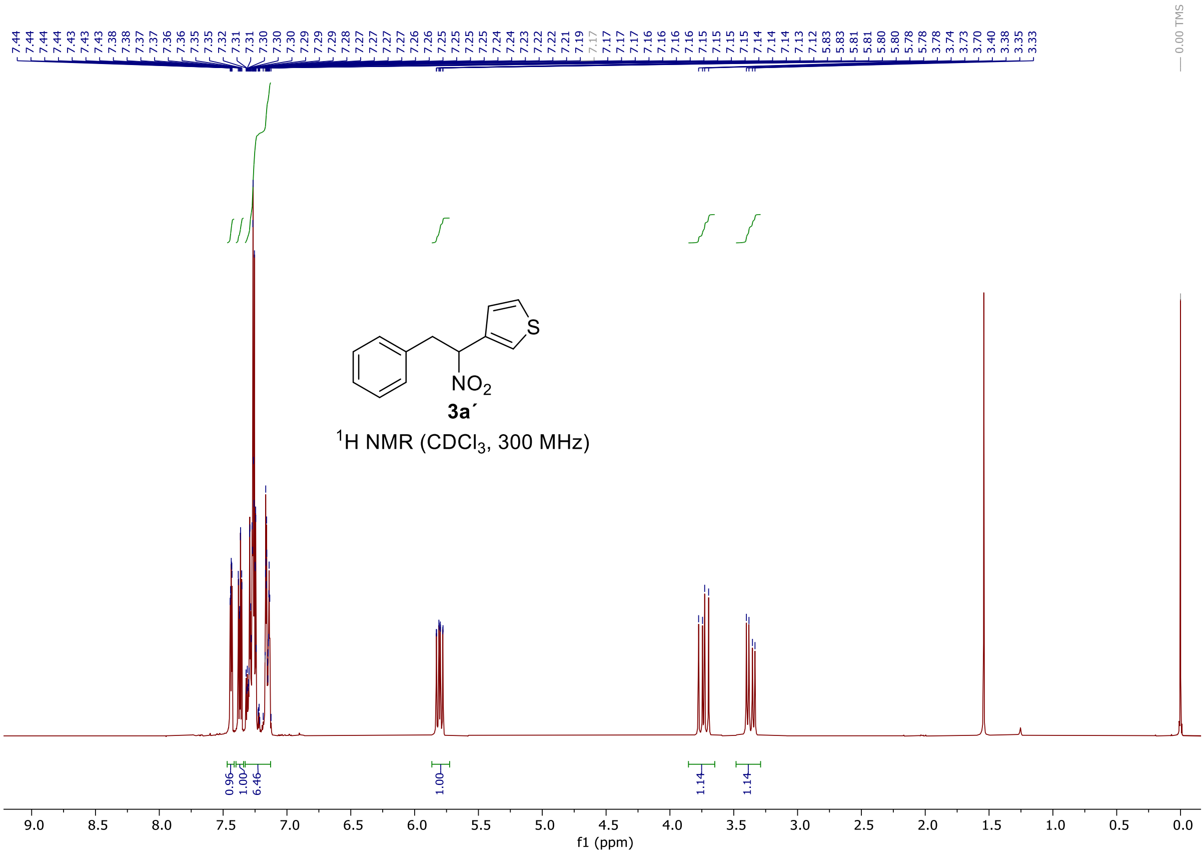

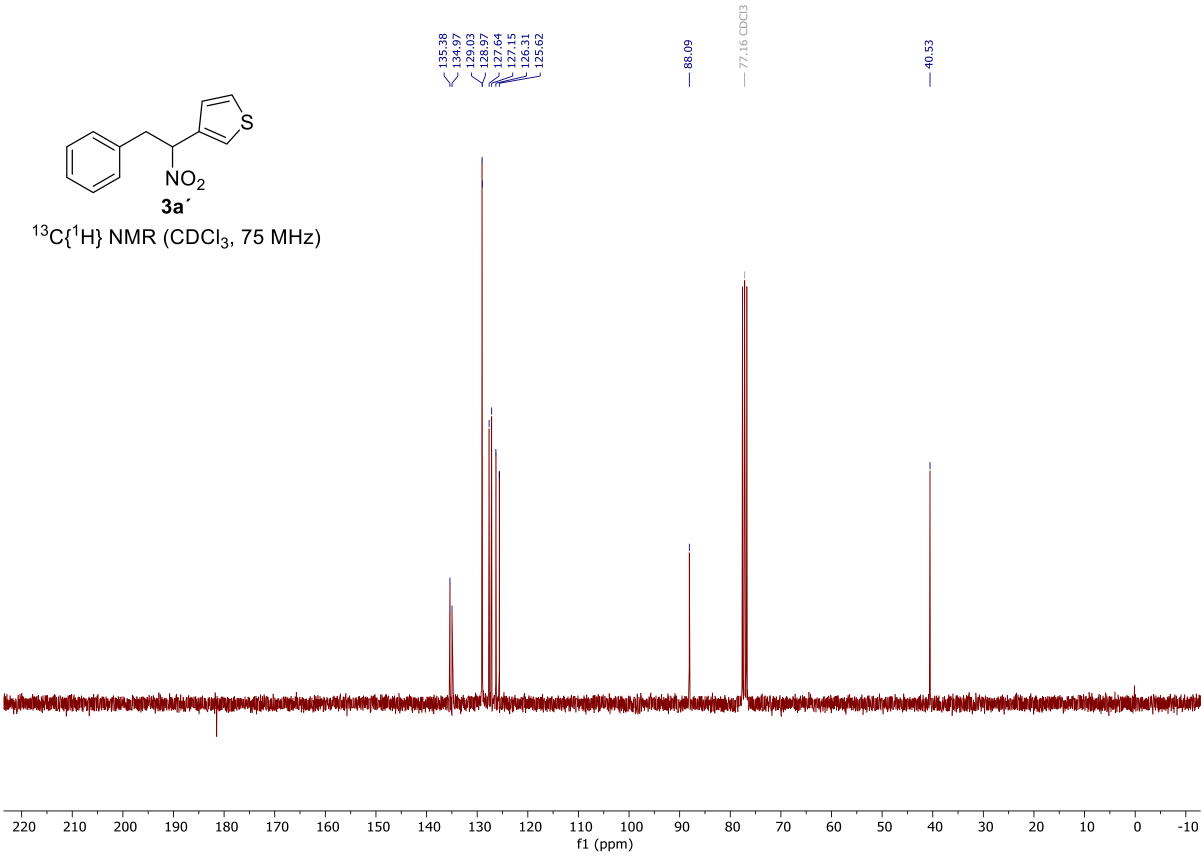

Figure S95

4.52 NMR spectra of 21e

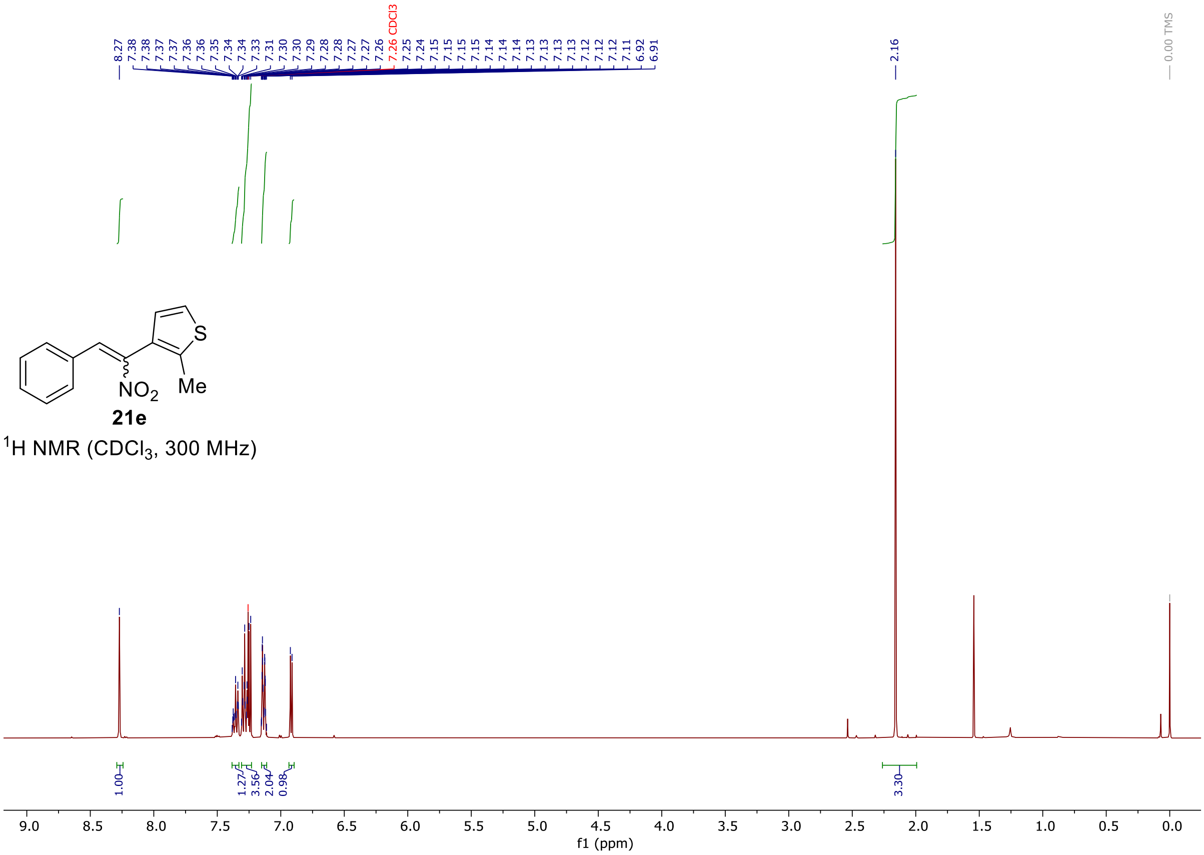

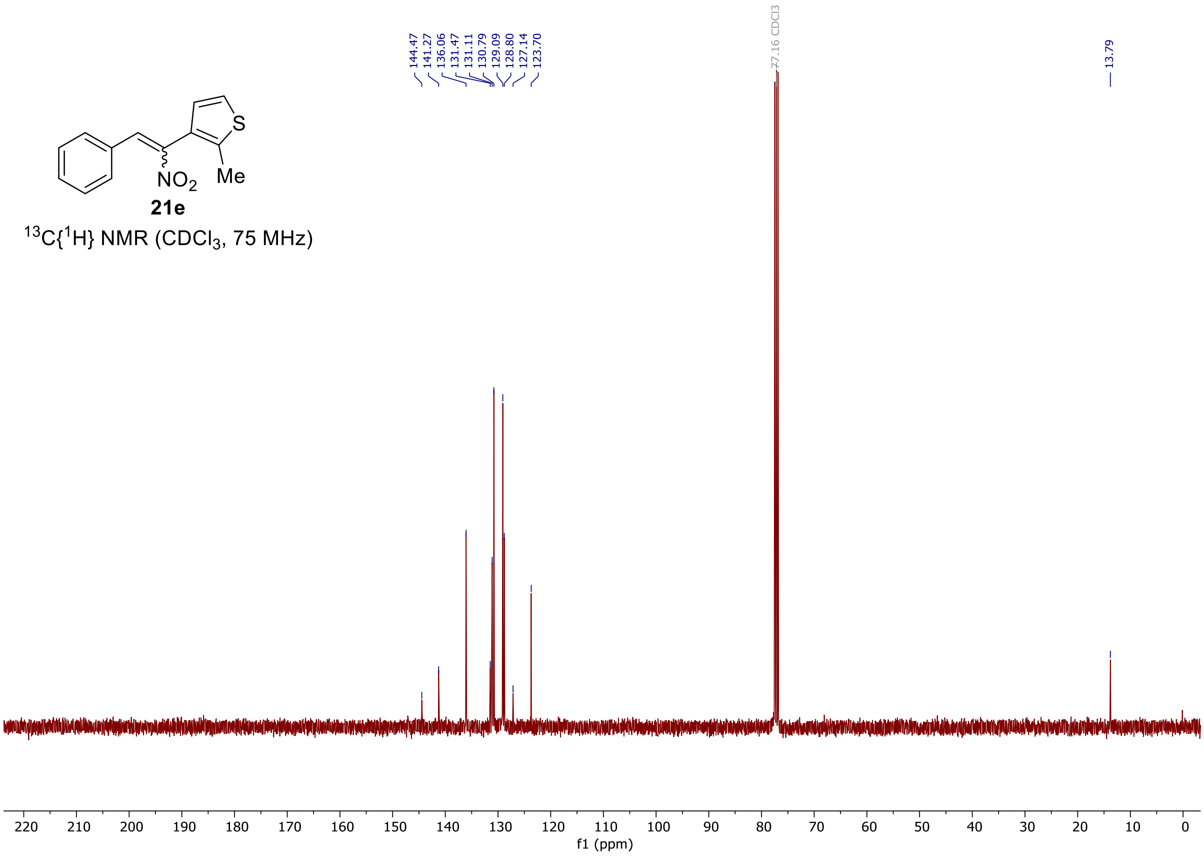

Figure S96

4.53 NMR spectra of 3b´

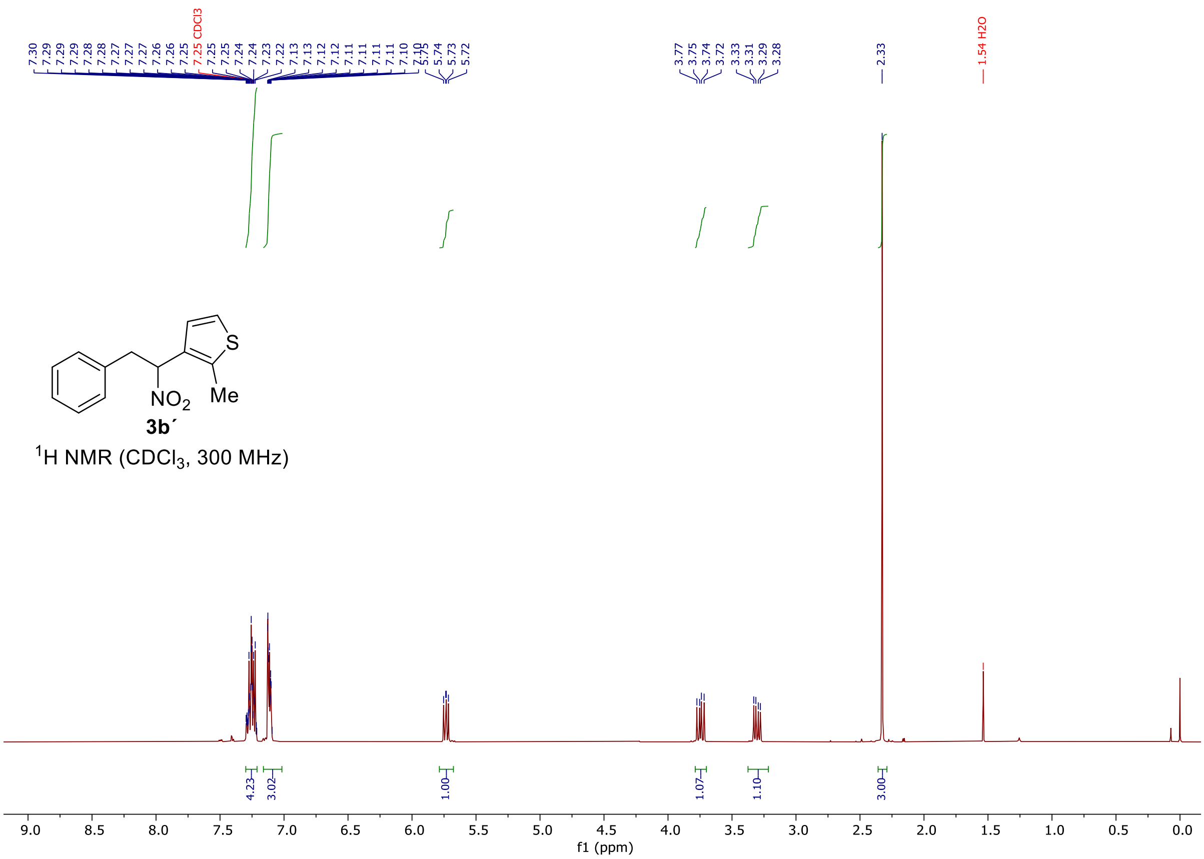

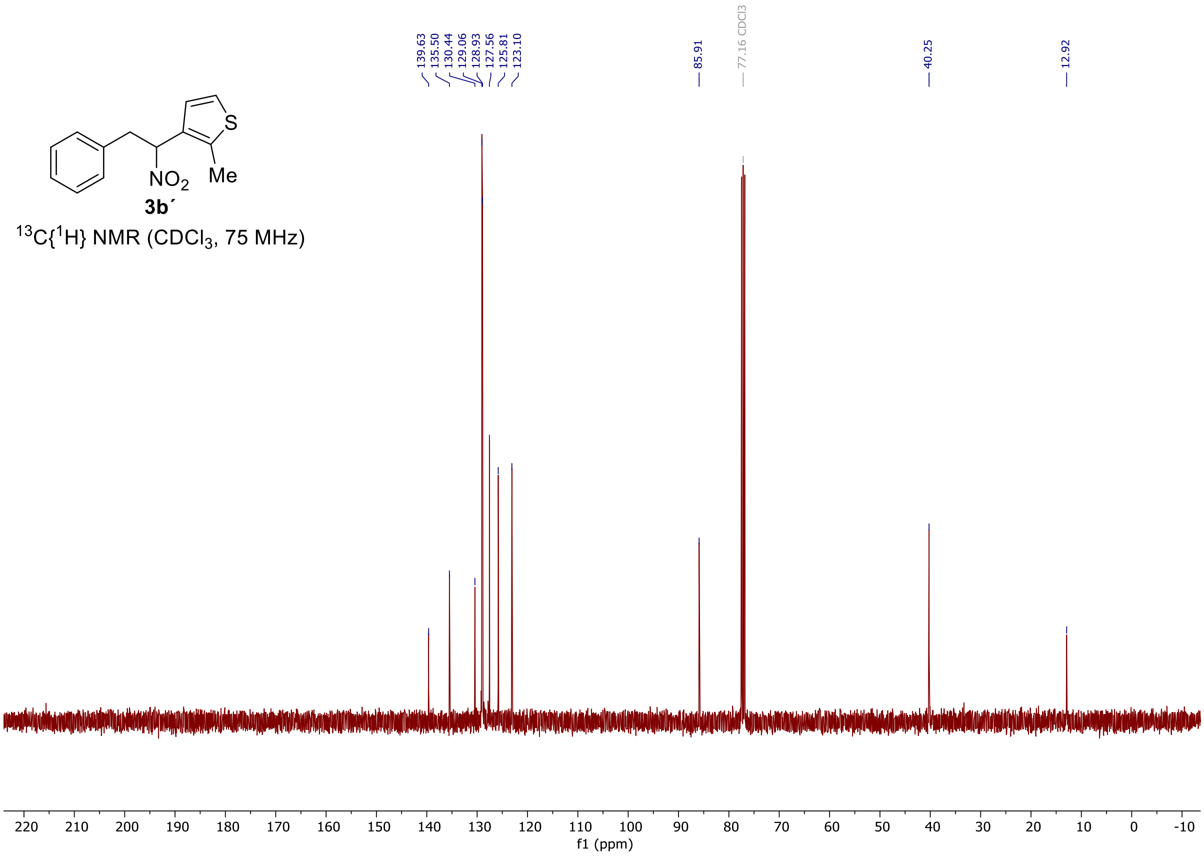

Figure S97

4.54 NMR spectra of 21f

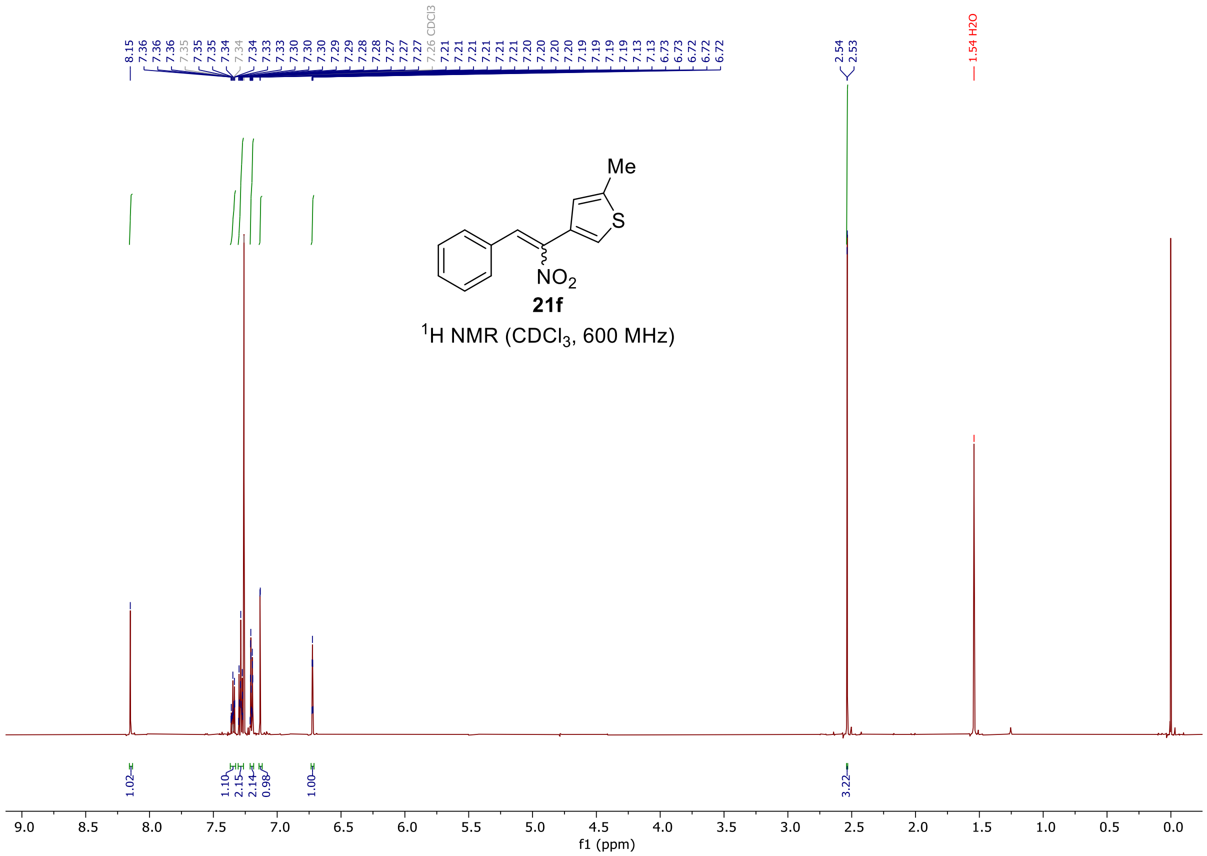

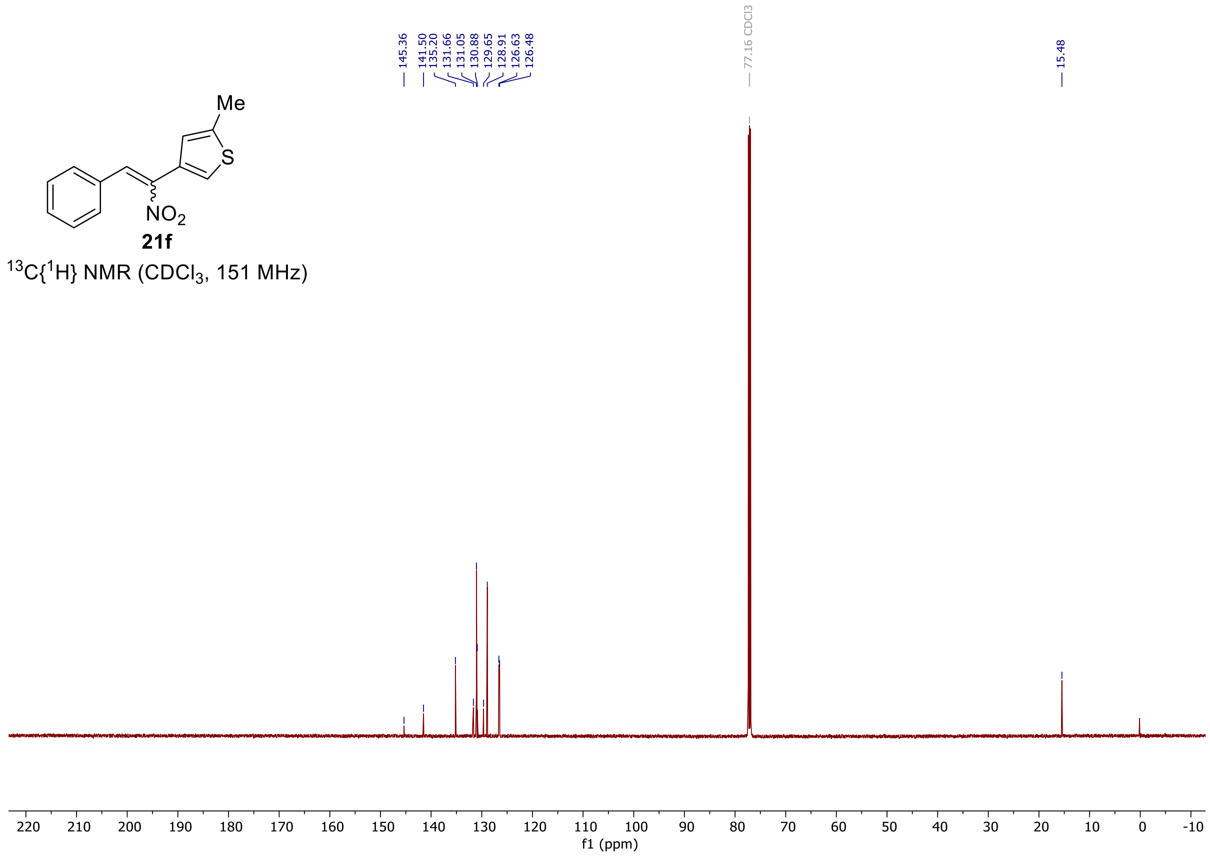

Figure S98

4.55 NMR spectra of 3b´´

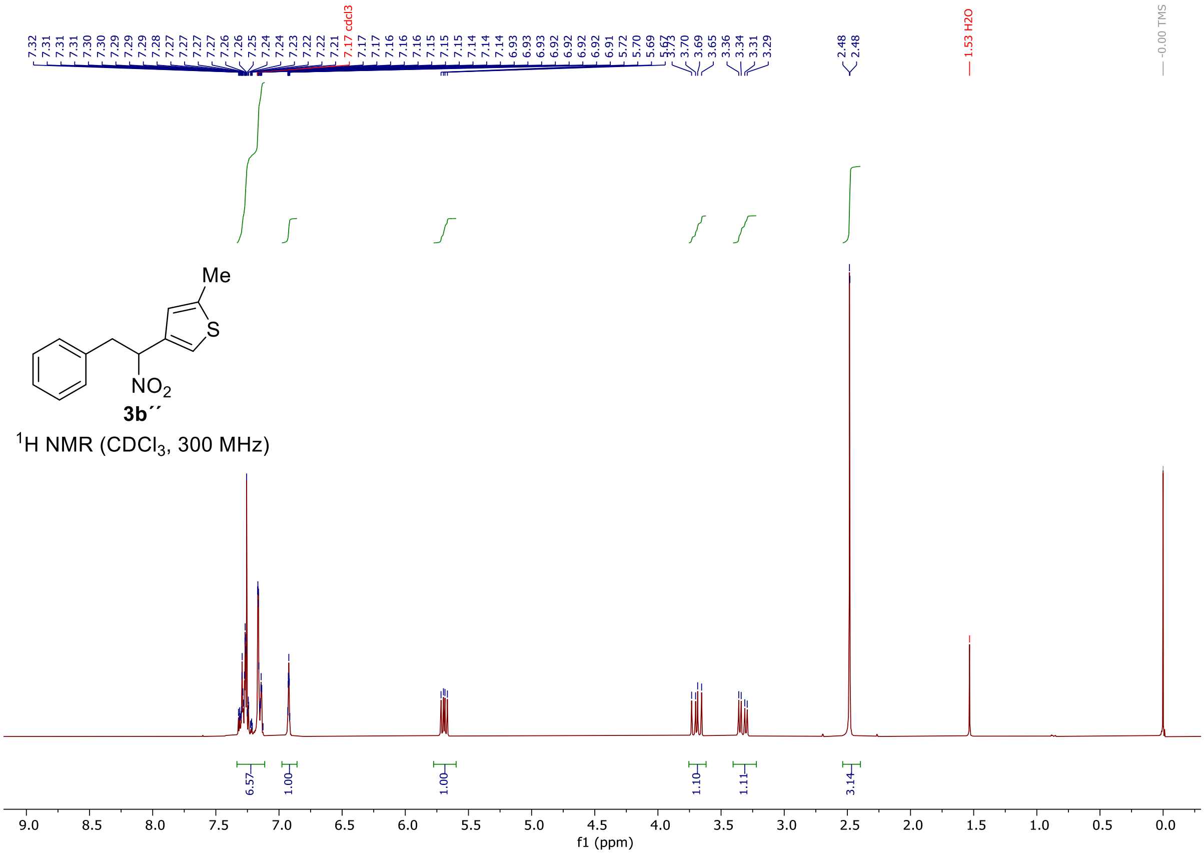

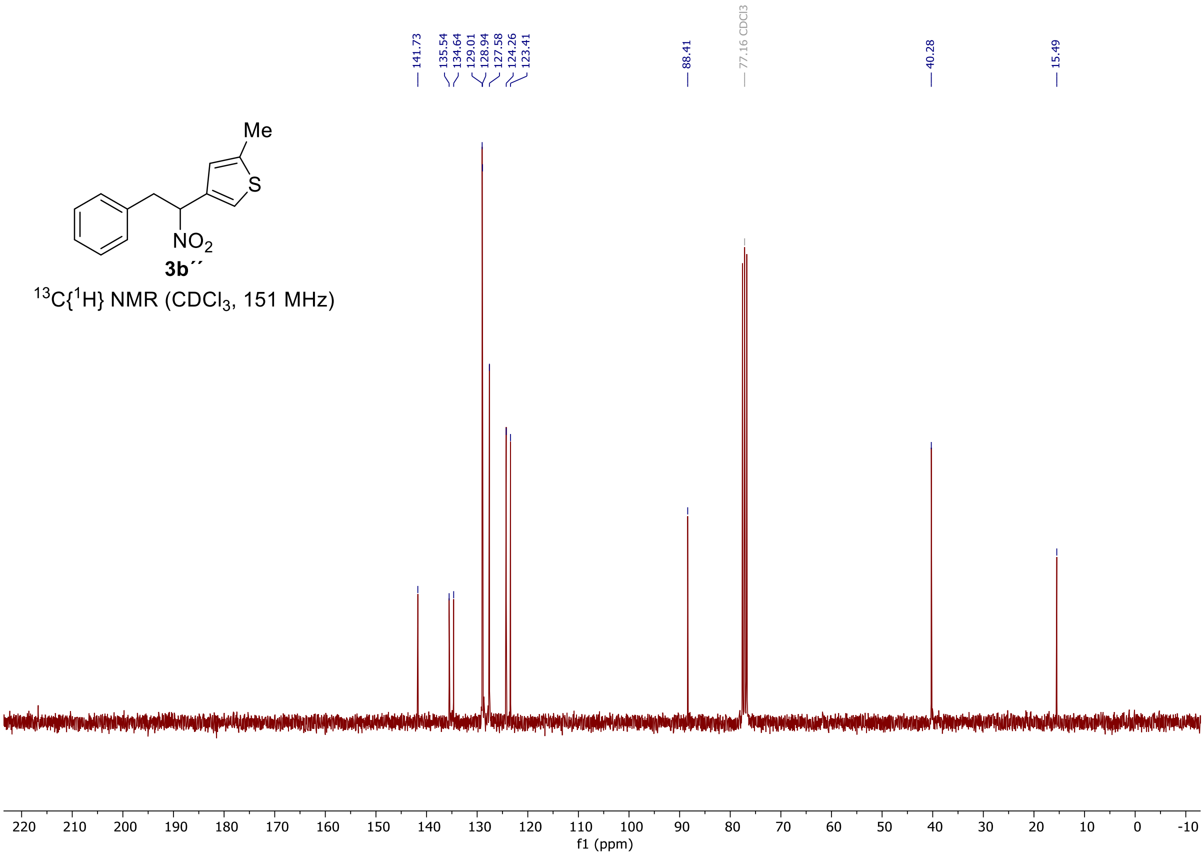

Figure S99

4.56 NMR spectra of 7

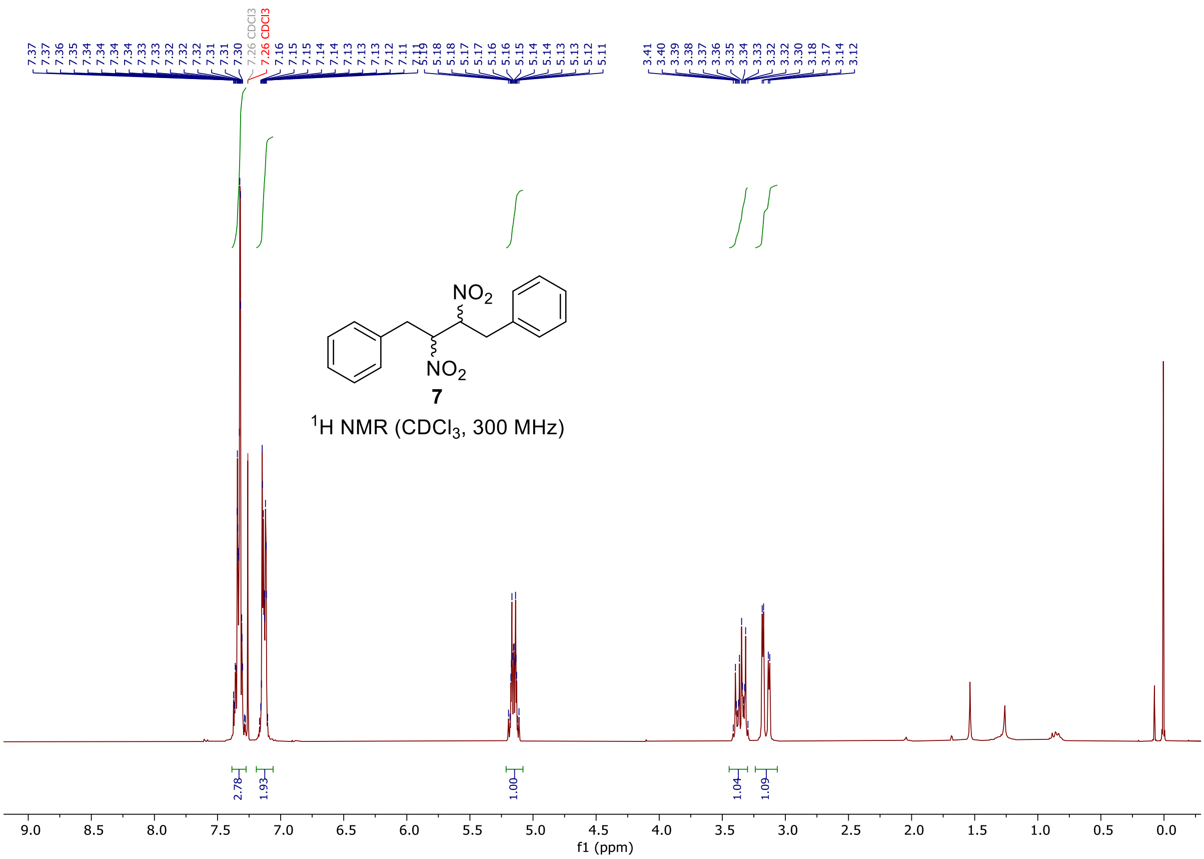

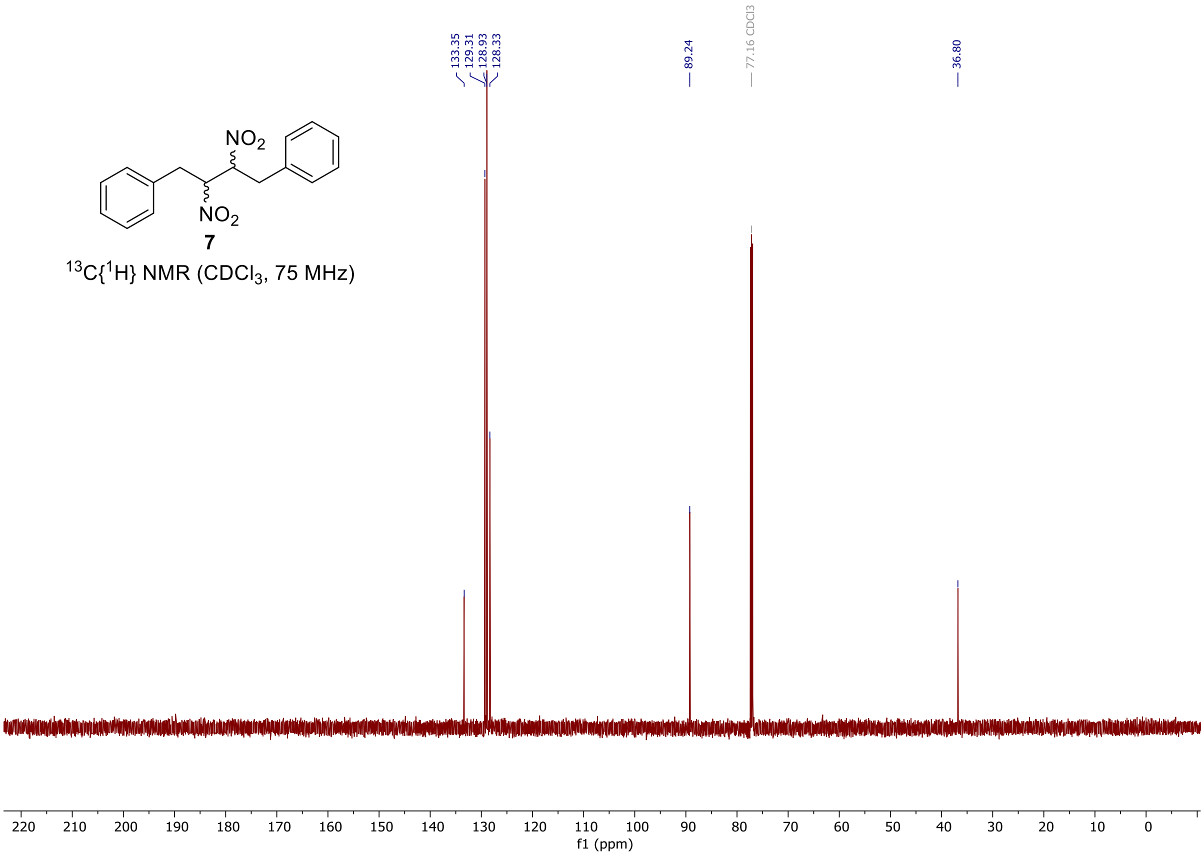

Figure S100

4.57 NMR spectra of 8a

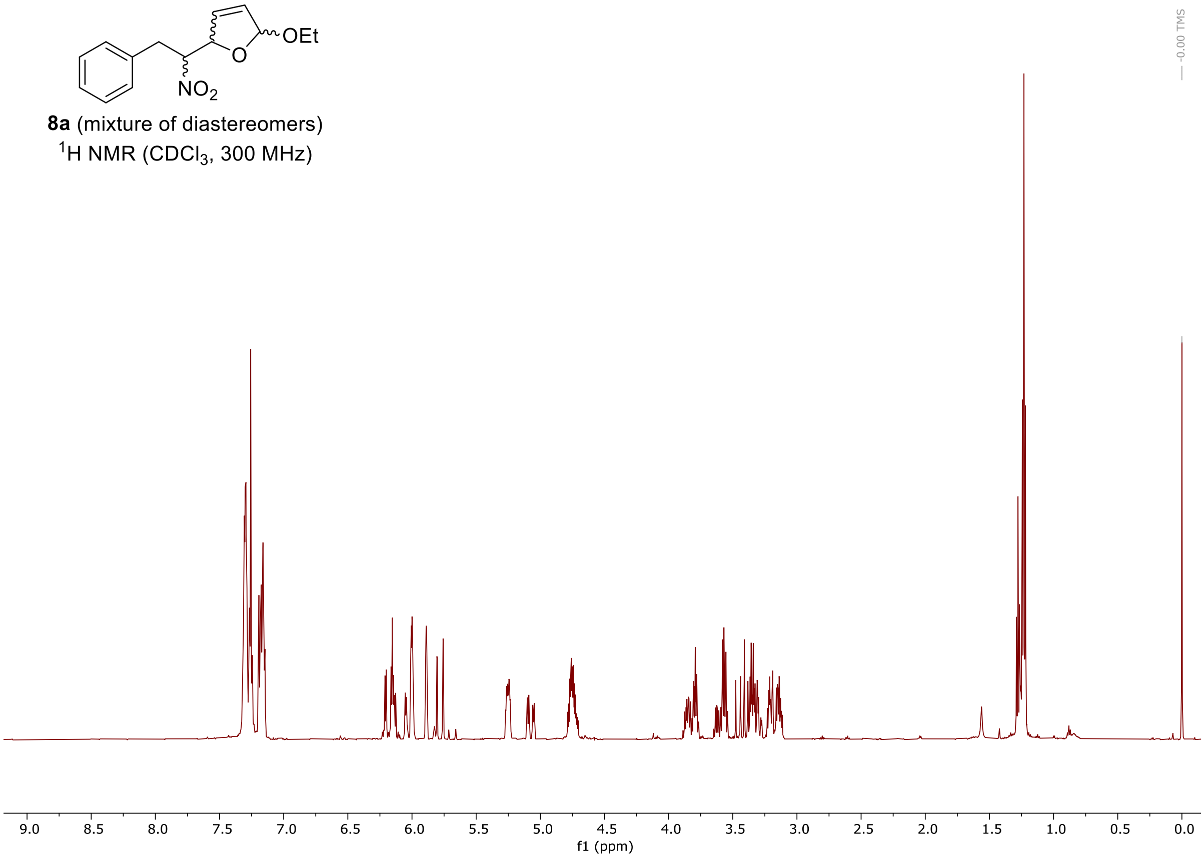

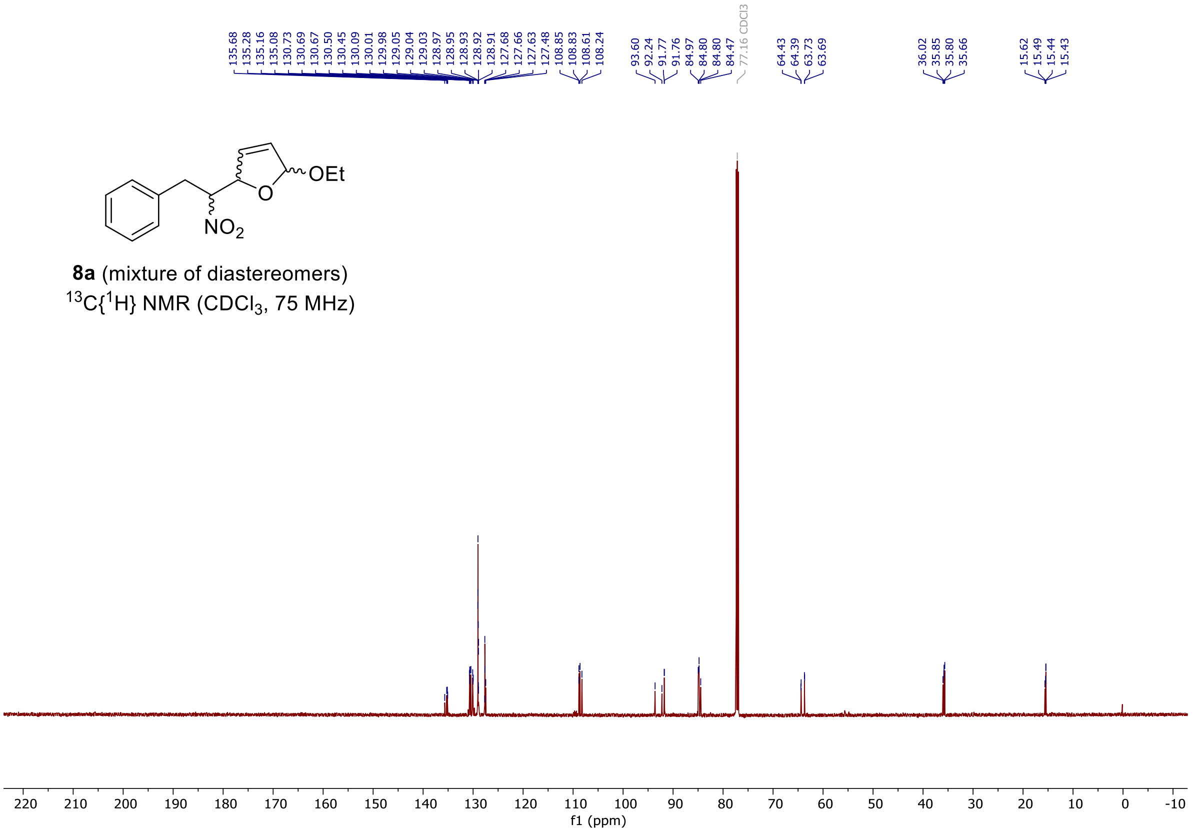

Figure S101

4.58 NMR spectra of 31

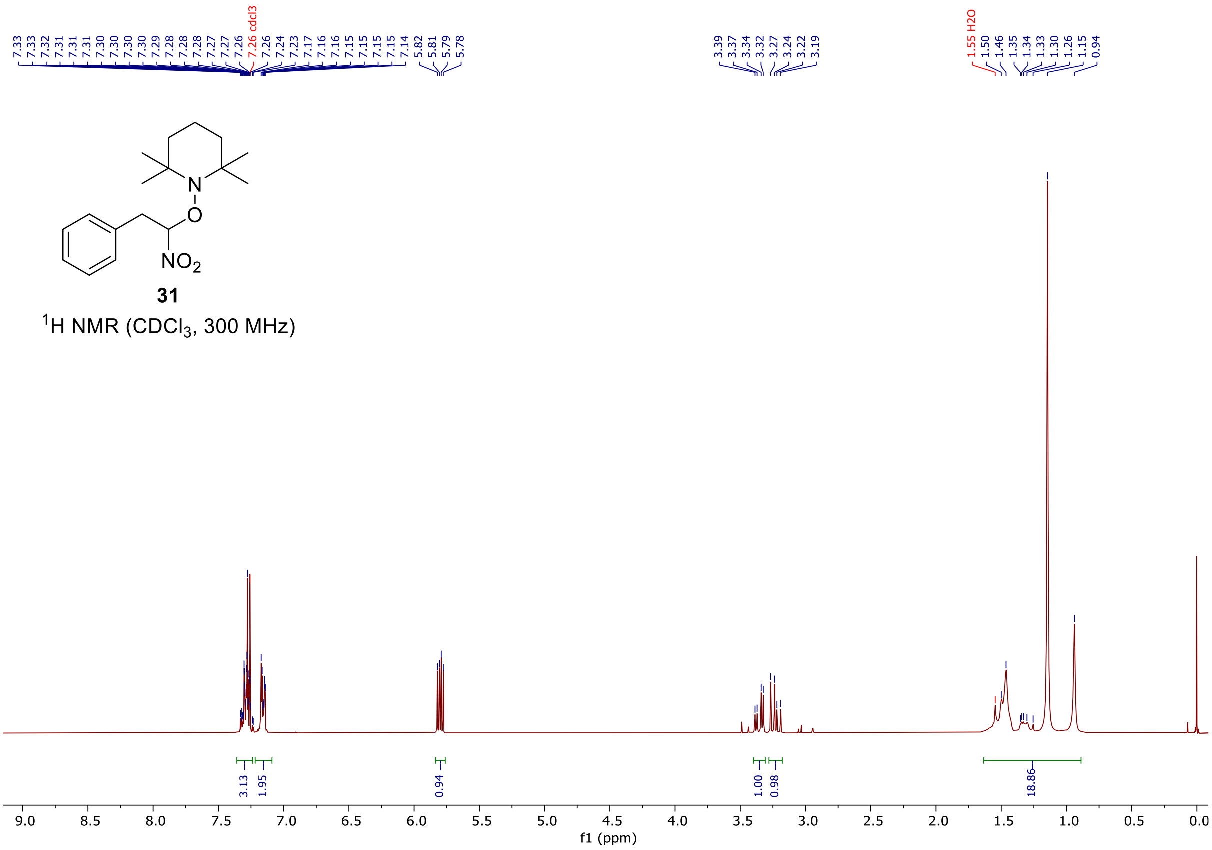

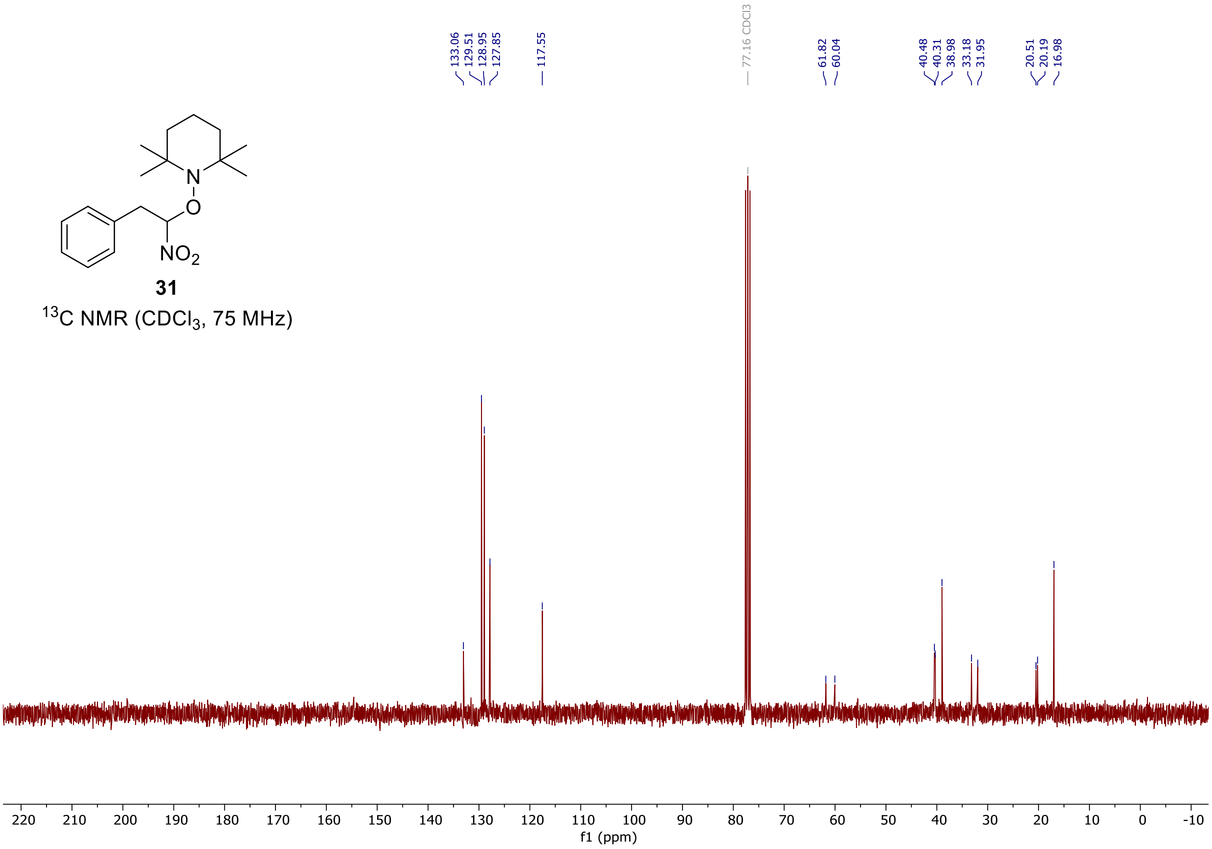

Figure S102

5. References

1. Marčeková, M.; Gerža, P.; Šoral, M.; Moncol, J.; Berkeš, D.; Kolarovič, A.; Jakubec, P. Visible-Light-Promoted Cross-Coupling of N-Alkylpyridinium Salts and Nitrostyrenes. *Org. Lett.* **2019**, *21,* 4580–4584.
2. Marsh, G. P.; Parsons, P. J.; McCarthy, C.; Corniquet, X. G. An Efficient Synthesis of Nitroalkenes by Alkene Cross Metathesis: Facile Access to Small Ring Systems *Org. Lett.* **2007**, *9,* 2613–2616.
3. Barber, D. M.; Sanganee, H. J.; Dixon, D. J. One-Pot Catalytic Enantioselective Synthesis of Tetrahydropyridines via a Nitro-Mannich/Hydroamination Cascade. *Org. Lett.* **2012**, *14*, 5290–5293.
4. Barber, D. M.; Sanganee, H.; Dixon, D. J. One-pot nitro-Mannich/hydroamination cascades for the direct synthesis of 2,5-disubstituted pyrroles using base and gold catalysis. *Chem. Commun.* **2011**, *47*, 4379–4381.
5. Ambros, R.; Schneider, M. R.; Von Angerer, S. Indolo[2,1-a]isoquinolines. Syntheses, steroid hormone receptor binding affinities, and cytostatic activity. *J. Med. Chem.* **1990**, *33*, 153–160.
6. Gregory, A. W.; Chambers, A.; Hawkins, A.; Jakubec, P.; Dixon, D. J. Iridium-Catalyzed Reductive Nitro-Mannich Cyclization. *Chem. Eur. J.* **2015**, *21*, 111–114.
7. Kim, R. S.; Dinh-Nguyen, L. V.; Shimkin, K. W.; Watson, D. A. Copper-Catalyzed Propargylation of Nitroalkanes. *Org. Lett.* **2020**, *22*, 8106–8110.
8. Xiang, J.; Sun, E.-X.; Lian, C.-X.; Yuan, W.-C.; Zhu, J.; Wang, Q. The highly chemoselective transfer hydrogenation of the carbon–carbon double bond of conjugated nitroalkenes by a rhodium complex. *Tetrahedron* **2012**, *68*, 4609–4620.
9. Antonova, Y. A.; Ioffe, S. L.; Sukhorukov, A. Y.; Tabolin, A. A. *Eur. J. Org. Chem.* **2021**, *2021*, 3197.
10. Wakamatsu, S.; Shimo, K. Solvent-Catalyzed Michael Reaction of Nitroparaffins with Acrylic Acid Derivatives in Liquid Ammonia. *J. Org. Chem.* **1962**, *27*, 1609–1611.
11. Rezazadeh, S.; Devannah, V.; Watson, D. A. Nickel-Catalyzed C-Alkylation of Nitroalkanes with Unactivated Alkyl Iodides *J. Am. Chem. Soc.* **2017**, *139*, 8110–8113.
12. Shi, W.-Y.; Ding, Y.-N.; Zheng, N.; Gou, X.-Y.; Zhang, Z.; Chen, X.; Luan, Y.-Y.; Niu, Z.-J.; Liang, Y.-M. Highly regioselective and stereoselective synthesis of C-Aryl glycosides via nickel-catalyzed ortho-C–H glycosylation of 8-aminoquinoline benzamides. *Chem. Commun.* **2021**, *57*, 8945–8948.
13. Li, F.; Zhang, Y.; Kwon, S. R.; Lutkenhaus, J. L. Electropolymerized Polythiophenes Bearing Pendant Nitroxide Radicals. Electropolymerized Polythiophenes Bearing Pendant Nitroxide Radicals. *ACS Macro Lett.* **2016**, *5*, 337–341.
14. Bharathiraja, G.; Sakthivel, S.; Sengoden, M.; Punniyamurthy, T. A Novel Tandem Sequence to Pyrrole Syntheses by 5-endo-dig Cyclization of 1,3-Enynes with Amines. *Org. Lett.* **2013**, *15*, 4996–4999.
15. Ganesh, M.; Namboothiri, I. N. N. Stereospecific approach to α,β-disubstituted nitroalkenes via coupling of α-bromonitroalkenes with boronic acids and terminal acetylenes. *Tetrahedron* **2007**, *63*, 11973–11983.
16. Najibi, A.; Goerigk, L. DFT-D4 counterparts of leading meta-generalized-gradient approximation and hybrid density functionals for energetics and geometries. *J. Comput. Chem.* **2020**, *41*, 2562–2572.
17. Mardirossian, N.; Head-Gordon, M. ωB97X-V: A 10-parameter, range-separated hybrid, generalized gradient approximation density functional with nonlocal correlation, designed by a survival-of-the-fittest strategy. *Phys. Chem. Chem. Phys.* **2014**, *16*, 9904–9924.
18. Weigend, F.; Ahlrichs, R. Balanced basis sets of split valence, triple zeta valence and quadruple zeta valence quality for H to Rn: Design and assessment of accuracy. *Phys. Chem. Chem. Phys.* **2005**, 7, 3297–3305.

19. Marenich, A. V.; Cramer, C. J.; Truhlar, D. G. Universal Solvation Model Based on Solute Electron Density and on a Continuum Model of the Solvent Defined by the Bulk Dielectric Constant and Atomic Surface Tensions. *J. Phys. Chem. B* **2009**, *113*, 6378–6396.
20. de Souza, B. GOAT: A Global Optimization Algorithm for Molecules and Atomic Clusters. *Angew. Chem. Int. Ed.* **2025**, *64*, e202500393.
21. Bannwarth, C.; Ehlert, S.; Grimme, S. GFN2-xTB—An Accurate and Broadly Parametrized Self-Consistent Tight-Binding Quantum Chemical Method with Multipole Electrostatics and Density-Dependent Dispersion Contributions. *J. Chem. Theory Comp.* **2019**, *15*, 1652–1671.
22. Neese, F. Software update: The ORCA program system—Version 5.0. WIREs CMS **2022**, *12*, e1606.
23. Nair, V.; Deepthi, A. Cerium(IV) Ammonium Nitrate - A Versatile Single-Electron Oxidant. *Chem. Rev.* **2007**, *107*, 1862–1891.
